# Large-scale analysis of structural brain asymmetries in schizophrenia via the ENIGMA consortium

**DOI:** 10.1073/pnas.2213880120

**Published:** 2023-03-28

**Authors:** Dick Schijven, Merel C. Postema, Masaki Fukunaga, Junya Matsumoto, Kenichiro Miura, Sonja M. C. de Zwarte, Neeltje E. M. van Haren, Wiepke Cahn, Hilleke E. Hulshoff Pol, René S. Kahn, Rosa Ayesa-Arriola, Víctor Ortiz-García de la Foz, Diana Tordesillas-Gutierrez, Javier Vázquez-Bourgon, Benedicto Crespo-Facorro, Dag Alnæs, Andreas Dahl, Lars T. Westlye, Ingrid Agartz, Ole A. Andreassen, Erik G. Jönsson, Peter Kochunov, Jason M. Bruggemann, Stanley V. Catts, Patricia T. Michie, Bryan J. Mowry, Yann Quidé, Paul E. Rasser, Ulrich Schall, Rodney J. Scott, Vaughan J. Carr, Melissa J. Green, Frans A. Henskens, Carmel M. Loughland, Christos Pantelis, Cynthia Shannon Weickert, Thomas W. Weickert, Lieuwe de Haan, Katharina Brosch, Julia-Katharina Pfarr, Kai G. Ringwald, Frederike Stein, Andreas Jansen, Tilo T. J. Kircher, Igor Nenadić, Bernd Krämer, Oliver Gruber, Theodore D. Satterthwaite, Juan Bustillo, Daniel H. Mathalon, Adrian Preda, Vince D. Calhoun, Judith M. Ford, Steven G. Potkin, Jingxu Chen, Yunlong Tan, Zhiren Wang, Hong Xiang, Fengmei Fan, Fabio Bernardoni, Stefan Ehrlich, Paola Fuentes-Claramonte, Maria Angeles Garcia-Leon, Amalia Guerrero-Pedraza, Raymond Salvador, Salvador Sarró, Edith Pomarol-Clotet, Valentina Ciullo, Fabrizio Piras, Daniela Vecchio, Nerisa Banaj, Gianfranco Spalletta, Stijn Michielse, Therese van Amelsvoort, Erin W. Dickie, Aristotle N. Voineskos, Kang Sim, Simone Ciufolini, Paola Dazzan, Robin M. Murray, Woo-Sung Kim, Young-Chul Chung, Christina Andreou, André Schmidt, Stefan Borgwardt, Andrew M. McIntosh, Heather C. Whalley, Stephen M. Lawrie, Stefan du Plessis, Hilmar K. Luckhoff, Freda Scheffler, Robin Emsley, Dominik Grotegerd, Rebekka Lencer, Udo Dannlowski, Jesse T. Edmond, Kelly Rootes-Murdy, Julia M. Stephen, Andrew R. Mayer, Linda A. Antonucci, Leonardo Fazio, Giulio Pergola, Alessandro Bertolino, Covadonga M. Díaz-Caneja, Joost Janssen, Noemi G. Lois, Celso Arango, Alexander S. Tomyshev, Irina Lebedeva, Simon Cervenka, Carl M. Sellgren, Foivos Georgiadis, Matthias Kirschner, Stefan Kaiser, Tomas Hajek, Antonin Skoch, Filip Spaniel, Minah Kim, Yoo Bin Kwak, Sanghoon Oh, Jun Soo Kwon, Anthony James, Geor Bakker, Christian Knöchel, Michael Stäblein, Viola Oertel, Anne Uhlmann, Fleur M. Howells, Dan J. Stein, Henk S. Temmingh, Ana M. Diaz-Zuluaga, Julian A. Pineda-Zapata, Carlos López-Jaramillo, Stephanie Homan, Ellen Ji, Werner Surbeck, Philipp Homan, Simon E. Fisher, Barbara Franke, David C. Glahn, Ruben C. Gur, Ryota Hashimoto, Neda Jahanshad, Eileen Luders, Sarah E. Medland, Paul M. Thompson, Jessica A. Turner, Theo G. M. van Erp, Clyde Francks

**Affiliations:** ^a^Language & Genetics Department, Max Planck Institute for Psycholinguistics, Nijmegen 6525 XD, The Netherlands; ^b^Department of Neurology, Alzheimer Center Amsterdam, Amsterdam Neuroscience, Amsterdam UMC, Vrije Universiteit Amsterdam, Amsterdam 1081 HZ, The Netherlands; ^c^Division of Cerebral Integration, National Institute for Physiological Sciences, Okazaki 444-8585, Japan; ^d^Department of Pathology of Mental Diseases, National Center of Neurology and Psychiatry, National Institute of Mental Health, Tokyo 187-8551, Japan; ^e^Department of Psychiatry, University Medical Center Utrecht Brain Center, University Medical Center Utrecht, Utrecht University, Utrecht 3584 CG, The Netherlands; ^f^Department of Child and Adolescent Psychiatry/Psychology, Erasmus University Medical Center Sophia Children's Hospital, Rotterdam 3015 CN, The Netherlands; ^g^Department of Psychiatry, Icahn School of Medicine at Mount Sinai, New York, NY 10029; ^h^The Mental Illness Research, Education and Clinical Centers, James J. Peters VA Medical Center, New York, NY 10468; ^i^Department of Psychiatry, Instituto de Investigación Marqués de Valdecilla, University Hospital Marqués de Valdecilla, Santander 39008, Spain; ^j^Centro de Investigación Biomédica en Red de Salud Mental, Instituto de Salud Carlos III, Madrid 28029, Spain; ^k^Department of Medicine and Psychiatry, School of Medicine, University of Cantabria, Santander 39011, Spain; ^l^Department of Psychiatry, Marqués de Valdecilla University Hospital, Instituto de Investigación Sanitaria Valdecilla, School of Medicine, University of Cantabria, Santander 39011, Spain; ^m^Department of Radiology, Instituto de Investigación Marqués de Valdecilla, Marqués de Valdecilla University Hospital, Santander 39011, Spain; ^n^Advanced Computing and e-Science, Instituto de Física de Cantabria, Universidad de Cantabria - Consejo Superior de Investigaciones Científicas, Santander 39005, Spain; ^o^Department of Psychiatry, School of Medicine, University of Sevilla, University Hospital Virgen del Rocío, Consejo Superior de Investigaciones Científicas - Instituto de Biomedicina de Sevilla, Sevilla 41013, Spain; ^p^Norwegian Centre for Mental Disorders Research, Institute of Clinical Medicine, University of Oslo, Oslo 0450, Norway; ^q^Department of Psychology, University of Oslo, Oslo 0373, Norway; ^r^Bjørknes College, Oslo 0456, Norway; ^s^Division of Mental Health and Addiction, Oslo University Hospital, Oslo 0372, Norway; ^t^KG Jebsen Center for Neurodevelopmental Disorders, University of Oslo, Oslo 0450, Norway; ^u^Department of Psychiatric Research, Diakonhjemmet Hospital, Oslo 0373, Norway; ^v^Centre for Psychiatry Research, Department of Clinical Neuroscience, Karolinska Institutet & Stockholm Health Care Services, Region Stockholm, Stockholm 113 64, Sweden; ^w^Department of Psychiatry, University of Maryland School of Medicine, Baltimore, MD 21201; ^x^School of Psychiatry, University of New South Wales, Sydney 2033, Australia; ^y^Neuroscience Research Australia, Sydney 2031, Australia; ^z^Edith Collins Centre (Translational Research in Alcohol, Drugs & Toxicology), Sydney Local Health District, Sydney 2050, Australia; ^aa^Specialty of Addiction Medicine, Central Clinical School, Faculty of Medicine and Health, University of Sydney, Sydney 2006, Australia; ^bb^School of Medicine, The University of Queensland, Brisbane 4006, Australia; ^cc^School of Psychological Sciences, University of Newcastle, Newcastle 2308, Australia; ^dd^Queensland Brain Institute, The University of Queensland, Brisbane 4072, Australia; ^ee^Queensland Centre for Mental Health Research, The University of Queensland, Brisbane 4076, Australia; ^ff^Centre for Brain and Mental Health Research, University of Newcastle, Newcastle 2308, Australia; ^gg^Priority Research Centre for Stroke and Brain Injury, University of Newcastle, Newcastle 2308, Australia; ^hh^Hunter Medical Research Institute, Newcastle 2305, Australia; ^ii^School of Biomedical Science and Pharmacy, Faculty of Health and Medicine, University of Newcastle, Newcastle 2308, Australia; ^jj^School of Medicine and Public Health, University of Newcastle, Newcastle 2308, Australia; ^kk^PRC for Health Behaviour, Hunter Medical Research Institute, Newcastle 2305, Australia; ^ll^Hunter New England Mental Health Service, Newcastle 2305, Australia; ^mm^Melbourne Neuropsychiatry Centre, Department of Psychiatry, University of Melbourne, Melbourne 3053, Australia; ^nn^Department of Neuroscience and Physiology, Upstate Medical University, Syracuse, NY 13210; ^oo^Early Psychosis Department, Department of Psychiatry, Amsterdam UMC (location AMC), Amsterdam 1105 AZ, The Netherlands; ^pp^Arkin Institute for Mental Health, Amsterdam 1033 NN, The Netherlands; ^qq^Department of Psychiatry and Psychotherapy, Philipps-Universität Marburg, Marburg 35039, Germany; ^rr^Center for Mind, Brain and Behavior, Marburg 35032, Germany; ^ss^Core-Facility Brainimaging, Faculty of Medicine, Philipps-Universität Marburg, Marburg 35032, Germany; ^tt^Department of General Psychiatry, Section for Experimental Psychopathology and Neuroimaging, Heidelberg University, Heidelberg 69115, Germany; ^uu^Department of Psychiatry, Perelman School of Medicine, University of Pennsylvania, Philadelphia, PA 19104; ^vv^Lifespan Brain Institute, University of Pennsylvania & Children's Hospital of Philadelphia, Philadelphia, PA 19104; ^ww^Center for Biomedical Image Computing and Analytics, Perelman School of Medicine, University of Pennsylvania, Philadelphia, PA 19104; ^xx^Department of Psychiatry and Neuroscience, University of New Mexico, Albuquerque, NM 87106; ^yy^Department of Psychiatry and Behavioral Sciences and Weill Institute for Neurosciences, University of California, San Francisco, CA 94143; ^zz^Mental Health Service, Veterans Affairs San Francisco Healthcare System, San Francisco, CA 94121; ^aaa^Department of Psychiatry and Human Behavior, University of California Irvine, Irvine, CA 92697; ^bbb^Psychology Department and Neuroscience Institute, Georgia State University, Atlanta, GA 30303; ^ccc^Tri-Institutional Center for Translational Research in Neuroimaging and Data Science, Georgia State University, Georgia Institute of Technology and Emory University, Atlanta, GA 30303; ^ddd^San Francisco VA Medical Center, University of California, San Francisco, CA 94121; ^eee^Long Beach VA Health Care System, Long Beach, CA 90822; ^fff^Beijing Huilongguan Hospital, Peking University Huilongguan Clinical Medical School, Beijing 100096, P.R. China; ^ggg^Chongqing University Three Gorges Hospital, Chongqing 404188, P.R. China; ^hhh^Division of Psychological and Social Medicine and Developmental Neurosciences, Translational Developmental Neuroscience Section, Technische Universität Dresden, University Hospital C.G. Carus, Dresden 01307, Germany; ^iii^Department of Child and Adolescent Psychiatry, Eating Disorder Treatment and Research Center, Technische Universität Dresden, Faculty of Medicine, University Hospital C.G. Carus, Dresden 01307, Germany; ^jjj^FIDMAG Germanes Hospitalàries Research Foundation, Barcelona 08035, Spain; ^kkk^Mental Health Research Networking Center (Ciber del Área de Salud Mental), Madrid 28029, Spain; ^lll^Benito Menni Complex Assistencial en Salut Mental, Barcelona 08830, Spain; ^mmm^Laboratory of Neuropsychiatry, Istituto di Ricovero e Cura a Carattere Scientifico Santa Lucia Foundation, Rome 00179, Italy; ^nnn^Menninger Department of Psychiatry and Behavioral Sciences, Baylor College of Medicine, Houston, TX 77030; ^ooo^Department of Psychiatry and Neuropsychology, School for Mental Health and Neuroscience, Maastricht University Medical Centre, Maastricht University, Maastricht 6229 ER, The Netherlands; ^ppp^Campbell Family Mental Health Institute, Centre for Addiction and Mental Health, Toronto M5S 2S1, Canada; ^qqq^Department of Psychiatry, University of Toronto, Toronto M5T 1R8, Canada; ^rrr^West Region, Institute of Mental Health, Singapore 539747, Singapore; ^sss^Yong Loo Lin School of Medicine, National University of Singapore, Singapore 119228, Singapore; ^ttt^Department of Psychosis Studies, Institute of Psychiatry, Psychology and Neuroscience, King's College London, London SE5 8AF, United Kingdom; ^uuu^Department of Psychological Medicine, Institute of Psychiatry, Psychology and Neuroscience, King's College London, London SE5 8AF, United Kingdom; ^vvv^Department of Psychiatry, Jeonbuk National University Medical School, Jeonju 54896, Republic of Korea; ^www^Research Institute of Clinical Medicine, Jeonbuk National University-Biomedical Research Institute, Jeonbuk National University Hospital, Jeonju 54896, Republic of Korea; ^xxx^Department of Psychiatry, University Psychiatric Clinics (Universitäre Psychiatrische Kliniken), University of Basel, Basel 4002, Switzerland; ^yyy^Department of Psychiatry and Psychotherapy, University of Lübeck, Lübeck 23562, Germany; ^zzz^Division of Psychiatry, Centre for Clinical Brain Sciences, University of Edinburgh, Edinburgh EH16 4SB, United Kingdom; ^aaaa^Department of Psychiatry, Faculty of Medicine and Health Sciences, Stellenbosch University, Stellenbosch 7505, South Africa; ^bbbb^Stellenbosch University Genomics of Brain Disorders Research Unit, South African Medical Research Council, Cape Town 7505, South Africa; ^cccc^Department of Psychiatry and Mental Health, Faculty of Health Sciences, University of Cape Town, Cape Town 7935, South Africa; ^dddd^Neuroscience Institute, University of Cape Town, Cape Town 7935, South Africa; ^eeee^Institute for Translational Psychiatry, Westfälische Wilhelms-Universität Münster, Münster 48149, Germany; ^ffff^The Mind Research Network, Albuquerque, NM 87106; ^gggg^Department of Education, Psychology, Communication, University of Bari Aldo Moro, Bari 70121, Italy; ^hhhh^Department of Basic Medical Science, Neuroscience and Sense Organs, University of Bari Aldo Moro, Bari 70121, Italy; ^iiii^Psychiatry Unit, Bari University Hospital, Bari 70121, Italy; ^jjjj^Department of Child and Adolescent Psychiatry, Institute of Psychiatry and Mental Health, Hospital General Universitario Gregorio Marañón, Madrid 28009, Spain; ^kkkk^Ciber del Área de Salud Mental, Instituto de Salud Carlos III, Madrid 28029, Spain; ^llll^Instituto de Investigación Sanitaria Gregorio Marañón, Madrid 28009, Spain; ^mmmm^School of Medicine, Universidad Complutense, Madrid 28040, Spain; ^nnnn^Laboratory of Neuroimaging and Multimodal Analysis, Mental Health Research Center, Moscow 115522, Russian Federation; ^oooo^Department of Medical Sciences, Psychiatry, Uppsala University, Uppsala 751 85, Sweden; ^pppp^Department of Physiology and Pharmacology, Karolinska Institutet, Stockholm 171 65, Sweden; ^qqqq^Department of Psychiatry, Psychotherapy and Psychosomatics, Psychiatric University Hospital Zurich (PUK), Zurich 8008, Switzerland; ^rrrr^Montreal Neurological Institute, McGill University, Montreal H3A 2B4, Canada; ^ssss^Department of Psychiatry, Division of Adult Psychiatry, Geneva University Hospitals, Geneva 1202, Switzerland; ^tttt^National Institute of Mental Health, Klecany 250 67, Czech Republic; ^uuuu^Department of Psychiatry, Dalhousie University, Halifax B3H 2E2, Canada; ^vvvv^MR Unit, Department of Diagnostic and Interventional Radiology, Institute for Clinical and Experimental Medicine, Prague 140 21, Czech Republic; ^wwww^Department of Neuropsychiatry, Seoul National University Hospital, Seoul 08826, Republic of Korea; ^xxxx^Department of Psychiatry, Seoul National University College of Medicine, Seoul 08826, Republic of Korea; ^yyyy^Department of Brain and Cognitive Sciences, Seoul National University College of Natural Sciences, Seoul 08826, Republic of Korea; ^zzzz^Department of Psychiatry, University of Oxford, Oxford OX3 7JX, United Kingdom; ^aaaaa^Department of Psychiatry, Psychosomatic Medicine and Psychotherapy, University Hospital Frankfurt, Frankfurt am Main 60528, Germany; ^bbbbb^Department of Child and Adolescent Psychiatry, Technische Universität Dresden, Dresden 01187, Germany; ^ccccc^SA MRC Unit on Risk & Resilience in Mental Disorders, University of Cape Town, Cape Town 7505, South Africa; ^ddddd^Department of Psychiatry, Research Group in Psychiatry (GIPSI), Faculty of Medicine, Universidad de Antioquia, Medellín 050010, Colombia; ^eeeee^Experimental Psychopathology and Psychotherapy, Department of Psychology, University of Zurich, Zurich 8050, Switzerland; ^fffff^Center for Psychiatric Neuroscience, Feinstein Institute for Medical Research, Manhasset, NY 11030; ^ggggg^Division of Psychiatry Research, Zucker Hillside Hospital, Northwell Health, New York, NY 11004; ^hhhhh^Department of Psychiatry, Zucker School of Medicine at Northwell/Hofstra, New York, NY 11549; ^iiiii^Donders Institute for Brain, Cognition and Behaviour, Radboud University, Nijmegen 6500 HB, The Netherlands; ^jjjjj^Department of Human Genetics, Radboud University Medical Center, Nijmegen 6525 GA, The Netherlands; ^kkkkk^Department of Psychiatry, Radboud University Medical Center, Nijmegen 6525 GA, The Netherlands; ^lllll^Department of Psychiatry, Boston Children's Hospital and Harvard Medical School, Boston, MA 02115; ^mmmmm^Olin Neuropsychiatry Research Center, Institute of Living, Hartford, CT 06102; ^nnnnn^Department of Radiology, Perelman School of Medicine, Philadelphia, PA 19104; ^ooooo^Department of Neurology, Perelman School of Medicine, Philadelphia, PA 19104; ^ppppp^Imaging Genetics Center, Mark & Mary Stevens Neuroimaging & Informatics Institute, Keck School of Medicine, University of Southern California, Los Angeles, CA 90033; ^qqqqq^School of Psychology, University of Auckland, Auckland 1010, New Zealand; ^rrrrr^Department of Women’s and Children’s Health, Uppsala University, Uppsala 752 37, Sweden; ^sssss^Laboratory of Neuro Imaging, School of Medicine, University of Southern California, Los Angeles, CA 90033; ^ttttt^Psychiatric Genetics, QIMR Berghofer Medical Research Institute, Brisbane 4006, Australia; ^uuuuu^Clinical Translational Neuroscience Laboratory, Department of Psychiatry and Human Behavior, University of California Irvine, Irvine, CA 92697; ^vvvvv^Center for the Neurobiology of Learning and Memory, University of California Irvine, Irvine, CA 92697

**Keywords:** Schizophrenia, brain imaging, asymmetry, cortical, subcortical

## Abstract

Left–right asymmetry is an important organizing feature of the healthy brain that may be altered in schizophrenia, but most studies have used relatively small samples and heterogeneous approaches, resulting in equivocal findings. We carried out the largest case–control study of structural brain asymmetries in schizophrenia, with MRI data from 5,080 affected individuals and 6,015 controls across 46 datasets, using a single image analysis protocol. Asymmetry indexes were calculated for global and regional cortical thickness, surface area, and subcortical volume measures. Differences of asymmetry were calculated between affected individuals and controls per dataset, and effect sizes were meta-analyzed across datasets. Small average case–control differences were observed for thickness asymmetries of the rostral anterior cingulate and the middle temporal gyrus, both driven by thinner left-hemispheric cortices in schizophrenia. Analyses of these asymmetries with respect to the use of antipsychotic medication and other clinical variables did not show any significant associations. Assessment of age- and sex-specific effects revealed a stronger average leftward asymmetry of pallidum volume between older cases and controls. Case–control differences in a multivariate context were assessed in a subset of the data (N = 2,029), which revealed that 7% of the variance across all structural asymmetries was explained by case–control status. Subtle case–control differences of brain macrostructural asymmetry may reflect differences at the molecular, cytoarchitectonic, or circuit levels that have functional relevance for the disorder. Reduced left middle temporal cortical thickness is consistent with altered left-hemisphere language network organization in schizophrenia.

## Significance

Schizophrenia has been proposed to involve altered left-hemispheric dominance for language in the brain, but research in limited sample sizes has not clarified whether structural asymmetry differs in this condition. In MRI data from 5,080 affected individuals and 6,015 controls, we found altered asymmetry of two brain regions driven by thinner left-hemisphere cortex in schizophrenia: the rostral anterior cingulate and middle temporal gyrus. The latter is a core region of the left-hemisphere language network. Effects were very small in terms of macroanatomical asymmetry, but might be compatible with altered lateralized function. Across all brain regions considered together, 7% of variance in asymmetry was linked to case–control status, indicating a more diffuse pattern of subtly altered anatomical asymmetry.

Schizophrenia is a serious mental illness characterized by various combinations of symptoms that may include delusions, hallucinations, disorganized speech, affective flattening, avolition, and executive function deficits (1). Left–right asymmetry is an important feature of human brain organization for diverse cognitive functions—for example, roughly 90% of people present with a left-hemisphere dominance for language and right-handedness (2–5). A possible role of altered structural and functional brain asymmetry in schizophrenia has been studied for several decades (6–10). Theoretical work has especially focused on disrupted laterality for language in relation to disorganized speech perception and production—the former may sometimes result in auditory verbal hallucinations which are a relatively prevalent symptom (11–14). Individuals with schizophrenia have been reported to show decreased left-lateralized language dominance (15, 16), as well as an absence or even reversal of structural asymmetries of language-related regions around the Sylvian fissure (which divides the temporal lobe from the frontal and parietal lobes) (13, 17–19). Language disturbances such as idiosyncratic semantic associations or reduced grammatical complexity are also commonly reported (20). Furthermore, the rate of nonright-handedness in schizophrenia is elevated compared to that of the general population (13, 21–25). Interestingly, some genomic loci that influence aspects of structural brain asymmetry or hand preference overlap with those associated with schizophrenia (26–29). Thus, there might be an etiological link between altered brain asymmetry and schizophrenia.

However, alterations in structural asymmetry of the cerebral cortex in schizophrenia have so far only been reported in studies with relatively small samples (13, 17–19, 30–36); to our knowledge, the largest case–control sample consisted of 167 affected individuals and 159 controls (33). Many of the existing findings are inconsistent and/or remain unreplicated, which is possibly due to low statistical power which limits the sensitivity to detect true effects and also increases the risk of overestimating effect sizes (37–39). The reproducibility of findings may be further affected by the heterogeneity of clinical and demographic characteristics across studies. Moreover, varying approaches to process and analyze MRI data limit the possibility to reproduce results and/or to perform meta-analyses. For example, in studies targeting specific regions of interest, regions have been inconsistently defined, while studies that involved cortex-wide mapping used different image analysis protocols. Studies of subcortical volumetric asymmetries in schizophrenia have generally suffered from similar issues (40–42), with the notable exception of a study in 884 affected individuals and 1,680 controls that used a single image analysis pipeline (43). This study found an increased leftward asymmetry of the pallidum in schizophrenia (driven by a larger pallidum volume in the left hemisphere) compared to controls, which was also detectable in adolescents with subclinical psychotic experiences (43, 44).

The Enhancing Neuro Imaging Genetics through Meta-Analysis (ENIGMA, http://enigma.ini.usc.edu) consortium aims to perform large-scale analyses by combining imaging data from research groups across the world, processed with standardized protocols (45, 46). Previously, this consortium reported large-scale cortical thinning, smaller surface area, and altered subcortical volume in individuals with schizophrenia compared to controls (47, 48). However, asymmetry was not measured in these previous ENIGMA studies, and no tests were performed to assess whether case–control effects were different in the two hemispheres. The ENIGMA consortium has investigated structural brain asymmetries in other disorders (49): major depressive disorder (50), autism spectrum disorder (ASD) (51), obsessive compulsive disorder (OCD) (52), and attention deficit/hyperactivity disorder (ADHD) (53). Case–control group-level effects were small for all of these disorders, with ASD showing the most widespread asymmetry differences—mostly involving regional cortical thickness measures—with a maximum Cohen’s *d* of 0.13 (51). Similar effect sizes may be anticipated for schizophrenia. Therefore, a large sample size is likely required to detect and accurately measure any effects. Although small group-average differences of brain macroanatomy are unlikely to have clinical uses by themselves, they may help to identify brain regions and networks that have clinically relevant disruptions at other neurobiological levels—for example molecular or cytoarchitectonic—which can be investigated in future studies. Of note, the ENIGMA consortium has recently reported on asymmetry alterations with respect to subcortical *shape* (2,833 individuals with schizophrenia versus 3,929 controls), based on an automated approach quantifying local concave versus convex surface curvature (54), but that study did not address subcortical *volume* asymmetries, and omitted the cerebral cortex.

For the current study, we were able to measure both cortical and subcortical structural asymmetries in schizophrenia using by far the largest sample to date: 5,080 affected individuals and 6,015 controls, from 46 separate datasets. The datasets were collected originally as distinct studies over approximately 25 years, using different recruitment schemes, MRI scanning equipment, and parameters. Importantly, for the current study, all primary MRI data were processed through a single pipeline for cortical atlas-based segmentation/subcortical parcellation and quality control.

Given previous theoretical and empirical work linking schizophrenia to reduced language laterality and function (see above), we had a particular interest in whether typical structural asymmetries of the core cerebral cortical language network might be reduced in schizophrenia—this includes asymmetries of lateral temporal cortex and inferior frontal cortex (55). However, linguistic tasks can also recruit various other brain regions (56), while disrupted cognition in schizophrenia affects multiple domains beyond language (1). Our primary aim was therefore to map potentially altered structural asymmetry in schizophrenia across all cortical and subcortical regions, for a thorough and unconstrained mapping of brain asymmetry in schizophrenia, supported by our unprecedented sample size. We achieved this through separate region-by-region testing of case–control group average differences in asymmetry (followed by false discovery rate (FDR) correction), where the testing was two tailed, i.e., we allowed for either reductions, increases, or even reversals of asymmetry in affected individuals compared to controls. Due to restrictions on sharing individual-level data for many of the primary datasets, case–control differences were first tested for each regional asymmetry index (AI) separately within each dataset, and effects were then combined across datasets using meta-analysis methodology.

We also performed various secondary/exploratory analyses of the data. We explored possible associations of structural brain asymmetries with medication use and other disorder-specific measures: age at onset; duration of illness; as well as total, positive, and negative symptom scores. In addition, we tested age- and sex-specific asymmetry differences. Finally, for 14 datasets for which individual-level data were available, we tested for a multivariate association of case–control status simultaneously with regional AIs across the brain.

Together, these analyses aimed to provide insights into the extent and mapping of structural brain asymmetry alterations in schizophrenia, and how they relate to key clinical variables.

## Methods and Materials

### Datasets.

Structural MRI data were derived from 46 separate datasets (45 case–control and one case-only) totaling 11,095 individuals, via researcher participation in the ENIGMA schizophrenia working group. Of these, 5,080 were affected with schizophrenia and 6,015 were unaffected controls (*SI Appendix*, Table S1*A*). The datasets came from various countries around the world and were collected over the last roughly 25 y (*SI Appendix*, Fig. S1). For each of the datasets, all relevant local ethical regulations were complied with, and appropriate informed consent was obtained for all individuals. The present study was carried out under approval from the Ethics Committee of the Faculty of Social Sciences of Radboud University Nijmegen. Sample size-weighted mean age across datasets was 33.3 (range 16.2 to 44.0) years for individuals with schizophrenia and 33.0 (11.8 to 43.6) years for controls. Affected individuals and controls were 67% and 52% males, respectively. Diagnostic interviews were conducted by registered clinical research staff using different diagnostic criteria (either the Diagnostic and Statistical Manual of Mental Disorders [DSM]-III, DSM-IV, DSM-5 or International Classification of Diseases-10) (*SI Appendix*, Table S2). No controls had present or past indications of schizophrenia.

### Image Acquisition, Processing, and Quality Control.

T1-weighted structural brain MRI scans were acquired at each study site. Dataset-specific scanner information, field strengths, and image acquisition parameters are provided in *SI Appendix*, Table S2. For data from all sites, image processing and segmentation were performed using FreeSurfer (see *SI Appendix*, Table S2 for software versions) (57). For each individual, using the “recon-all” pipeline, cerebral cortical thickness and surface area measures were derived for 34 bilaterally paired Desikan–Killiany (DK) atlas regions, as well as whole hemisphere-level average cortical thickness and surface area measures (58). Volumes for 8 bilaterally paired regions from a neuroanatomical atlas of brain subcortical structures (59) were derived using the “aseg” segmentation command in FreeSurfer. A standardized ENIGMA quality control procedure was applied at each participating site (described in full here: http://enigma.ini.usc.edu/protocols/imaging-protocols/). Briefly, this included outlier detection in the derived cortical and subcortical measures and visual inspection of segmentations projected onto the T1-weighted image of each individual. Predefined guidelines for visual inspection were followed. Measurements from regions with poor segmentation were excluded, as well as individuals whose data failed overall quality checks. Data-sharing limitations did not allow the central analysis group to have access to individual-level data for the majority of participating study sites. For further processing and analyses of the data, a script running in R software (R Foundation for Statistical Computing, Vienna, Austria, www.R-project.org) (60) was prepared and distributed among participating sites, to ensure coordinated collection of descriptive and summary statistics for subsequent meta-analysis by the central analysis team.

### Asymmetry Index Calculation.

For each bilaterally paired brain regional measure, we used the left (*L*) and right (*R*) hemispheric measurements to calculate $AI=\frac{L-R}{\left(L+R\right)/2}$ , where the denominator corrects for automatic scaling of the index with the magnitude of the bilateral measure. This formula for AI calculation has been widely used (2, 52, 61–63). A negative value of the AI reflects a larger right hemispheric measurement (*R* > *L*) and a positive value a larger left hemispheric measurement (*L* > *R*). Left or right measurements equal to 0 were set to missing, as these most likely reflected data entry errors. Furthermore, when a left or right measurement was missing, the corresponding measurement in the opposite hemisphere was also set to missing. Calculated AIs were used for additional quality control of image orientation in each dataset (Supporting Information 1, *SI Appendix*, Table S3).

### Asymmetry Differences between Individuals with Schizophrenia and Unaffected Controls.

Group differences were examined separately for each brain regional AI and each case–control dataset, using univariate linear regression implemented in R. Our primary analysis model included diagnosis (case–control status) as the main binary predictor, and sex and age as covariates (model 1 in Supporting Information 2). For ten datasets where more than one scanner had been used (*SI Appendix*, Table S2), we added *n*-1 binary dummy covariates (where *n* is the number of scanners in a given dataset), to statistically control for scanner effects. Collinearity between predictor variables was assessed using the R-package *usdm* (v1-1.18) (64), and high collinearity (variance inflation factor > 5) was not found for any dataset. Linear regression analysis for any structural AI was not performed if the total sample size of a given dataset was lower than ten plus the number of scanner covariates, or if one of the diagnostic groups had a sample size lower than five. For each brain regional AI and each case–control dataset, we extracted the *t*-statistic for the “diagnosis” term to calculate its corresponding Cohen’s *d* effect size, SE, and 95% CI, using $d=\frac{t\left(n_{1}+n_{2}\right)}{\sqrt{n_{1}n_{2}}\sqrt{df}}$ , $\begin{array}{c} {\mathrm{se}}_{d}=\sqrt{\left(\frac{n_{1}+n_{2}-1}{n_{1}+n_{2}-3}\right)\left[\left(\frac{4}{n_{1}+n_{2}}\right)\left(1+\frac{d^{2}}{8}\right)\right]\;} \end{array}$ ,and $\begin{array}{c} 95\%\;CI\;=\;\left[d-1.96\times se_{d},\;d+1.96\times se_{d}\right] \end{array}$ (65). In these equations, *d* is the Cohen’s *d* effect size, *t* is the *t*-statistic, *se* is the SE, *n*_1_ is the number of unaffected controls, *n*_2_ is the number of individuals with schizophrenia, and *df* is the degrees of freedom in the linear model.

### Random-Effects Meta-analysis.

For each brain regional AI (*SI Appendix*, Figs. S2–S4), effect sizes for diagnosis from each case–control dataset were meta-analyzed in a random-effects model fitted with a restricted maximum likelihood estimator, using the function “rma” in the R package *metafor* (v3.0-2) (66). The meta-analyzed effect sizes were projected on 3D meshes of inflated cortical or subcortical models from Brainder (www.brainder.org/research/brain-for-blender/), using Matlab R2020a (version 9.8.0.1323502; MathWorks, Natick, MA, USA). We calculated false discovery rate (FDR)-corrected *P* values using the Benjamini–Hochberg method to account for multiple tests (67) (i.e., separately for testing 35 cortical thickness AIs, 35 cortical surface area AIs, and eight subcortical volume AIs). Effects with *p*_FDR_ < 0.05 were considered statistically significant. For AIs that showed significant group differences between cases and controls, the group differences for the corresponding left and right measurements separately were also assessed post hoc (again using linear modeling with diagnosis, age, and sex as predictors), to help describe the asymmetry differences.

### Sensitivity and Secondary Analyses.

For any AI that showed a significant case–control group difference in the primary meta-analysis, we carried out various sensitivity and secondary analyses as detailed in Supporting Information 3. The sensitivity analyses assessed the robustness of effects with respect to: 1) Individual datasets with “outlier” effects. 2) Heterogeneity of technical, diagnostic, or geographic differences between datasets. 3) Handedness, intracranial volume, or nonlinear age effects. Secondary analyses assessed medication group differences and correlations of asymmetries with clinical variables (in affected individuals only). In addition, for all AIs in all case–control datasets, we applied models which were the same as the primary analysis but also included either diagnosis-by-age or diagnosis-by-sex interaction terms.

### Multivariate Analysis of Case–Control Asymmetry Differences.

To examine case–control group differences across all brain regional AIs simultaneously in one model, we conducted a multivariate analysis based on 14 datasets for which individual-level data were available to the central analysis team. For this analysis, we only retained individuals with complete data for all bilateral measures of cortical and subcortical structures, which were 935 individuals affected with schizophrenia and 1,095 unaffected controls (*SI Appendix*, Table S1*C*). We separately adjusted the left and right measurements using ComBat harmonization (an empirical Bayesian method) to remove dataset effects (68), where each dataset (and each scanner within multiscanner datasets) was treated as a distinct “batch.” Diagnosis, age, and sex were used as covariates when finding the data harmonization parameters in ComBat. After ComBat adjustment, one additional control individual was removed due to being assigned a negative corrected right hemisphere lateral ventricle volume (*SI Appendix*, Fig. S5). AIs for cortical and subcortical measures were then calculated using the same formula as above, and collinearity between AIs was assessed by calculating a correlation matrix. AIs did not show higher pair-wise correlations than 0.5 (*SI Appendix*, Figs. S6 and S7). A multivariate analysis of covariance (MANCOVA) using the “manova” function in R was applied, testing all 76 regional structural brain AIs simultaneously against case–control status, with age and sex as covariates. We ran one million label-swapping permutations of case–control labels and calculated a permutation p-value by assessing the number of times the F-statistic of an analysis with permuted data was equal to or larger than the F-statistic of the analysis with real data, divided by the total number of permutations. When permuting case–control labels, we conserved case–control numbers within each dataset (and within scanner for multiscanner datasets). To help interpret the MANCOVA results, we also derived univariate case–control association statistics for each separate structural AI from the multivariate association analysis output, using univariate analysis of covariance (ANCOVA) (“summary.aov” function in R).

## Results

### Asymmetry Differences between Individuals with Schizophrenia and Unaffected Controls.

**Fig. 1.**
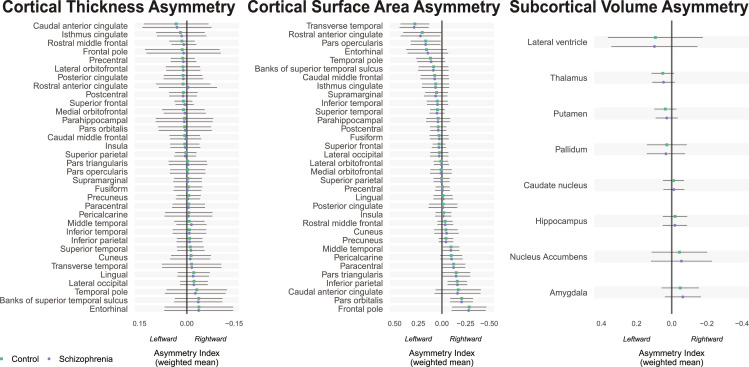
Average structural asymmetries of the brain in individuals with schizophrenia and unaffected controls. For each bilaterally paired structural measure, the mean asymmetry index (AI) across datasets, weighted by sample size, is shown for individuals with schizophrenia (purple) and unaffected controls (green). A positive AI indicates left > right asymmetry, whereas a negative AI indicates right > left asymmetry. Error bars show pooled SDs. Figure was generated in R using package *ggplot2* (69).

**Fig. 2.**
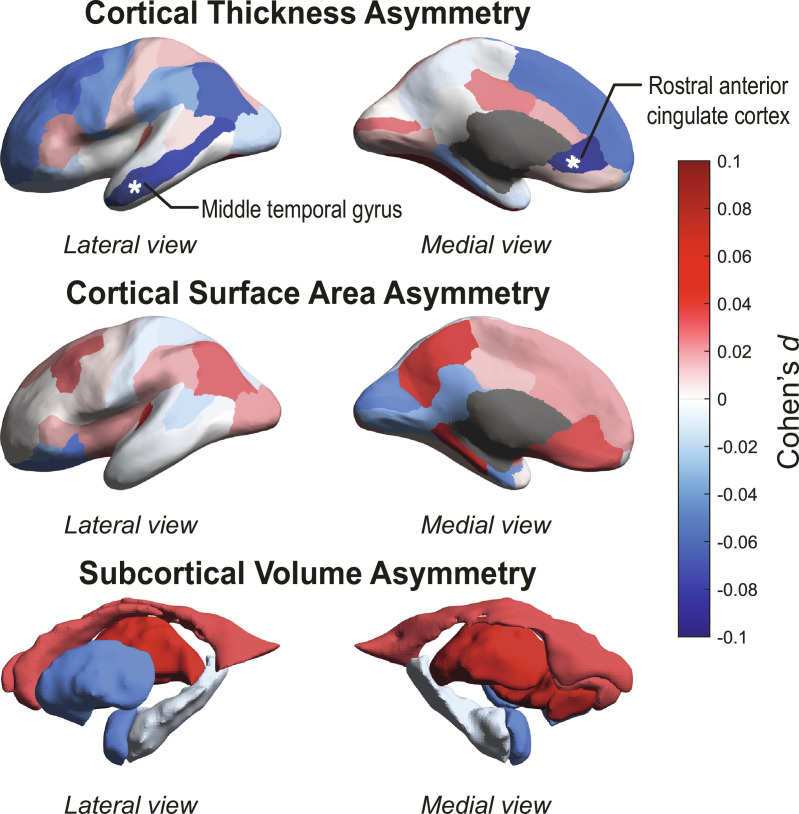
Map of cortical and subcortical asymmetry differences between individuals with schizophrenia and unaffected controls. Cohen’s *d* effect sizes from random-effects meta-analysis are projected on inflated left hemisphere cortical surface models (for cortical thickness and surface area) or subcortical structures (for subcortical volumes). Positive effects are shown in red shades (larger leftward or smaller rightward asymmetry in cases versus controls), while negative effects are shown in blue shades (smaller leftward or larger rightward asymmetry in cases versus controls). Gray shades indicate masked out structures. See also Fig. 1 and *SI Appendix*, Table S4 for directions of effects. Regions significant at *p*_FDR_ < 0.05 are labeled and marked with asterisks.

In our primary analysis (model 1), total hemispheric average cortical thickness asymmetry (*d* = −0.053, *z* = −1.92, *P* = 0.055) and surface area asymmetry (*d* = 0.027, *z* = 1.23, *P* = 0.22) did not significantly differ between affected individuals and controls. At a regional level (Fig. 1 and *SI Appendix*, Figs. S2–S4 and Table S4), there was a small but significant case–control difference in cortical thickness asymmetry of the rostral anterior cingulate cortex (*d* = −0.083, *z* = −3.21, *P* = 1.3 × 10^−3^, *p*_FDR_ = 0.047, reversal from leftward average asymmetry in controls to rightward average asymmetry in cases), and also in cortical thickness asymmetry of the middle temporal gyrus (*d* = −0.074, *z* = −2.99, *P* = 2.8 × 10^−3^, *p*_FDR_ = 0.048, increased average rightward asymmetry in cases) (Fig. 2 and *SI Appendix*, Figs. S8 and S9 and Table S5). Post hoc analysis of unilateral effects showed that both of these regional asymmetry differences were driven primarily by thinner left than right cortex in individuals with schizophrenia compared to controls (Table 1 and *SI Appendix*, Table S6). The middle temporal cortex is a core language network region (56), and left-hemisphere thinning is compatible with disrupted leftward laterality of brain organization for language in schizophrenia (10, 11). Nominally significant regional case–control associations (i.e., which did not survive multiple testing correction) were found for the AIs of inferior parietal cortex thickness, cuneus surface area, parahippocampal gyrus surface area, and nucleus accumbens volume (Fig. 2 and *SI Appendix*, Table S5).

**Table 1.** Significant brain regional thickness asymmetry differences between individuals with schizophrenia and unaffected controls

	Sample size(N)		Mean AI(SD)		Cohen’s *d* effect size[95% CI]			Average asymmetry	
Region	CTR	SZ	CTR	SZ	Left	Right	AI	CTR	SZ
**Rostral anterior cingulate cortex**	5,811	4,851	0.012 (0.086)	–0.0035 (0.092)	–0.20 [–0.28, –0.11]	–0.094 [–0.15, –0.036]	–0.083 [–0.13, –0.032]	Leftward	Reversed to rightward
**Middle temporal gyrus**	5,673	4,684	–0.0080 (0.048)	–0.015 (0.048)	–0.41 [–0.50, –0.32]	–0.36 [–0.44, –0.27]	–0.074 [–0.12, –0.026]	Rightward	Increased rightward

Mean AI = weighted mean asymmetry index across datasets. SD = pooled SD across datasets (positive mean indicates average leftward asymmetry; negative mean indicates average rightward asymmetry). Cohen’s *d* effect sizes are shown from separate meta-analysis of left-hemisphere, right-hemisphere, and asymmetry index differences between cases (SZ) and controls (CTR). No regional measures of cortical surface area asymmetry or subcortical volume asymmetry showed significant case–control differences after false discovery rate correction.

### Sensitivity Analyses.

For rostral anterior cingulate thickness asymmetry, there were three datasets in the primary meta-analysis which had outlier case–control effect sizes when compared to the meta-analyzed effect. After excluding these datasets and repeating the meta-analysis for this AI, the case–control difference remained, with the same directionality (*d* = −0.073, *z* = −3.51, *P* = 4.5 × 10^−4^) (*SI Appendix*, Table S7). For middle temporal gyrus thickness asymmetry, the exclusion of two outlier datasets also yielded a similar result compared to the primary analysis (*d* = −0.079, *z* = −3.44, *P* = 5.9 × 10^−4^), again with the same directionality (*SI Appendix*, Table S7).

Meta-regression analysis did not identify any significant moderators (no Cochran’s Q omnibus test *P* values < 0.05) (*SI Appendix*, Figs. S10–S23), i.e., Cohen’s *d* effect sizes reflecting asymmetry differences between individuals with schizophrenia and unaffected controls were not significantly influenced by scanner strength, scanner manufacturer, use of a single scanner versus multiple scanners, image slice orientation, FreeSurfer version, diagnostic tool, or the geographic origin of datasets.

In models that included either handedness, ICV, both handedness and ICV, or age^2^ as additional covariates (models 2 to 5), the case–control differences for both of these regional AIs remained nominally significant, with similar directions and magnitudes of effect compared to the case–control differences found in the primary analysis (*SI Appendix*, Table S8), despite differences in sample sizes resulting from limited availability of some of these variables.

### Medication Group Differences.

Rostral anterior cingulate thickness asymmetry did not differ between affected individuals across medication groups (model 6) (*SI Appendix*, Table S9). For the middle temporal gyrus, there was a nominally significant increase in average rightward asymmetry in affected individuals taking first-generation versus second-generation antipsychotics at the time of scanning (*d* = −0.21, *z* = −2.56, *P* = 0.011, *p*_FDR_ = 0.13), i.e., this was not significant after multiple testing correction (*SI Appendix*, Table S9).

### Correlations of Asymmetries with Clinical Variables.

We found nominally significant correlations between rostral anterior cingulate thickness asymmetry and negative symptom severity measured with the Scale for the Assessment of Negative Symptoms (SANS) (*r* = 0.049, *z* = 2.08, *P* = 0.038, *p*_FDR_ = 0.32, decreased rightward asymmetry with higher negative symptom rate) (*SI Appendix*, Table S10*A*) and between middle temporal gyrus thickness asymmetry and duration of illness (*r* = −0.048, *z* = −1.97, *P* = 0.049, *p*_FDR_ = 0.32, increased rightward asymmetry with longer duration of illness) (*SI Appendix*, Table S10*B*), but these correlations did not remain significant when correcting for multiple testing. No correlations with chlorpromazine-equivalent medication dose, age at onset, Positive and Negative Syndrome Scale (PANSS) scores (total or positive and negative subscales), or Scale for the Assessment of Positive Symptoms (SAPS) scores were found for either the rostral anterior cingulate thickness asymmetry or middle temporal gyrus thickness asymmetry (*SI Appendix*, Table S10).

### Age- and Sex-Specific Effects.

In secondary analyses across all AIs using models with interaction terms, we found a significant diagnosis-by-age interaction (model 8) for pallidum volume asymmetry (*d* = 0.081, *z* = 3.26, *P* = 1.1 × 10^−3^, *p*_FDR_ = 9.0 × 10^−3^, stronger leftward asymmetry with higher age in cases) (*SI Appendix*, Fig. S24 and Tables S11 and S12*A*). This association was driven by a significantly decreased average leftward asymmetry with increasing age in controls (*r* = −0.077, *P* = 1.1 × 10^−3^) that was not present in affected individuals (*SI Appendix*, Fig. S25 and Table S12*B*). In terms of the corresponding unilateral effects, left and right pallidum volume decreased with increasing age in individuals with schizophrenia (L: *r* = −0.17, *P* = 4.7 × 10^−9^; R: *r* =−0.20, *P* =4.7 × 10^−21^) and unaffected controls (L: *r* = −0.27, *P* = 2.1 × 10^−22^; R: *r* = −0.24, *p* = 6.2 × 10^−17^), but the two groups differed with respect to the side showing the stronger effect (*SI Appendix*, Table S12*B*). No significant diagnosis-by-sex interactions were found (model 9) (*SI Appendix*, Table S13).

### Multivariate Analysis of Case–Control Asymmetry Differences.

Considering all 76 regional structural brain AIs simultaneously in a multivariate model, applied to the 14 datasets for which individual-level data were available to the central analysis team (935 affected individuals and 1,094 controls), there was a significant multivariate structural brain asymmetry difference between cases and controls that accounted for roughly 7% of the variance considered across all 76 AIs (Wilks’ Λ = 0.932, approximate *F*(76, 1950) = 1.87, *P* = 1.25 × 10^−5^). Only three of the *F*-statistics resulting from one million label-swapping permutations were larger than the *F*-statistic from the true analysis, resulting in a permutation *P* = 3.0 × 10^−6^. We also derived univariate (ANCOVA) association statistics from the multivariate model to understand which AIs contributed most to the significant multivariate association. The structural AIs that showed nominally significant, univariate case–control differences in the 14 datasets available for this analysis were those for pallidum volume, nucleus accumbens volume, and eight regional surface area or thickness measures distributed widely over the cerebral cortex (Table 2). These did not include the two cortical regional AIs that showed significant case–control differences in the meta-analysis over all the 45 case–control datasets, but did include AIs of other language-related regions of the temporal lobe: superior temporal sulcus surface area asymmetry and transverse temporal gyrus thickness asymmetry (Table 2). The large differences in overall sample size and contributing datasets between the multivariate analysis and main meta-analysis are a likely cause of these somewhat different results.

**Table 2.** Multivariate analysis of case–control brain asymmetry differences between 935 individuals with schizophrenia and 1,094 controls for which individual-level data were available (14 datasets)

Structural asymmetry	Approximate *F*	*P*
Multivariate test (all regional cortical and subcortical asymmetries)	1.87	Nominal *P* = 1.25 × 10^−5^ Permutation *P* = 3.0 × 10^−6^
Most significant univariate effects	*F*	*P*
Pallidum (volume asymmetry)	29.1	7.8 × 10^−8^
Nucleus accumbens (volume asymmetry)	9.3	2.3 × 10^−3^
Rostral middle frontal gyrus (surface area asymmetry)	7.7	5.5 × 10^−3^
Parahippocampal gyrus (surface area asymmetry)	7.2	7.4 × 10^−3^
Parahippocampal gyrus (thickness asymmetry)	5.5	0.019
Transverse temporal gyrus (thickness asymmetry)	5.4	0.021
Cuneus (surface area asymmetry)	5.4	0.021
Banks of superior temporal sulcus (surface area asymmetry)	4.9	0.027
Insula (surface area asymmetry)	4.6	0.031
Medial orbitofrontal cortex (thickness asymmetry)	3.9	0.048

Results are shown for the multivariate MANCOVA over all asymmetries, and the specific asymmetries with nominal significance (*P* < 0.05) in the corresponding univariate ANCOVAs, with their F statistics (*F*) and *P* values (*P*).

## Discussion

In this study, we investigated group differences in structural brain asymmetries between individuals with schizophrenia and unaffected controls, in the largest sample to date. The large sample size offered unprecedented statistical power to identify group differences based on the clinical diagnosis of schizophrenia, and to measure their effect sizes (37–39). Subtle differences of regional asymmetry were found for rostral anterior cingulate thickness, middle temporal gyrus thickness, and pallidum volume (the latter in older individuals). The Cohen’s *d* effect sizes were less than 0.1; i.e., very small (70). In light of previous large-scale analyses of bilateral cortical and subcortical alterations in schizophrenia (47, 48), our results suggest that morphometric alterations in this disorder are largely the same for the left and right hemispheres, involving only subtle asymmetrical effects at the group average level. This suggests that effect sizes of brain asymmetry differences in schizophrenia reported in earlier, much smaller studies (see Introduction) are likely to have been overestimated. Nonetheless, in a multivariate context, 7% of the total variance across all regional asymmetries was explained by case–control status, indicating a diffuse and subtle alteration of brain asymmetry in schizophrenia.

Subtle group differences of asymmetry in terms of macroanatomic features, such as those studied here, may reflect effects at other neurobiological levels that have functional relevance for disorder symptoms—for example molecular, cytoarchitectonic, and/or circuit levels (71–73). For example, cortical thickness measures can correlate with the degree of myelination (74), such that quantitative neuroimaging methods that are more sensitive to microstructural tissue content may reveal alterations in the regions implicated by this study. Neurite orientation dispersion and density imaging can be used to study cortical microstructural asymmetries (73), or the ratio of T1w and T2w images in gray matter can indicate cortical myelin content (75). We suggest that future studies using such techniques can be focused on the regions identified in this study. In addition, postmortem studies of hemispheric differences in gene expression in schizophrenia are motivated.

The middle temporal gyrus is prominently involved in the brain’s language network (56), so that our finding of lower left-sided cortical thickness in schizophrenia in this region is broadly consistent with a prominent theory in the literature: That left-hemisphere language dominance may be reduced in this disorder (10, 11). Cortical thinning of the left-hemispheric middle temporal gyrus has been associated with auditory verbal hallucinations in schizophrenia (76), and is reported in individuals with first-episode schizophrenia and high familial risk for the disorder (77, 78). In terms of gray matter volume, an opposite pattern (reduced right, increased left) has been reported for the middle temporal gyrus in putatively at-risk children compared to typically developing children (79). However, volume measures confound cortical thickness and surface area, and since these two aspects of cortical anatomy are known to vary substantially independently (28, 80, 81), it is unclear how these earlier volume-based findings may relate to the present findings based on cortical thickness asymmetry. Again, earlier findings in smaller samples may have been false positives or had over-estimated effect sizes.

The rostral anterior cingulate cortex is an important hub in emotional and cognitive control (82), both of which are often affected in schizophrenia. In this region, we observed a thinner left-sided cortex in affected individuals than controls on average, which was more pronounced than on the right side. This may be consistent with a previous study where adolescent/young adult relatives of individuals with schizophrenia showed a longitudinal decline of gray matter volume in the left rostral anterior cingulate cortex compared to controls (83). It is therefore possible that asymmetrical differences in this region emerge before schizophrenia onset, although the previous study included only 23 relatives, so its reported effects remain equivocal, and it used volume rather than thickness measures. In the present study, we saw no evidence for an age*diagnosis interaction effect for this regional thickness asymmetry, which is consistent with a preonset alteration that subsequently remains stable through adulthood.

Multivariate analysis in 14 of the datasets, for which individual-level data were available, resulted in a highly significant case–control difference. Various regional asymmetries contributed to this multivariate association, with pallidum volume asymmetry showing the largest individual contribution. Pallidum volume asymmetry was especially associated with schizophrenia in older individuals, as observed in secondary testing of univariate interaction models across all the 45 case–control datasets. Larger pallidum volume in schizophrenia compared to controls—with a stronger effect in the left hemisphere—has been reported before (43, 44, 48, 84), although some datasets in our analysis partly overlapped with three of these studies (43, 44, 48). An age-dependent relationship between familial risk for schizophrenia and larger left pallidum volume has also been described in a small study of young adults (85)—this suggested that alterations of pallidum asymmetry might already be present in a prodromal stage of the disease. However, in the present study, the group difference in pallidum volume was absent in younger individuals and became more apparent in older adults. This also explains why the association was not significant in the primary univariate meta-analysis of all datasets together, i.e., it was driven by a subset of datasets that included older individuals, and that were also available for multivariate analysis (*SI Appendix*, Fig. S25). The pallidum is prominently involved in reward and motivation (86), and impaired reward anticipation and a loss of motivation are well-known negative symptoms of schizophrenia (87). However, how pallidum structural asymmetry may relate to functional disorder-relevant changes remains unknown.

Various brain regional asymmetries have shown significant heritability in a recent genome-wide analysis of general population data (28), including rostral anterior cingulate thickness asymmetry and pallidum volume asymmetry (but not middle temporal gyrus thickness asymmetry). When polygenic risk for schizophrenia was assessed with respect to these heritable asymmetries in a multivariate analysis (29), one of the strongest associations was with rostral anterior cingulate thickness asymmetry. The direction of that effect was consistent with the present study, i.e., a rightward shift of asymmetry with increased polygenic risk for schizophrenia. In contrast, pallidum volume asymmetry showed little relation to schizophrenia polygenic risk (29), suggesting nonheritable contributions to this association. These genetic findings were established with adult general population data (UK Biobank) (29), but together with the current case–control findings, they indicate that altered rostral anterior cingulate thickness asymmetry may be a link between genetic susceptibility and disorder presentation. Left–right asymmetry of the brain originates during development in utero (71, 88–93), and specific genomic loci that affect brain asymmetry have recently been identified (28, 94). Some of the implicated genes may be involved in patterning the left–right axis of the embryonic or fetal brain, and genes expressed at different levels on the left and right sides of the embryonic central nervous system were found to be particularly likely to affect schizophrenia susceptibility (88). However, other genes may affect brain asymmetry as it changes throughout the lifespan (2, 95) and therefore may affect susceptibility to asymmetry-associated disorders later in life.

The magnitudes of effects in this study were in line with those reported in recent large-scale studies of brain asymmetry in other psychiatric disorders carried out through the ENIGMA consortium (50–53). In ASD, a similar decreased leftward asymmetry of rostral anterior cingulate thickness was reported (51)—this region is important in cognitive control which can be impaired in both schizophrenia and ASD. For ADHD, a nominally significant increase in rightward asymmetry of middle temporal gyrus thickness was reported, while in adults specifically, less leftward asymmetry of pallidum volume was found (53). The former finding is consistent in its direction of effect with the present study, while the latter is opposite. For OCD, the pallidum was found to be less left lateralized in cases versus controls in a pediatric dataset and this effect was again opposite to our current findings in older individuals with schizophrenia (52). These cross-disorder comparisons suggest that clinical and etiological similarities and differences between schizophrenia and other psychiatric disorders might be partly reflected in asymmetry alterations involving some of the same brain regions. For further discussion of brain asymmetry alterations across multiple psychiatric traits, see Mundorf et al. (96).

Schizophrenia is a highly heterogeneous disorder covering a range of possible symptoms, which may correspond to differing underlying disease mechanisms. Our primary analysis only considered case–control group average differences based on the overall diagnosis of schizophrenia, and in secondary analyses, we did not find significant correlations of asymmetries with major clinical variables within cases after adjusting for multiple testing—including age at onset, duration of illness, and symptom scores. However, data for several variables were only available from a limited number of study sites (medication, handedness, clinical variables), reducing the sample size and thus statistical power in these secondary analyses. More detailed clinical data would be useful to gather in future large-scale studies of structural asymmetries. For example, a future study could investigate middle temporal gyrus thickness asymmetry in relation to the presence and severity of auditory verbal hallucinations (note that PANSS question 3 does not distinguish between auditory, visual, olfactory, or somatic types of hallucination, so a more targeted clinical assessment would be required).

This was the largest study of structural brain asymmetries in schizophrenia to date, and made use of a single image processing and analysis pipeline to support analysis across multiple datasets. The fact that we used data from a range of imaging equipment, diagnostic tools, and regions of the world ensures generalizability of our findings, as they pertain to the diverse manner in which schizophrenia is diagnosed and studied internationally. Therefore, a major strength of our approach is in showing consensus effects across intersite variations in techniques and samples. Unlike in a highly selected, single-site or single-equipment study, the broad and generalizable total dataset made it unlikely that any single factor confounded our findings. We used a meta-analytic approach after testing for effects separately within each dataset, where cases and controls were matched for technical and demographic factors within each dataset. This allowed us to assume and control for variations between datasets in our main analysis. In addition, meta-regression analyses indicated that between-dataset variability in technical, diagnostic, or geographic aspects had no significant impact on the associations between schizophrenia and regional brain asymmetries identified in this study. It is also worth noting that several findings from the ENIGMA-Schizophrenia working group (not related to asymmetry) have been replicated by The Cognitive Genetics Collaborative Research Organization in a sample collected in Japan (97), supporting generalization of findings across populations.

We used cross-sectional datasets, limiting the possible interpretation with respect to cause–effect relations, longitudinal changes in asymmetry, or medication effects on asymmetry. Many of the individuals with schizophrenia were likely to be past or current users of medication, although data on medication were only available for a subset of datasets and were also limited to medication use at the time of scanning. We found no evidence that the asymmetries of rostral anterior cingulate thickness or middle temporal gyrus thickness were different in affected individuals using medication versus those not using medication, which may indicate that the case–control differences of asymmetry that we detected had a developmental origin, rather than reflecting medication use. Indeed, medication effects on cortical thickness may be predominantly bilateral, without necessarily affecting asymmetry. We are not aware of any comparably sized prospective/randomized study in which medication effects could be disentangled from case–control effects.

We found a tentative difference of middle temporal gyrus thickness asymmetry between individuals who were taking first-generation versus second-generation antipsychotics. In principle, this finding might reflect a change of asymmetry in response to first-generation medication in particular, or else clinical differences of disorder presentation linked to asymmetry which then affect treatment choices. We saw nominally significant evidence that this same regional asymmetry relates to illness duration. However, the medication subgroup analyses were limited by relatively small sample sizes compared to the primary case–control analysis, and this particular association did not survive multiple testing correction. Also, medication status did not include information on previously used antipsychotics. This association therefore remains uncertain until replicated.

We used macroanatomical brain atlases for both the cortical and subcortical structures, which is the most feasible approach for large-scale analysis across multiple datasets, but limits spatial resolution. With higher resolution mapping, regions that showed negative results in our study may harbor more focal case–control asymmetry differences, which could be revealed for example through vertex-wise cortical mapping (63, 94, 98), or subcortical partitioning into subfields or nuclei.

This study focused on group average differences, but individual-level deviations in affected individuals may be highly heterogeneous and not well captured by group-average approaches (99). Future studies may investigate individual or patient subgroup asymmetry deviations from a normative range or structural pattern, which may deliver clinical utility, for example through contributing to diagnosis or prognosis. This concept has shown promising results in recent studies even in smaller samples (99, 100). The small group-average effects that we identified in the present study are unlikely to have clinical utility when considered in isolation, although they may contribute to multivariate prediction models in future research, for example when considering brain features across multiple imaging modalities.

In summary, we performed the largest study of asymmetry differences between individuals with schizophrenia and unaffected controls to date. Effect sizes were small, but several regional case–control asymmetry differences in cortical thickness and subcortical volume were suggested, and multivariate analysis indicated that 7% of variation across all regional asymmetries could be explained by the case–control group difference. Our findings therefore support a long-standing theory that the brain’s asymmetry can be different in schizophrenia (10, 11), even if earlier studies in smaller samples were likely to have overestimated the effect sizes in relation to structural asymmetry. Altered asymmetry in schizophrenia may conceivably occur during development through disruption of a genetically regulated program of asymmetrical brain development, and/or through different trajectories of lifespan-related changes in brain asymmetries. The specific regions implicated here provide targets for future research on the molecular and cellular basis of altered lateralized cognitive functions in schizophrenia, which may ultimately help to identify pathophysiological mechanisms.

## Supplementary Material

Appendix 01 (PDF)

## Data Availability

This study made use of 46 separate data sets collected around the world, under a variety of different consent procedures and regulatory bodies, during recent decades. Requests to access the data sets will be considered in relation to the relevant consents, rules and regulations, and can be made via the schizophrenia working group of the ENIGMA consortium: http://enigma.ini.usc.edu/ongoing/enigma-schizophrenia-working-group/.
