# Supplementary material for: Large-scale analysis of structural brain asymmetries in schizophrenia via the ENIGMA consortium
Source: Proc Natl Acad Sci U S A. 2023 Mar 28;120(14):e2213880120. doi: 10.1073/pnas.2213880120 (PMC10083554; doi:10.1073/pnas.2213880120)
Supplement: Supplementary file 1 — Appendix 01 (PDF) [file pnas.2213880120.sapp.pdf]

PNAS

Supporting Information for

Large-scale analysis of structural brain asymmetries in schizophrenia via the ENIGMA consortium

Dick Schijven, Merel C. Postema, Masaki Fukunaga, Junya Matsumoto, Kenichiro Miura, Sonja M.C. de Zwarte, Neeltje E.M. van Haren, Wiepke Cahn, Hilleke E. Hulshoff Pol, René S. Kahn, Rosa Ayesa-Arriola, Víctor Ortiz-García de la Foz, Diana Tordesillas-Gutierrez, Javier Vázquez-Bourgon, Benedicto Crespo-Facorro, Dag Alnæs, Andreas Dahl, Lars T. Westlye, Ingrid Agartz, Ole A. Andreassen, Erik G. Jönsson, Peter Kochunov, Jason M. Bruggemann, Stanley V. Catts, Patricia T. Michie, Bryan J. Mowry, Yann Quidé, Paul E. Rasser, Ulrich Schall, Rodney J. Scott, Vaughan J. Carr, Melissa J. Green, Frans A. Henskens, Carmel M. Loughland, Christos Pantelis, Cynthia Shannon Weickert, Thomas W. Weickert, Lieuwe de Haan, Katharina Brosch, Julia-Katharina Pfarr, Kai G. Ringwald, Frederike Stein, Andreas Jansen, Tilo T.J. Kircher, Igor Nenadić, Bernd Krämer, Oliver Gruber, Theodore D. Satterthwaite, Juan Bustillo, Daniel H. Mathalon, Adrian Preda, Vince D. Calhoun, Judith M. Ford, Steven G. Potkin, Jingxu Chen, Yunlong Tan, Zhiren Wang, Hong Xiang, Fengmei Fan, Fabio Bernardoni, Stefan Ehrlich, Paola Fuentes-Claramonte, Maria Angeles Garcia-Leon, Amalia Guerrero-Pedraza, Raymond Salvador, Salvador Sarró, Edith Pomarol-Clotet, Valentina Ciullo, Fabrizio Piras, Daniela Vecchio, Nerisa Banaj, Gianfranco Spalletta, Stijn Michielse, Therese van Amelsvoort, Erin W. Dickie, Aristotle N. Voineskos, Kang Sim, Simone Ciufolini, Paola Dazzan, Robin M. Murray, Woo-Sung Kim, Young-Chul Chung, Christina Andreou, André Schmidt, Stefan Borgwardt, Andrew M. McIntosh, Heather C. Whalley, Stephen M. Lawrie, Stefan du Plessis, Hilmar K. Luckhoff, Freda Scheffler, Robin Emsley, Dominik Grotegerd, Rebekka Lencer, Udo Dannlowski, Jesse T. Edmond, Kelly Rootes-Murdy, Julia M. Stephen, Andrew R. Mayer, Linda A. Antonucci, Leonardo Fazio, Giulio Pergola, Alessandro Bertolino, Covadonga M. Díaz-Caneja, Joost Janssen, Noemi G. Lois, Celso Arango, Alexander S. Tomyshev, Irina Lebedeva, Simon Cervenka, Carl M. Sellgren, Foivos Georgiadis, Matthias Kirschner, Stefan Kaiser, Tomas Hajek, Antonin Skoch, Filip Spaniel, Minah Kim, Yoo Bin Kwak, Sanghoon Oh, Jun Soo Kwon, Anthony James, Geor Bakker, Christian Knöchel, Michael Stäblein, Viola Oertel, Anne Uhlmann, Fleur M. Howells, Dan J. Stein, Henk S. Temmingh, Ana M. Diaz-Zuluaga, Julian A. Pineda-Zapata, Carlos López-Jaramillo, Stephanie Homan, Ellen Ji, Werner Surbeck, Philipp Homan, Simon E. Fisher, Barbara Franke, David C. Glahn, Ruben C. Gur, Ryota Hashimoto, Neda Jahanshad, Eileen Luders, Sarah E. Medland, Paul M. Thompson, Jessica A. Turner, Theo G.M. van Erp, Clyde Francks

Corresponding author:

Clyde Francks
E-mail: Clyde.Francks@mpi.nl

This PDF file includes:

Supporting Information 1 to 3
Figures S1 to S25
Tables S1 to S13

Table of contents

Supporting Information 1: Quality control for image orientation 3

Supporting Information 2: Overview of statistical models for regression and partial correlation analyses 4

Supporting Information 3: Sensitivity and secondary analyses 6

Supporting figures 8

Supporting tables.. 53

SI References 83

Supporting Information 1: Quality control for image orientation

The standardized pipeline from raw image data through FreeSurfer does not introduce left-right flipping errors, but to ensure that such errors were not introduced during processing of raw imaging data by non-standard processes (e.g. during the conversion of DICOM to NIFTI files), we compared mean regional asymmetry indexes (AIs) for all datasets against grand sample-size adjusted means. If we noticed a large proportion of reversed average AIs for a dataset, we contacted the relevant site to re-check and correct their process. Table S3 provides a per-region overview of mean asymmetry direction in each dataset compared to grand sample-size adjusted means across datasets.

Supporting Information 2: Overview of statistical models for regression and partial correlation analyses

Below is an overview of the models for the linear regression and partial correlation analyses that were run by each participating site, and from which summary statistics for meta-analysis by the central analysis group were extracted. Model numbers refer to those indicated in the main manuscript text and Supporting Information 3. The independent variable highlighted in **bold** is the predictor of interest in each model, for which effects were combined across datasets in random-effects meta-analysis.

Abbreviations used in models:

Variable	Type	Description
AI	Continuous	Asymmetry index
Dx	Categorical (binary)	Diagnosis: schizophrenia or unaffected control
HAND	Categorical (binary)	Hand preference: right or non-right (left + ambidextrous)
ICV	Continuous	Intracranial volume
Scanner	Categorical (binary)	Optional covariate: If a site used multiple scanners to obtain images, *n*-1 binary dummy covariates (where *n* is the number of scanners in a given dataset) were added, to differentiate which scanner an individual's data came from.
AP-group	Categorical	Antipsychotic medication groups, tested as binary variables for between-group comparisons.
Clinical variable	Continuous	Schizophrenia-specific clinical variable. We included chlorpromazine-equivalent (CPZ) medication dose, age at onset, duration of illness, PANSS total score, PANSS positive symptom score, PANSS negative symptom score, SAPS score or SANS score.

Models to assess case-control differences

Primary model:

[1] AI ~ **Dx** + Age + Sex (+ Scanner)

Primary model with additional covariates:

[2] AI ~ **Dx** + Age + Sex + HAND (+ Scanner)

[3] AI ~ **Dx** + Age + Sex + ICV (+ Scanner)

[4] AI ~ **Dx** + Age + Sex + HAND + ICV (+ Scanner)

[5] AI ~ **Dx** + Age + Age^2 + Sex (+ Scanner)

Models to assess medication group differences

Antipsychotic medication between-group comparisons within affected individuals:

[6] AI ~ **AP-group** + Age + Sex (+ Scanner)

Models to assess correlations with clinical variables in affected individuals

[7] Linear model: AI ~ **Clinical variable** + Sex + Age (+ Scanner)

Partial correlation: ρ(AI)(**Clinical variable**)·{Sex, Age, (Scanner)}

Models to assess diagnosis-by-age and diagnosis-by-sex interactions, including correlations with age

[8] AI ~ Dx + Age + Sex + **Dx*Age +** (Scanner)

[8b] Linear model: AI ~ **Age** + Sex (+ Scanner)

Partial correlation: ρ(AI)(**Age**)·{Sex, (Scanner)}

[9] AI ~ Dx + Age + Sex + **Dx*Sex** + (Scanner)

Supporting Information 3: Sensitivity and secondary analyses

Sensitivity analyses

For any asymmetry index (AI) that showed a significant case-control group difference in the primary meta-analysis, we carried out three types of sensitivity analyses:

First, we identified datasets within which the 95% CI of the diagnosis effect did not overlap with the 95% CI of the meta-analyzed effect – using the 'find.outliers' function in the R package *dmetar* (v0.0.9) (1) – and then repeated the meta-analysis after excluding such outlier datasets.

Second, to assess whether between-dataset heterogeneity in effect sizes could be partly explained by known aspects of technical, diagnostic or geographic variability between datasets, we applied meta-regression and the Cochran's Q test. As possible moderators we tested scanner strength, scanner manufacturer, use of a single scanner versus multiple scanners, image slice orientation, FreeSurfer version, diagnostic tool, and geographic origin of datasets (ethnicity was not recorded). See Table S2 for more information on these possible moderators.

Third, we applied models that included the same covariates as the primary analysis, but additionally included either handedness (right-handed vs. non-right-handed), intracranial volume (ICV), both handedness and ICV, or age^2 (models 2-5 in Supporting Information 2).

Medication group differences

For AIs that showed significant case-control group differences in the primary analysis, we explored associations with antipsychotic medication use at the time of scanning, through between-group comparisons of AIs of unmedicated individuals with schizophrenia, affected individuals taking only first-generation (typical) antipsychotics, affected individuals taking only second-generation (atypical) antipsychotics, and those taking both first- and second-generation antipsychotics. Sex and age were included as covariates (model 6 in Supporting Information 2) and derived Cohen's *d* effect sizes were again meta-analyzed across datasets in a random-effects model. Applying a minimum group size threshold of 5 within any given dataset, sufficient data on the presence/absence of antipsychotic medication use for at least one comparison were available for 31 of the datasets (Table S1B), and the sample sizes for each between-group comparison are in Table S9. We calculated FDR corrected p-values to correct for all of the multiple subgroup comparisons and structural asymmetries tested.

Correlations of asymmetries with clinical variables

For AIs that showed significant case-control group differences in the primary analysis, we assessed relationships between these AIs and clinical variables within affected individuals only: age at onset, duration of illness, chlorpromazine equivalent medication dose (at the time of scanning), as well as positive, negative, and total symptom severity scores from the Positive and Negative Syndrome Scale (PANSS) (2), or the Scale for the Assessment of Positive Symptoms (SAPS) (3) and Scale for the Assessment of Negative Symptoms (SANS) (4) (separately depending on data availability, see Table S1A). Partial correlations between brain AIs and these quantitative measures were estimated using

the ‘pcor.test’ function in the R package *ppcor* (v1.1) (5). Age and sex were included as covariates (model 7 in Supporting Information 2). The same minimum sample size requirement for dataset inclusion was applied as in the linear regression analyses (above). Correlation coefficients were meta-analyzed across datasets in a mixed-effects model including dataset as a random effect. We calculated FDR corrected p-values to control for all of the clinical variables and structural asymmetries tested. Sample sizes for each model are shown in Table S10.

Age- and sex-specific effects

For all AIs in all case-control datasets we applied models which were the same as the primary analysis but additionally included either diagnosis-by-age or diagnosis-by-sex interaction terms. We then carried out meta-analyses of the interaction effect estimates across datasets to assess possible AI differences between affected individuals and controls that were relatively specific to either males or females, or differed with age (models 8-9 in Supporting Information 2). In the same way as our primary analysis, we calculated FDR corrected p-values to account for multiple regional asymmetries tested.

Supporting figures

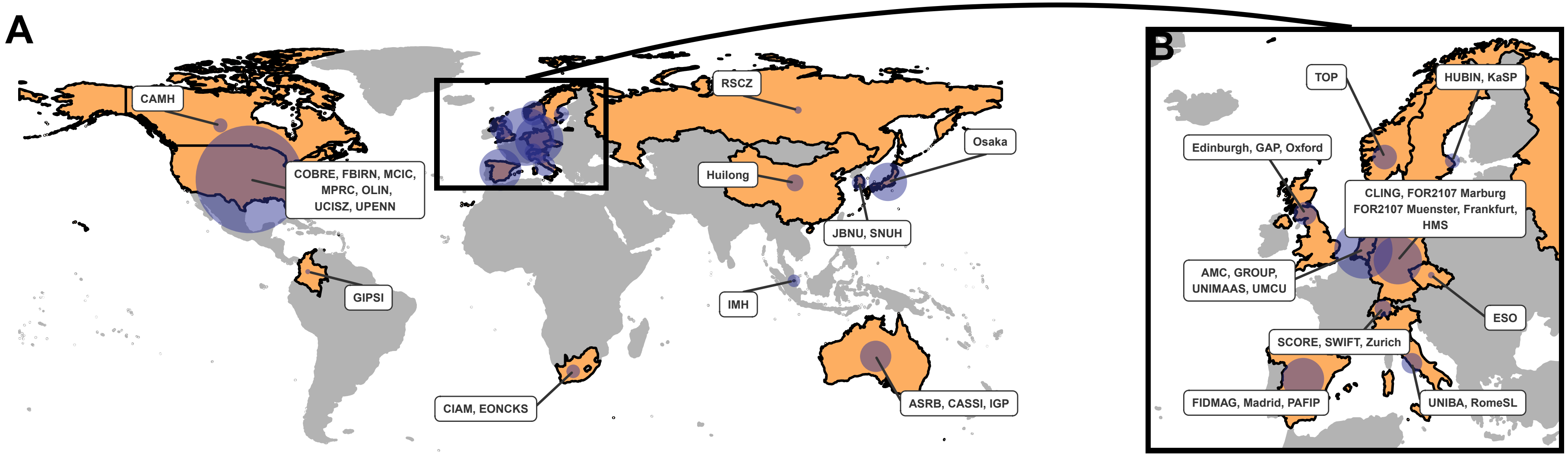

Fig. S1. Geographic origin of included datasets. A) Countries from which one or more datasets originate are highlighted in the world map, with dataset names included in labels. The relative sample size of datasets per country is indicated by blue circles. **B)** Zoomed map of Europe. For more details, see Table S1. Figure generated in R using packages *ggplot2* (6), *rnaturalearth* (7), *sf* (8) and *ggrepel* (9).

Fig. S2 (page 10-18). Overall and per-dataset average and range for cortical thickness asymmetries. For each cortical thickness asymmetry measure, the average in controls (green circles) and individuals affected with schizophrenia (purple squares) is shown. The top (highlighted) row contains the grand sample size-weighted mean and standard deviation (thick line segments). The other rows contain per-dataset averages, standard deviations and minimum and maximum values (indicated with thin line segments).

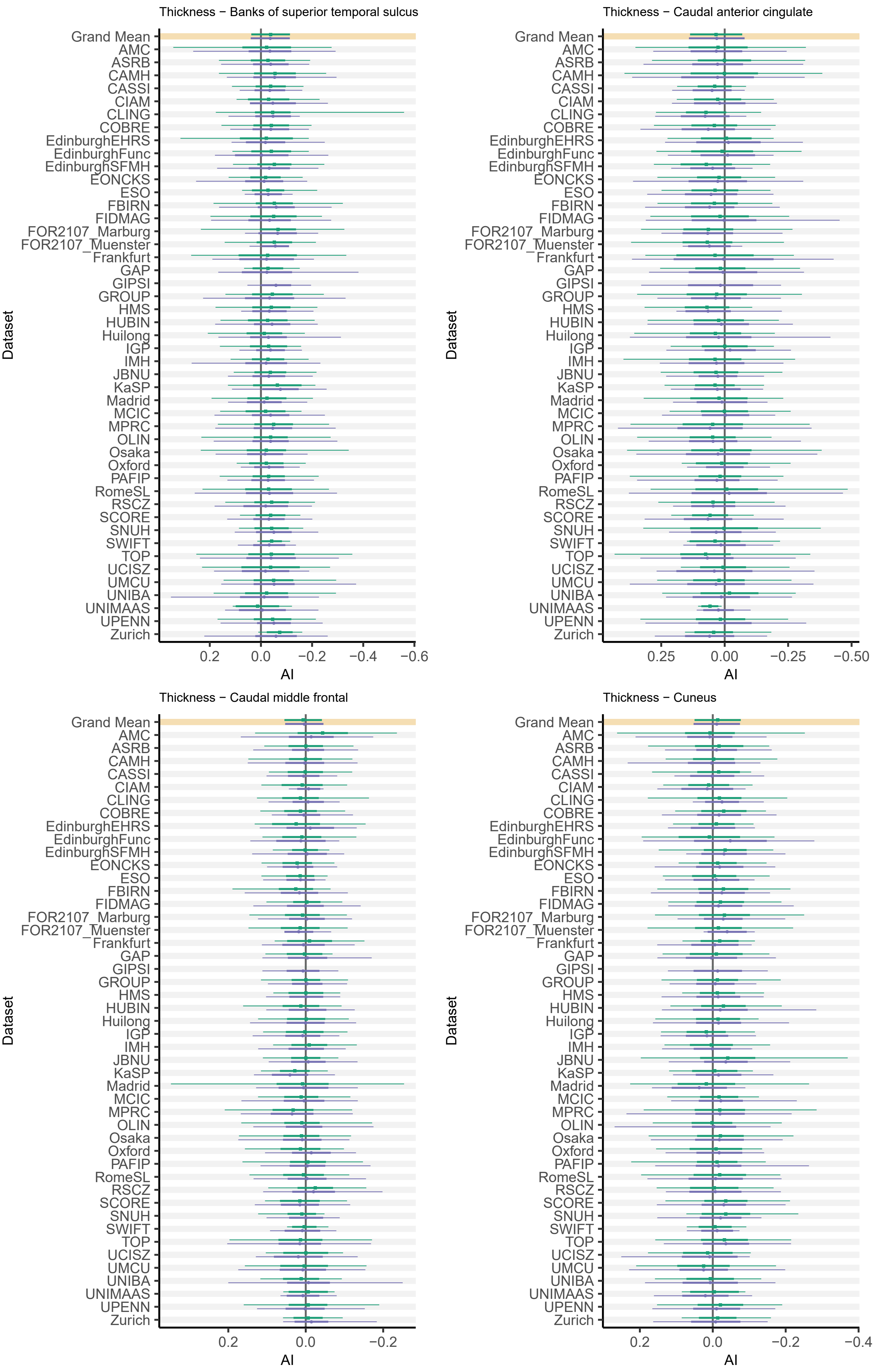
Thickness − Banks of superior temporal sulcus
Thickness − Caudal anterior cingulate
Thickness − Caudal middle frontal
Thickness − Cuneus
Dataset
Grand Mean
AMC
ASRB
CAMH
CASSI
CIAM
CLING
COBRE
EdinburghEHRS
EdinburghFunc
EdinburghSFMH
EONCKS
ESO
FBIRN
FIDMAG
FOR2107_Marburg
FOR2107_Muenster
Frankfurt
GAP
GIPSI
GROUP
HMS
HUBIN
Huilong
IGP
IMH
JBNU
KaSP
Madrid
MCIC
MPRC
OLIN
Osaka
Oxford
PAFIP
RomeSL
RSCZ
SCORE
SNUH
SWIFT
TOP
UCISZ
UMCU
UNIBA
UNIMAAS
UPENN
Zurich
0.2
0.0
−0.2
−0.4
−0.6
0.25
0.00
−0.25
−0.50
AI

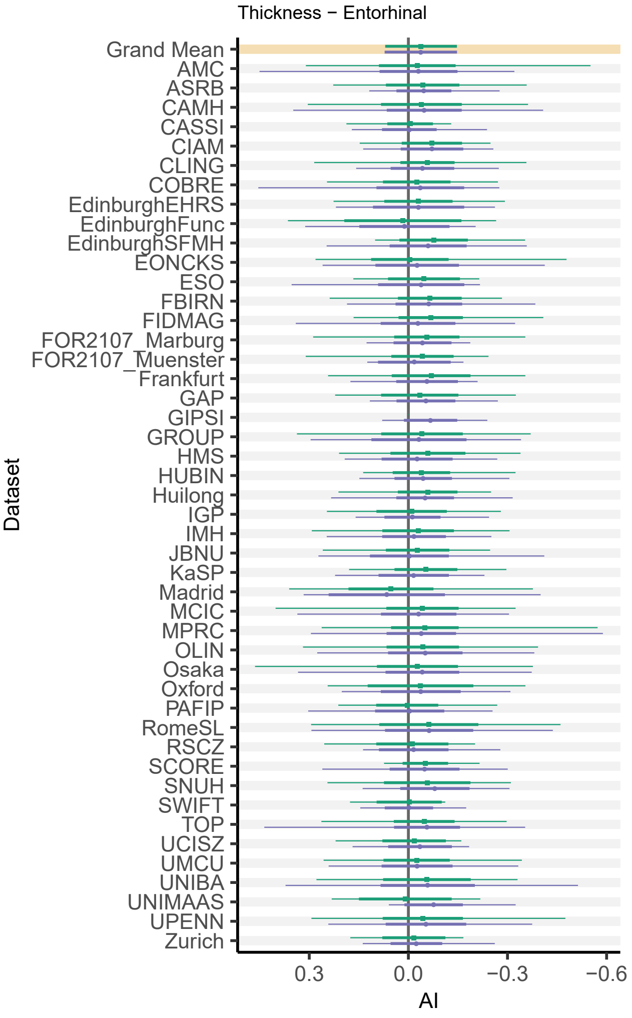
Thickness − Entorhinal
Dataset
AI
Grand Mean
AMC
ASRB
CAMH
CASSI
CIAM
CLING
COBRE
EdinburghEHRS
EdinburghFunc
EdinburghSFMH
EONCKS
ESO
FBIRN
FIDMAG
FOR2107_Marburg
FOR2107_Muenster
Frankfurt
GAP
GIPSI
GROUP
HMS
HUBIN
Huilong
IGP
IMH
JBNU
KaSP
Madrid
MCIC
MPRC
OLIN
Osaka
Oxford
PAFIP
RomeSL
RSCZ
SCORE
SNUH
SWIFT
TOP
UCISZ
UMCU
UNIBA
UNIMAAS
UPENN
Zurich
0.3
0.0
−0.3
−0.6

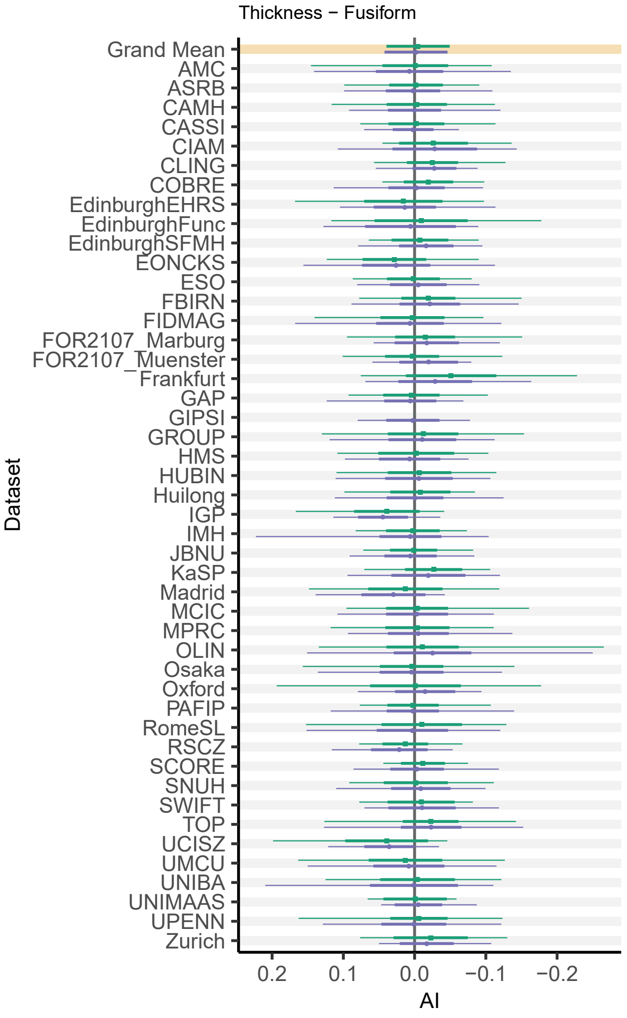
Thickness − Fusiform
Dataset
AI
0.2
0.1
0.0
−0.1
−0.2

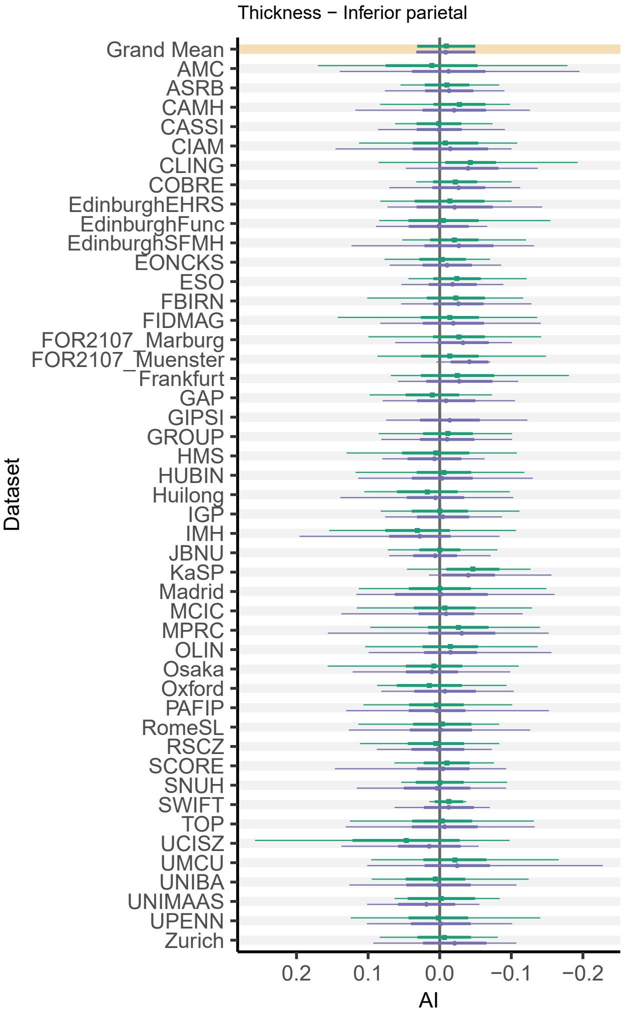
Thickness − Inferior parietal
Dataset
AI
0.2
0.1
0.0
−0.1
−0.2

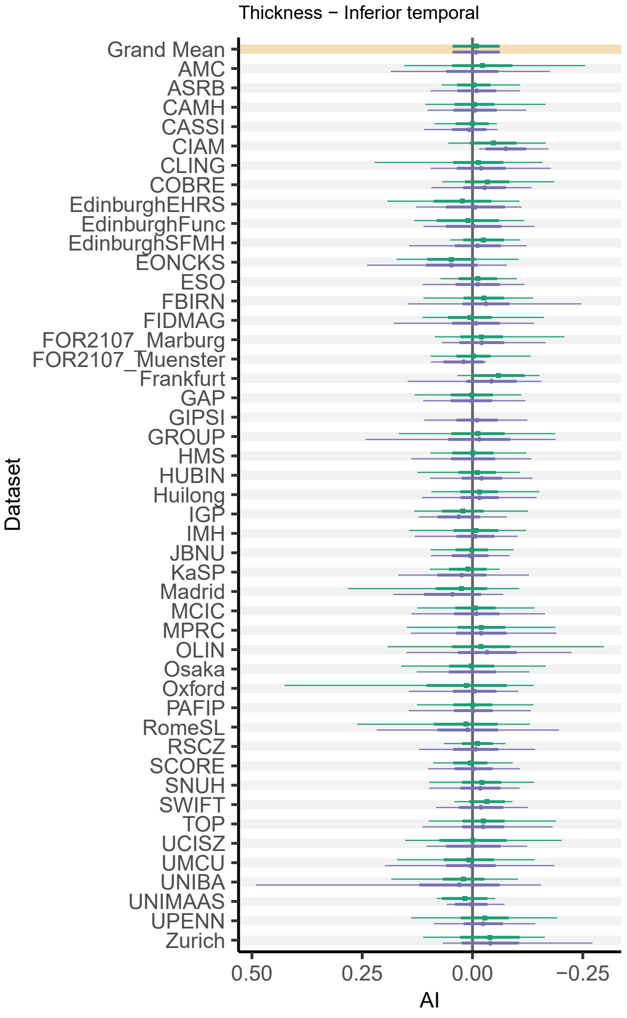
Thickness − Inferior temporal
Dataset
AI
0.50
0.25
0.00
−0.25

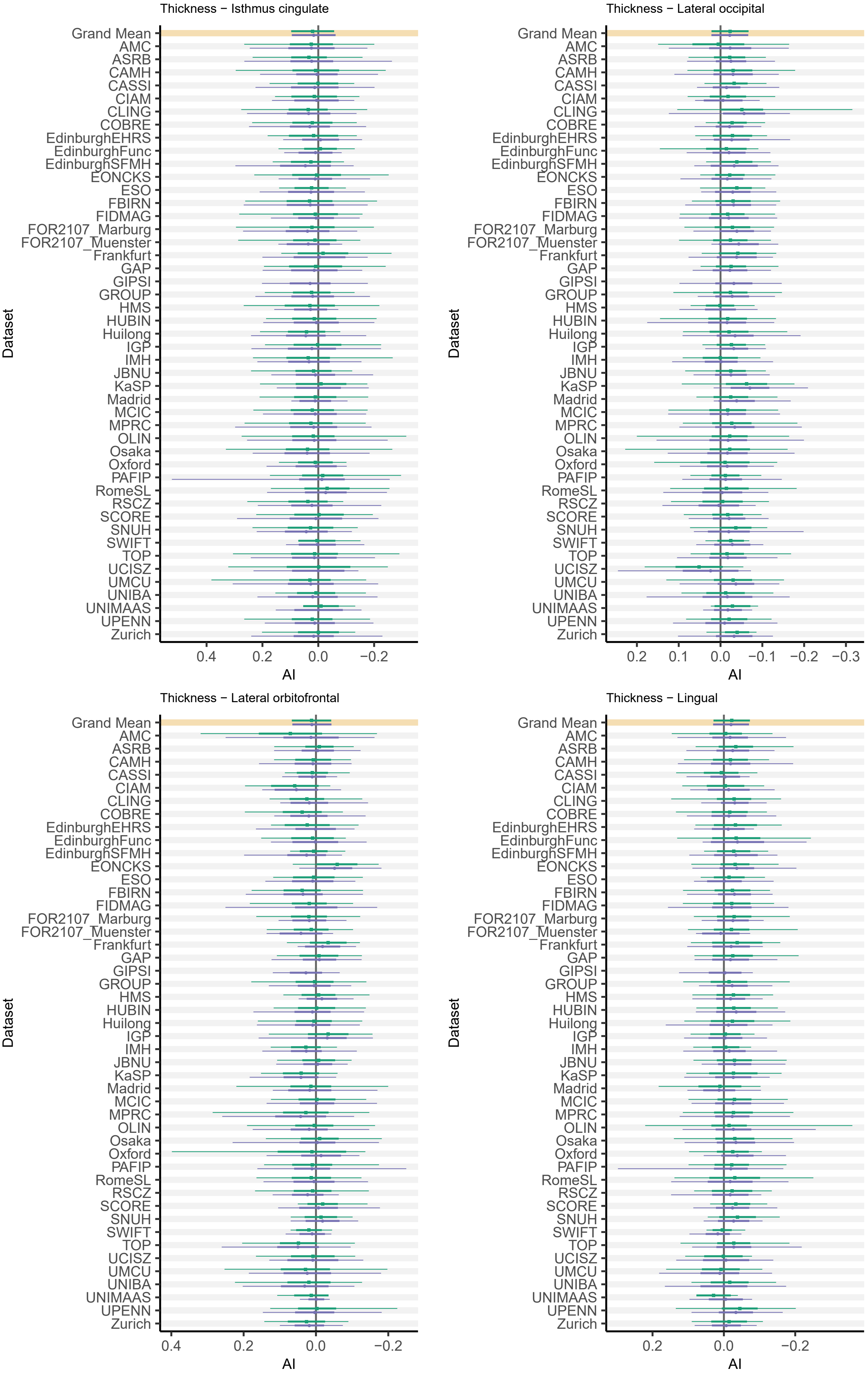
Thickness − Isthmus cingulate
Thickness − Lateral occipital
Thickness − Lateral orbitofrontal
Thickness − Lingual
Dataset
Grand Mean
AMC
ASRB
CAMH
CASSI
CIAM
CLING
COBRE
EdinburghEHRS
EdinburghFunc
EdinburghSFMH
EONCKS
ESO
FBIRN
FIDMAG
FOR2107_Marburg
FOR2107_Muenster
Frankfurt
GAP
GIPSI
GROUP
HMS
HUBIN
Huilong
IGP
IMH
JBNU
KaSP
Madrid
MCIC
MPRC
OLIN
Osaka
Oxford
PAFIP
RomeSL
RSCZ
SCORE
SNUH
SWIFT
TOP
UCISZ
UMCU
UNIBA
UNIMAAS
UPENN
Zurich
0.4 0.2 0.0 −0.2
0.2 0.1 0.0 −0.1 −0.2 −0.3
0.4 0.2 0.0 −0.2
0.2 0.0 −0.2
AI

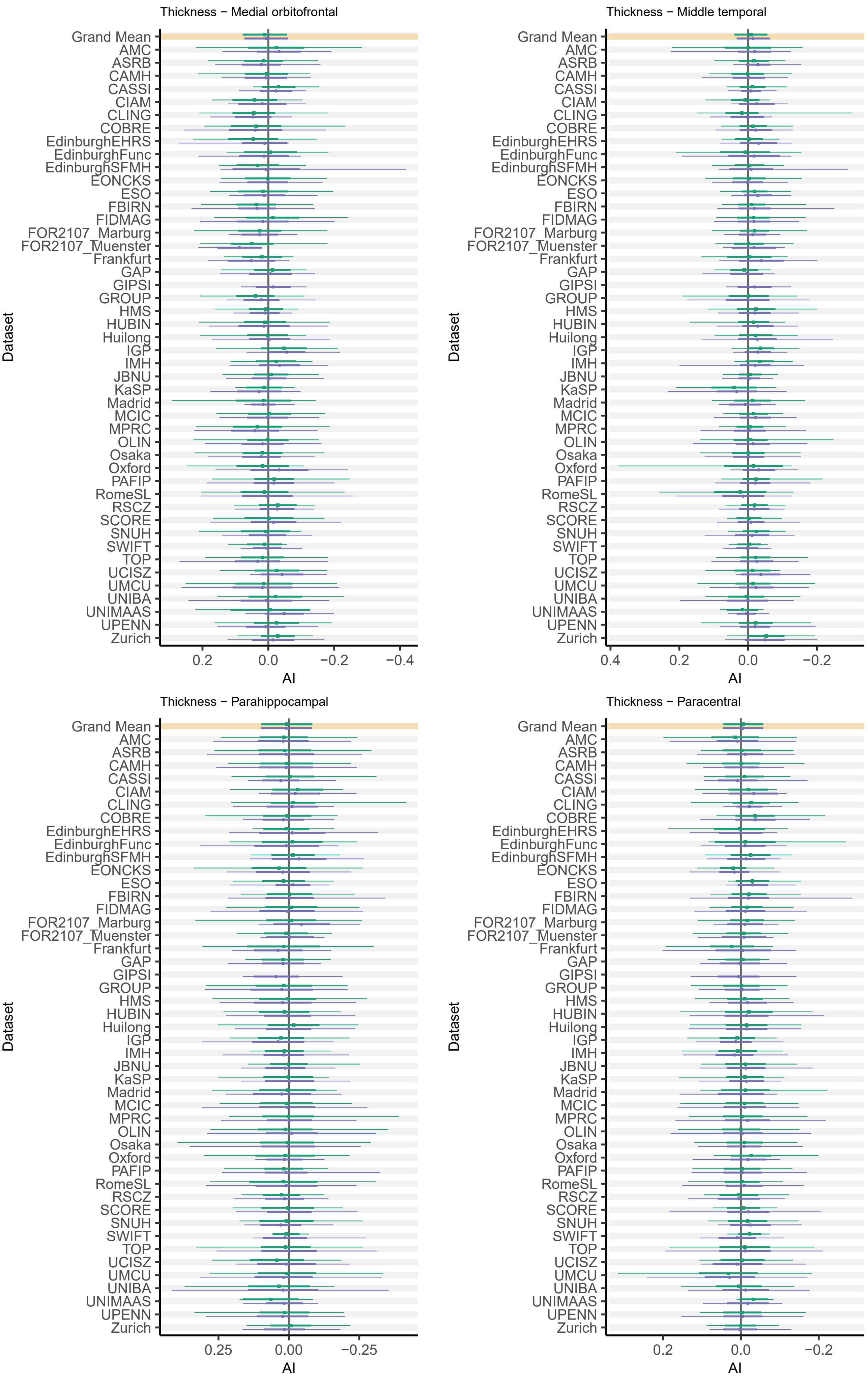

Thickness − Medial orbitofrontal
Thickness − Middle temporal
Thickness − Parahippocampal
Thickness − Paracentral
Grand Mean
AMC
ASRB
CAMH
CASSI
CIAM
CLING
COBRE
EdinburghEHRS
EdinburghFunc
EdinburghSFMH
EONCKS
ESO
FBIRN
FIDMAG
FOR2107_Marburg
FOR2107_Muenster
Frankfurt
GAP
GIPSI
GROUP
HMS
HUBIN
Huilong
IGP
IMH
JBNU
KaSP
Madrid
MCIC
MPRC
OLIN
Osaka
Oxford
PAFIP
RomeSL
RSCZ
SCORE
SNUH
SWIFT
TOP
UCISZ
UMCU
UNIBA
UNIMAAS
UPENN
Zurich
Dataset
AI
0.2
0.0
−0.2
−0.4
0.4
0.25
0.00
−0.25

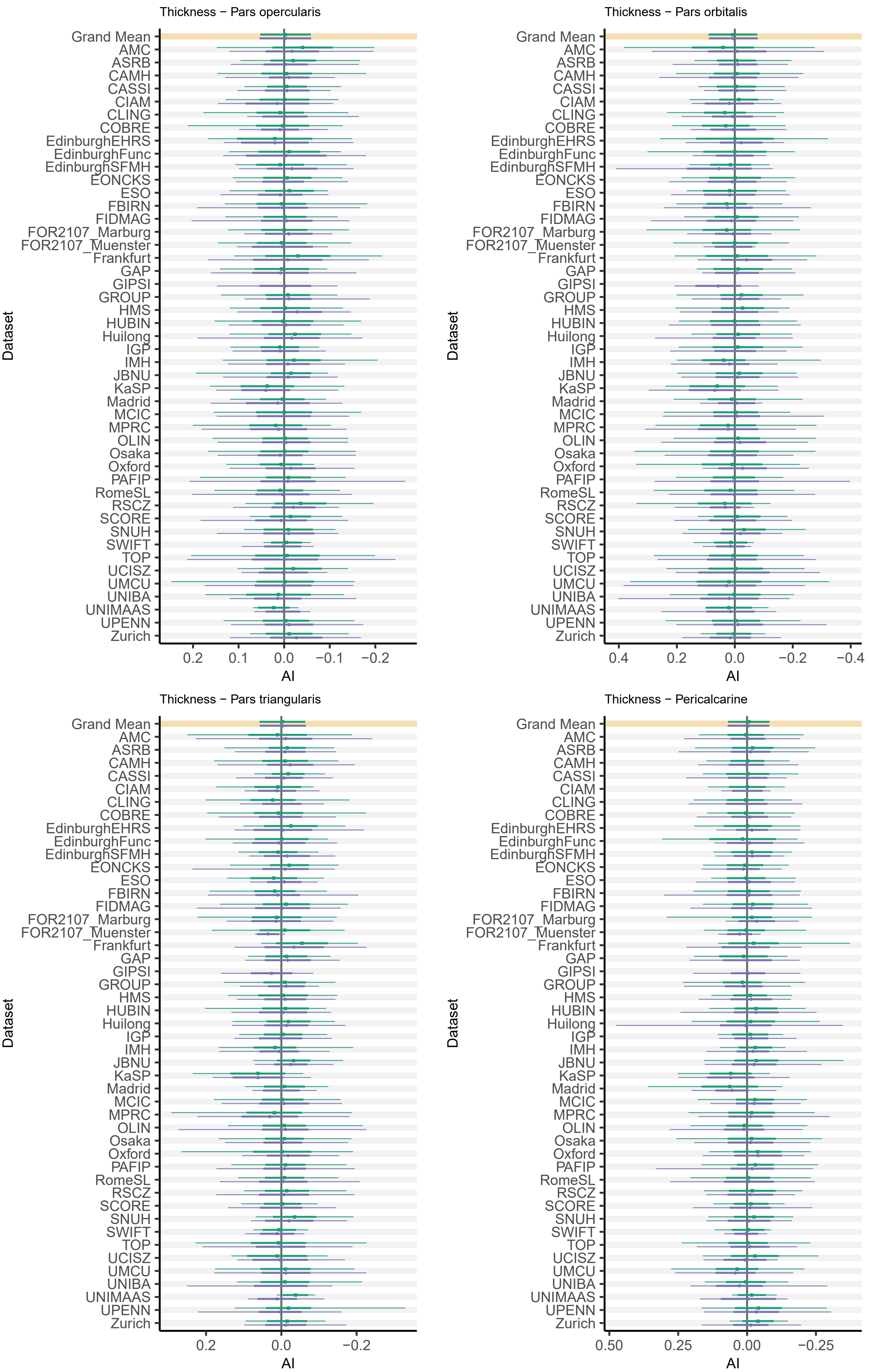
Thickness − Pars opercularis
Thickness − Pars orbitalis
Thickness − Pars triangularis
Thickness − Pericalcarine
Grand Mean
AMC
ASRB
CAMH
CASSI
CIAM
CLING
COBRE
EdinburghEHRS
EdinburghFunc
EdinburghSFMH
EONCKS
ESO
FBIRN
FIDMAG
FOR2107_Marburg
FOR2107_Muenster
Frankfurt
GAP
GIPSI
GROUP
HMS
HUBIN
Huilong
IGP
IMH
JBNU
KaSP
Madrid
MCIC
MPRC
OLIN
Osaka
Oxford
PAFIP
RomeSL
RSCZ
SCORE
SNUH
SWIFT
TOP
UCISZ
UMCU
UNIBA
UNIMAAS
UPENN
Zurich
Dataset
0.2
0.1
0.0
−0.1
−0.2
0.4
−0.4
0.50
0.25
0.00
−0.25
AI

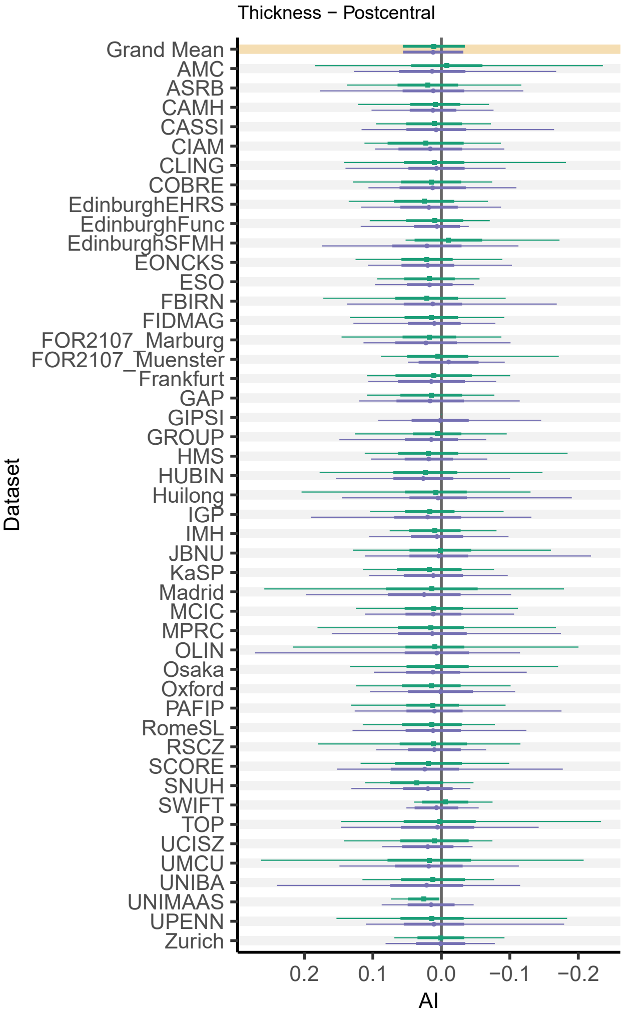
Thickness − Postcentral
Grand Mean
AMC
ASRB
CAMH
CASSI
CIAM
CLING
COBRE
EdinburghEHRS
EdinburghFunc
EdinburghSFMH
EONCKS
ESO
FBIRN
FIDMAG
FOR2107_Marburg
FOR2107_Muenster
Frankfurt
GAP
GIPSI
GROUP
HMS
HUBIN
Huilong
IGP
IMH
JBNU
KaSP
Madrid
MCIC
MPRC
OLIN
Osaka
Oxford
PAFIP
RomeSL
RSCZ
SCORE
SNUH
SWIFT
TOP
UCISZ
UMCU
UNIBA
UNIMAAS
UPENN
Zurich
Dataset
0.2
0.1
0.0
−0.1
−0.2
AI

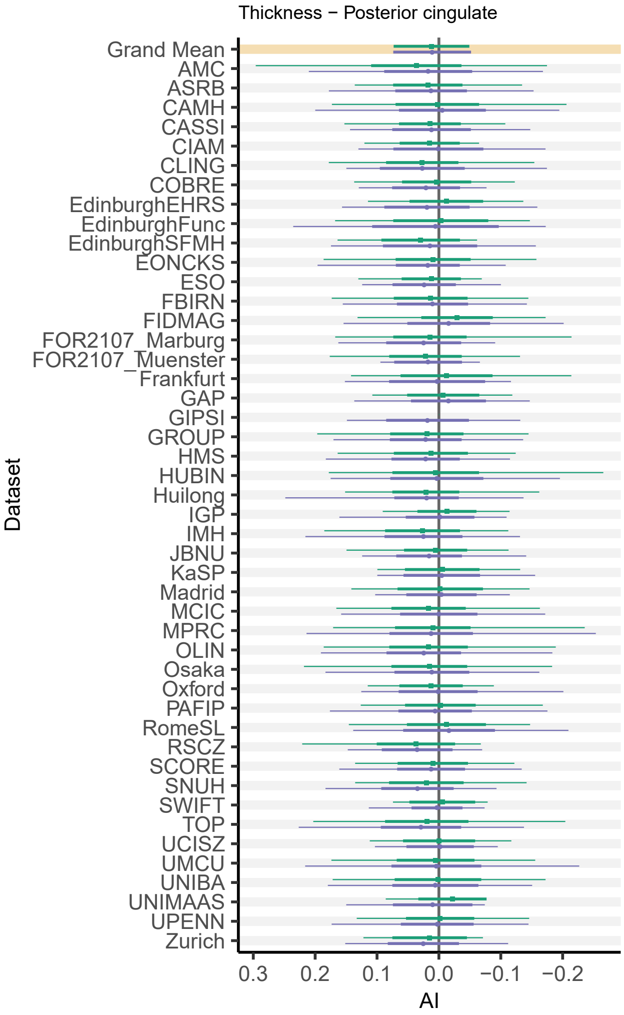
Thickness − Posterior cingulate
Grand Mean
AMC
ASRB
CAMH
CASSI
CIAM
CLING
COBRE
EdinburghEHRS
EdinburghFunc
EdinburghSFMH
EONCKS
ESO
FBIRN
FIDMAG
FOR2107_Marburg
FOR2107_Muenster
Frankfurt
GAP
GIPSI
GROUP
HMS
HUBIN
Huilong
IGP
IMH
JBNU
KaSP
Madrid
MCIC
MPRC
OLIN
Osaka
Oxford
PAFIP
RomeSL
RSCZ
SCORE
SNUH
SWIFT
TOP
UCISZ
UMCU
UNIBA
UNIMAAS
UPENN
Zurich
Dataset
0.3
0.2
0.1
0.0
−0.1
−0.2
AI

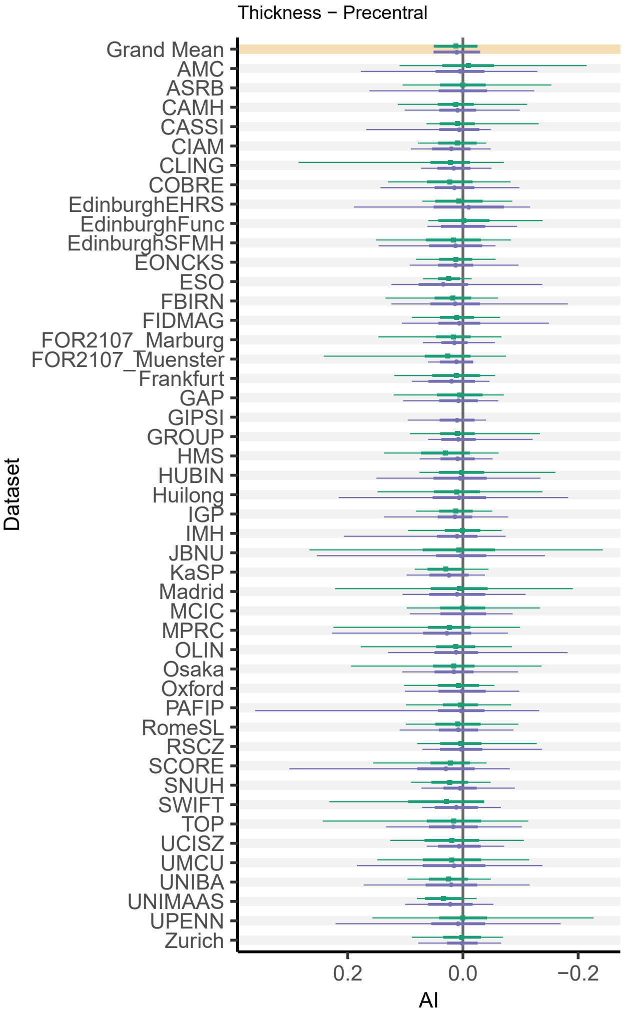
Thickness − Precentral
Grand Mean
AMC
ASRB
CAMH
CASSI
CIAM
CLING
COBRE
EdinburghEHRS
EdinburghFunc
EdinburghSFMH
EONCKS
ESO
FBIRN
FIDMAG
FOR2107_Marburg
FOR2107_Muenster
Frankfurt
GAP
GIPSI
GROUP
HMS
HUBIN
Huilong
IGP
IMH
JBNU
KaSP
Madrid
MCIC
MPRC
OLIN
Osaka
Oxford
PAFIP
RomeSL
RSCZ
SCORE
SNUH
SWIFT
TOP
UCISZ
UMCU
UNIBA
UNIMAAS
UPENN
Zurich
Dataset
0.2
0.0
−0.2
AI

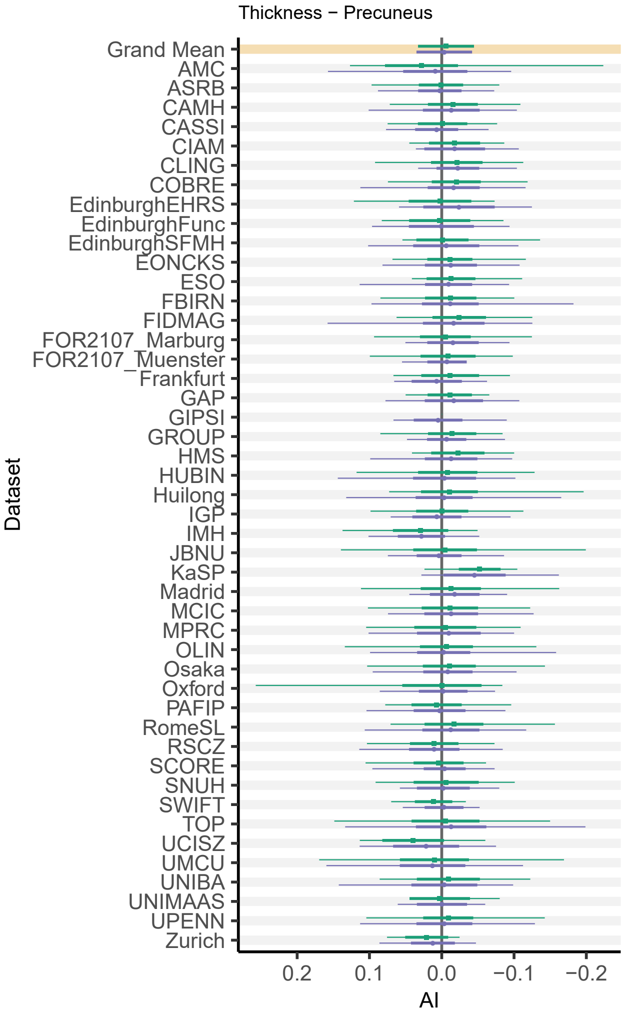
Thickness − Precuneus
Grand Mean
AMC
ASRB
CAMH
CASSI
CIAM
CLING
COBRE
EdinburghEHRS
EdinburghFunc
EdinburghSFMH
EONCKS
ESO
FBIRN
FIDMAG
FOR2107_Marburg
FOR2107_Muenster
Frankfurt
GAP
GIPSI
GROUP
HMS
HUBIN
Huilong
IGP
IMH
JBNU
KaSP
Madrid
MCIC
MPRC
OLIN
Osaka
Oxford
PAFIP
RomeSL
RSCZ
SCORE
SNUH
SWIFT
TOP
UCISZ
UMCU
UNIBA
UNIMAAS
UPENN
Zurich
Dataset
0.2
0.1
0.0
−0.1
−0.2
AI

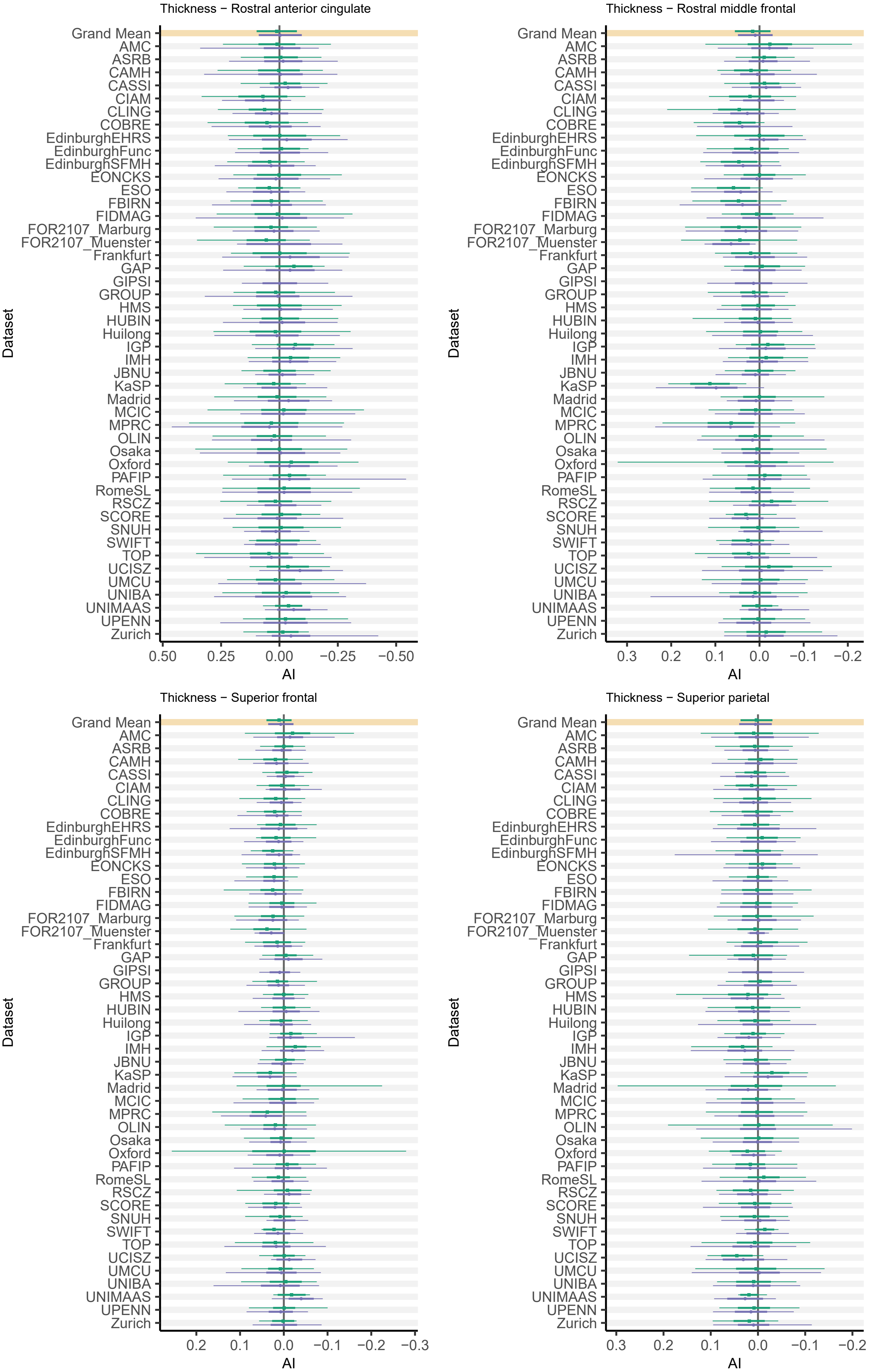
Thickness − Rostral anterior cingulate
Thickness − Rostral middle frontal
Thickness − Superior frontal
Thickness − Superior parietal
Dataset
Grand Mean
AMC
ASRB
CAMH
CASSI
CIAM
CLING
COBRE
EdinburghEHRS
EdinburghFunc
EdinburghSFMH
EONCKS
ESO
FBIRN
FIDMAG
FOR2107_Marburg
FOR2107_Muenster
Frankfurt
GAP
GIPSI
GROUP
HMS
HUBIN
Huilong
IGP
IMH
JBNU
KaSP
Madrid
MCIC
MPRC
OLIN
Osaka
Oxford
PAFIP
RomeSL
RSCZ
SCORE
SNUH
SWIFT
TOP
UCISZ
UMCU
UNIBA
UNIMAAS
UPENN
Zurich
0.50 0.25 0.00 −0.25 −0.50
0.3 0.2 0.1 0.0 −0.1 −0.2
0.2 0.1 0.0 −0.1 −0.2 −0.3
AI

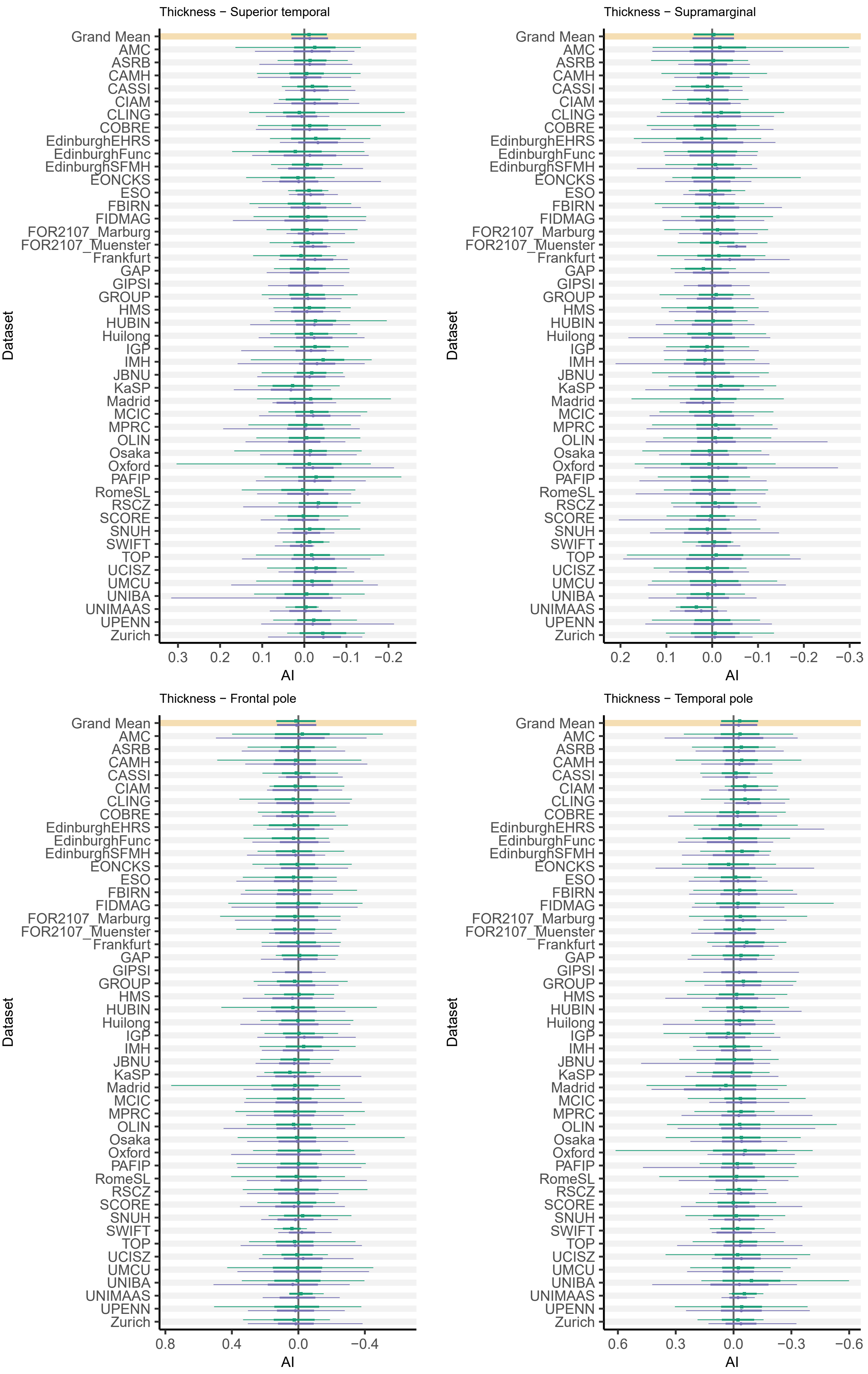
Thickness − Superior temporal
Grand Mean
AMC
ASRB
CAMH
CASSI
CIAM
CLING
COBRE
EdinburghEHRS
EdinburghFunc
EdinburghSFMH
EONCKS
ESO
FBIRN
FIDMAG
FOR2107_Marburg
FOR2107_Muenster
Frankfurt
GAP
GIPSI
GROUP
HMS
HUBIN
Huilong
IGP
IMH
JBNU
KaSP
Madrid
MCIC
MPRC
OLIN
Osaka
Oxford
PAFIP
RomeSL
RSCZ
SCORE
SNUH
SWIFT
TOP
UCISZ
UMCU
UNIBA
UNIMAAS
UPENN
Zurich
Dataset
0.3
0.2
0.1
0.0
−0.1
−0.2
AI
Thickness − Supramarginal
Grand Mean
AMC
ASRB
CAMH
CASSI
CIAM
CLING
COBRE
EdinburghEHRS
EdinburghFunc
EdinburghSFMH
EONCKS
ESO
FBIRN
FIDMAG
FOR2107_Marburg
FOR2107_Muenster
Frankfurt
GAP
GIPSI
GROUP
HMS
HUBIN
Huilong
IGP
IMH
JBNU
KaSP
Madrid
MCIC
MPRC
OLIN
Osaka
Oxford
PAFIP
RomeSL
RSCZ
SCORE
SNUH
SWIFT
TOP
UCISZ
UMCU
UNIBA
UNIMAAS
UPENN
Zurich
Dataset
0.2
0.1
0.0
−0.1
−0.2
−0.3
AI
Thickness − Frontal pole
Grand Mean
AMC
ASRB
CAMH
CASSI
CIAM
CLING
COBRE
EdinburghEHRS
EdinburghFunc
EdinburghSFMH
EONCKS
ESO
FBIRN
FIDMAG
FOR2107_Marburg
FOR2107_Muenster
Frankfurt
GAP
GIPSI
GROUP
HMS
HUBIN
Huilong
IGP
IMH
JBNU
KaSP
Madrid
MCIC
MPRC
OLIN
Osaka
Oxford
PAFIP
RomeSL
RSCZ
SCORE
SNUH
SWIFT
TOP
UCISZ
UMCU
UNIBA
UNIMAAS
UPENN
Zurich
Dataset
0.8
0.4
0.0
−0.4
AI
Thickness − Temporal pole
Grand Mean
AMC
ASRB
CAMH
CASSI
CIAM
CLING
COBRE
EdinburghEHRS
EdinburghFunc
EdinburghSFMH
EONCKS
ESO
FBIRN
FIDMAG
FOR2107_Marburg
FOR2107_Muenster
Frankfurt
GAP
GIPSI
GROUP
HMS
HUBIN
Huilong
IGP
IMH
JBNU
KaSP
Madrid
MCIC
MPRC
OLIN
Osaka
Oxford
PAFIP
RomeSL
RSCZ
SCORE
SNUH
SWIFT
TOP
UCISZ
UMCU
UNIBA
UNIMAAS
UPENN
Zurich
Dataset
0.6
0.3
0.0
−0.3
−0.6
AI

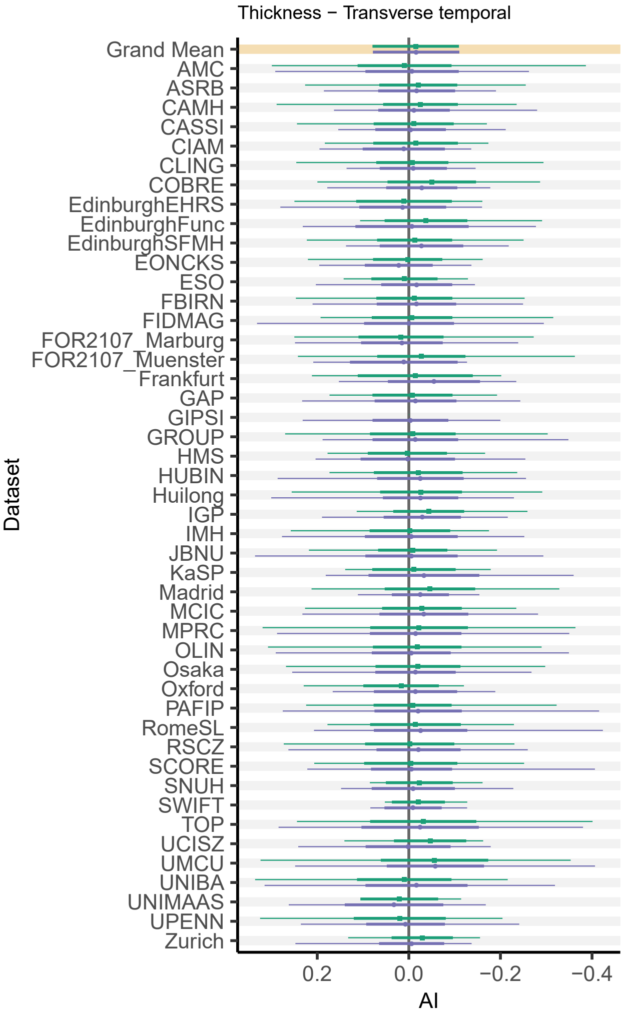
Thickness − Transverse temporal
Grand Mean
AMC
ASRB
CAMH
CASSI
CIAM
CLING
COBRE
EdinburghEHRS
EdinburghFunc
EdinburghSFMH
EONCKS
ESO
FBIRN
FIDMAG
FOR2107_Marburg
FOR2107_Muenster
Frankfurt
GAP
GIPSI
GROUP
HMS
HUBIN
Huilong
IGP
IMH
JBNU
KaSP
Madrid
MCIC
MPRC
OLIN
Osaka
Oxford
PAFIP
RomeSL
RSCZ
SCORE
SNUH
SWIFT
TOP
UCISZ
UMCU
UNIBA
UNIMAAS
UPENN
Zurich
Dataset
0.2
0.0
−0.2
−0.4
AI

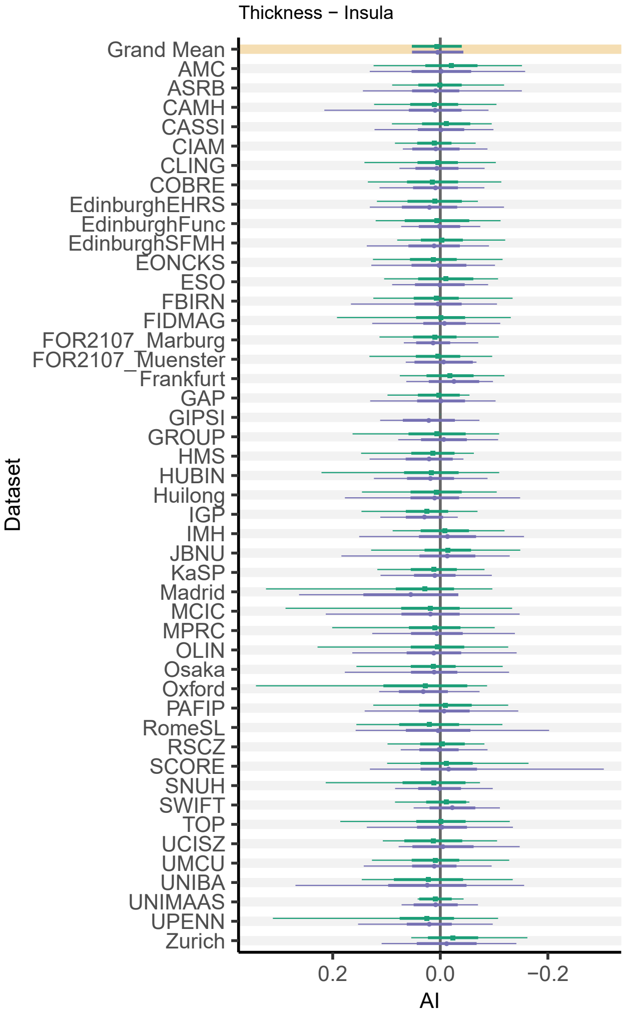
Thickness − Insula
Grand Mean
AMC
ASRB
CAMH
CASSI
CIAM
CLING
COBRE
EdinburghEHRS
EdinburghFunc
EdinburghSFMH
EONCKS
ESO
FBIRN
FIDMAG
FOR2107_Marburg
FOR2107_Muenster
Frankfurt
GAP
GIPSI
GROUP
HMS
HUBIN
Huilong
IGP
IMH
JBNU
KaSP
Madrid
MCIC
MPRC
OLIN
Osaka
Oxford
PAFIP
RomeSL
RSCZ
SCORE
SNUH
SWIFT
TOP
UCISZ
UMCU
UNIBA
UNIMAAS
UPENN
Zurich
Dataset
0.2
0.0
−0.2
AI

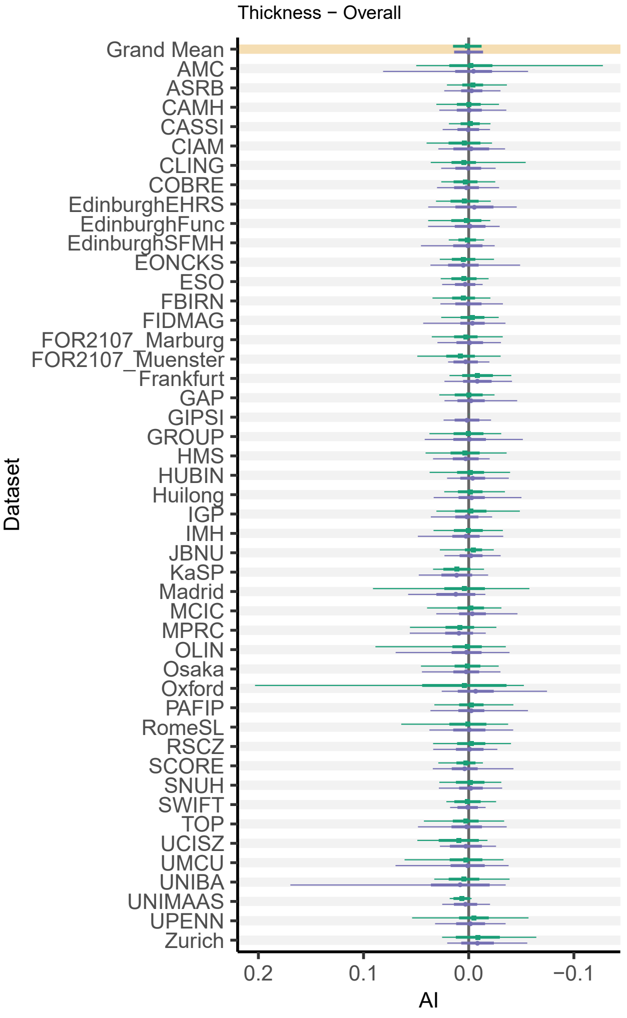
Thickness − Overall
Grand Mean
AMC
ASRB
CAMH
CASSI
CIAM
CLING
COBRE
EdinburghEHRS
EdinburghFunc
EdinburghSFMH
EONCKS
ESO
FBIRN
FIDMAG
FOR2107_Marburg
FOR2107_Muenster
Frankfurt
GAP
GIPSI
GROUP
HMS
HUBIN
Huilong
IGP
IMH
JBNU
KaSP
Madrid
MCIC
MPRC
OLIN
Osaka
Oxford
PAFIP
RomeSL
RSCZ
SCORE
SNUH
SWIFT
TOP
UCISZ
UMCU
UNIBA
UNIMAAS
UPENN
Zurich
Dataset
0.2
0.1
0.0
−0.1
AI

Fig. S3 (page 20-28). Overall and per-dataset average and range for cortical surface area asymmetries. For each cortical surface area asymmetry measure, the average in controls (green circles) and individuals affected with schizophrenia (purple squares) is shown. The top (highlighted) row contains the grand sample size-weighted mean and standard deviation (thick line segments). The other rows contain per-dataset averages, standard deviations and minimum and maximum values (indicated with thin line segments).

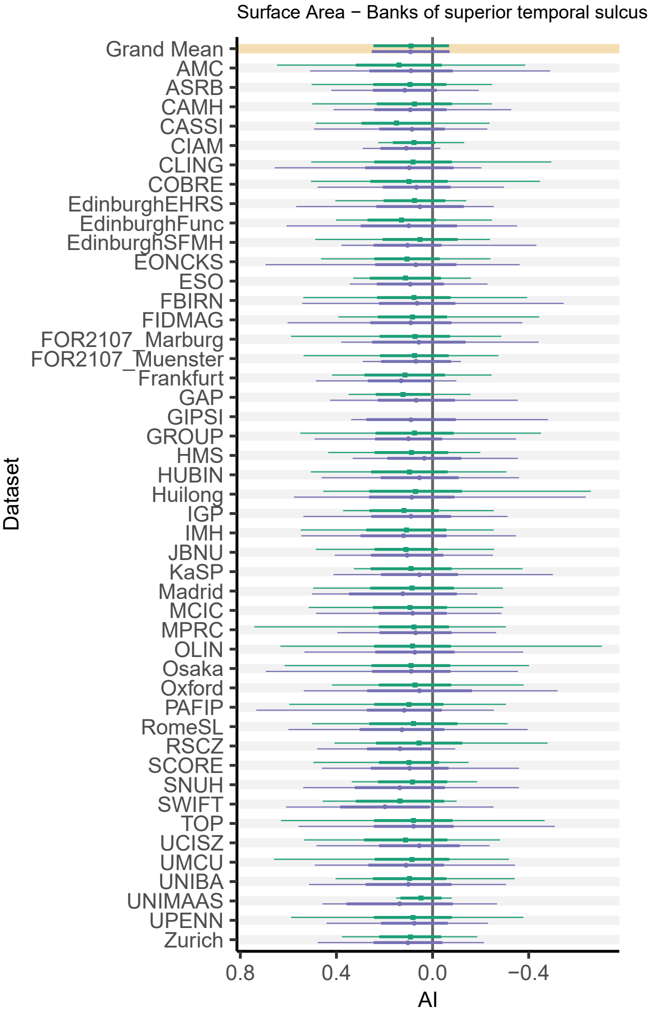
Surface Area − Banks of superior temporal sulcus
Dataset
Grand Mean
AMC
ASRB
CAMH
CASSI
CIAM
CLING
COBRE
EdinburghEHRS
EdinburghFunc
EdinburghSFMH
EONCKS
ESO
FBIRN
FIDMAG
FOR2107_Marburg
FOR2107_Muenster
Frankfurt
GAP
GIPSI
GROUP
HMS
HUBIN
Huilong
IGP
IMH
JBNU
KaSP
Madrid
MCIC
MPRC
OLIN
Osaka
Oxford
PAFIP
RomeSL
RSCZ
SCORE
SNUH
SWIFT
TOP
UCISZ
UMCU
UNIBA
UNIMAAS
UPENN
Zurich
0.8
0.4
0.0
−0.4
AI

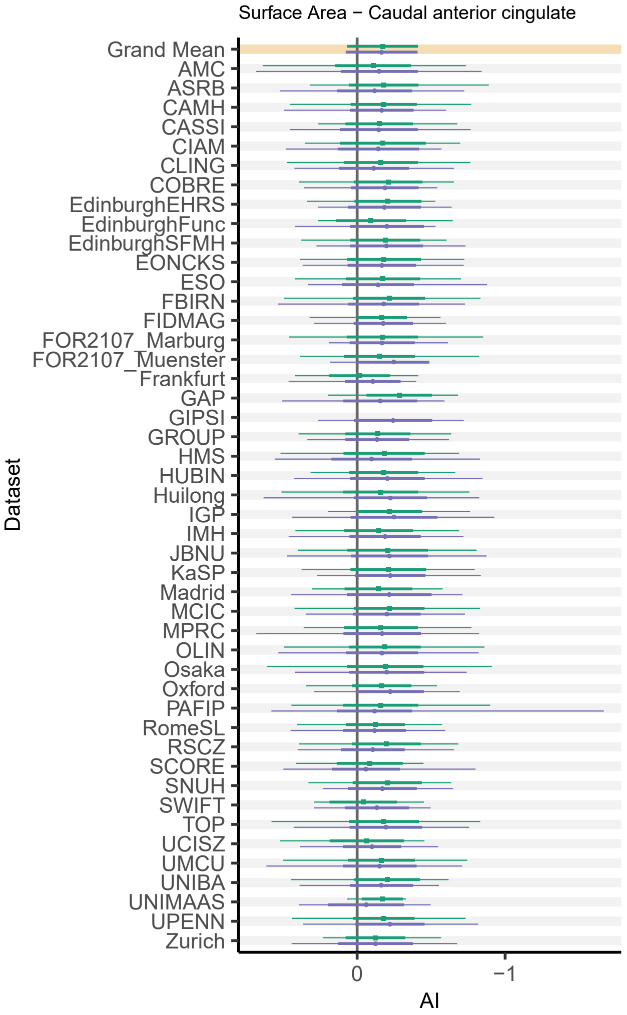
Surface Area − Caudal anterior cingulate
Dataset
Grand Mean
AMC
ASRB
CAMH
CASSI
CIAM
CLING
COBRE
EdinburghEHRS
EdinburghFunc
EdinburghSFMH
EONCKS
ESO
FBIRN
FIDMAG
FOR2107_Marburg
FOR2107_Muenster
Frankfurt
GAP
GIPSI
GROUP
HMS
HUBIN
Huilong
IGP
IMH
JBNU
KaSP
Madrid
MCIC
MPRC
OLIN
Osaka
Oxford
PAFIP
RomeSL
RSCZ
SCORE
SNUH
SWIFT
TOP
UCISZ
UMCU
UNIBA
UNIMAAS
UPENN
Zurich
0
−1
AI

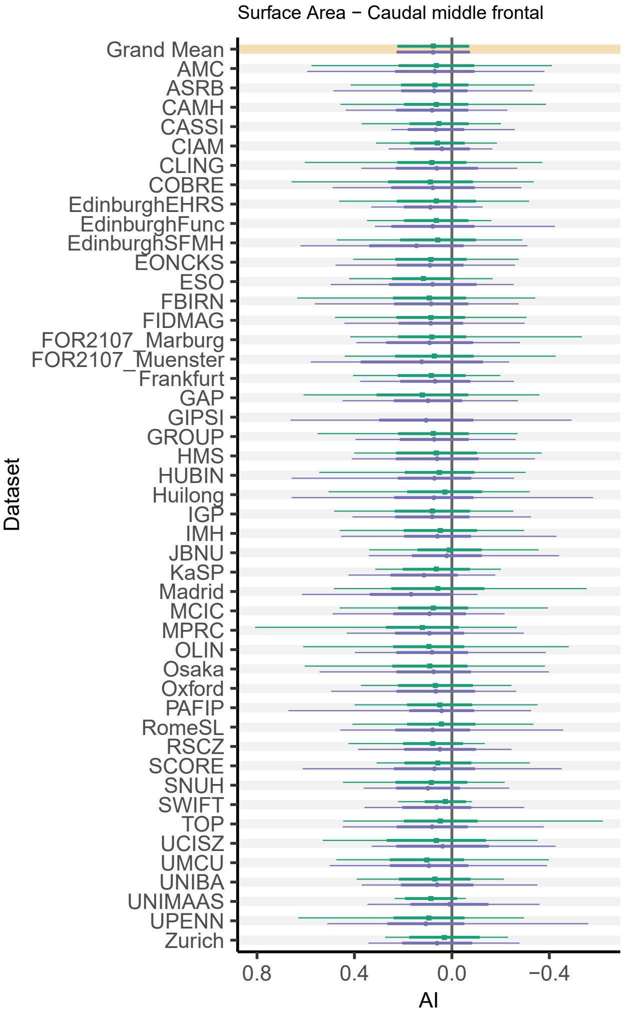
Surface Area − Caudal middle frontal
Dataset
Grand Mean
AMC
ASRB
CAMH
CASSI
CIAM
CLING
COBRE
EdinburghEHRS
EdinburghFunc
EdinburghSFMH
EONCKS
ESO
FBIRN
FIDMAG
FOR2107_Marburg
FOR2107_Muenster
Frankfurt
GAP
GIPSI
GROUP
HMS
HUBIN
Huilong
IGP
IMH
JBNU
KaSP
Madrid
MCIC
MPRC
OLIN
Osaka
Oxford
PAFIP
RomeSL
RSCZ
SCORE
SNUH
SWIFT
TOP
UCISZ
UMCU
UNIBA
UNIMAAS
UPENN
Zurich
0.8
0.4
0.0
−0.4
AI

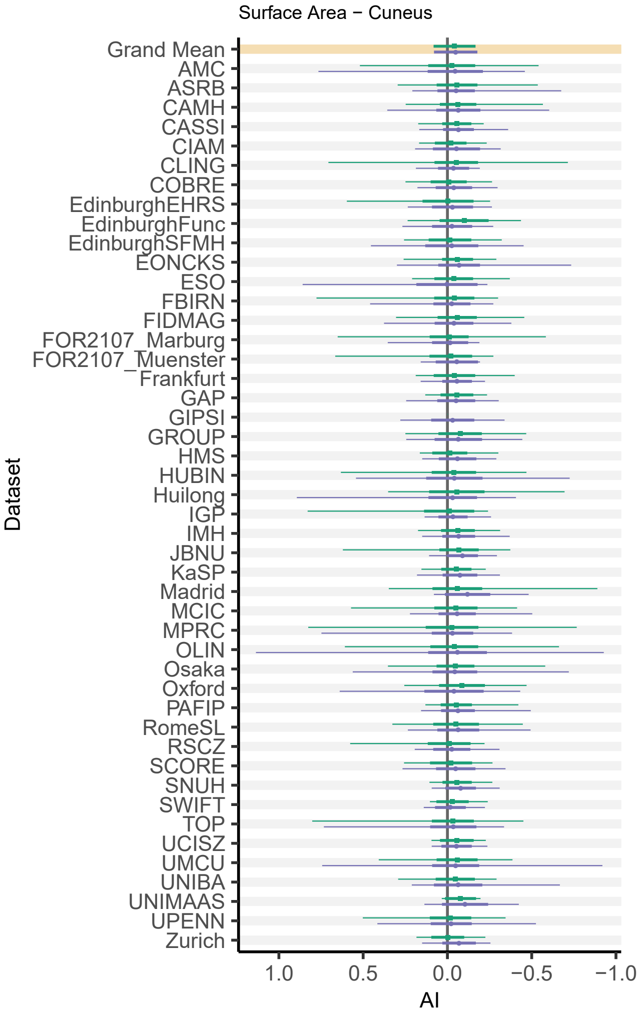
Surface Area − Cuneus
Dataset
Grand Mean
AMC
ASRB
CAMH
CASSI
CIAM
CLING
COBRE
EdinburghEHRS
EdinburghFunc
EdinburghSFMH
EONCKS
ESO
FBIRN
FIDMAG
FOR2107_Marburg
FOR2107_Muenster
Frankfurt
GAP
GIPSI
GROUP
HMS
HUBIN
Huilong
IGP
IMH
JBNU
KaSP
Madrid
MCIC
MPRC
OLIN
Osaka
Oxford
PAFIP
RomeSL
RSCZ
SCORE
SNUH
SWIFT
TOP
UCISZ
UMCU
UNIBA
UNIMAAS
UPENN
Zurich
1.0
0.5
0.0
−0.5
−1.0
AI

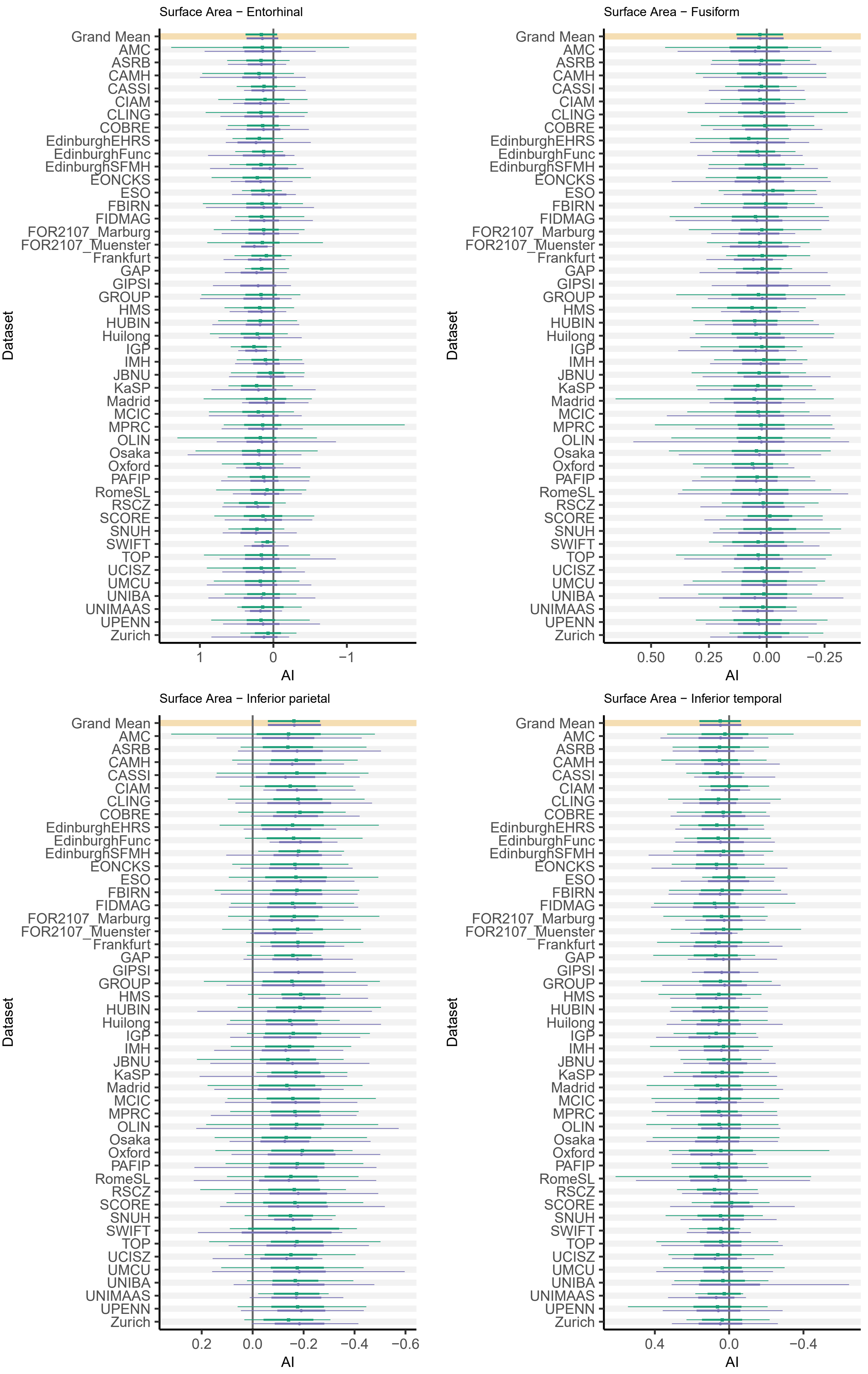

Surface Area − Entorhinal
Surface Area − Fusiform
Surface Area − Inferior parietal
Surface Area − Inferior temporal
Grand Mean
AMC
ASRB
CAMH
CASSI
CIAM
CLING
COBRE
EdinburghEHRS
EdinburghFunc
EdinburghSFMH
EONCKS
ESO
FBIRN
FIDMAG
FOR2107_Marburg
FOR2107_Muenster
Frankfurt
GAP
GIPSI
GROUP
HMS
HUBIN
Huilong
IGP
IMH
JBNU
KaSP
Madrid
MCIC
MPRC
OLIN
Osaka
Oxford
PAFIP
RomeSL
RSCZ
SCORE
SNUH
SWIFT
TOP
UCISZ
UMCU
UNIBA
UNIMAAS
UPENN
Zurich
Dataset
AI
1 0 −1
0.50 0.25 0.00 −0.25
0.2 0.0 −0.2 −0.4 −0.6
0.4 0.0 −0.4

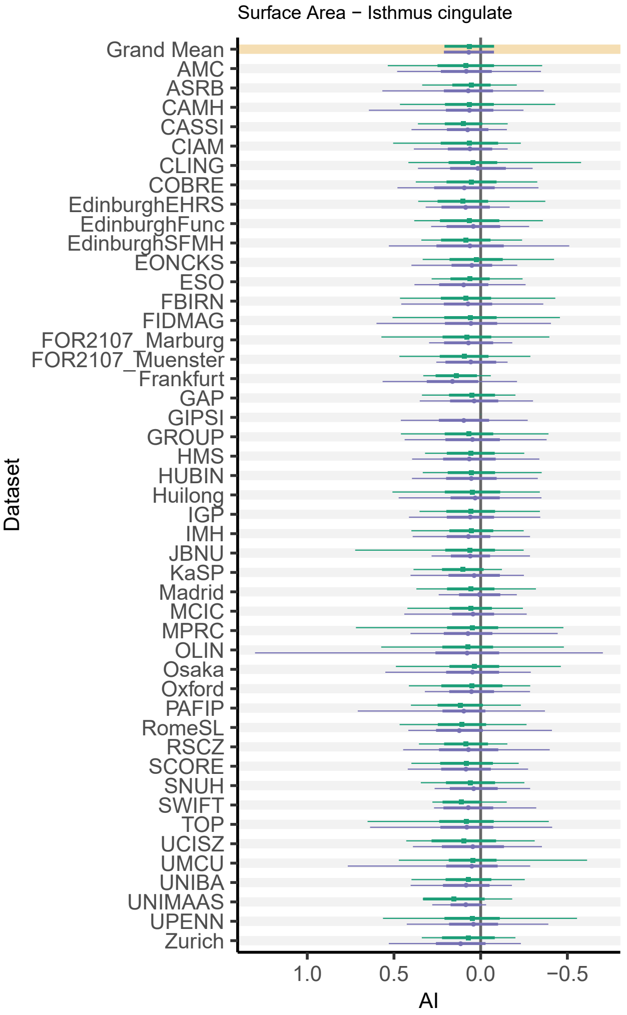
Surface Area − Isthmus cingulate
Grand Mean
AMC
ASRB
CAMH
CASSI
CIAM
CLING
COBRE
EdinburghEHRS
EdinburghFunc
EdinburghSFMH
EONCKS
ESO
FBIRN
FIDMAG
FOR2107_Marburg
FOR2107_Muenster
Frankfurt
GAP
GIPSI
GROUP
HMS
HUBIN
Huilong
IGP
IMH
JBNU
KaSP
Madrid
MCIC
MPRC
OLIN
Osaka
Oxford
PAFIP
RomeSL
RSCZ
SCORE
SNUH
SWIFT
TOP
UCISZ
UMCU
UNIBA
UNIMAAS
UPENN
Zurich
Dataset
1.0
0.5
0.0
−0.5
AI

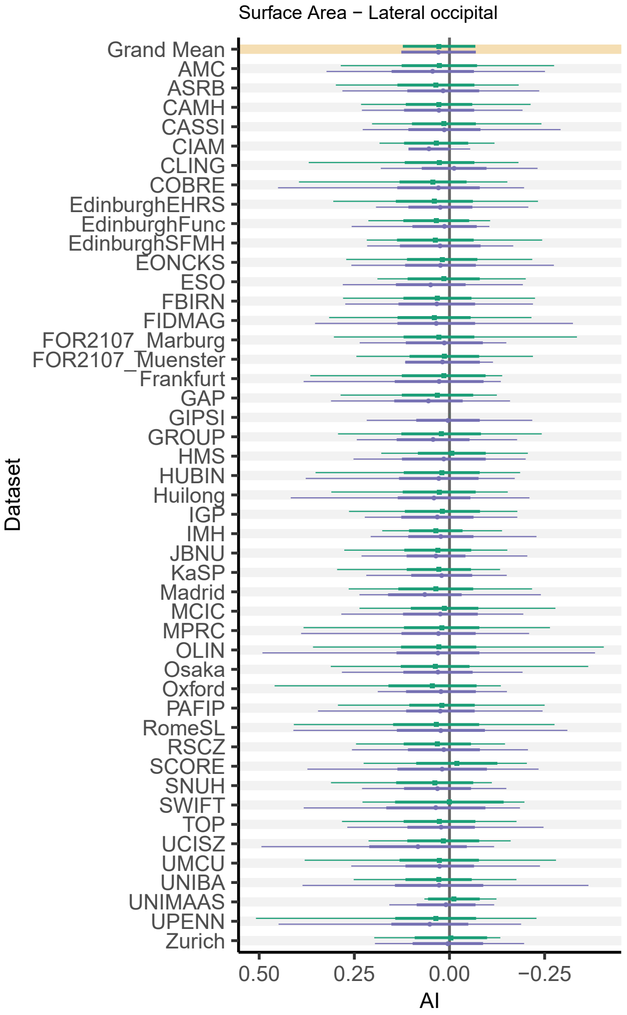
Surface Area − Lateral occipital
Grand Mean
AMC
ASRB
CAMH
CASSI
CIAM
CLING
COBRE
EdinburghEHRS
EdinburghFunc
EdinburghSFMH
EONCKS
ESO
FBIRN
FIDMAG
FOR2107_Marburg
FOR2107_Muenster
Frankfurt
GAP
GIPSI
GROUP
HMS
HUBIN
Huilong
IGP
IMH
JBNU
KaSP
Madrid
MCIC
MPRC
OLIN
Osaka
Oxford
PAFIP
RomeSL
RSCZ
SCORE
SNUH
SWIFT
TOP
UCISZ
UMCU
UNIBA
UNIMAAS
UPENN
Zurich
Dataset
0.50
0.25
0.00
−0.25
AI

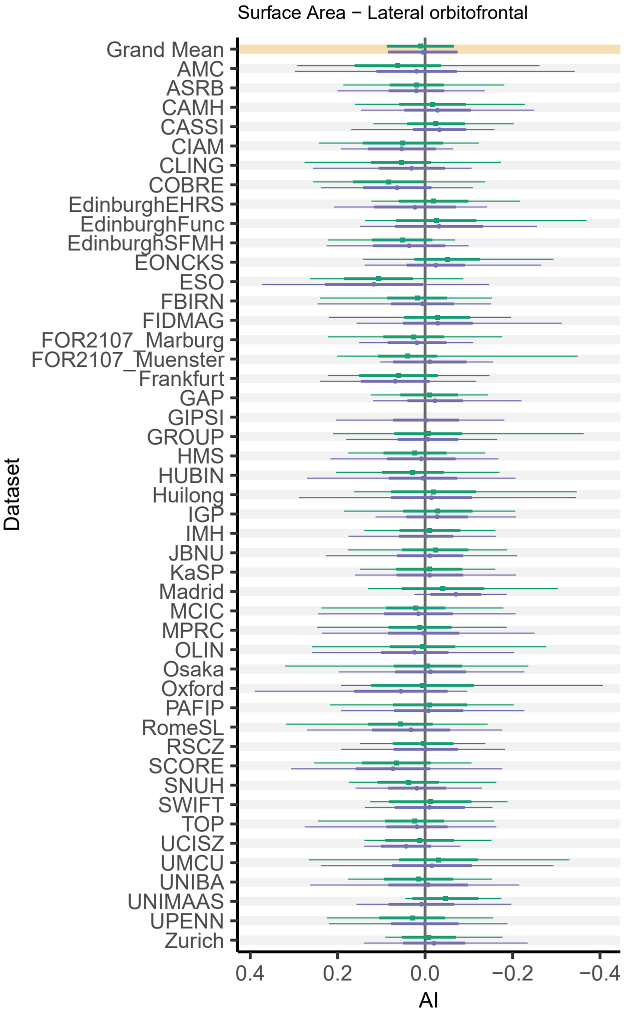
Surface Area − Lateral orbitofrontal
Grand Mean
AMC
ASRB
CAMH
CASSI
CIAM
CLING
COBRE
EdinburghEHRS
EdinburghFunc
EdinburghSFMH
EONCKS
ESO
FBIRN
FIDMAG
FOR2107_Marburg
FOR2107_Muenster
Frankfurt
GAP
GIPSI
GROUP
HMS
HUBIN
Huilong
IGP
IMH
JBNU
KaSP
Madrid
MCIC
MPRC
OLIN
Osaka
Oxford
PAFIP
RomeSL
RSCZ
SCORE
SNUH
SWIFT
TOP
UCISZ
UMCU
UNIBA
UNIMAAS
UPENN
Zurich
Dataset
0.4
0.2
0.0
−0.2
−0.4
AI

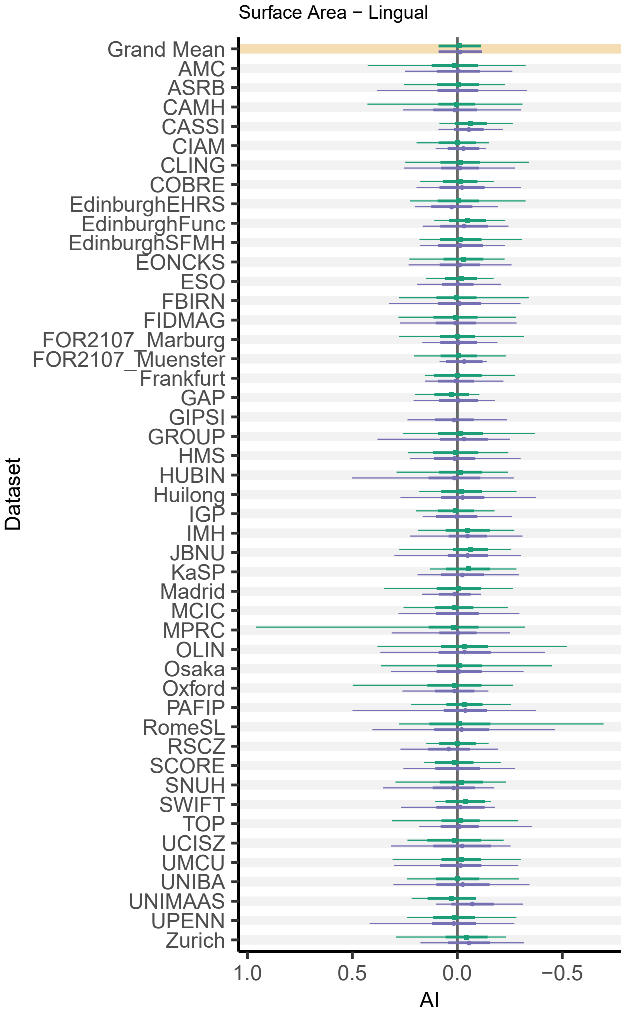
Surface Area − Lingual
Grand Mean
AMC
ASRB
CAMH
CASSI
CIAM
CLING
COBRE
EdinburghEHRS
EdinburghFunc
EdinburghSFMH
EONCKS
ESO
FBIRN
FIDMAG
FOR2107_Marburg
FOR2107_Muenster
Frankfurt
GAP
GIPSI
GROUP
HMS
HUBIN
Huilong
IGP
IMH
JBNU
KaSP
Madrid
MCIC
MPRC
OLIN
Osaka
Oxford
PAFIP
RomeSL
RSCZ
SCORE
SNUH
SWIFT
TOP
UCISZ
UMCU
UNIBA
UNIMAAS
UPENN
Zurich
Dataset
1.0
0.5
0.0
−0.5
AI

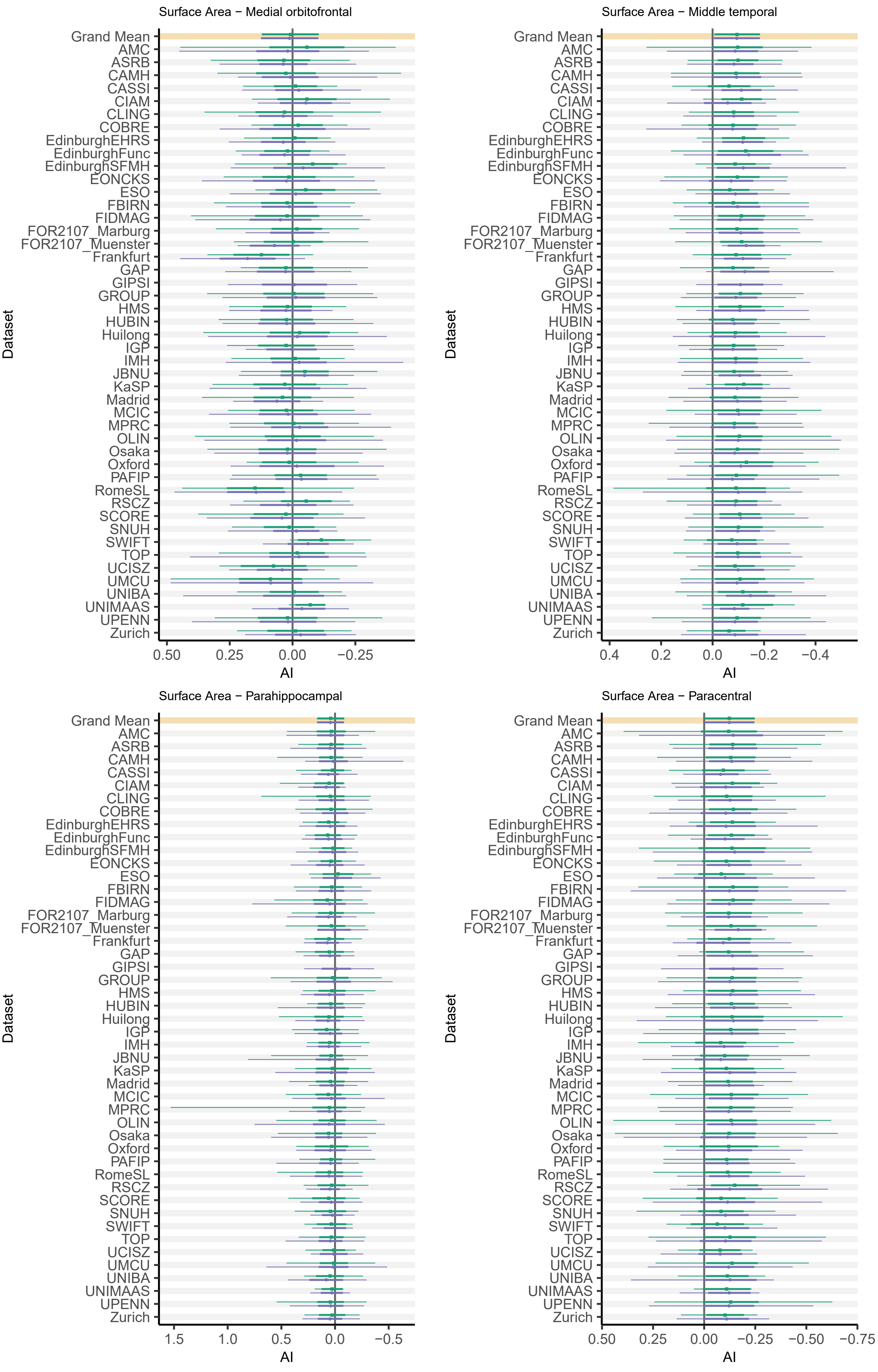

Surface Area − Medial orbitofrontal
Surface Area − Middle temporal
Surface Area − Parahippocampal
Surface Area − Paracentral
Dataset
Grand Mean
AMC
ASRB
CAMH
CASSI
CIAM
CLING
COBRE
EdinburghEHRS
EdinburghFunc
EdinburghSFMH
EONCKS
ESO
FBIRN
FIDMAG
FOR2107_Marburg
FOR2107_Muenster
Frankfurt
GAP
GIPSI
GROUP
HMS
HUBIN
Huilong
IGP
IMH
JBNU
KaSP
Madrid
MCIC
MPRC
OLIN
Osaka
Oxford
PAFIP
RomeSL
RSCZ
SCORE
SNUH
SWIFT
TOP
UCISZ
UMCU
UNIBA
UNIMAAS
UPENN
Zurich
0.50 0.25 0.00 −0.25
0.4 0.2 0.0 −0.2 −0.4
1.5 1.0 0.5 0.0 −0.5
0.50 0.25 0.00 −0.25 −0.50 −0.75
AI

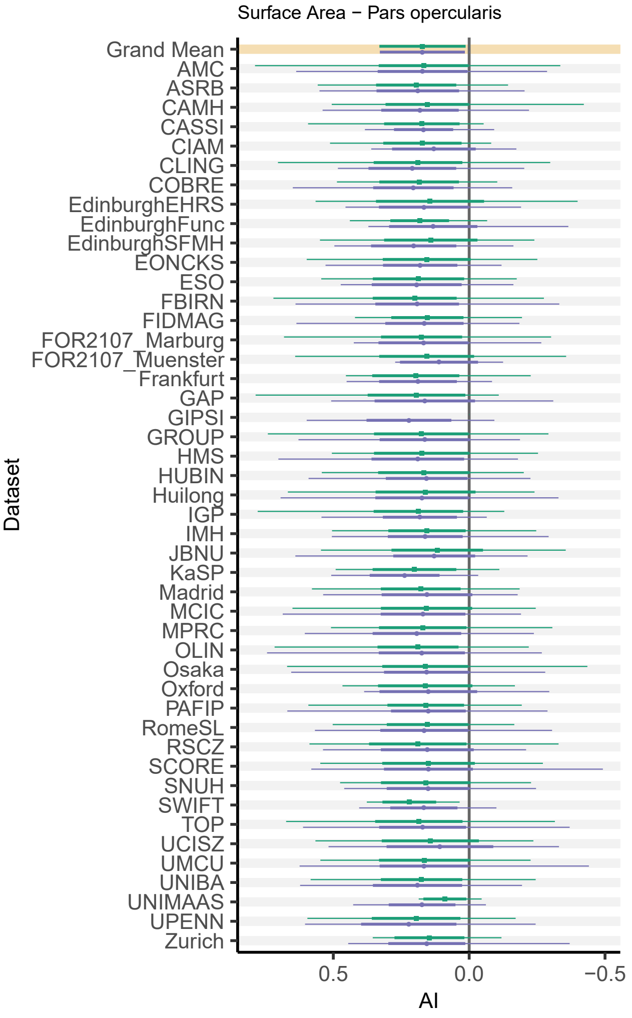
Surface Area − Pars opercularis
Grand Mean
AMC
ASRB
CAMH
CASSI
CIAM
CLING
COBRE
EdinburghEHRS
EdinburghFunc
EdinburghSFMH
EONCKS
ESO
FBIRN
FIDMAG
FOR2107_Marburg
FOR2107_Muenster
Frankfurt
GAP
GIPSI
GROUP
HMS
HUBIN
Huilong
IGP
IMH
JBNU
KaSP
Madrid
MCIC
MPRC
OLIN
Osaka
Oxford
PAFIP
RomeSL
RSCZ
SCORE
SNUH
SWIFT
TOP
UCISZ
UMCU
UNIBA
UNIMAAS
UPENN
Zurich
Dataset
0.5
0.0
−0.5
AI

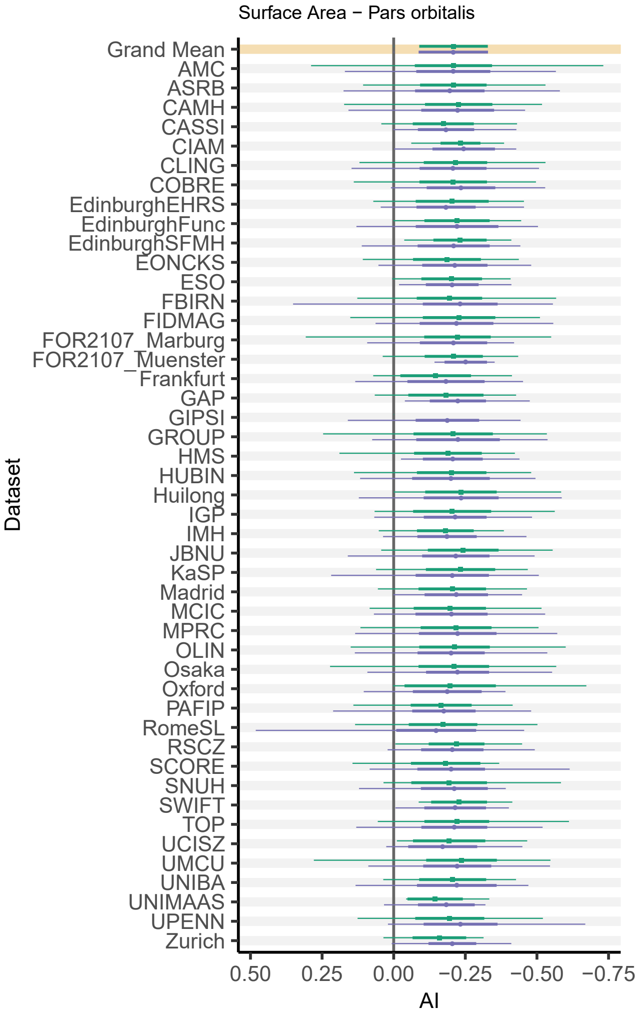
Surface Area − Pars orbitalis
Grand Mean
AMC
ASRB
CAMH
CASSI
CIAM
CLING
COBRE
EdinburghEHRS
EdinburghFunc
EdinburghSFMH
EONCKS
ESO
FBIRN
FIDMAG
FOR2107_Marburg
FOR2107_Muenster
Frankfurt
GAP
GIPSI
GROUP
HMS
HUBIN
Huilong
IGP
IMH
JBNU
KaSP
Madrid
MCIC
MPRC
OLIN
Osaka
Oxford
PAFIP
RomeSL
RSCZ
SCORE
SNUH
SWIFT
TOP
UCISZ
UMCU
UNIBA
UNIMAAS
UPENN
Zurich
Dataset
0.50
0.25
0.00
−0.25
−0.50
−0.75
AI

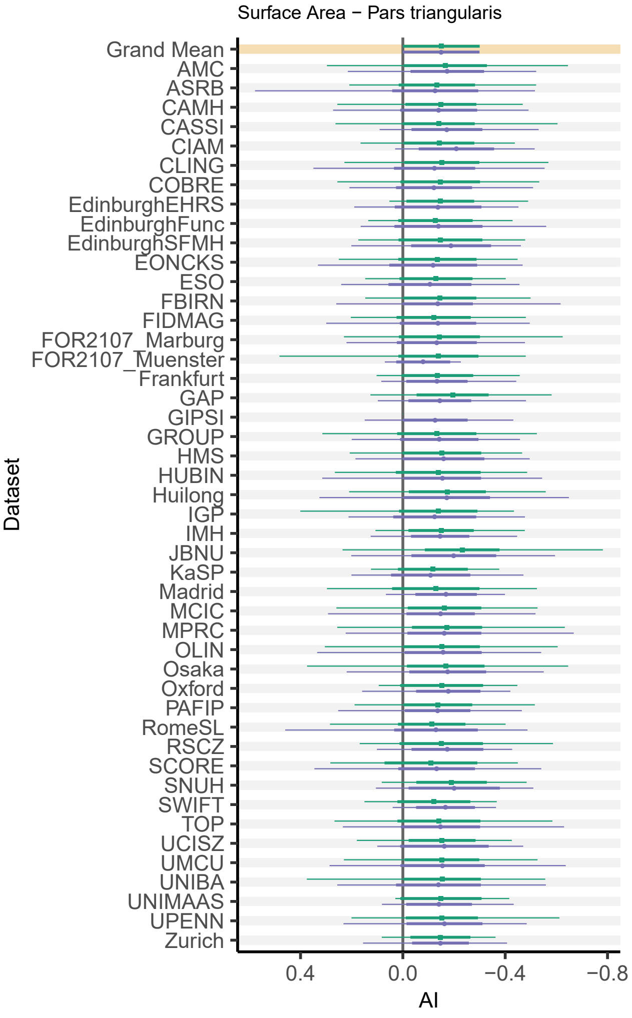
Surface Area − Pars triangularis
Grand Mean
AMC
ASRB
CAMH
CASSI
CIAM
CLING
COBRE
EdinburghEHRS
EdinburghFunc
EdinburghSFMH
EONCKS
ESO
FBIRN
FIDMAG
FOR2107_Marburg
FOR2107_Muenster
Frankfurt
GAP
GIPSI
GROUP
HMS
HUBIN
Huilong
IGP
IMH
JBNU
KaSP
Madrid
MCIC
MPRC
OLIN
Osaka
Oxford
PAFIP
RomeSL
RSCZ
SCORE
SNUH
SWIFT
TOP
UCISZ
UMCU
UNIBA
UNIMAAS
UPENN
Zurich
Dataset
0.4
0.0
−0.4
−0.8
AI

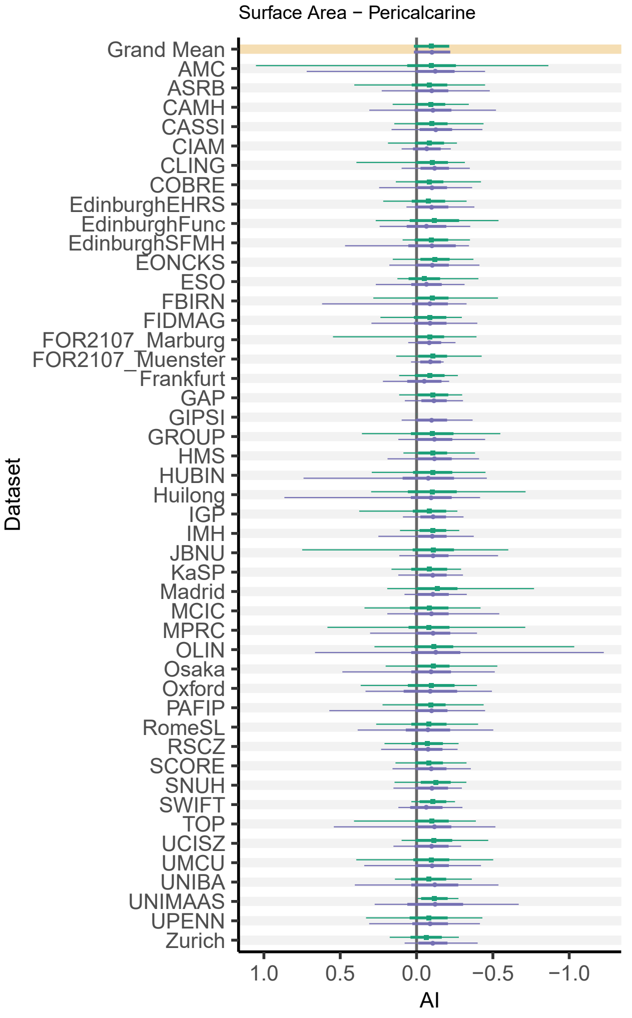
Surface Area − Pericalcarine
Grand Mean
AMC
ASRB
CAMH
CASSI
CIAM
CLING
COBRE
EdinburghEHRS
EdinburghFunc
EdinburghSFMH
EONCKS
ESO
FBIRN
FIDMAG
FOR2107_Marburg
FOR2107_Muenster
Frankfurt
GAP
GIPSI
GROUP
HMS
HUBIN
Huilong
IGP
IMH
JBNU
KaSP
Madrid
MCIC
MPRC
OLIN
Osaka
Oxford
PAFIP
RomeSL
RSCZ
SCORE
SNUH
SWIFT
TOP
UCISZ
UMCU
UNIBA
UNIMAAS
UPENN
Zurich
Dataset
1.0
0.5
0.0
−0.5
−1.0
AI

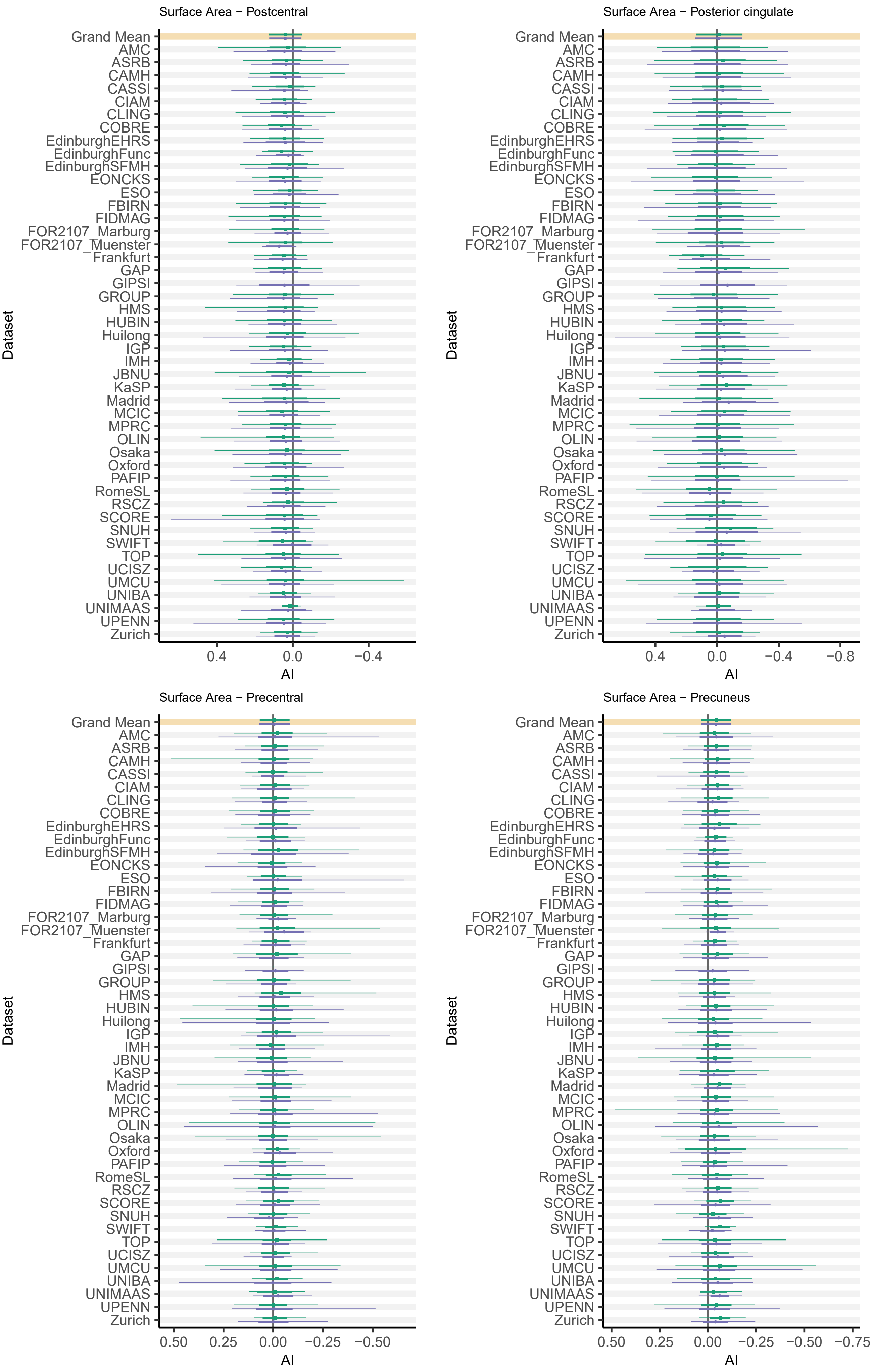
Surface Area − Postcentral
Dataset
Grand Mean
AMC
ASRB
CAMH
CASSI
CIAM
CLING
COBRE
EdinburghEHRS
EdinburghFunc
EdinburghSFMH
EONCKS
ESO
FBIRN
FIDMAG
FOR2107_Marburg
FOR2107_Muenster
Frankfurt
GAP
GIPSI
GROUP
HMS
HUBIN
Huilong
IGP
IMH
JBNU
KaSP
Madrid
MCIC
MPRC
OLIN
Osaka
Oxford
PAFIP
RomeSL
RSCZ
SCORE
SNUH
SWIFT
TOP
UCISZ
UMCU
UNIBA
UNIMAAS
UPENN
Zurich
0.4
0.0
−0.4
AI
Surface Area − Posterior cingulate
Dataset
0.4
0.0
−0.4
−0.8
AI
Surface Area − Precentral
Dataset
0.50
0.25
0.00
−0.25
−0.50
AI
Surface Area − Precuneus
Dataset
0.50
0.25
0.00
−0.25
−0.50
−0.75
AI

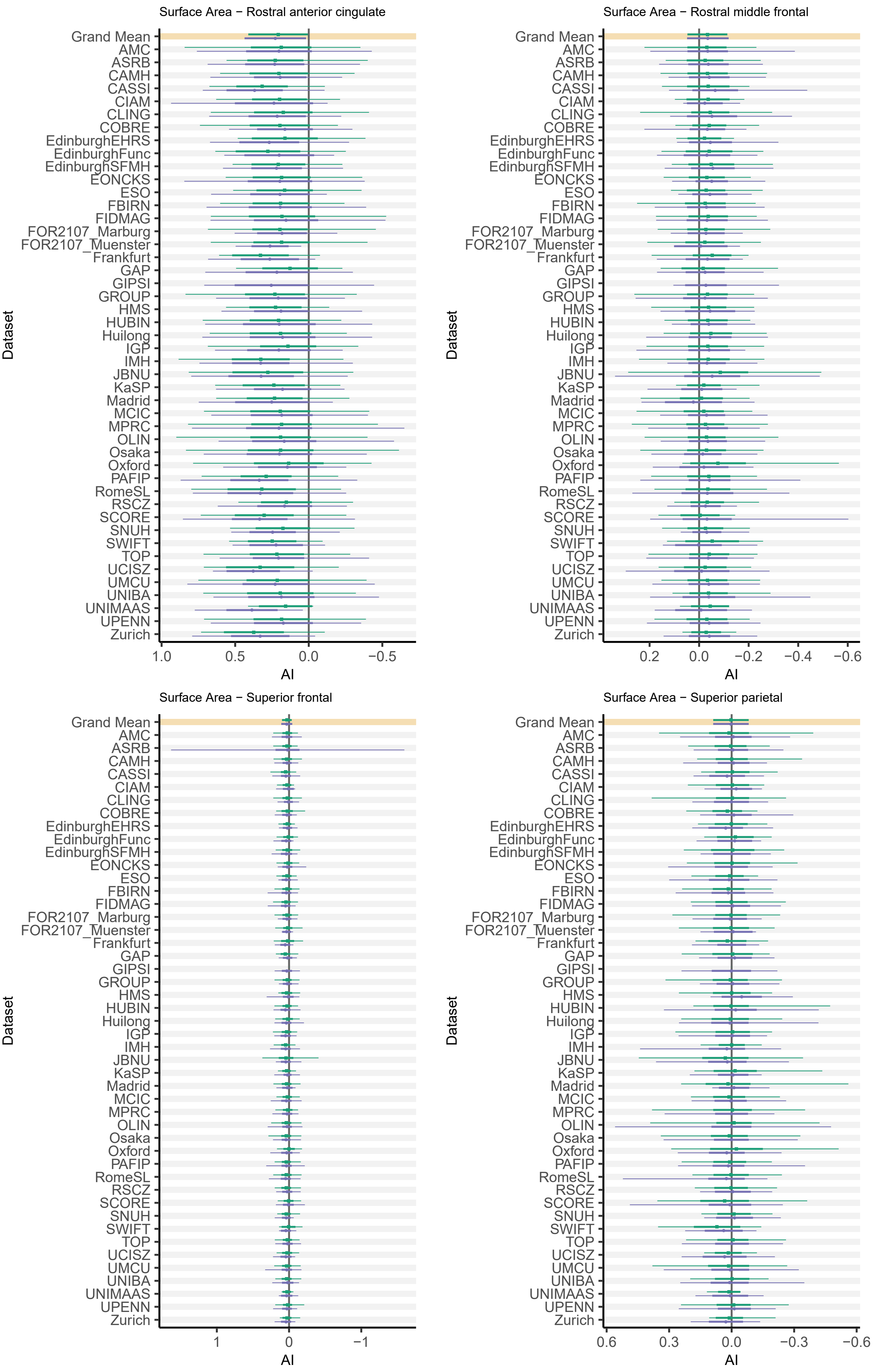
Surface Area − Rostral anterior cingulate
Surface Area − Rostral middle frontal
Surface Area − Superior frontal
Surface Area − Superior parietal
Dataset
Grand Mean
AMC
ASRB
CAMH
CASSI
CIAM
CLING
COBRE
EdinburghEHRS
EdinburghFunc
EdinburghSFMH
EONCKS
ESO
FBIRN
FIDMAG
FOR2107_Marburg
FOR2107_Muenster
Frankfurt
GAP
GIPSI
GROUP
HMS
HUBIN
Huilong
IGP
IMH
JBNU
KaSP
Madrid
MCIC
MPRC
OLIN
Osaka
Oxford
PAFIP
RomeSL
RSCZ
SCORE
SNUH
SWIFT
TOP
UCISZ
UMCU
UNIBA
UNIMAAS
UPENN
Zurich
1.0 0.5 0.0 −0.5
0.2 0.0 −0.2 −0.4 −0.6
1 0 −1
0.6 0.3 0.0 −0.3 −0.6
AI

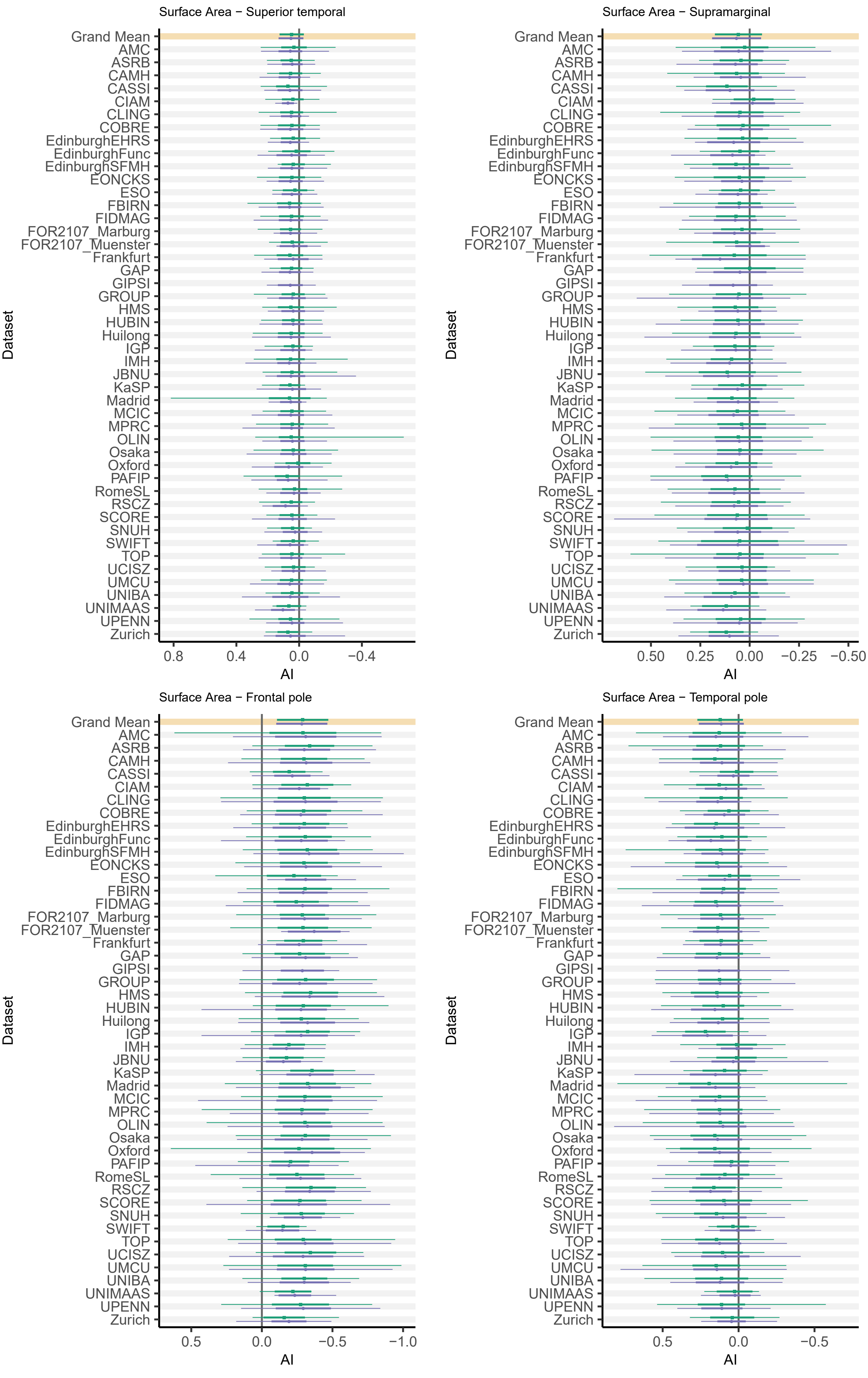
Surface Area − Superior temporal
Surface Area − Supramarginal
Surface Area − Frontal pole
Surface Area − Temporal pole
Dataset
Grand Mean
AMC
ASRB
CAMH
CASSI
CIAM
CLING
COBRE
EdinburghEHRS
EdinburghFunc
EdinburghSFMH
EONCKS
ESO
FBIRN
FIDMAG
FOR2107_Marburg
FOR2107_Muenster
Frankfurt
GAP
GIPSI
GROUP
HMS
HUBIN
Huilong
IGP
IMH
JBNU
KaSP
Madrid
MCIC
MPRC
OLIN
Osaka
Oxford
PAFIP
RomeSL
RSCZ
SCORE
SNUH
SWIFT
TOP
UCISZ
UMCU
UNIBA
UNIMAAS
UPENN
Zurich
0.8 0.4 0.0 −0.4
0.50 0.25 0.00 −0.25 −0.50
0.5 0.0 −0.5 −1.0
0.5 0.0 −0.5
AI

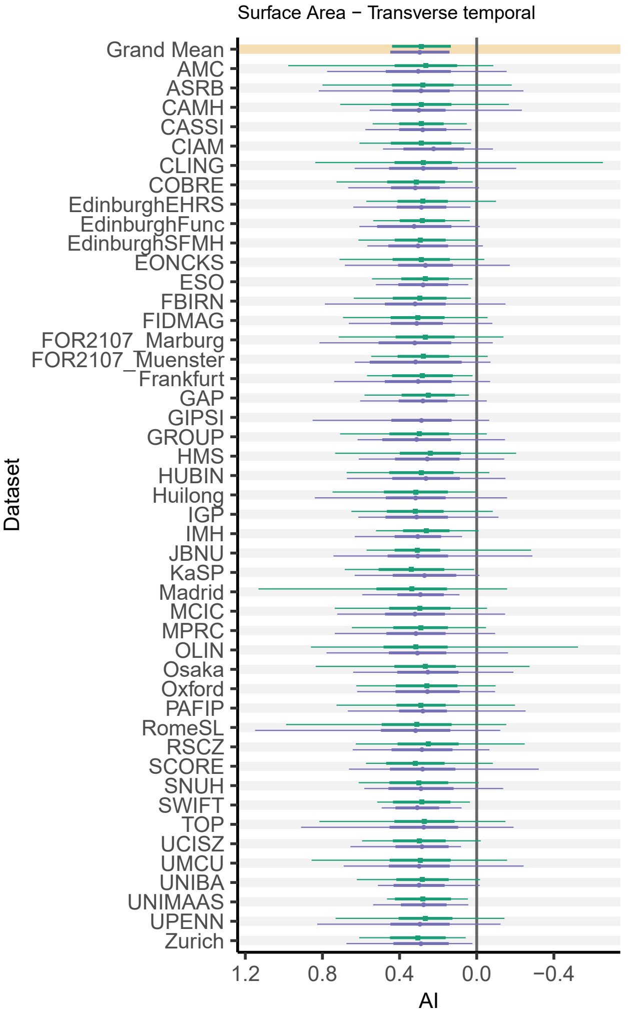
Surface Area − Transverse temporal
Dataset
Grand Mean
AMC
ASRB
CAMH
CASSI
CIAM
CLING
COBRE
EdinburghEHRS
EdinburghFunc
EdinburghSFMH
EONCKS
ESO
FBIRN
FIDMAG
FOR2107_Marburg
FOR2107_Muenster
Frankfurt
GAP
GIPSI
GROUP
HMS
HUBIN
Huilong
IGP
IMH
JBNU
KaSP
Madrid
MCIC
MPRC
OLIN
Osaka
Oxford
PAFIP
RomeSL
RSCZ
SCORE
SNUH
SWIFT
TOP
UCISZ
UMCU
UNIBA
UNIMAAS
UPENN
Zurich
1.2 0.8 0.4 0.0 −0.4
AI

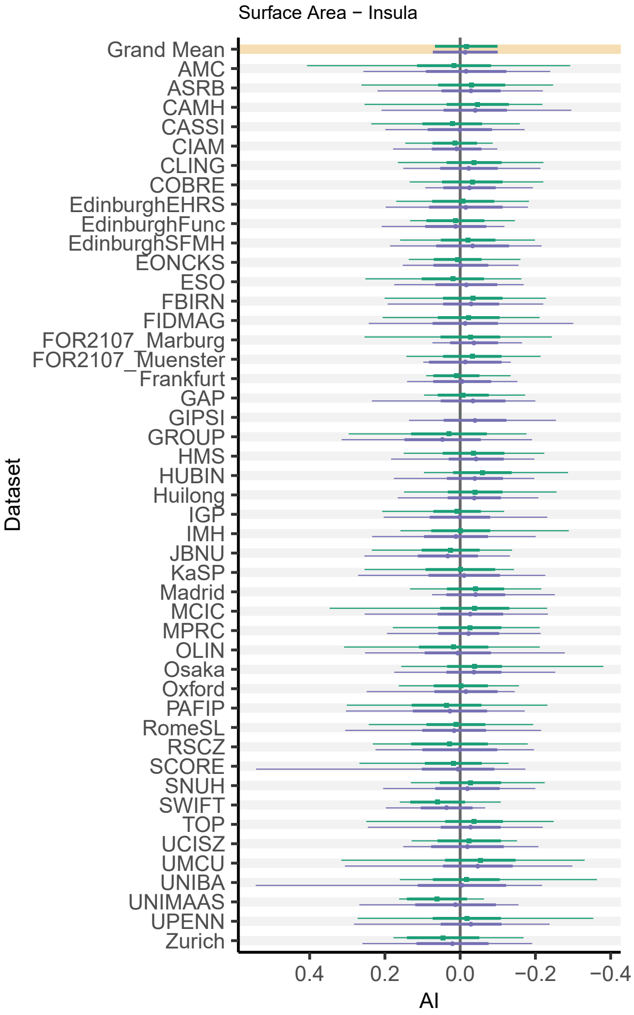
Surface Area − Insula
Dataset
Grand Mean
AMC
ASRB
CAMH
CASSI
CIAM
CLING
COBRE
EdinburghEHRS
EdinburghFunc
EdinburghSFMH
EONCKS
ESO
FBIRN
FIDMAG
FOR2107_Marburg
FOR2107_Muenster
Frankfurt
GAP
GIPSI
GROUP
HMS
HUBIN
Huilong
IGP
IMH
JBNU
KaSP
Madrid
MCIC
MPRC
OLIN
Osaka
Oxford
PAFIP
RomeSL
RSCZ
SCORE
SNUH
SWIFT
TOP
UCISZ
UMCU
UNIBA
UNIMAAS
UPENN
Zurich
0.4 0.2 0.0 −0.2 −0.4
AI

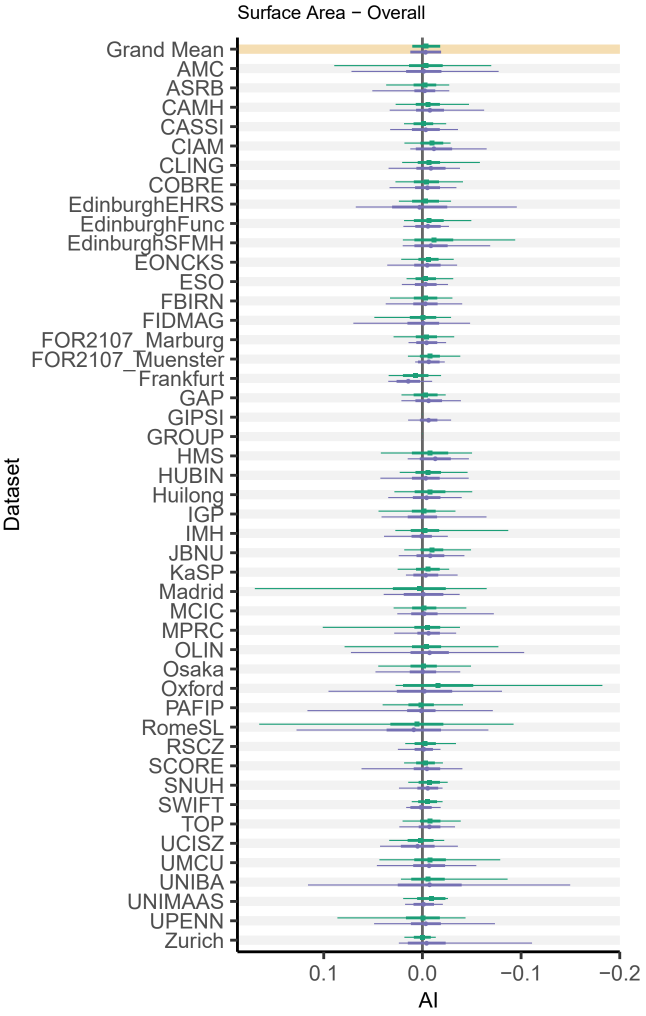
Surface Area − Overall
Dataset
Grand Mean
AMC
ASRB
CAMH
CASSI
CIAM
CLING
COBRE
EdinburghEHRS
EdinburghFunc
EdinburghSFMH
EONCKS
ESO
FBIRN
FIDMAG
FOR2107_Marburg
FOR2107_Muenster
Frankfurt
GAP
GIPSI
GROUP
HMS
HUBIN
Huilong
IGP
IMH
JBNU
KaSP
Madrid
MCIC
MPRC
OLIN
Osaka
Oxford
PAFIP
RomeSL
RSCZ
SCORE
SNUH
SWIFT
TOP
UCISZ
UMCU
UNIBA
UNIMAAS
UPENN
Zurich
0.1 0.0 −0.1 −0.2
AI

Fig. S4 (page 30-31). Overall and per-dataset average and range for subcortical volume asymmetries. For each subcortical volume asymmetry measure, the average in controls (green circles) and individuals affected with schizophrenia (purple squares) is shown. The top (highlighted) row contains the grand sample size-weighted mean and standard deviation (thick line segments). The other rows contain per-dataset averages, standard deviations and minimum and maximum values (indicated with thin line segments).

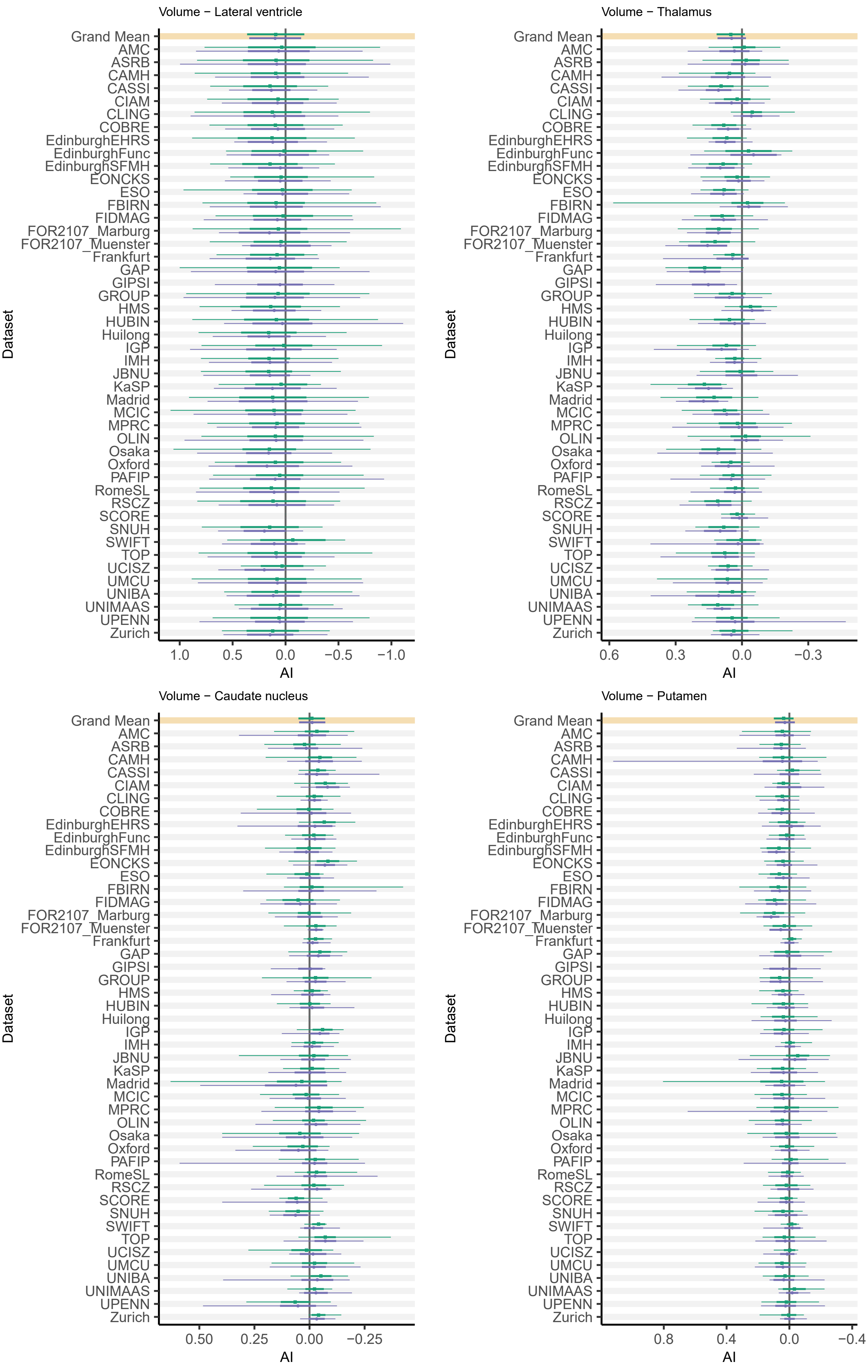
Volume − Lateral ventricle
Volume − Thalamus
Volume − Caudate nucleus
Volume − Putamen
Dataset
AI
Grand Mean
AMC
ASRB
CAMH
CASSI
CIAM
CLING
COBRE
EdinburghEHRS
EdinburghFunc
EdinburghSFMH
EONCKS
ESO
FBIRN
FIDMAG
FOR2107_Marburg
FOR2107_Muenster
Frankfurt
GAP
GIPSI
GROUP
HMS
HUBIN
Huilong
IGP
IMH
JBNU
KaSP
Madrid
MCIC
MPRC
OLIN
Osaka
Oxford
PAFIP
RomeSL
RSCZ
SCORE
SNUH
SWIFT
TOP
UCISZ
UMCU
UNIBA
UNIMAAS
UPENN
Zurich
1.0
0.5
0.0
−0.5
−1.0
0.6
0.3
0.0
−0.3
0.50
0.25
0.00
−0.25
0.8
0.4
0.0
−0.4

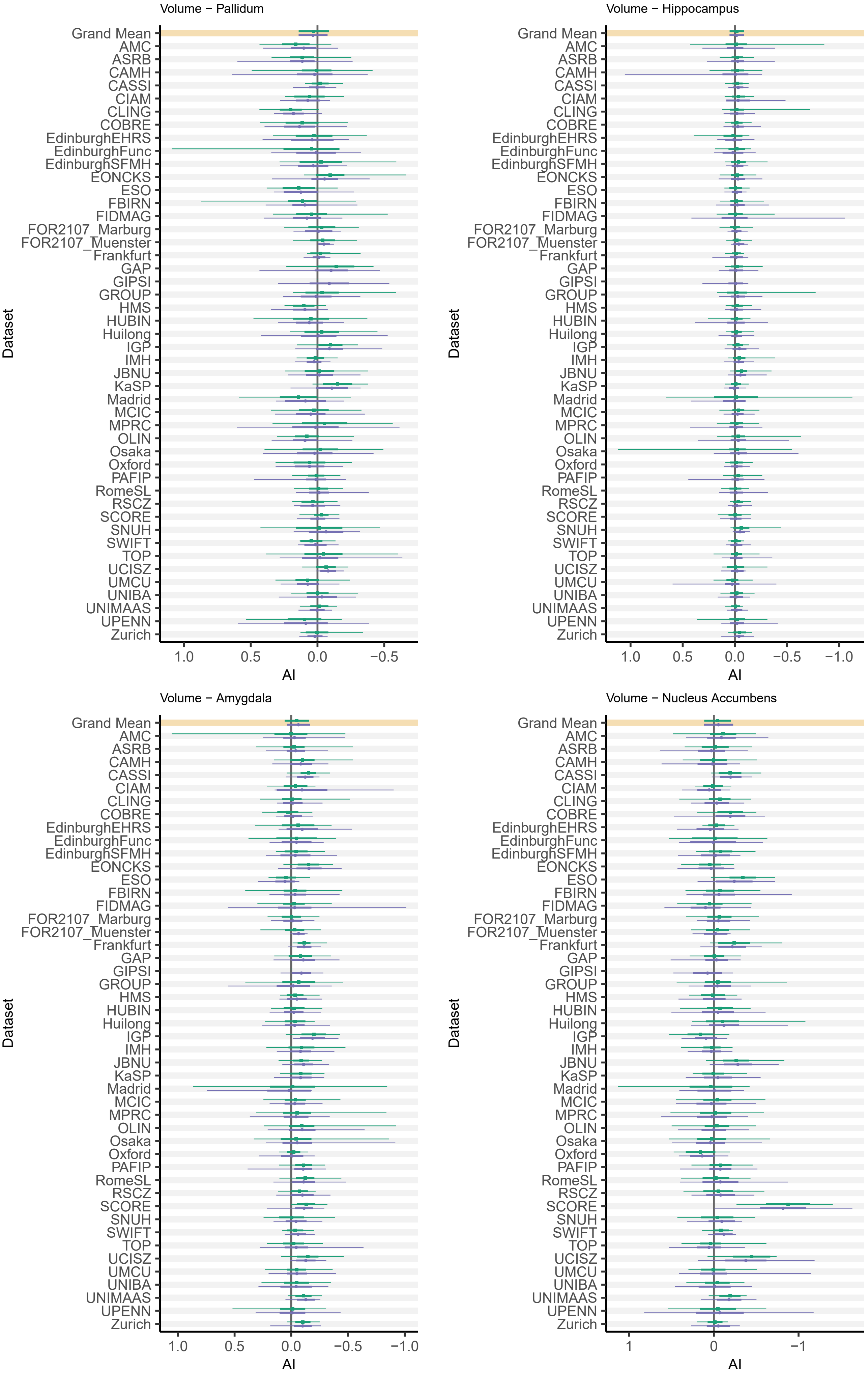
Volume − Pallidum
Volume − Hippocampus
Volume − Amygdala
Volume − Nucleus Accumbens
Dataset
Grand Mean
AMC
ASRB
CAMH
CASSI
CIAM
CLING
COBRE
EdinburghEHRS
EdinburghFunc
EdinburghSFMH
EONCKS
ESO
FBIRN
FIDMAG
FOR2107_Marburg
FOR2107_Muenster
Frankfurt
GAP
GIPSI
GROUP
HMS
HUBIN
Huilong
IGP
IMH
JBNU
KaSP
Madrid
MCIC
MPRC
OLIN
Osaka
Oxford
PAFIP
RomeSL
RSCZ
SCORE
SNUH
SWIFT
TOP
UCISZ
UMCU
UNIBA
UNIMAAS
UPENN
Zurich
1.0 0.5 0.0 −0.5
1.0 0.5 0.0 −0.5 −1.0
1 0 −1
AI

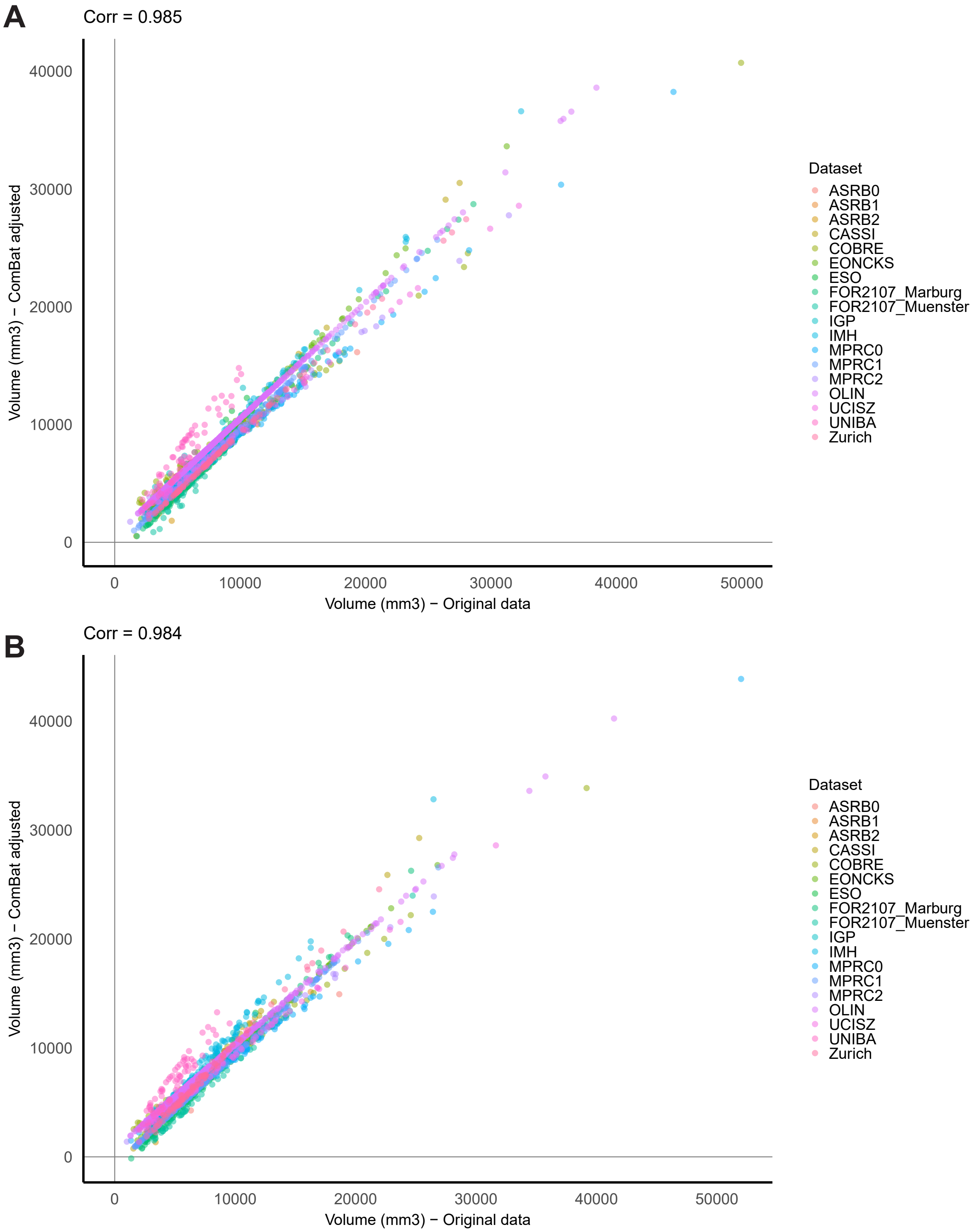

Fig. S5. Original and ComBat adjusted lateral ventricle volumes. Original lateral ventricle volumes (x-axis) versus ComBat (10) adjusted measurements (y-axis) for the left **(A)** and right **(B)** hemispheres are shown for individuals across 14 datasets (color-coded, two datasets – ASRB and MPRC – are split because of multiple scanners). Correlations (Corr) between original and ComBat adjusted measurements are shown above each figure. One subject from the FOR2107 Marburg dataset had a slightly negative adjusted right lateral ventricle volume and was therefore excluded.

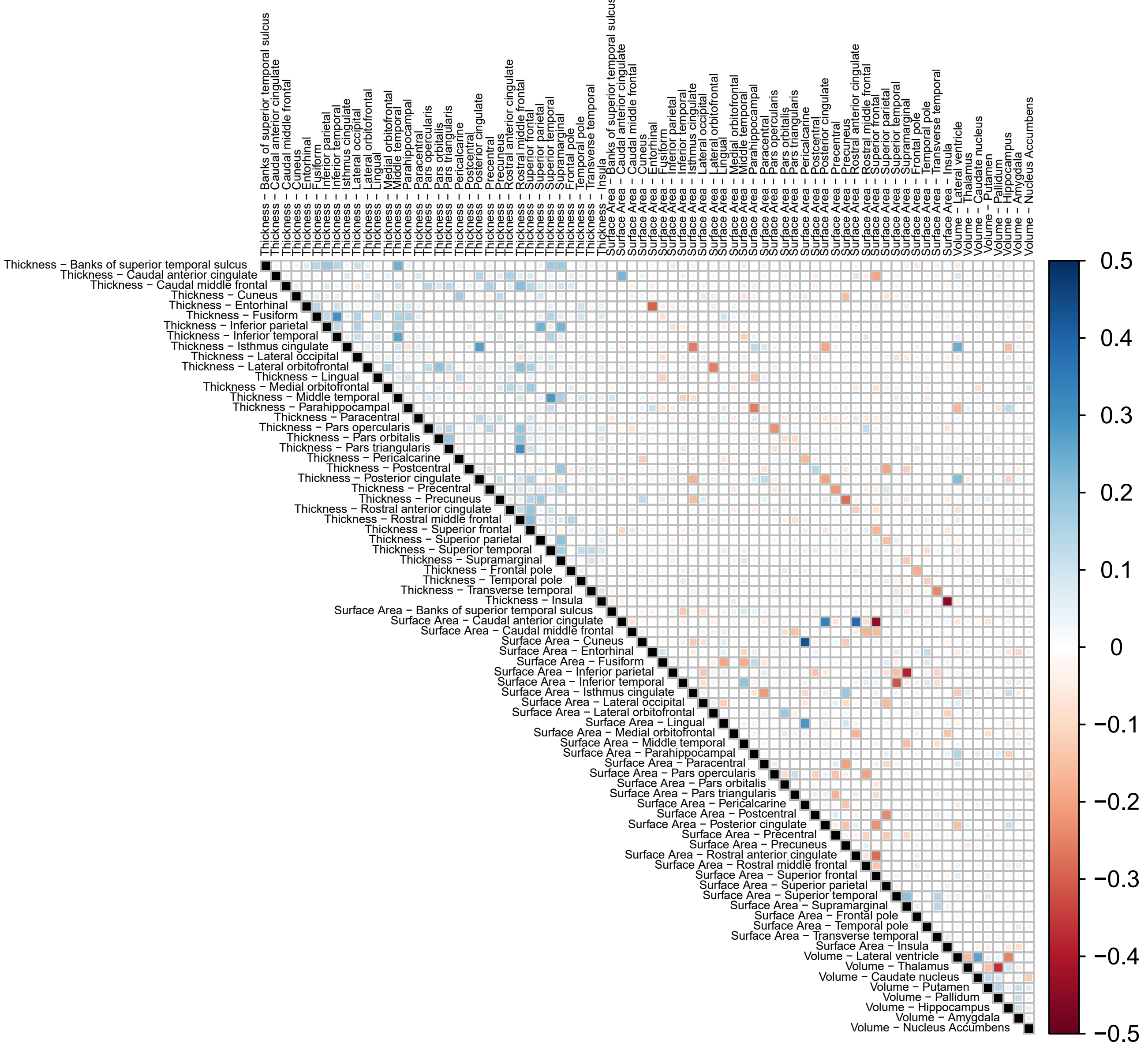

Fig. S6. Correlations between structural asymmetries in the 14 datasets available for multivariate analysis (i.e. where individual-level data were available to the central analysis team). The correlations between AIs are shown at the intersections of rows and columns. Positive correlations are shown in blue shades, negative correlations are shown in red shades. Figure generated using the *corrplot* package in R (11).

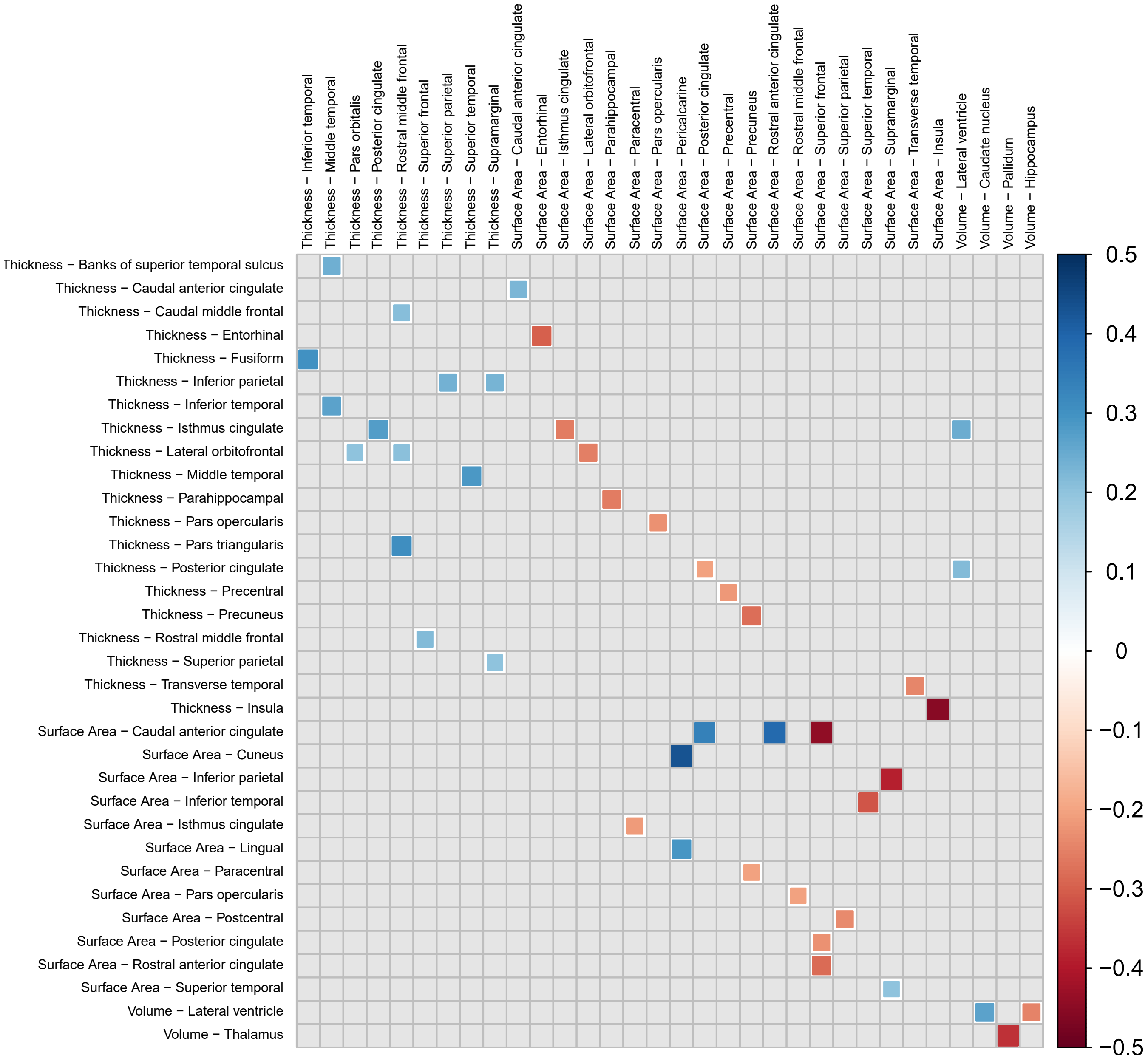

Fig. S7. Correlations > 0.2 between structural asymmetries in the 14 datasets available for multivariate analysis (i.e. where individual-level data were available to the central analysis team). The correlations between AIs are shown at the intersections of rows and columns. Only correlations > 0.2 are shown and structural asymmetries not having any such large correlations are excluded from the matrix (i.e. this figure shows a subset of the same correlation matrix as in Fig. S6, to aid in visualization of the larger correlations only). Positive correlations are shown in blue shades, negative correlations are shown in red shades. Figure generated using the *corrplot* package in R (11).

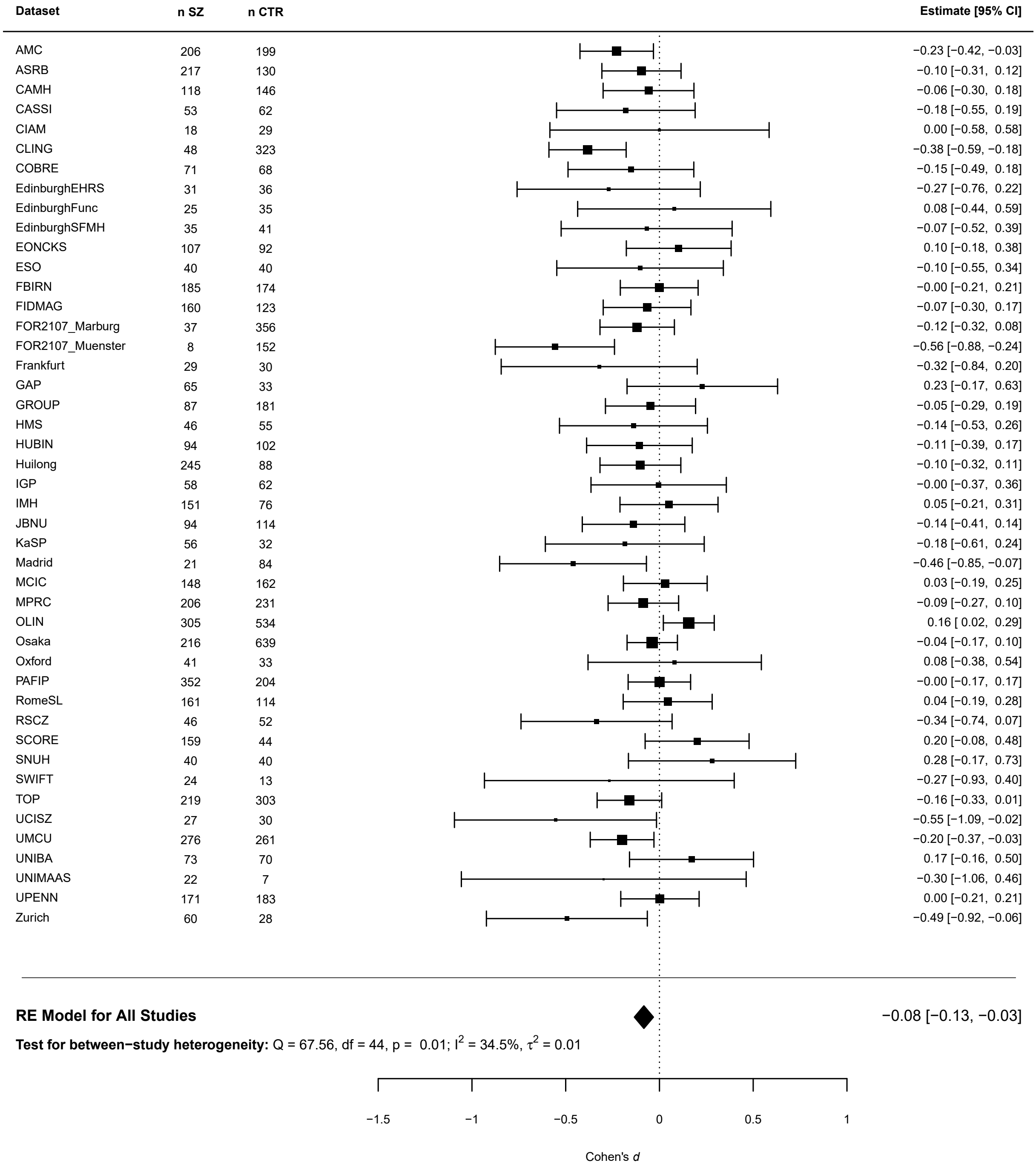

Fig. S8. Forest plot for random effects meta-analysis of rostral anterior cingulate thickness asymmetry differences between schizophrenia individuals and unaffected controls. Shown are the per-dataset effect sizes, including confidence intervals, of affected individuals (n SZ) and unaffected controls (n CTR). Cohen's *d* dot sizes represent relative dataset sizes. The meta-analyzed effect sizes across all studies is shown (black diamond), as well as between-dataset heterogeneity statistics (Cochran's Q test statistics).

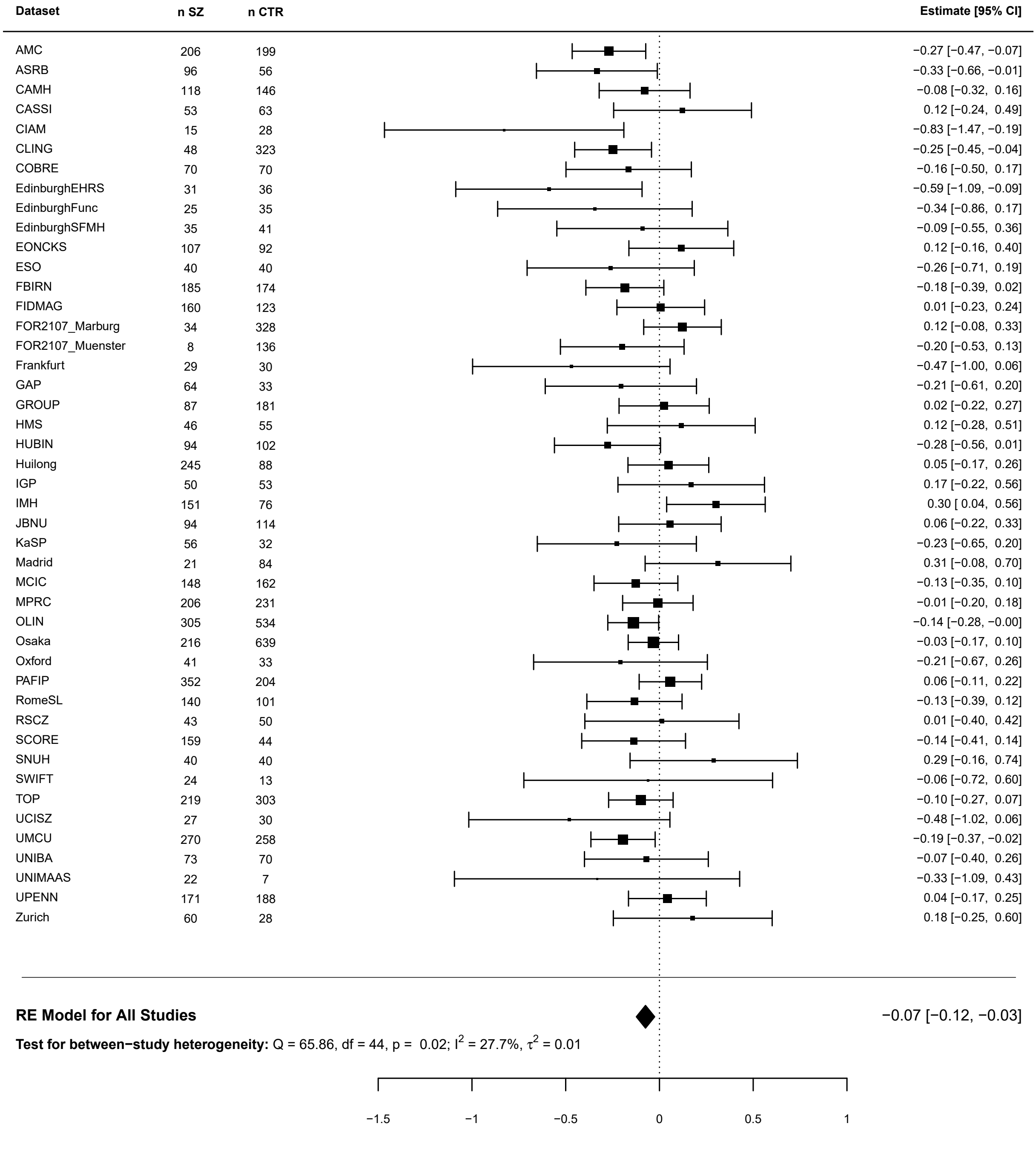

Fig. S9. Forest plot for random effects meta-analysis of middle temporal gyrus thickness asymmetry differences between schizophrenia individuals and unaffected controls. Shown are the per-dataset effect sizes, including confidence intervals, of affected individuals (n SZ) and unaffected controls (n CTR). Cohen's *d* dot sizes represent relative dataset sizes. The meta-analyzed effect sizes across all studies is shown (black diamond), as well as between-dataset heterogeneity statistics (Cochran's Q test statistics).

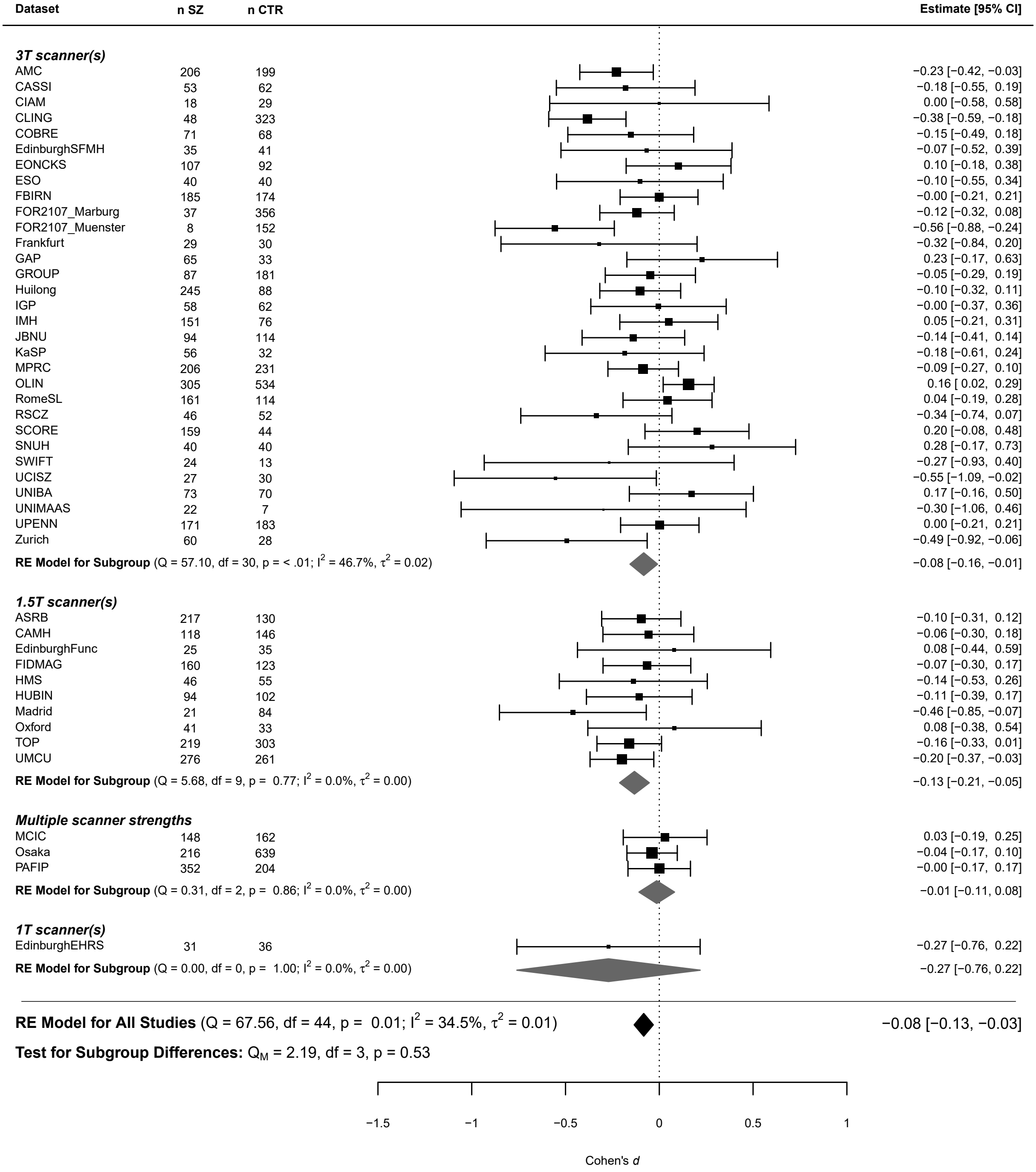

Fig. S10. Forest plot grouped by scanner field strength, for random effects meta-analysis of rostral anterior cingulate thickness asymmetry differences between individuals with schizophrenia and unaffected controls. Shown are the per-dataset effect sizes, including confidence intervals, and the numbers of affected individuals (n SZ) and unaffected controls (n CTR). Cohen's *d* dot sizes represent relative dataset sizes. Meta-analyzed effect sizes are shown at a group level (gray diamonds) and across all studies (black diamonds), as well as between-dataset heterogeneity statistics (Cochran's Q test statistics). Scanner field strength group differences were tested using an omnibus test for heterogeneity. Groups are ordered from largest to smallest.

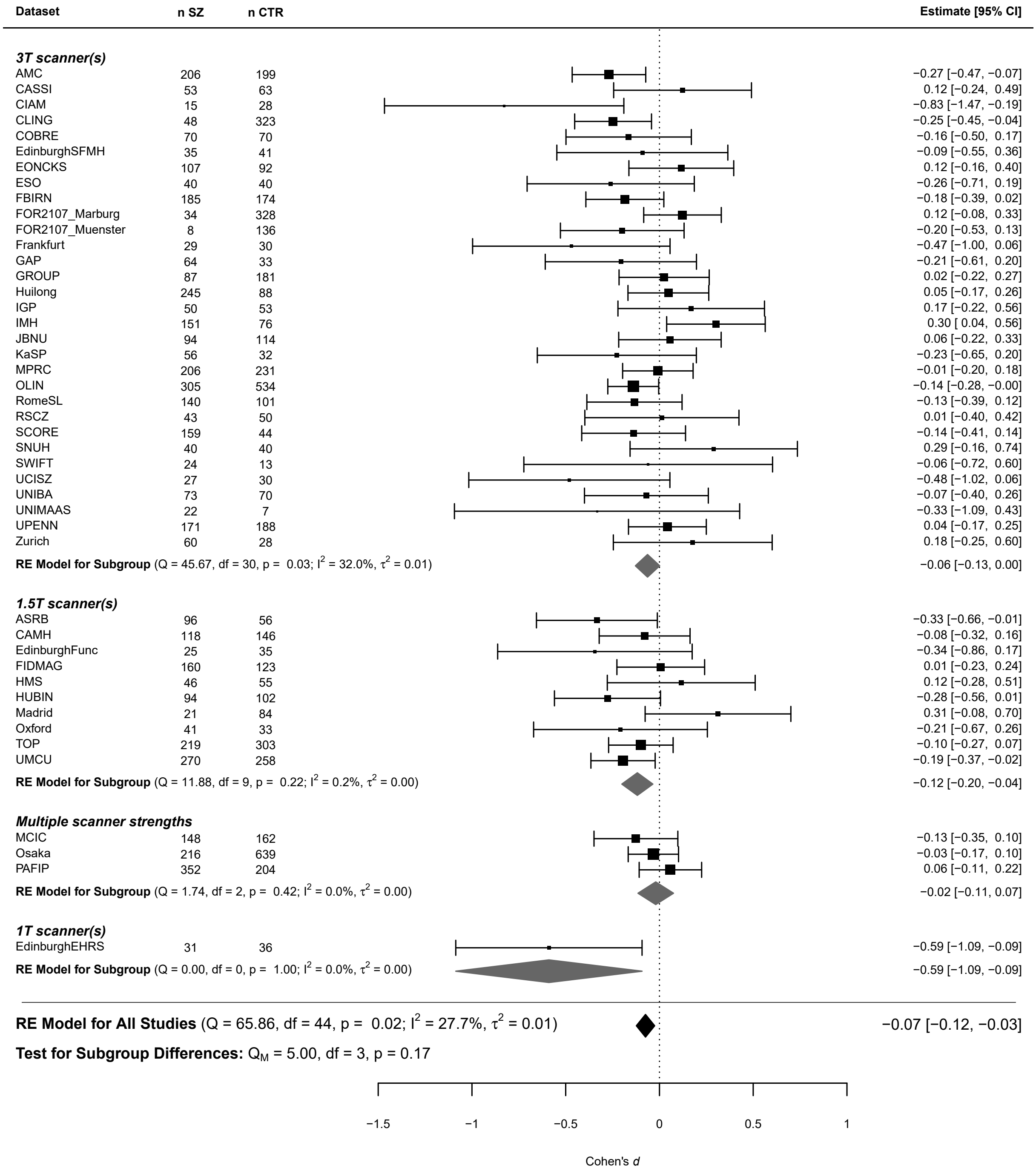

Fig. S11. Forest plot grouped by scanner field strength, for random effects meta-analysis of middle temporal gyrus thickness asymmetry differences between schizophrenia individuals and unaffected controls. Shown are the per-dataset effect sizes, including confidence intervals, and the numbers of affected individuals (n SZ) and unaffected controls (n CTR). Cohen's *d* dot sizes represent relative dataset sizes. Meta-analyzed effect sizes are shown at a group level (gray diamonds) and across all studies (black diamonds), as well as between-dataset heterogeneity statistics (Cochran's Q test statistics). Scanner field strength group differences were tested using an omnibus test for heterogeneity. Groups are ordered from largest to smallest.

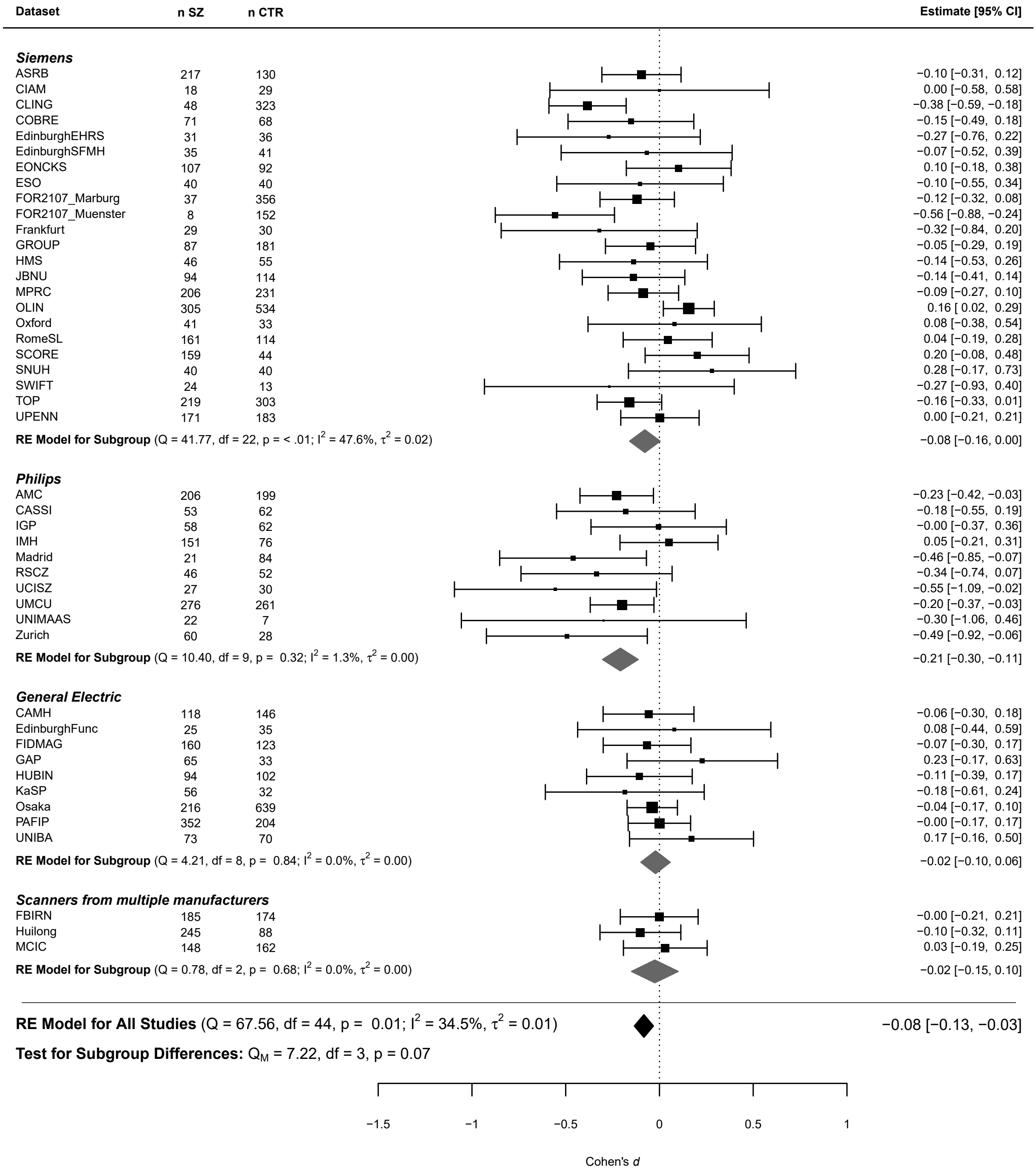

Fig. S12. Forest plot grouped by scanner manufacturer, for random effects meta-analysis of rostral anterior cingulate thickness asymmetry differences between schizophrenia individuals and unaffected controls. Shown are the per-dataset effect sizes, including confidence intervals, and the numbers of affected individuals (n SZ) and unaffected controls (n CTR). Cohen's *d* dot sizes represent relative dataset sizes. Meta-analyzed effect sizes are shown at a group level (gray diamonds) and across all studies (black diamonds), as well as between-dataset heterogeneity statistics (Cochran's Q test statistics). Scanner manufacturer group differences were tested using an omnibus test for heterogeneity. Groups are ordered from largest to smallest.

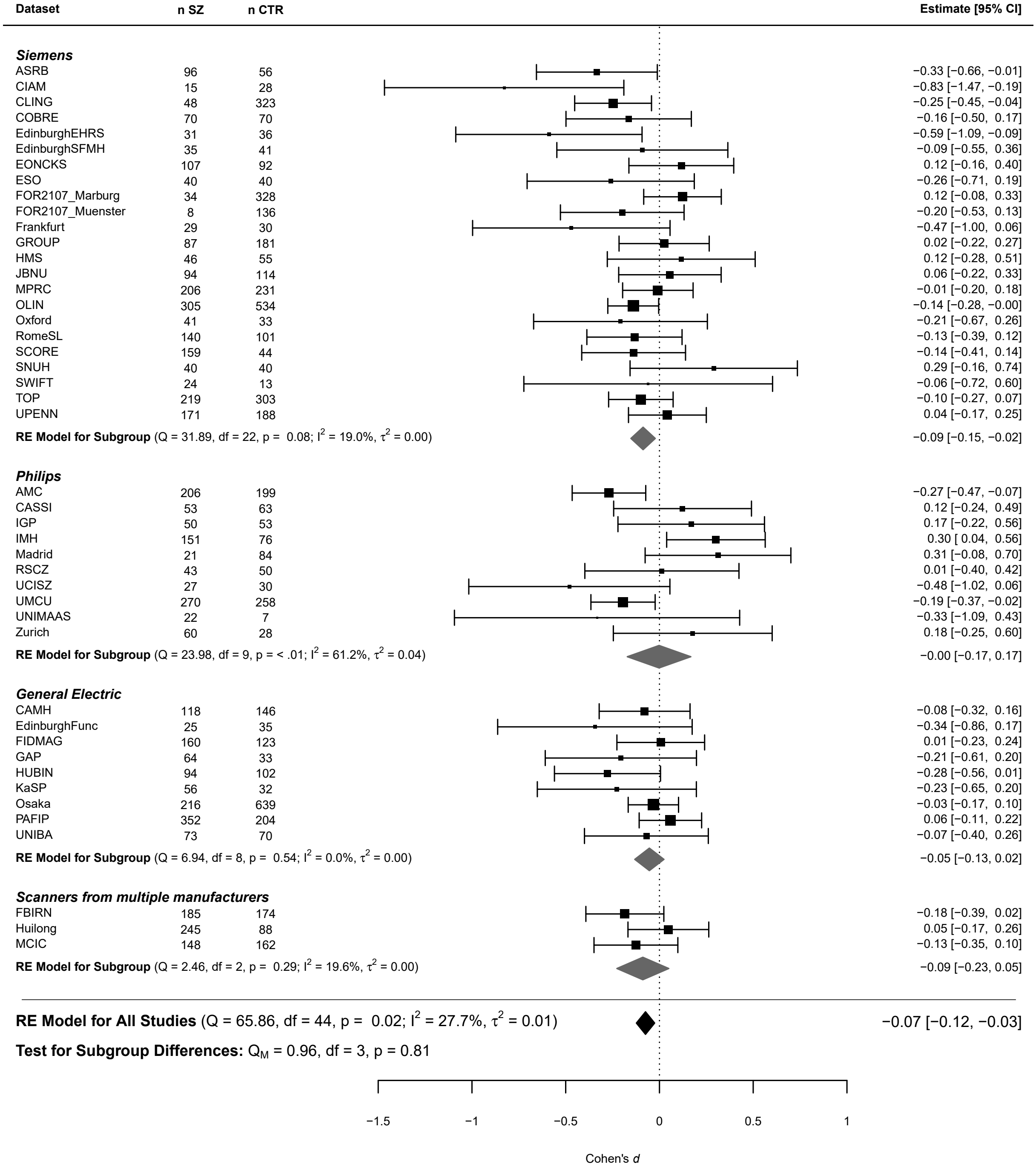

Fig. S13. Forest plot grouped by scanner manufacturer, for random effects meta-analysis of middle temporal gyrus asymmetry differences between schizophrenia individuals and unaffected controls. Shown are the per-dataset effect sizes, including confidence intervals, and the numbers of affected individuals (n SZ) and unaffected controls (n CTR). Cohen's *d* dot sizes represent relative dataset sizes. Meta-analyzed effect sizes are shown at a group level (gray diamonds) and across all studies (black diamonds), as well as between-dataset heterogeneity statistics (Cochran's Q test statistics). Scanner manufacturer group differences were tested using an omnibus test for heterogeneity. Groups are ordered from largest to smallest.

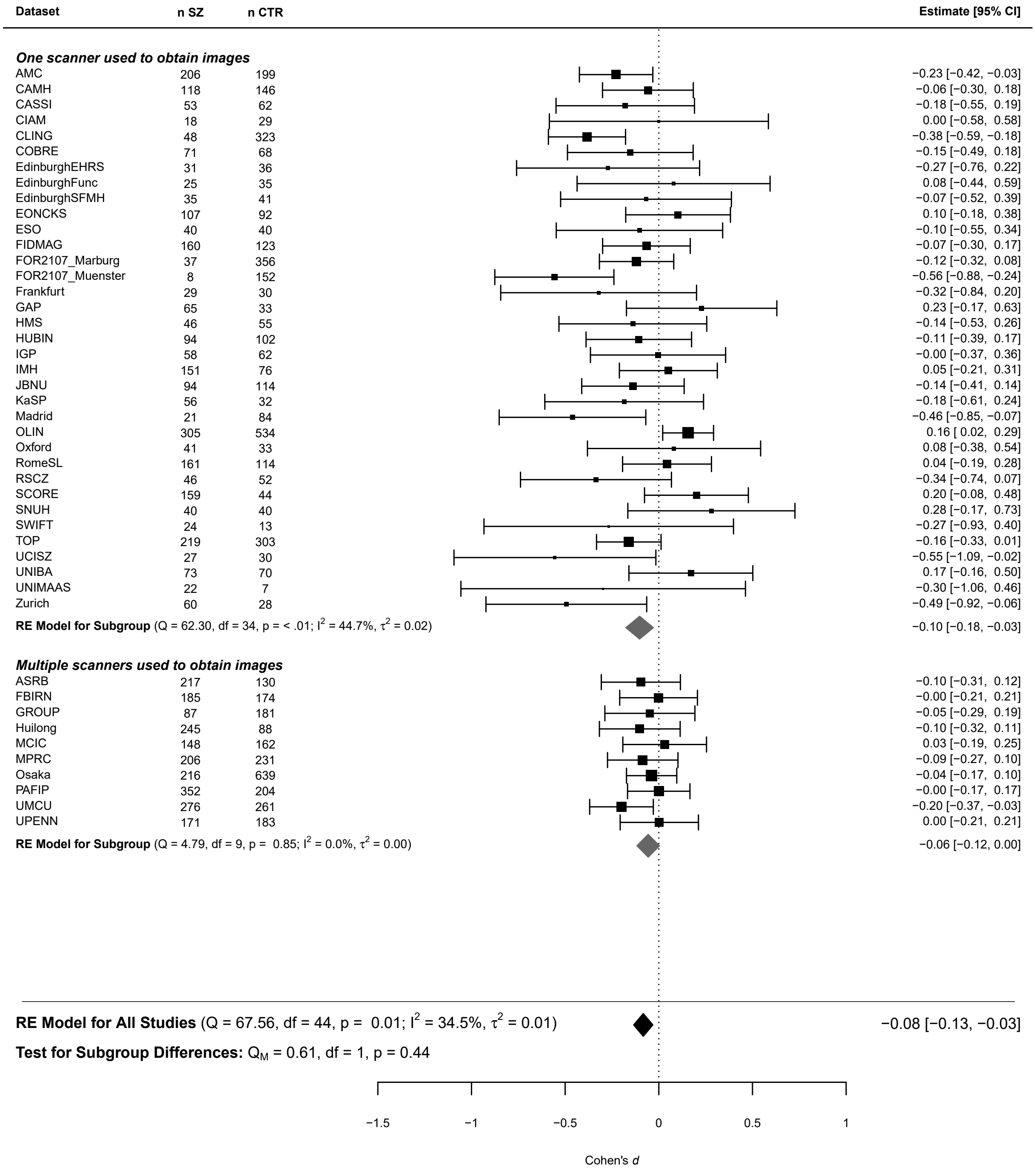

Fig. S14. Forest plot where datasets are grouped by use of a single scanner versus multiple scanners, for random effects meta-analysis of rostral anterior cingulate thickness asymmetry differences between schizophrenia individuals and unaffected controls. Shown are the per-dataset effect sizes, including confidence intervals, and the numbers of affected individuals (n SZ) and unaffected controls (n CTR). Cohen's *d* dot sizes represent relative dataset sizes. Meta-analyzed effect sizes are shown at a group level (gray diamonds) and across all studies (black diamonds), as well as between-dataset heterogeneity statistics (Cochran's Q test statistics). Single scanner versus multiple scanner group differences were tested using an omnibus test for heterogeneity. Groups are ordered from largest to smallest.

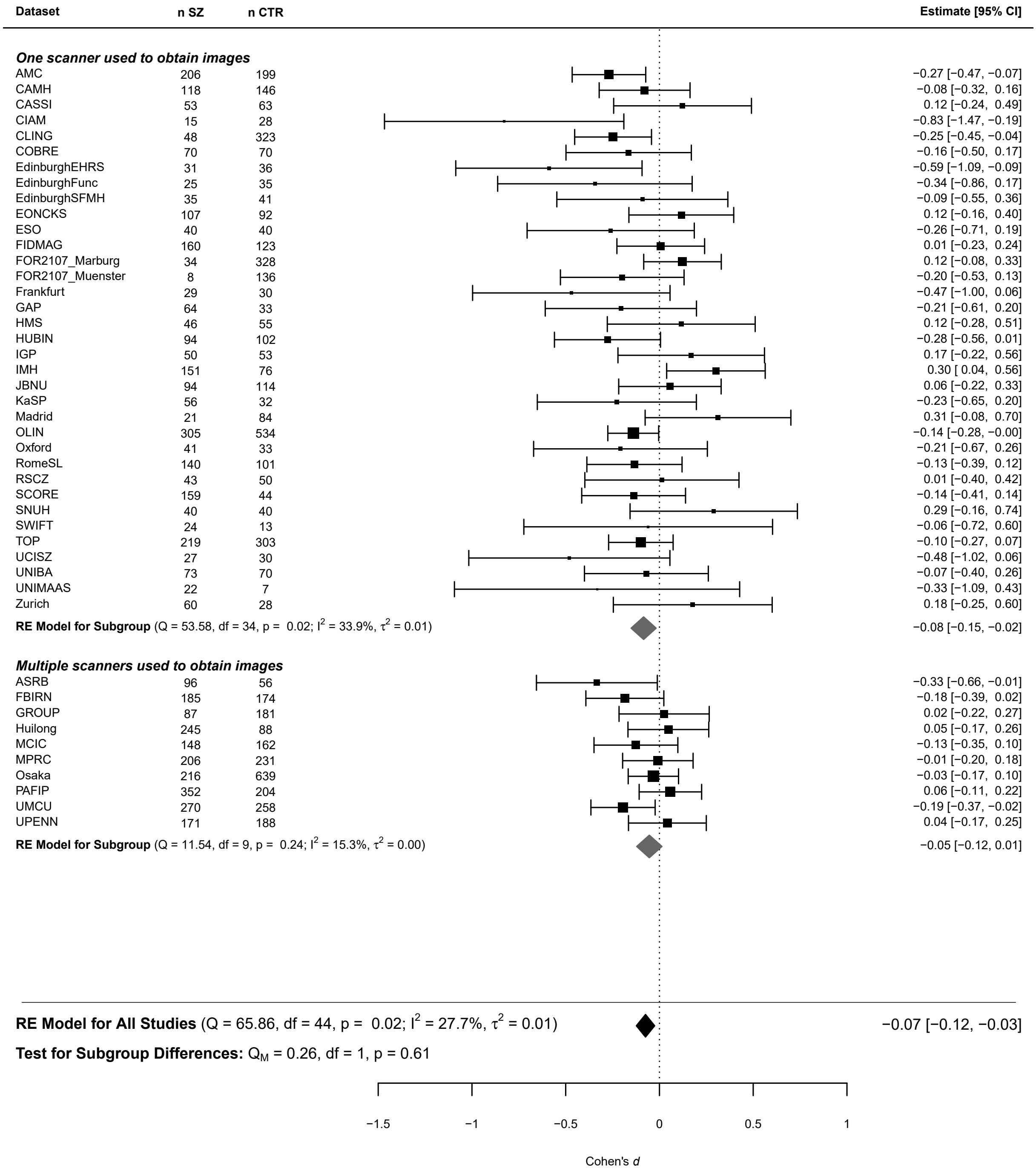

Fig. S15. Forest plot where datasets are grouped by the use of a single scanner versus multiple scanners, for random effects meta-analysis of middle temporal gyrus thickness asymmetry differences between schizophrenia individuals and unaffected controls. Shown are the per-dataset effect sizes, including confidence intervals, and the numbers of affected individuals (n SZ) and unaffected controls (n CTR). Cohen's *d* dot sizes represent relative dataset sizes. Meta-analyzed effect sizes are shown at a group level (gray diamonds) and across all studies (black diamonds), as well as between-dataset heterogeneity statistics (Cochran's Q test statistics). Single scanner versus multiple scanner group differences were tested using an omnibus test for heterogeneity. Groups are ordered from largest to smallest.

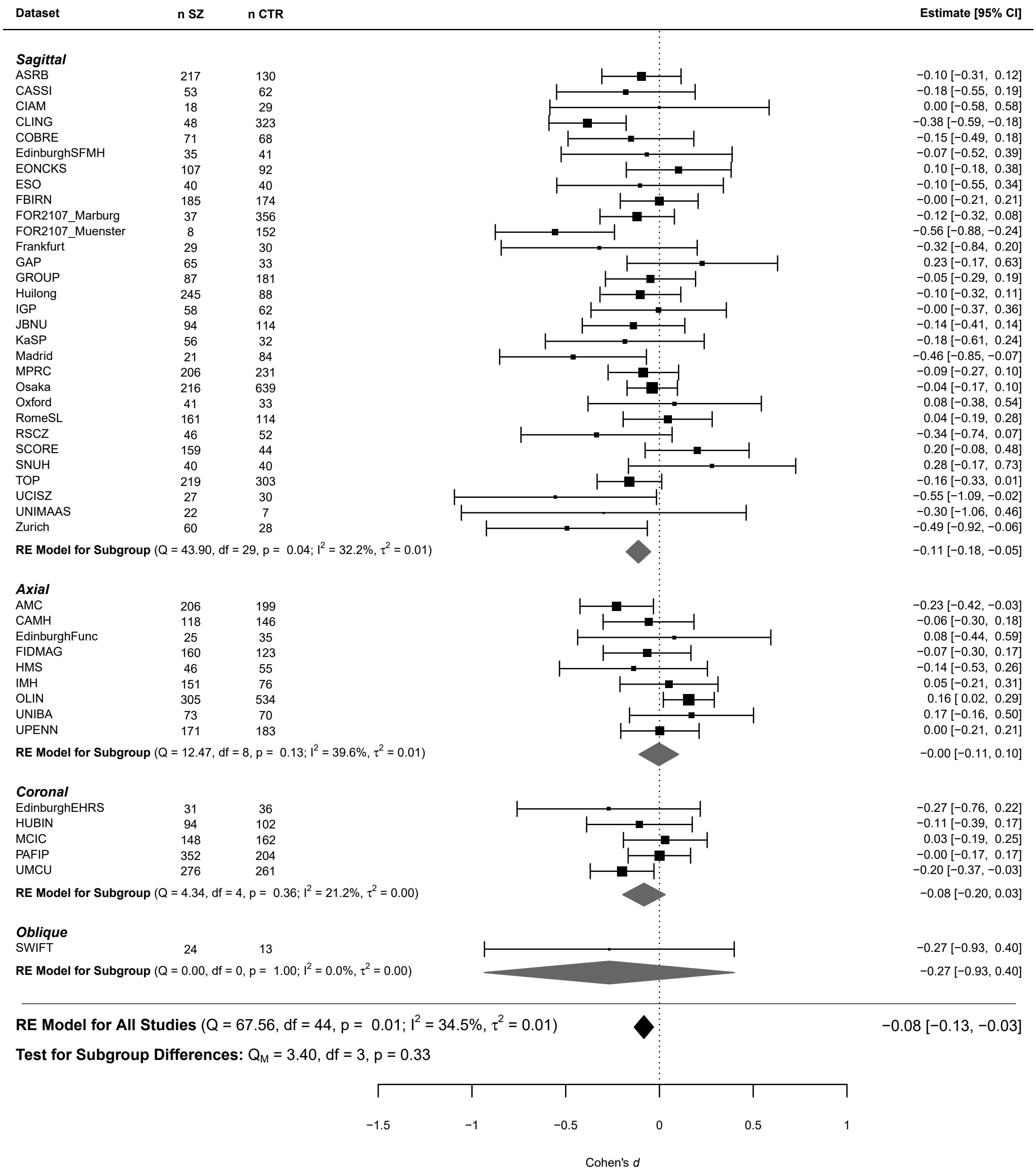

Fig. S16. Forest plot grouped by image slice orientation, for random effects meta-analysis of rostral anterior cingulate thickness asymmetry differences between schizophrenia individuals and unaffected controls. Shown are the per-dataset effect sizes, including confidence intervals, and the numbers of affected individuals (n SZ) and unaffected controls (n CTR). Cohen's *d* dot sizes represent relative dataset sizes. Meta-analyzed effect sizes are shown at a group level (gray diamonds) and across all studies (black diamonds), as well as between-dataset heterogeneity statistics (Cochran's Q test statistics). Image slice orientation group differences were tested using an omnibus test for heterogeneity. Groups are ordered from largest to smallest.

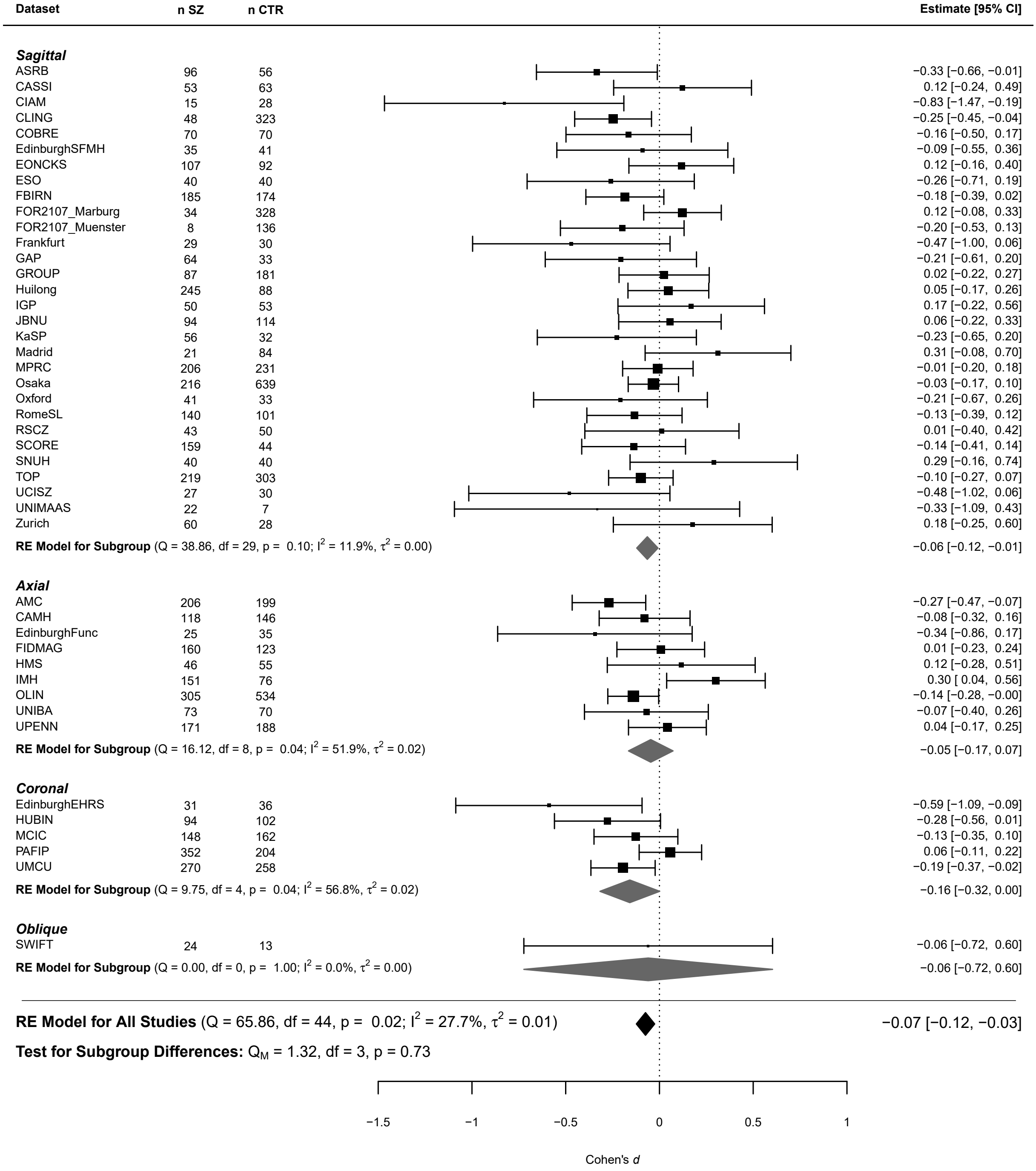

Fig. S17. Forest plot grouped by image slice orientation, for random effects meta-analysis of middle temporal gyrus thickness asymmetry differences between schizophrenia individuals and unaffected controls. Shown are the per-dataset effect sizes, including confidence intervals, and the numbers of affected individuals (n SZ) and unaffected controls (n CTR). Cohen's *d* dot sizes represent relative dataset sizes. Meta-analyzed effect sizes are shown at a group level (gray diamonds) and across all studies (black diamonds), as well as between-dataset heterogeneity statistics (Cochran's Q test statistics). Image slice orientation group differences were tested using an omnibus test for heterogeneity. Groups are ordered from largest to smallest.

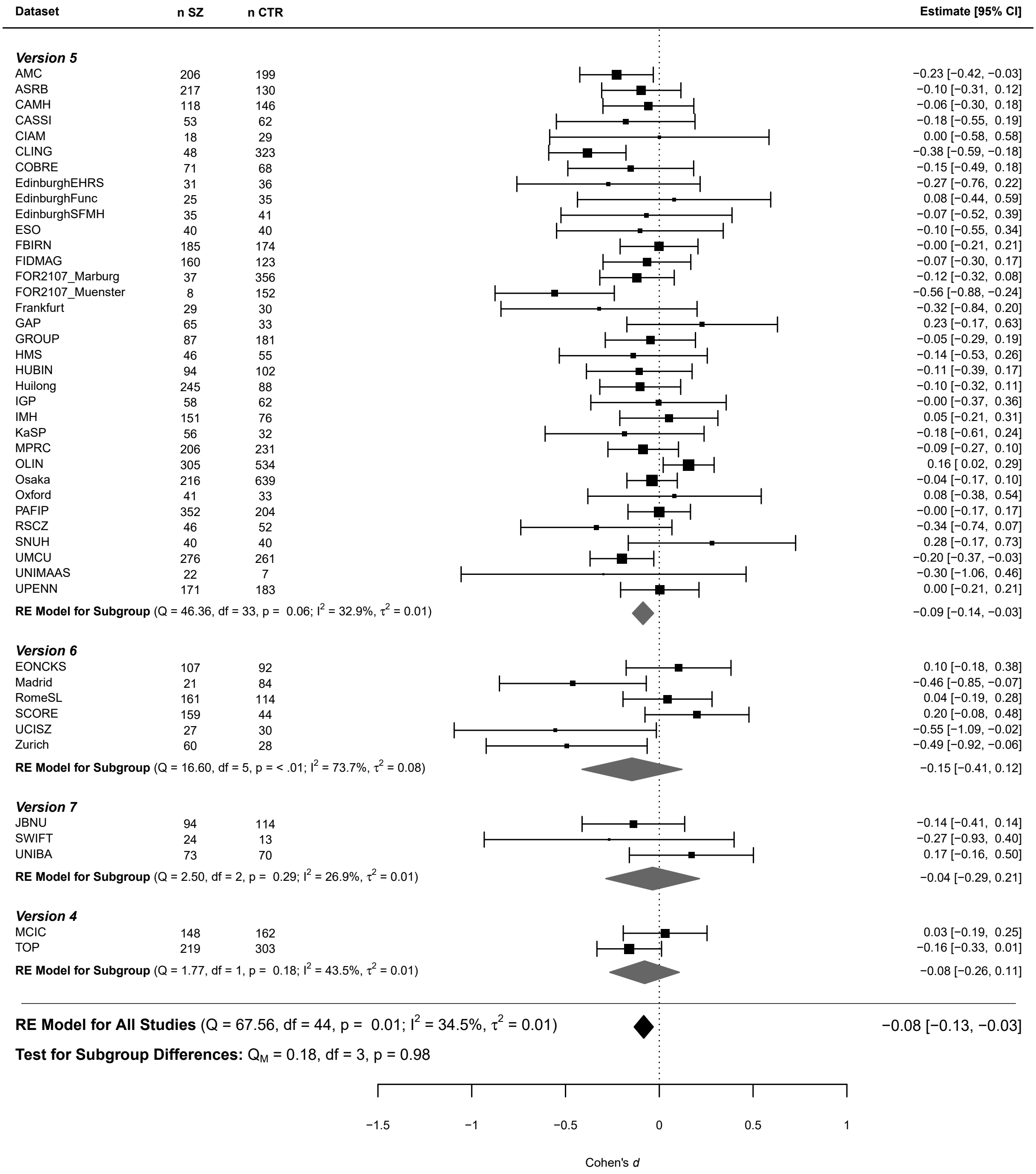

Fig. S18. Forest plot grouped by Freesurfer version, for random effects meta-analysis of rostral anterior cingulate thickness asymmetry differences between schizophrenia individuals and unaffected controls. Shown are the per-dataset effect sizes, including confidence intervals, and the numbers of affected individuals (n SZ) and unaffected controls (n CTR). Cohen's *d* dot sizes represent relative dataset sizes. Meta-analyzed effect sizes are shown at a group level (gray diamonds) and across all studies (black diamonds), as well as between-dataset heterogeneity statistics (Cochran's Q test statistics). Freesurfer version group differences were tested using an omnibus test for heterogeneity. Groups are ordered from largest to smallest.

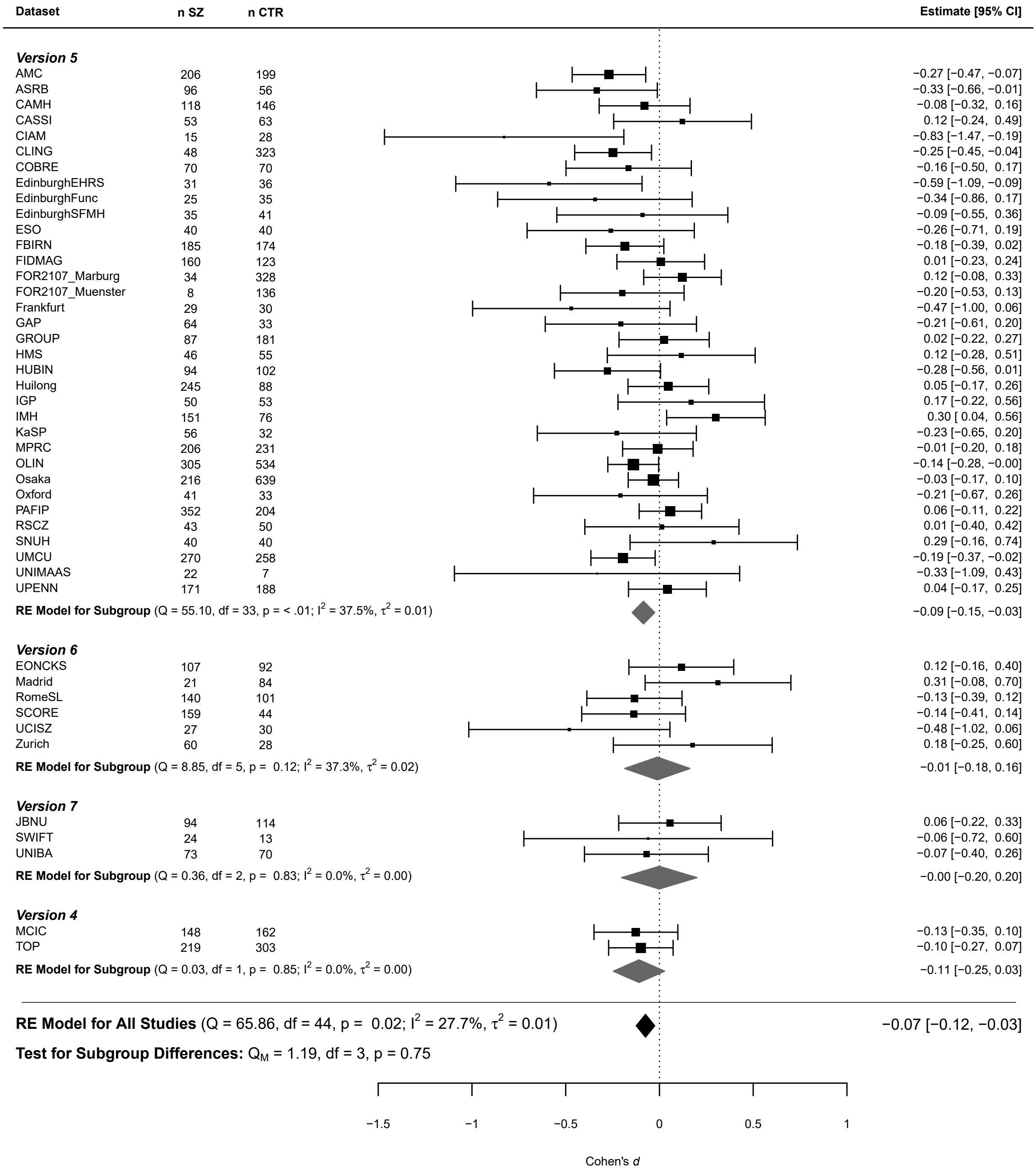

Fig. S19. Forest plot grouped by Freesurfer version, for random effects meta-analysis of middle temporal gyrus thickness asymmetry differences between schizophrenia individuals and unaffected controls. Shown are the per-dataset effect sizes, including confidence intervals, and the numbers of affected individuals (n SZ) and unaffected controls (n CTR). Cohen's *d* dot sizes represent relative dataset sizes. Meta-analyzed effect sizes are shown at a group level (gray diamonds) and across all studies (black diamonds), as well as between-dataset heterogeneity statistics (Cochran's Q test statistics). Freesurfer version group differences were tested using an omnibus test for heterogeneity. Groups are ordered from largest to smallest.

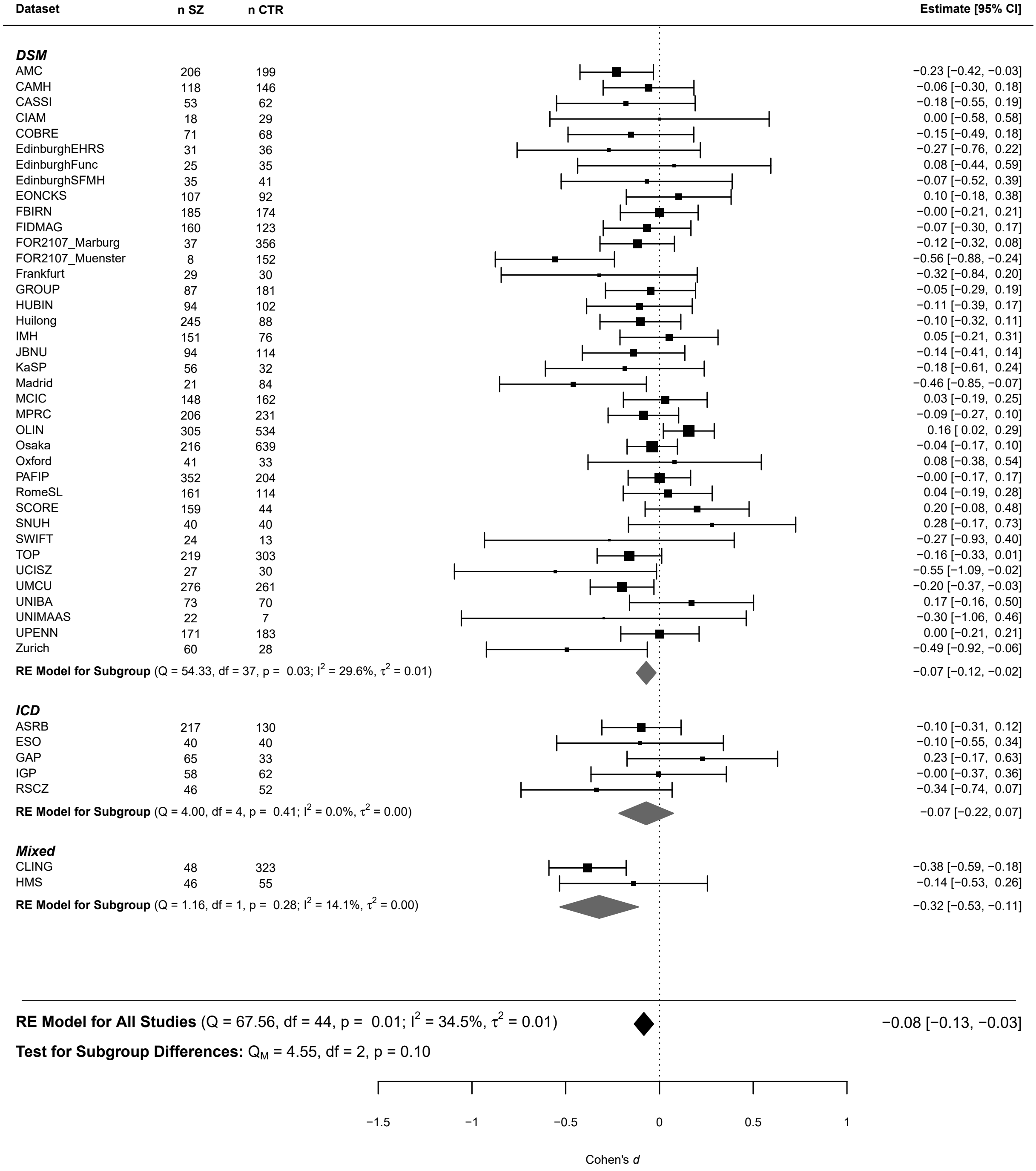

Fig. S20. Forest plot grouped by diagnostic tool, for random effects meta-analysis of rostral anterior cingulate thickness asymmetry differences between schizophrenia individuals and unaffected controls. Shown are the per-dataset effect sizes, including confidence intervals, and the numbers of affected individuals (n SZ) and unaffected controls (n CTR). Cohen's *d* dot sizes represent relative dataset sizes. Meta-analyzed effect sizes are shown at a group level (gray diamonds) and across all studies (black diamonds), as well as between-dataset heterogeneity statistics (Cochran's Q test statistics). Diagnostic tool group differences were tested using an omnibus test for heterogeneity. Groups are ordered from largest to smallest.

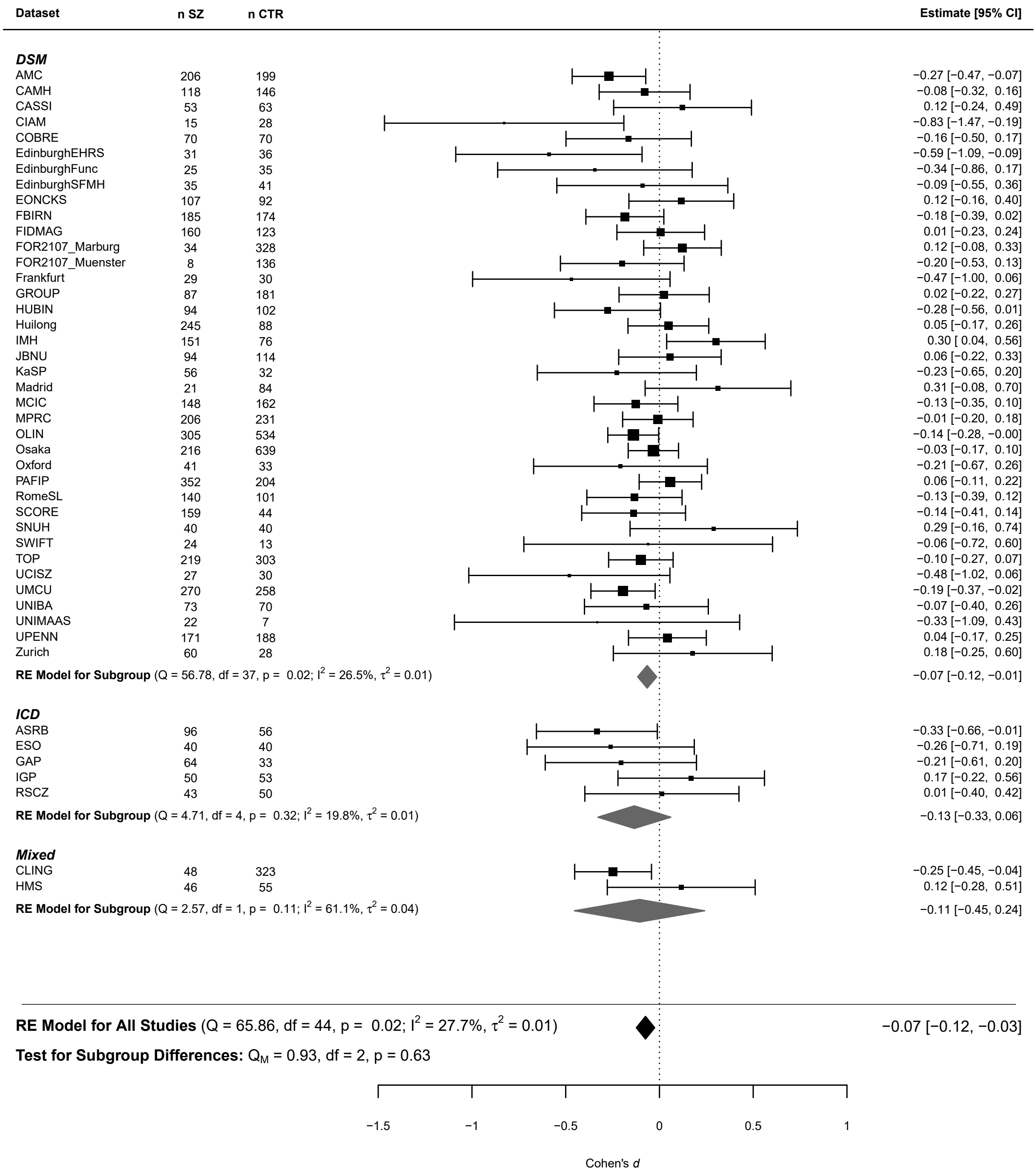

Fig. S21. Forest plot grouped by diagnostic tool, for random effects meta-analysis of middle temporal gyrus thickness asymmetry differences between schizophrenia individuals and unaffected controls. Shown are the per-dataset effect sizes, including confidence intervals, and the numbers of affected individuals (n SZ) and unaffected controls (n CTR). Cohen's *d* dot sizes represent relative dataset sizes. Meta-analyzed effect sizes are shown at a group level (gray diamonds) and across all studies (black diamonds), as well as between-dataset heterogeneity statistics (Cochran's Q test statistics). Diagnostic tool group differences were tested using an omnibus test for heterogeneity. Groups are ordered from largest to smallest.

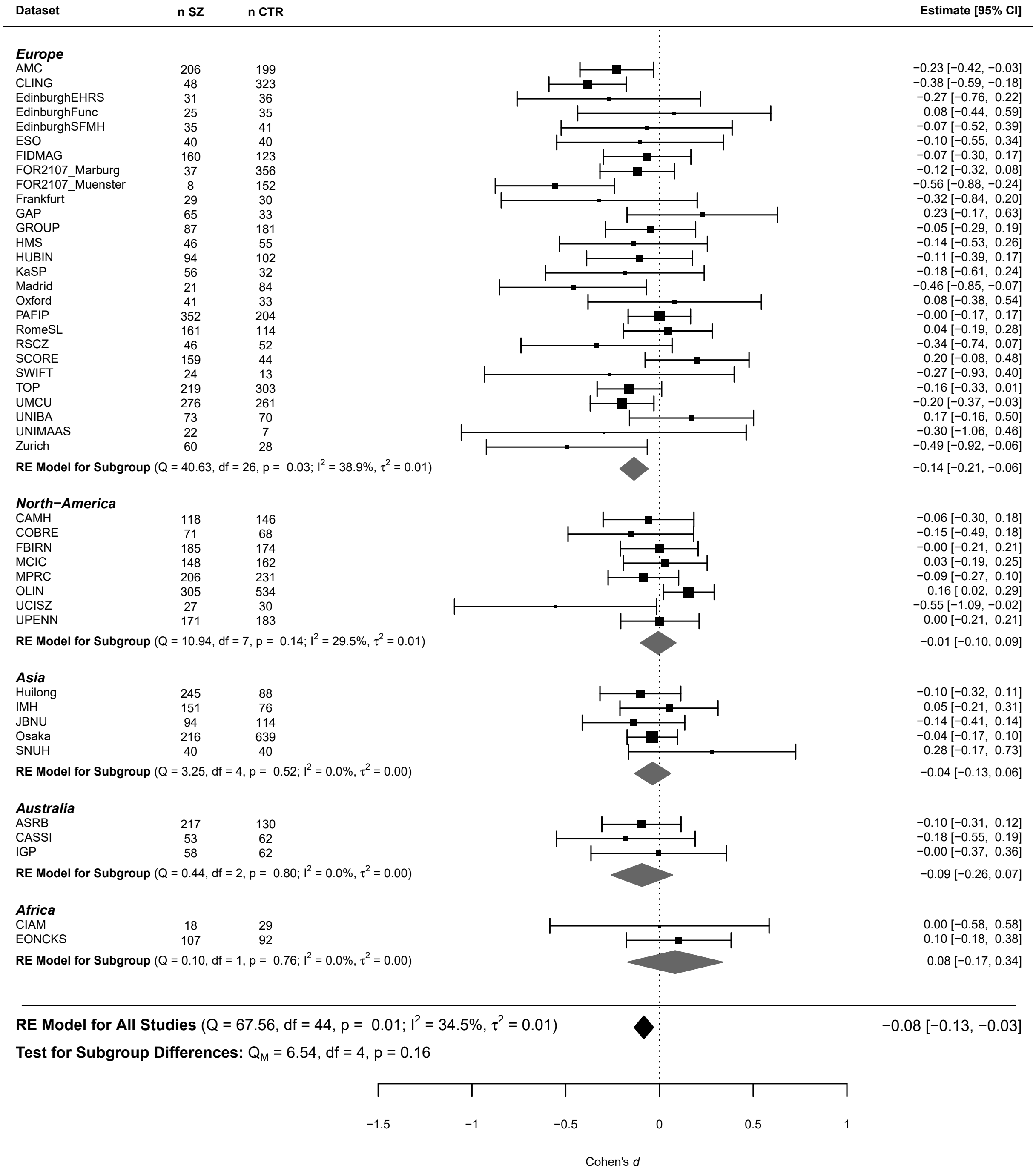

Fig. S22. Forest plot grouped by geographic origin, for random effects meta-analysis of rostral anterior cingulate thickness asymmetry differences between schizophrenia individuals and unaffected controls. Shown are the per-dataset effect sizes, including confidence intervals, and the numbers of affected individuals (n SZ) and unaffected controls (n CTR). Cohen's *d* dot sizes represent relative dataset sizes. Meta-analyzed effect sizes are shown at a group level (gray diamonds) and across all studies (black diamonds), as well as between-dataset heterogeneity statistics (Cochran's Q test statistics). Image slice orientation group differences were tested using an omnibus test for heterogeneity. Geographic origin groups are ordered from largest to smallest.

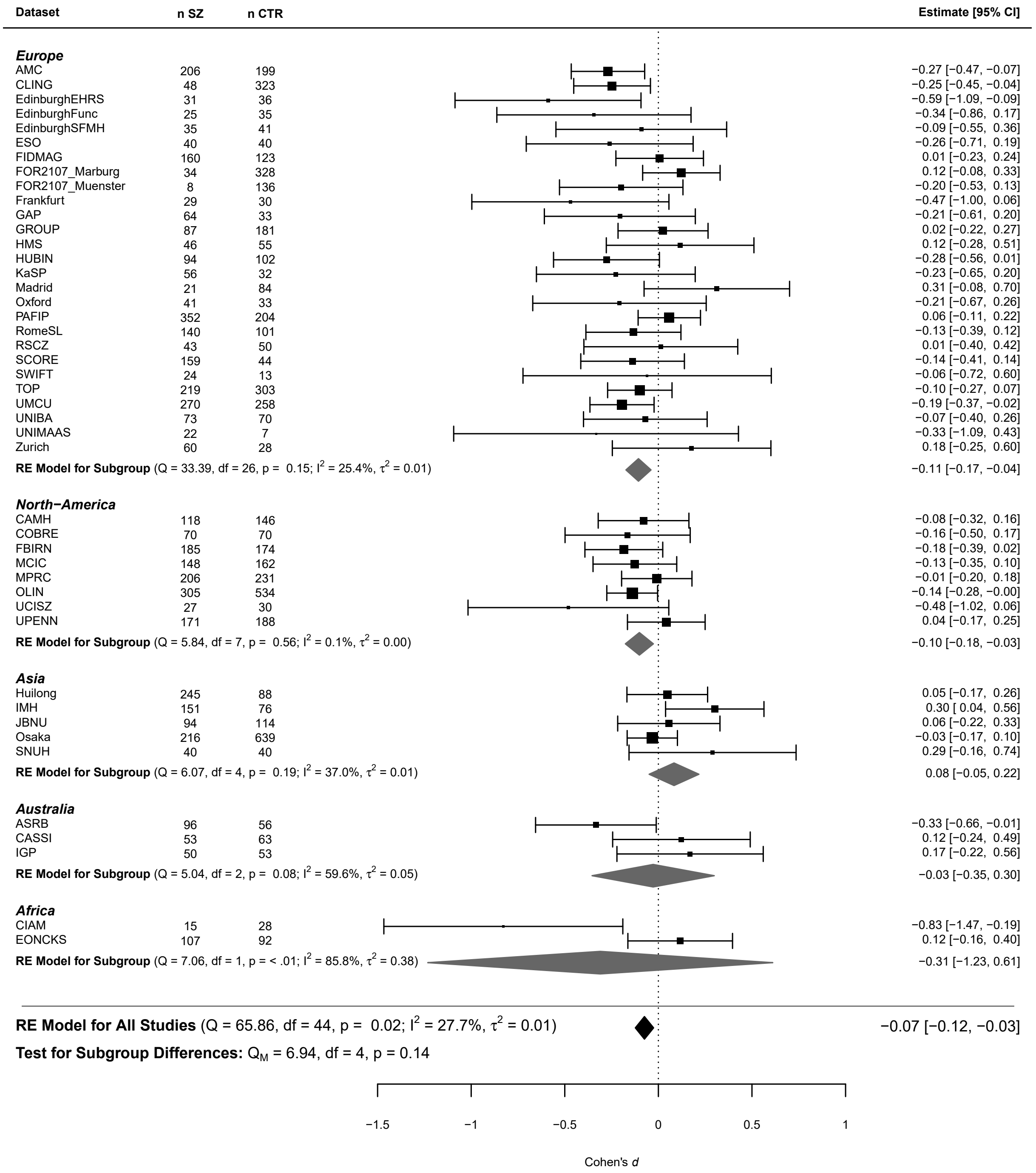

Fig. S23. Forest plot grouped by geographic origin, for random effects meta-analysis of middle temporal gyrus thickness asymmetry differences between schizophrenia individuals and unaffected controls. Shown are the per-dataset effect sizes, including confidence intervals, and the numbers of affected individuals (n SZ) and unaffected controls (n CTR). Cohen's *d* dot sizes represent relative dataset sizes. Meta-analyzed effect sizes are shown at a group level (gray diamonds) and across all studies (black diamonds), as well as between-dataset heterogeneity statistics (Cochran's Q test statistics). Geographic origin group differences were tested using an omnibus test for heterogeneity. Groups are ordered from largest to smallest.

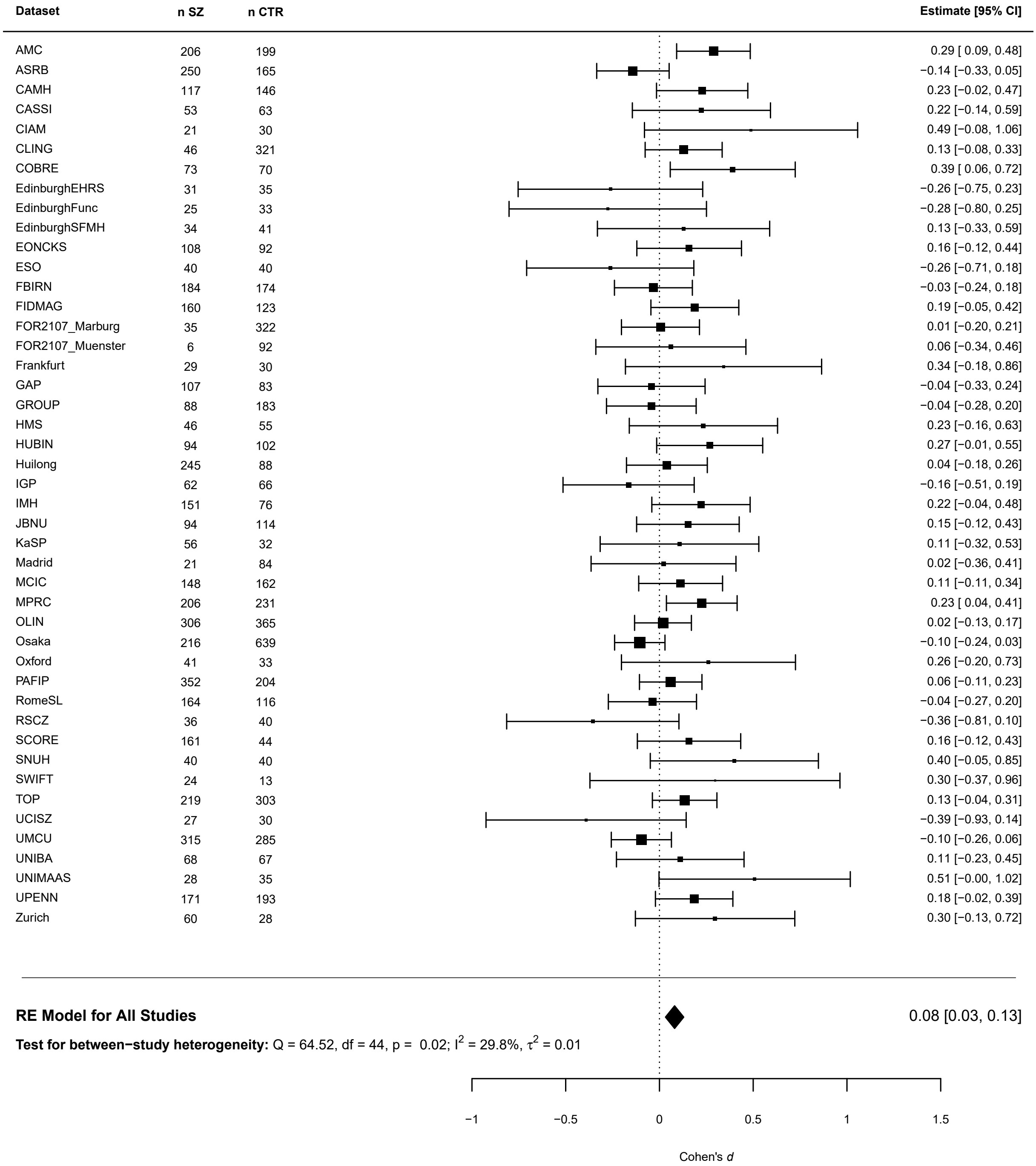

Fig. S24. Forest plot for random effects meta-analysis of pallidum volume asymmetry differences between schizophrenia individuals and unaffected controls, with age interaction. Shown are the per-dataset effect sizes, including confidence intervals, of affected individuals (n SZ) and unaffected controls (n CTR). Cohen's *d* dot sizes represent relative dataset sizes. The meta-analyzed effect sizes across all studies is shown (black diamond), as well as between-dataset heterogeneity statistics (Cochran's Q test statistics).

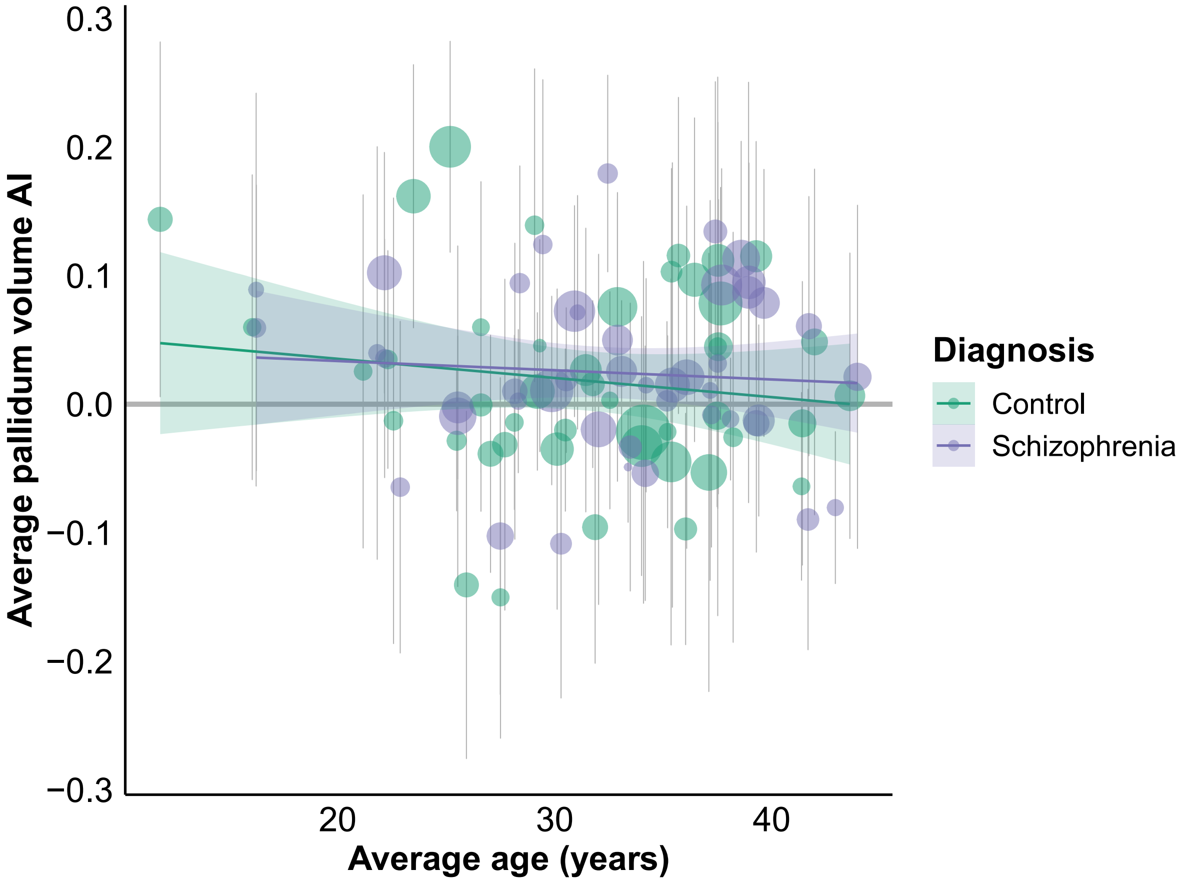

Fig. S25. Average pallidum volume asymmetry against average age per dataset. The average pallidum volume asymmetry index is plotted separately per dataset and for controls (green) and individuals with schizophrenia (purple). Point size indicates the relative sample size of each group per dataset, error bars show standard deviations of the average AI. The regression lines and their shaded confidence intervals show the linear relationships between pallidum volume AIs and age separately in cases and controls – revealing a possible, small diagnosis-by-age interaction effect.

Supporting tables

Table S1A. Dataset summary descriptions and data availability

Dataset	Country	N (Total)	N (SZ)	N (CTR)	M/F (SZ)	M/F (CTR)	Age (SZ) (years)	Age (CTR) (years)	Handedness (SZ) (N R/L/A)	Handedness (CTR) (N R/L/A)	Age at Onset (years)	Duration of illness (years)	PANSS Total Score	PANSS Negative Score	PANSS Positive Score	SANS Total Score	SAPS Total Score
**AMC**	Netherlands	405	206	199	180 / 26	130 / 69	22.15	23.49			19.99	2.18					
**ASRB**	Australia	429	263	166	177 / 86	79 / 87	38.59	39.28	240 / 23 / 0	136 / 30 / 0	23.61	14.98				18.53	
**CAMH**	Canada	264	118	146	70 / 48	77 / 69	43.95	43.6	107 / 8 / 3	139 / 6 / 1	24.9	19.17	53.11	13.97	13.92		
**CASSI**	Australia	116	53	63	35 / 18	33 / 30	35.17	30.49	45 / 4 / 3	53 / 4 / 4	23.08	12.11	33.38	8.58	8.17		
**CIAM**	South Africa	51	21	30	13 / 8	16 / 14	31.05	26.6	18 / 3 / 0	28 / 1 / 1	22.76	8.29	55.52	15.19	13.57		
**CLING**	Germany	371	48	323	35 / 13	132 / 191	32.44	25.18	43 / 5 / 0	307 / 15 / 1	24.4	7.73	49.38	11.91	11.17		
**COBRE**	United States	143	73	70	60 / 13	50 / 20	37.4	35.7	60 / 10 / 3	67 / 1 / 2	21.36	15.82	59.97	14.85	15.16		
**EdinburghEHRS**	United Kingdom	67	31	36	19 / 12	17 / 19	21.82	21.17	28 / 1 / 2	31 / 3 / 2	21.81						
**EdinburghFunc**	United Kingdom	60	25	35	11 / 14	18 / 17	37.16	37.51	14 / 9 / 0	32 / 2 / 0	22.25	15.5	43.04	10	10.64		
**EdinburghSFMH**	United Kingdom	76	35	41	23 / 12	23 / 18	37.51	38.22	20 / 4 / 6	26 / 4 / 2	22.97	14.35	56.37	14.06	13.37	27.94	
**EONCKS**	South Africa	200	108	92	74 / 34	51 / 41	34.17	31.86	94 / 10 / 0	80 / 10 / 0	20.93	13.24				33.03	23.21
**ESO**	Czech Republic	80	40	40	20 / 20	20 / 20	29.45	29.07	40 / 0 / 0	38 / 1 / 0	28.8	0.6	63.83	16.07	14.2		
**FBIRN**	United States	359	185	174	139 / 46	124 / 50	38.95	37.52	168 / 13 / 4	165 / 7 / 2	21.79	17.22	58.6	14.54	15.46	19.63	16.66
**FIDMAG**	Spain	283	160	123	124 / 36	54 / 69	39.64	37.54	156 / 3 / 1	123 / 0 / 0	23.01	15.53	76.21	22.58	16.83	37.19	
**FOR2107 Marburg**	Germany	403	37	366	23 / 14	143 / 223	37.22	34	32 / 5 / 0	340 / 25 / 1	21.17	15.89				18.76	13.24
**FOR2107 Muenster**	Germany	163	8	155	4 / 4	60 / 95	33.38	27.04	7 / 1 / 0	142 / 12 / 1	22.25	11.12				8.12	6.38
**Frankfurt**	Germany	59	29	30	20 / 9	13 / 17	38.1	35.2	29 / 0 / 0	30 / 0 / 0	35.62	10.91	67.19	16.7	16.93		
**GAP**	United Kingdom	209	122	87	85 / 37	32 / 55	27.49	25.93					62.35	16.47	15.22		
**GIPSI**	Colombia	43	43		35 / 8		33.53				19.07	14.12				32.21	9.26
**GROUP**	Netherlands	271	88	183	59 / 29	83 / 100	28.16	30.11									
**HMS**	Germany	101	46	55	32 / 14	28 / 27	28.39	35.38	38 / 4 / 4	39 / 3 / 3			90.22	22.09	21.07		
**HUBIN**	Sweden	196	94	102	70 / 24	69 / 33	41.71	41.97	78 / 9 / 3	90 / 6 / 3	24.51	17.05				22.29	9.02
**Huilong**	China	333	245	88	133 / 112	49 / 39	25.53	27.7									
**IGP**	Australia	138	68	70	40 / 28	38 / 32	41.67	36.03	57 / 1 / 8	60 / 1 / 8	22.87	18.82	55.47	14.54	13.78	29.11	17.24
**IMH**	Singapore	227	151	76	105 / 46	47 / 29	33.08	31.75	138 / 12 / 1	68 / 8 / 0	25.92	6.53	39.91	8.99	10.62		
**JBNU**	South Korea	208	94	114	57 / 37	48 / 66	39.29	41.41	88 / 2 / 4	113 / 0 / 0	29.99	9.03	52.96	12.63	14.23		
**KaSP**	Sweden	88	56	32	34 / 22	15 / 17	30.29	27.5	49 / 5 / 1	32 / 0 / 0		1.21	74.04	17.05	18.62		
**Madrid**	Spain	105	21	84	17 / 4	59 / 25	16.24	11.82	19 / 2 / 0	75 / 4 / 0	15.76	0.48	96.48	24.62	23.71		
**MCIC**	United States	311	148	163	113 / 35	101 / 62	32.89	31.43	131 / 7 / 6	149 / 5 / 9	22.75	10.22				23.31	22.81
**MPRC**	United States	437	206	231	128 / 78	96 / 135	35.42	37.1									
**OLIN**	United States	868	312	556	174 / 138	310 / 246	37.69	37.64									
**Osaka**	Japan	855	216	639	118 / 98	318 / 321	36.08	34.09	205 / 10 / 1	602 / 36 / 1	24.43	11.22	80.88	19.6	18.67		
**Oxford**	United Kingdom	74	41	33	24 / 17	15 / 18	16.25	16.06			14.48	1.79		16.2	22.24		
**PAFIP**	Spain	556	352	204	214 / 138	127 / 77	29.86	29.2	295 / 22 / 21	182 / 12 / 9	28.9	1.01	63.52	10.41	19.66	9.78	26.31

**RomeSL**	Italy	280	164	116	110 / 54	73 / 43	39.4	37.48	153 / 5 / 2	15 / 1 / 0	24.5	14.91	86.3	20.97	20.91	28.82	31.48
**RSCZ**	Russia	98	46	52	46 / 0	52 / 0	22.16	22.31	46 / 0 / 0	52 / 0 / 0	21.07	1.1	59.85	18.54	11.24		
**SCORE**	Switzerland	205	161	44	117 / 44	17 / 27	25.53	25.48	147 / 12 / 2	40 / 4 / 0	24.46	1.07				15.8	
**SNUH**	South Korea	80	40	40	18 / 22	20 / 20	22.88	22.57	0 / 35 / 5	0 / 35 / 5	22.32	5.96	68.6	17.18	16.57		
**SWIFT**	Switzerland	37	24	13	17 / 7	5 / 8	34.21	29.31	24 / 0 / 0	13 / 0 / 0	24.77	9.51	57.96	12.75	16.38		
**TOP**	Norway	522	219	303	130 / 89	159 / 144	32.02	35.36	172 / 22 / 2	279 / 22 / 2	23.93	8.27	62.02	15.53	14.87		
**UCISZ**	United States	57	27	30	22 / 5	23 / 7	42.93	41.37	22 / 2 / 3	25 / 4 / 1	25	17.5	59.96	16.04	15.56	22.81	13.41
**UMCU**	Netherlands	600	315	285	236 / 79	165 / 120	30.91	32.89	274 / 26 / 7	225 / 36 / 2	21.91	9.06	65.54	16.59	15.86		
**UNIBA**	Italy	143	73	70	54 / 19	28 / 42	33.48	26.6	49 / 4 / 20	51 / 2 / 17	20.69	11.81	78.59	22.1	17.41		
**UNIMAAS**	Netherlands	66	31	35	21 / 10	24 / 11	28.32	28.14	4 / 20 / 0	4 / 18 / 0	21.13	7.19	49.65	11.97	12.58		
**UPENN**	United States	370	177	193	105 / 72	90 / 103	38.93	36.44	148 / 23 / 6	174 / 15 / 4	20.74	17.35				23.69	18.28
**Zurich**	Switzerland	88	60	28	45 / 15	18 / 10	30.53	32.54	10 / 44 / 6	0 / 24 / 4	22.23	8.36	48.65	14.5	10.72	24.92	
**TOTAL/MEAN**		11095	5080	6015	3386 / 1694	3149 / 2866	33.32	32.98	3248 / 366 / 124	4025 / 357 / 85	23.63	9.98	63.62	16.03	15.59	21.32	20.8

N: Sample size; SZ: Individuals affected with schizophrenia; CTR: Unaffected controls; M: Males; F: Females; R: Right-handed; L: Left-handed; A: Ambidextrous; PANSS: Positive And Negative Syndrome Scale; SANS: Scale for the Assessment of Negative Symptoms; SAPS: Scale for the Assessment of Positive Symptoms.

Table S1B. Dataset medication information

Dataset	N Unmedicated	M/F Unmedicated	N First Generation (Typical)	M/F First Generation (Typical)	N Second Generation (Atypical)	M/F Second Generation (Atypical)	N Both (Atypical + Typical)	M/F Both (Atypical + Typical)	Mean CPZ (All)
**AMC**	11	10 / 1			122	104 / 18			484.37
**ASRB**	43	30 / 13	12	9 / 3	198	134 / 64	9	4 / 5	
**CAMH**	19	14 / 5	7	3 / 4	84	48 / 36	8	5 / 3	288.63
**CASSI**			3	1 / 2	46	30 / 16	4	4 / 0	598.24
**CIAM**	4	1 / 3	5	4 / 1	9	6 / 3	3	2 / 1	
**CLING**	8	7 / 1			35	24 / 11	5	4 / 1	651.04
**COBRE**			7	7 / 0	62	50 / 12	1	0 / 1	547.22
**EdinburghEHRS**	3	2 / 1	9	6 / 3	11	7 / 4	6	2 / 4	497.58
**EdinburghFunc**			8	4 / 4	15	6 / 9	2	1 / 1	590.32
**EdinburghSFMH**	11	6 / 5	1	1 / 0	23	16 / 7			309.85
**EONCKS**	9	8 / 1	17	11 / 6	79	54 / 25			
**ESO**					29	15 / 14	2	2 / 0	
**FBIRN**			20	17 / 3	137	102 / 35	10	6 / 4	373.3
**FIDMAG**	2	1 / 1	100	77 / 23	9	8 / 1	27	20 / 7	573.75
**FOR2107 Marburg**	6	5 / 1	3	0 / 3	25	17 / 8	3	1 / 2	403.72
**FOR2107 Muenster**	1	0 / 1			7	4 / 3			306.66
**Frankfurt**			3	3 / 0	26	17 / 9			605.14
**GAP**					10	7 / 3	3	3 / 0	194.77
**GIPSI**	3	2 / 1	2	2 / 0	29	22 / 7	9	9 / 0	422.95
**GROUP**									
**HMS**	6	4 / 2			39	27 / 12	1	1 / 0	312.96
**HUBIN**	6	4 / 2	40	31 / 9	38	26 / 12	10	9 / 1	272.67
**Huilong**			7	7 / 0	95	47 / 48			
**IGP**	10	5 / 5	3	1 / 2	53	33 / 20	2	1 / 1	655.24
**IMH**			60	45 / 15	66	45 / 21	24	15 / 9	200.23
**JBNU**	28	14 / 14	62	40 / 22	1	1 / 0	3	2 / 1	280.21
**KaSP**	29	19 / 10			25	14 / 11	2	1 / 1	
**Madrid**			20	16 / 4			1	1 / 0	
**MCIC**	8	6 / 2	10	6 / 4	117	90 / 27	7	5 / 2	533.53
**MPRC**									
**OLIN**									
**Osaka**	21	11 / 10	10	3 / 7	139	77 / 62	46	27 / 19	603.81
**Oxford**	41	24 / 17							353.82
**PAFIP**			24	16 / 8	328	198 / 130			134.28
**RomeSL**	11	6 / 5	26	13 / 13	82	59 / 23	42	30 / 12	302.23
**RSCZ**									
**SCORE**	121	92 / 29	2	2 / 0	38	23 / 15			203.18
**SNUH**	9	3 / 6			30	15 / 15	1	0 / 1	188.05

**SWIFT**			2	2 / 0	18	11 / 7			553.95
**TOP**	28	16 / 12	5	2 / 3	158	97 / 61	18	10 / 8	404.39
**UCISZ**									
**UMCU**	27	20 / 7	87	58 / 29	166	129 / 37	5	5 / 0	
**UNIBA**			4	4 / 0	43	33 / 10	7	4 / 3	641.97
**UNIMAAS**	31	21 / 10							
**UPENN**			13	9 / 4	65	39 / 26	5	3 / 2	481.65
**Zurich**	3	2 / 1			55	41 / 14	2	2 / 0	494.76
**TOTAL/MEAN**	499	333 / 166	572	400 / 172	2512	1676 / 836	268	179 / 89	394.38

N: Sample size; M: Males; F: Females; CPZ: Chlorpromazine equivalent medication dose. Grayed out numbers indicate medication groups that had insufficient sample size in a dataset (< 5 individuals).

Table S1C. Subset for multivariate analysis (complete individual-level data available to the central analysis team)

Dataset	N (Total)	N (SZ)	N (CTR)	M/F (SZ)	M/F (CTR)	Age (SZ) (years)	Age (CTR) (years)
**ASRB 1**	2	1	1	0 / 1	1 / 0	56.00	36.00
**ASRB 2**	4	3	1	2 / 1	1 / 0	46.00	30.00
**ASRB 3**	3	2	1	0 / 2	0 / 1	52.50	51.00
**CASSI**	81	35	46	20 / 15	21 / 25	32.97	30.91
**COBRE**	102	53	49	45 / 8	35 / 14	37.70	37.41
**EONCKS**	199	107	92	73 / 34	51 / 41	34.07	31.86
**ESO**	12	6	6	3 / 3	3 / 3	33.00	28.83
**FOR2107 Marburg**	142	16	126	9 / 7	42 / 84	38.19	35.37
**FOR2107 Muenster**	32	1	31	0 / 1	13 / 18	27.00	28.58
**IGP**	7	2	5	1 / 1	3 / 2	41.33	35.00
**IMH**	147	89	58	59 / 30	35 / 23	33.94	32.57
**MPRC 1**	158	95	63	60 / 35	22 / 41	37.36	43.05
**MPRC 2**	215	76	139	42 / 34	55 / 84	32.13	34.46
**MPRC 3**	63	35	28	26 / 9	18 / 10	37.31	35.86
**OLIN**	649	297	352	167 / 130	201 / 151	38.04	38.87
**UCISZ**	57	27	30	22 / 5	23 / 7	42.93	41.37
**UNIBA**	69	30	39	21 / 9	11 / 28	33.77	26.26
**Zurich**	88	60	28	45 / 15	18 / 10	30.53	32.54
**TOTAL/MEAN**	2030	935	1095	595 / 340	553 / 542	35.97	35.84

N: Sample size; SZ: Individuals affected with schizophrenia; CTR: Unaffected controls; M: Males; F: Females.

Table S2. Dataset-specific information

Dataset	Instrument for SZ diagnosis	Recruitment of healthy controls and absence of SZ diagnosis	Instrument for handedness assessment	Number of scanners	Scanner manufacturer and type	Imaging protocols	Slice orientation	FreeSurfer version	Operating system
**AMC**	DSM-IV	Random mailing/schools in the neighborhood of Amsterdam. No life-time psychotic symptoms.	No handedness data	1	3T Philips Intera	TR: 8-9.8, M=9,4 (0,41). TE:3,5-4,6, M=4,26 (0,46). Slice thickness: 1/1.2, flip angle: 8degr, rows/columns: 192-288, M=255,49 (7,00). Pixel spacing: 1mm	Axial	v5.0.0	Linux CentOS 4 x86_64
**ASRB**	ICD-10	Mini-International Neuropsychiatric Interview (MINI)	Edinburgh Handedness Inventory	5	1.5T Siemens Avanto	High-resolution T1-weighted structural magnetic resonance imaging (sMRI) brain scans (MPRAGE) were acquired using an optimized magnetization prepared rapid acquisition gradient echo on 1.5 T Siemens Avanto scanners (Siemens, Erlangen, Germany) across five Australian research sites. Image parameters were set to 176 slices of 1mm thickness, no gap with field-of-view 250 x 250 mm2, repetition time 1980 ms, echo time 4.3 ms, data acquisition matrix 256 x 256, with a flip matrix of 15°, resulting in a voxel size of 0.98×0.98×1.0 mm3.	Sagittal	v5.1.0	Mac OS X
**CAMH**	DSM-IV	DSM-IV	Self-report	1	1.5T General Electric	SPGR, TR/TE/TI=12.3/5.3/300ms, flip angle=20°, 256x256x128 matrix, FOV=240x240mm, slice thickness=1.5mm.	Axial	v5.3.0	Xubuntu x86_64 linux
**CASSI**	A diagnosis of schizophrenia or schizoaffective disorder was determined using the Structured Clinical Interview for Diagnostic and Statistical Manual IV-TR Axis I Disorders by a clinician trained in administration of the SCID which was confirmed independently by another clinician.	Exclusion criteria for healthy controls consisted of a personal history of or a first-degree relative with a DSM-IV Axis I psychiatric diagnosis, history of substance abuse or dependence (within the past 5 years), head injuries with loss of consciousness, seizures, central nervous system infection, untreated diabetes or hypertension or mental retardation.	Edinburgh Handedness Inventory	1	3T Philips Achieva	T1 weighted gradient echo planar. Number of acquisitions: 180. Flip angle: 90 degrees. TE: 2.4 ms. TR 5.4 ms. Field of view 256. Image dimensions 256x256 voxels. Voxel size = 1x1x1 mm.	Sagittal	v5.1.0	Mac OS X 10.8
**CIAM**	SCID using DSM-IV by clinically trained research team members. Only those participants which made a clear diagnosis of schizophrenia were included in our cohort.	Clear of any Axis I disorders as per SCID using DSM-IV	Edinburgh Handedness Inventory	1	3T Siemens Allegra	MPRAGE sequence: TR = 2530 ms, graded TE = 1.53, 3.21, 4.89, 6.57 ms, flip angle = 7°, FOV = 256 mm, slice thickness = 1.33 mm, 128 slices, voxel size 1.3x1.0x1.3, scan time 8:06. Single channel coil used.	Sagittal	v5.3.0	Linux
**CLING**	ICD-10 and DSM-IV	Clinical interview	Edinburgh Handedness Inventory	1	3T Siemens TIM Trio	MRI scanning was performed on a 3.0-Tesla Magnetom TIM Trio (Siemens, Erlangen, Germany). A T1-weighted, 3D magnetization prepared rapid gradient echo sequence (MPRAGE) (TR/TE/TI/FA=2250 ms/3.26 ms/900 ms/9°; image matrix = 256 x 256; duration 8 min and 26 sec) was acquired generating 192 sagittal slices with a voxel size of 1 mm3.	Sagittal	v5.3.0	Ubuntu 12.04
**COBRE**	Structured Clinical Interview for DSM-IV Axis I Disorders (SCID) for diagnostic confirmation (consensus was reached by two research psychiatrists using the SCID-DSM-IV-TR, patient version) and evaluation for co-morbidities.	Healthy controls were recruited from the same geographic location via IRB-approved advertisement and completed the SCID-Non-Patient Edition to rule out Axis I conditions. Additional exclusion criteria for HCs included a current or past psychiatric disorder (with the exception of one lifetime major depressive episode), head trauma with a loss of consciousness greater than 5 min, recent history of substance abuse or dependence, depression or antidepressant use within the past 6 months, lifetime antidepressant use of more than one year, and history of a psychotic disorder in a first-degree relative.	Self-report	1	3T Siemens TIM Trio	T1-weighted images were acquired with a 5-echo multi-echo MPRAGE sequence [TE (echo times) = 1.64, 3.5, 5.36, 7.22, 9.08 ms, TR (repetition time) = 2.53 s, TI (inversion time) = 1.2 s, 7° flip angle, number of excitations (NEX) = 1, slice thickness = 1 mm, FOV (field of view) = 256 mm, resolution = 256x256].	Sagittal	v5.3.0	Linux RedHat

**EdinburghEHRS**	Psychiatric hospital case notes reviewed with the Operational Criteria (OPCRIT) check-list; diagnosis confirmed using the Present State Examination (PSE) according to the DSM-IV criteria for schizophrenia or schizophreniform disorder.	Controls were recruited from the social networks of schizophrenia high-risk participants and from local youth groups. The main criteria for recruitment was no personal or family history of major psychiatric disorder. Controls were matched to cases at the group level in terms of age, sex, education and ethnicity.	Annett Handedness Scale	1	1T Siemens	Scanned with a 1 Tesla 42 SPE Siemens MRI scanner (Siemens, Erlangen, Germany). 128 contiguous coronal T1-weighted slices (thickness1.88 mm, field-of-view 250 × 250 mm) were obtained using a Magnetisation Prepared Rapid Acquisition of Gradient Echo (MPRAGE)sequence (TR=10ms, TE=4ms, TI=200ms, relaxation time 500ms).	Coronal	v5.3.0	Linux
**EdinburghFunc**	Diagnosis established using Structured Clinical Interview for DSM Disorders (SCID) according to the DSM criteria.	Control participants were recruited from the pool of unaffected non-genetic relatives and social contacts of case participants. Absence of schizophrenia diagnosis was confirmed through SCID screening.	Self-report	1	1.5T General Electric Signa	A coronal gradient echo sequence with magnetization preparation and produced 128 coronal high-resolution T1-weighted images, which were used for structural image analysis (time of inversion [TI] 600 msec, echo time 3.4 msec, flip angle 15, field of view 22, slice thickness 1.7 mm, matrix 256 192).	Axial	v5.1.0	Linux
**EdinburghSFMH**	Diagnosis established using Structured Clinical Interview for DSM Disorders (SCID) according to the DSM criteria.	Control participants were recruited from the pool of unaffected non-genetic relatives and social contacts of case participants. Absence of schizophrenia diagnosis was confirmed through SCID screening.	Self-report	1	3T Siemens Verio	Used T1-weighted, magnetisation prepared rapid acquisition gradient echo (MP-RAGE) sequence prescribed using the AC-PC line, providing 160 saggital slices of 1.0mm thickness, with 256 x 256mm2 field of view, matrix size 256 x 256mm2. Further scan parameters – repetition time = 2300ms, echo time = 2.98ms, inversion time = 900ms and flip angle = 9 degrees.	Sagittal	v5.3.0	Linux
**EONCKS**	DSM-IV (PANS) clinical interview	DSM IV (PANS) clinical interview	Edinburgh Handedness Inventory	1	3T Siemens Allegra	MPRAGE 2080 ms repetition time; 4.88 ms echo time, Field of view: 230 mm, 176 slices, 0.9 mm X 0.9 mm X 1 mm voxel size.	Sagittal	v6	CentOS
**ESO**	M.I.N.I., ICD-10	M.I.N.I.	Edinburgh Handedness Inventory	1	3T Siemens TIM Trio	MP-RAGE 3D, 1mm thickness, acquisition matrix 256 x 256, TR=2300ms, TE=4.63ms, TI=900ms.	Sagittal	v5.3.0	Linux
**FBIRN**	SCID-I/P (DSM-IV-TR)	SCID-I/NP (DSM-IV-TR)	Edinburgh Handedness Inventory	7	3T Siemens TIM Trio; 3T General Electric Discovery MR750	High-resolution structural imaging scans were acquired on six 3T Siemens Tim® Trio System and one 3T General Electric Discovery MR750 scanner. MP-RAGE scan parameters for the Siemens scanner were: scan plane=sagittal, TR/TE/TI=2300/2.94/1100ms, GRAPPA acceleration factor=2, flip angle=9°, resolution=256×256x160, FOV=220mm2, voxel size=0.86x0.86x1.2mm, and NEX=1. IR-SPGR scan parameters for the General Electric scanner were: scan plane=sagittal, TR/TE/TI=5.95/1.99/450ms, ASSET acceleration factor=2, a flip angle=12°, resolution=256×256x166, FOV=220mm2, voxel size=0.86x0.86x1.2mm, and NEX=1. All scans covered the entire brain.	Sagittal	v5.3.0	CentOS 64bit
**FIDMAG**	DSM-IV	SCID. We also ask about any personal or first-degree relatives of any mental major disorder	Self-report	1	1.5T General Electric Signa	180 axial slices; 1mm slice thickness, no gap, matrix size 512x512; 0.5x0.5x1mm3 voxel resolution; TE 4ms, TR 2000ms, flip angle 15º	Axial	v5.3.0	Linux Ubuntu
**FOR2107_Marburg**	DSM-IV-TR using SCID-I	DSM-IV-TR: no current or former psychiatric diagnosis according to SCID I	Edinburgh Handedness Inventory	1	3T Siemens Magnetom TrioTim Syngo	MPRAGE imaging sequence. 1 acquisition. Flip angle: 9 degrees. TE: 2.26 ms. TR: 1900 ms. TI: 900 ms. Acceleration factor: 2Field of view: 256. Image dimensions: 256x256x176 voxels. Voxel size: 1x1x1 mm.	Sagittal	v5.3.0	Red Hat Enterprise Linux Server release 5.11 (Tikanga)
**FOR2107_Muenster**	Diagnosis were established based on SCID interviews including all available clinical information.	SCID, and family history for major psychiatric disorders was carefully assessed.	Edinburgh Handedness Inventory	1	3T Siemens PRISMA	MPRAGE imaging sequence. 1 acquisition. Flip angle: 8 degrees. TE: 2.28 ms. TR: 2130 ms. TI: 900 ms. Acceleration factor: 2. Field of view: 256. Image dimensions: 256x256x192 voxels. Voxel size: 1.0x1.0x1.0mm.	Sagittal	v5.3.0	Red Hat Enterprise Linux Server release 5.11 (Tikanga)
**Frankfurt**	through SCID I; a psychiatrist did the diagnosis.	SCID I	Edinburgh Handedness Inventory	1	3T Siemens Trio	176 slices, slice thickness 1mm, TR=7.92 ms, TE= 2.48 ms, voxel resolution= 1x1x1 mm, flip angle= 16°.	Sagittal	v5.1.0	Linux Axia
**GAP**	ICD-10 using Schedules for Clinical Assessment in Neuropsychiatry (SCAN)	Selected using internet and newspaper adverts and distribution of leaflets; administered the Psychosis Screening Questionnaire to exclude psychosis	No handedness data	1	3T General Electric Signa HDx	SAGITTAL ADNI MPRAGE GE, slice thickness = 1.2mm, spatial positions = 166 slices, flip angle = 8º, fov = 260mm x 260mm, TR/TE/TI = 6.988/2.848/650ms, matrix = 256mm x 256mm.	Sagittal	v5.3.0	Linux

**GIPSI**	DIGS using DSM-IV-TR.	Case-only cohort	No handedness data	1	3T Philips Ingenia	Sequence 3D T1-weighted TFE, 160 axial slices, 1x0.6x0.6 mm3 voxel resolution, TE/TR= 2.063/4.756 ms, flip angle= 15	Axial	v5.3.0	Linux Ubuntu
**GROUP**	DSM-IV via CASH interview	CASH interview	No handedness data	2	3T Siemens Allegra syngo MR A30	Modified Driven Equilibrium Fourier Transform (MDEFT) sequence; 176 slices, 1 mm isotropic voxel size, echo time 2.4 ms, repetition time 7.92 ms, inversion time 910 ms, flip angle 15°, total acquisition time 12 min 51 s; Magnetization Prepared Rapid Acquisition Gradient-Echo (MPRAGE; Alzheimer's Disease Neuroimaging Initiative) sequence 192 slices, 1 mm isotropic voxel size, echo time 2.6 ms, repetition time 2250 ms, inversion time 900 ms, flip angle 9°, total acquisition time 7 min 23 s. The matrix size was 256 × 256 and field of view was 256 × 256 mm2. The number of excitations was one. Two sequences were used because of a scanner update during data collection.	Sagittal	v5.0.0	Mac OS X
**HMS**	ICD-10 and DSM-IV	Clinical interview	Edinburgh Handedness Inventory	1	1.5T Siemens Magnetom Sonata	MRI scanning was performed on a 1.5-Tesla Magnetom Sonata (Siemens, Erlangen, Germany). A T1-weighted, magnetization prepared rapid gradient echo sequence (MPRAGE) (TR/TE/TI/FA=1900 ms/4.0 ms/700 ms/15°; image matrix = 256 x 256) was acquired generating 176 consecutive sagittal slices with a voxel size of 1 mm3. ~5 min.	Axial	v5.1.0	Linux
**HUBIN**	DSM-III-R/DSM-IV based on SCID-I and reviews of medical records	SCID-I	Edinburgh Handedness Inventory	1	1.5T General Electronic Signa	T1-weighted images, using a three-dimensional spoiled gradient recalled (SPGR) pulse sequence, were acquired with the following parameters; 1.5 mm coronal slices, no gap, 35° flip angle, repetition time (TR) = 24 ms, echo time (TE) = 6.0 ms, number of excitations (NEX) = 2, field of view (FOV) = 24 cm, acquisition matrix = 256 × 192. T2-weighted images were acquired with the following parameters; 2.0 mm coronal slices, no gap, TR = 6,000 ms, TE = 84 ms, NEX = 2, FOV = 24 cm, acquisition matrix = 256 × 192.	Coronal	v5.3.0	Linux RedHat
**Huilong**	DSM-IV	They participated through community recruitment. Confirmed as healthy controls through Interview with a psychiatist.	No handedness data	3	3T Siemens Verio; 3T GE Signa HDxt	T1-weighted, 3D BRAVO, 1x1x1mm, TE/TR/TI=2.5/6.8/1100ms, flip angle=7 degrees.	Sagittal	v5.3.0	Linux
**IGP**	ICD-10	Mini-International Neuropsychiatric Interview (MINI)	Edinburgh Handedness Inventory	1	3T Philips Achieva TX	MPRAGE imaging sequence. 200 acquisitions. Flip angle: 8 degrees. TE 4.1 ms. TR 8.9 ms. Field of view 240. Image dimensions 268x268. Voxel size 0.9x0.9x0.9.	Sagittal	v5.3.0	Mac OS X
**IMH**	SCID-P (DSM-IV)	SCID-NP	Edinburgh Handedness Inventory	1	3T Philips Achieva	MPRAGE imaging sequence. 3 acquisitions. Flip angle: 8 degrees. TE: 3.3 ms. TR 7200 ms. Field of view: 230. Image dimensions: 256x204 voxels. Voxel size: 0.9x0.9x0.9 mm.	Axial	v5.3.0	Mac OS X
**JBNU**	DSM-5	Healthy controls were recruited through interview using the non-patient version of the Structured Clinical Interview for DSM-IV (SCID-IV)	Edinburgh Handedness Inventory	1	3T Siemens MAGNETOM Verio Syngo	MPRAGE imaging sequence. Flip angle: 9 degrees. TE 2.45 ms. TR 1900 ms. Field of view 250. Image dimensions 350x263x350. Voxel size 1x1x1 mm.	Sagittal	v7.1.0	Linux
**KaSP**	SCID-I	MINI	Self-report	1	3T General Electric	3D IR prep fast SPGR, TR=7.904ms, TE=3.06ms, TI = 450ms, flip angle = 12, 146 slices, voxel size = 0.934 x 0.934 x 1.2 mm3, matrix = 256 x 256.	Sagittal	v5.3.0	Linux RedHat Enterprise 6.5

**Madrid**	A DSM-IV-TR diagnosis of schizophrenia in the schizophrenia group of control or its absence in the unaffected sample was established after a clinical interview using the Kiddie Schedule for Affective Disorders and Schizophrenia (K-SADS-PL) or the Structured Clinical Interview for DSM Disorders (SCID), for participants younger or older than 18, as appropriate. SCID for adults and K-SADS for adolescents	A DSM-IV-TR diagnosis of schizophrenia in the schizophrenia group of control or its absence in the unaffected sample was established after a clinical interview using the Kiddie Schedule for Affective Disorders and Schizophrenia (K-SADS-PL) or the Structured Clinical Interview for DSM Disorders (SCID), for participants younger or older than 18, as appropriate.	Neurological Evaluation Scale (NES)	1	1.5T Philips Intera	Sagittal T1 (FFE) 3D, 175 slices, voxel 1x0.94x0.94 mm3, FOV 256x256, TR=25 ms, TE=9.2 ms	Sagittal	v6	Linux
**MCIC**	A Structured Clinical Interview for DSM-IV (SCID/SCID-NP for controls) or the Comprehensive Assessment of Symptoms and History (CASH) were used to diagnose primary and co-morbid psychiatric disorders in controls and patients.	A Structured Clinical Interview for DSM-IV (SCID/SCID-NP for controls) or the Comprehensive Assessment of Symptoms and History (CASH) were used to diagnose primary and co-morbid psychiatric disorders in controls and patients.	Annett Handedness Scale	4	1.5T, 3T Siemens and General Electric	T1 scans: TR = 2530 ms for 3 T, TR = 12 ms for 1.5 T; TE = 3.79 ms for 3 T, TE = 4.76 ms for 1.5 T; FA = 7 for 3 T, FA = 20 for 1.5 T; TI = 1100 for 3 T; Bandwidth = 181 for 3 T, Bandwidth = 110 for 1.5 T; 0.625×0.625 mm voxel size; slice thickness 1.5 mm; FOV 256×256×128 cm matrix; FOV = 16 cm (could be increased to 18 cm when needed for full brain coverage).	Coronal	v4.0.1	Linux (multiple versions)
**MPRC**	Patients at the MPRC clinic; Clinical interview with SCID DSM-IV.	Local advertisement and clinical interview	No handedness data	3	3T Siemens Allegro; 3T Siemens Trio	Siemens Allegro: T1-weighted, 3D MPRAGE, 1x1x1mm, TE/TR/TI=4.3/2500/1000ms, flip angle=8 degrees. Siemens Trio: T1-weighted, 3D MPRAGE, 1x1x1mm, TE/TR/TI=2.9/2300/900ms, flip angle=9 degrees.	Sagittal	v5.3.0	Linux
**OLIN**	SCID	SCID-NP	No handedness data	1	3T Siemens Allegra	T1-weighted, 3D magnetization prepared rapid gradient-echo (MPRAGE) sequence (TR/TE/TI=2200/4.13/766 ms, flip angle=13°, voxel size [isotropic]=0.8mm, image size=240 x 320 x 208 voxels), with axial slices parallel to the AC-PC line.	Axial	v5.1.0	CentOS 7
**Osaka**	SCID-P; DSM-IV	SCID-NP	Edinburgh Handedness Inventory	2	1.5T GE Signa Excite; 3T GE Signa HDxt	3D-IR-FSPGR, TR/TE/TI=12.6/4.2/400ms, flip angle=15°, 256x256x124 matrix, FOV=240x240mm, slice thickness=1.4mm, Nex=1, No Asset, QD Head coil; 3D-IR-FSPGR, TR/TE/TI=7.2/2.9/400ms, flip angle=11°, 256x256x172 matrix, FOV=240x240mm, slice thickness=1.0mm, Nex=1, No Asset, 8ch Brain coil.	Sagittal	v5.3.0	SUSE Linux Enterprise Server 10; Red Hat Enterprise Linux 6
**Oxford**	KSADS-PL, DSM-IV	General population, recruited through GP practices. All screened with KSADS-PL.	No handedness data	1	1.5T Siemens Sonata	3D T1-weighted FLASH imaging sequence. Acquisition matrix 256 x 256, 208 slices, 1 x 1 mm^2 in-plane resolution, slice thickness 1 mm, TE/TR = 5.6/12 ms, flip angle alpha = 19°.	Sagittal	v5.3.0	Linux
**PAFIP**	Diagnosis of schizophrenia was confirmed using the Structured Clinical Interview for DSM-IV (SCID-I).	The unaffected controls had no current or past history of psychiatric neurological, or general medical illnesses, including substance abuse, according to an abbreviated version of the Comprehensive Assessment of Symptom and History (CASH).	Edinburgh Handedness Inventory	2	1.5T General Electric; 3T General Electric	Three-dimensional T1-weighted images, using a spoiled grass (SPGR) sequence acquired in the coronal plane with: echo time (TE)=5 ms, repetition time (TR)=24 ms, numbers of excitations (NEX)=2, rotation angle=45°, field of view (FOV)=26×19.5 cm, slice thickness=1.5mm and a matrix of 256×192.	Coronal	v5.0.0	Ubuntu 11.04 x86_64
**RomeSL**	Structured clinical interview for DSM-IV-TR (SCID-I/P)	Structured Clinical Interview for DSM-IV-TR Axis I Disorders, Research Version, Non-patient Edition (SCID-I-NP).	Edinburgh Handedness Inventory	1	3T Siemens Allegra	MPRAGE imaging sequence. T1-weighted, 3D MDEFT, 1x1x1xmm, TE/TR =2.4/7.92 ms, flip angle=15. TE: 910 ms. Acceleration factor: 1. Field of view: 256. Image dimensions: 176x224x256 voxels.	Sagittal	v6.0dev	Linux
**RSCZ**	ICD-10	Unaffected controls were recruited from acquaintances of the researchers and Mental Health Research Center staff, absence of SZ diagnosis were confirmed by self-report	Partly Annett Handedness Scale, partly Self-report	1	3T Philips Achieva	A turbo field echo sequence covering the whole brain. TR = 8,200 ms, TE = 3.7 ms, TI = 1,020 ms, flip angle = 8, SENSE factor = 1.5, FOV = 240 mm, voxel size of 0.83 × 0.83 mm with a slice thickness of 1 mm, no gap.	Sagittal	v5.3.0	CentOS 6.6
**SCORE**	DSM-IV	Self-report	Self-report	1	3T Magnetom Verio	MPRAGE: acquisition matrix: 256×256×176, isotropic spatial resolution: 1x1x1mm3, TI=1000ms, TR=2s, TE=3.4 ms, flip angle: 8° and bandwidth of 200 Hz/pixel.	Sagittal	v6.0dev	Ubuntu 18.04 LTS

**SNUH**	Schizophrenia patients were diagnosed using the Structured Clinical Interview for DSM, fourth edition (DSM-IV), Axis I (SCID-I)	Controls were screened, and confirmed using the SCID Nonpatient Edition (SCID-NP). They were excluded when they had any past or current SCID-NP axis I diagnoses and first- to third-degree biological relations with psychotic disorders.	Annett Handedness Scale	1	3T Siemens Trio	High-resolution T1-weighted, three-dimensional Magnetization Prepared Rapid Gradient Echo (TR = 670ms; TE=1.89ms; FOV=250mm; FA=9°; voxel size=1x1x1mm3).	Sagittal	v5.3.0	Mac OS X 10.9
**SWIFT**	SCID	Screening questionnaire of the Structured Clinical Interview for DSM-IV Axis I Disorders or the Mini-International Neuropsychiatric Interview.	Edinburgh Handedness Inventory	1	3T Siemens Trio	MPRAGE imaging sequence. Flip angle: 7 degrees. TE: 92 ms. TR: 8000 ms. Field of view: 256. Image dimensions: 158x255x255 voxels. Voxel size: 2x2x2 mm.	Oblique	v7.1.0	Linux CentOS 7 x86_64-7.1.0-20200511-813297b
**TOP**	The Structured Clinical Interview for DSM-IV axis 1 disorders (SCID-IV).	Healthy controls were randomly drawn from the national population registry in the same geographical area as the patients, and invited by letter to participate. They were screened prior to participation. Absence of current or previous history of a psychiatric disorder was determined by self-report. Current symptomatology was screened for on the day of inclusion using the Prime MD and alcohol and drug use were screened for using AUDIT/DUDIT.<br><br>The exclusion criteria for healthy controls were:<br>- Age outside of the range 18-65 years.<br>- Current or previous psychiatric disorder.<br>- History of severe mental illness in a first-degree relative.<br>- Any alcohol or drug abuse or dependence.	Self-report	1	1.5T Siemens Magnetom Sonata	Two sagittal T1-weighted magnetization prepared rapid gradient echo (MPRAGE) volumes were acquired with the Siemens tfl3d1_ns pulse sequence (TE = 3.93 ms, TR = 2730 ms, TI = 1000 ms, flip angle = 7°; FOV = 24 cm, voxel size= 1.33 x 0.94 x 1 mm3, number of partitions = 160).	Sagittal	v4.5.0	Linux CentOS or Ubuntu
**UCISZ**	SCID-I/P (DSM-IV-TR)	SCID-I/NP (DSM-IV-TR)	Edinburgh Handedness Inventory	1	3T Philips Achieva	High-resolution structural imaging scans were acquired on on a 3T Philips Achieve using a T1 Turbo Fast Spin Echo (TFE) with 200 sagittal slices, 320x274 matrix size, 0.75mm3 isotropic voxels, TR = 11ms, TE =4.562ms, flip angle = 18°, Turbo = 180.	Sagittal	v6.0dev	CentOS 64bit
**UMCU**	CASH or SCID, following DSM-IV	CASH or SCID, following DSM-IV	CASH or Edinburgh Handedness Inventory	2	1.5T Philips Intera and Achieva	T1-weighted three-dimensional fastfield echo (3D-FFE) scans with 160– 180 contiguous coronal slices [256 3 256 matrix, echo time (TE)=4.6 ms, repetition time (TR)=30 ms, flip angle=30 degrees, 1x1x1.2 mm3 voxels, field of view [FOV] = 256 mm/ 70%].	Coronal	v5.1.0	Linux centOS 4_x86_64-stable-pub
**UNIBA**	DSM-IV	SCID	Edinburgh Handedness Inventory	1	3T General Electric Signa	MPRAGE imaging sequence. Flip angle: 6 degrees. TE: 3 ms. TR 25 ms. Field of view: 256. Image dimensions: 256x256x124 voxels. Voxel size: 1x1x1.3 mm.	Axial	v7.1.0	Linux Xubuntu 18.04 LTS
**UNIMAAS**	Diagnosis was confirmed using the CASH by trained clinicians. Psychotic symptom severity was assessed using the PANSS.	Controls were age matched and they were found through advertisement in newspapers and social media platforms. Diagnosis was ruled-out through the CASH by trained clinicians	Edinburgh Handedness Inventory	1	3T Philips Ingenia	32-channel head sense coil. MPRAGE: 180 slices, voxel size 1 × 1 × 1 mm, TR: 7.0 ms, TE: 3.2 ms	Sagittal	v5.4	Linux
**UPENN**	SCID	SCID	Edinburgh Handedness Inventory	2	3T Siemens TIM Trio	MPRAGE, TR=1810 ms, TE= 3.51 ms, TI=1100 ms, flip angle 9, FOV= 240 x 180 mm, matrix= 256 × 192, resolution = 0.9 x 0.9 mm, slices = 160, slice/skip thickness = 1 mm/0 mm.	Axial	v5.3.0	Linux RedHat Enterprise 5
**Zurich**	Diagnosis of schizophrenia was confirmed using a structured Mini-International Neuropsychiatric Interview (MINI) for DSM-IV.	We excluded patients with any other DSM-IV Axis I disorder (in particular, current substance use disorder and major depressive disorder), those medicated with lorazepam at a dose higher than 1 mg, those with florid psychotic symptoms (i.e., any positive subscale item score higher than 4 on the Positive and Negative Syndrome Scale [PANSS]) and those with extrapyramidal side effects (i.e., a total score higher than 2 on the Modified Simpson–Angus Scale [MSAS]). Healthy controls were screened for any neuropsychiatric disorders using the structured Mini-International Neuropsychiatric Interview to ensure that they had no previous or present psychiatric illness. Both patients and healthy controls were required to have a normal physical and neurologic status and no history of major head injury or neurologic disorder.	Self-report	1	3T Philips	3D T1-weighted images were acquired with an ultra fast gradient echo T1-weighted sequence(TR=8.4ms, TE=3.8ms, flip angle=8°) in 160 sagittal plan slices (1mm slice thickness, no slice gap) of 240×240mm2 resulting in 1x1x1mm3voxels.	Sagittal	v6.0.0	Linux

Table S3A. Mean cortical thickness AI direction per dataset compared to sample size weighted grand mean across datasets

Region	AMC	ASRB	CAMH	CASSI	CIAM	CLING	COBRE	EdinburghEHRS	EdinburghFunc	EdinburghSFMH	EONCKS	ESO	FBIRN	FIDMAG	FOR2107 Marburg	FOR2107 Muenster	Frankfurt	GAP	GIPSI	GROUP	HMS	HUBIN	Huilong	IGP	IMH	JBNU	KaSP	Madrid	MCIC	MPRC	OLIN	Osaka	Oxford	PAFIP	RomeSL	RSCZ	SCORE	SNUH	SWIFT	TOP	UCISZ	UMCU	UNIBA	UNIMAAS	UPENN	Zurich	Datasets with + direction	Datasets with - direction	Weighted grand-mean AI
Caudal anterior cingulate cortex	+	+	+	+	+	+	+	-	+	+	+	+	+	+	+	+	+	+	+	+	+	+	+	-	+	+	+	+	-	+	+	+	+	+	-	+	+	+	+	+	+	+	-	+	+	+	41	5	0.033
Isthmus cingulate cortex	+	+	+	+	+	+	+	+	-	+	+	+	+	+	+	+	-	+	+	+	+	+	+	+	+	+	-	+	+	+	+	+	+	-	-	+	+	+	+	+	-	+	+	-	+	+	39	7	0.018
Rostral middle frontal gyrus	-	-	+	-	+	+	+	-	+	+	+	+	+	+	+	+	+	-	+	+	+	+	-	-	-	+	+	+	+	+	+	+	+	-	+	-	+	-	+	+	-	-	+	-	+	-	31	15	0.013
Frontal pole	-	+	+	+	+	+	+	+	+	+	-	+	+	+	+	+	-	-	-	+	+	+	-	-	-	+	+	+	+	+	+	+	-	+	-	+	+	-	-	+	-	+	+	+	+	+	33	13	0.012
Lateral orbitofrontal cortex	+	-	+	+	+	+	+	+	+	+	-	+	+	+	+	+	-	-	+	+	-	+	+	-	+	-	+	+	-	+	+	-	-	+	+	+	-	-	+	+	+	+	+	+	-	+	33	13	0.012
Precentral gyrus	-	+	+	+	+	+	+	-	-	+	+	+	+	+	+	+	+	+	+	+	+	+	+	+	+	+	+	+	+	+	+	+	+	+	+	+	+	+	+	+	+	+	+	+	+	+	43	3	0.012
Posterior cingulate cortex	+	+	-	+	+	+	+	+	+	+	+	+	+	-	+	+	-	-	+	+	+	+	+	-	+	+	-	-	+	+	+	+	+	+	-	+	+	+	+	+	-	+	+	+	+	+	37	9	0.012
Postcentral gyrus	+	+	+	+	+	+	+	+	+	+	+	+	+	+	+	+	+	+	+	+	+	+	+	+	+	+	+	+	+	+	+	+	+	+	+	+	+	+	+	+	+	+	+	+	+	+	46	0	0.011
Superior frontal gyrus	-	+	+	-	+	+	+	+	+	+	+	+	+	+	+	+	+	-	+	+	+	-	+	-	-	+	+	+	+	+	+	+	+	-	+	-	+	+	+	+	-	+	+	-	+	+	36	10	0.0091
Medial orbitofrontal cortex	-	+	+	-	+	+	+	+	+	+	+	+	+	+	+	+	+	-	-	+	+	+	-	-	-	-	+	+	-	+	+	+	-	-	+	-	-	+	+	+	-	+	-	-	-	-	28	18	0.0087
Parahippocampal gyrus	+	+	+	+	-	-	+	-	-	-	+	+	-	-	-	+	+	+	+	+	+	+	-	+	+	+	-	+	+	-	+	+	+	+	+	+	-	+	+	+	+	+	+	+	+	+	34	12	0.0077
Pars orbitalis	+	-	-	-	-	+	+	-	-	+	+	+	+	+	+	+	-	-	+	-	-	-	-	-	+	-	+	+	-	+	-	+	-	+	+	+	+	-	+	+	-	+	+	+	-	+	26	20	0.0060
Insula	-	+	+	-	+	+	+	+	+	+	+	-	+	-	+	+	-	-	+	+	+	+	+	+	-	-	+	+	+	+	+	+	+	-	+	-	-	+	-	-	+	+	+	+	+	-	32	14	0.0057
Caudal middle frontal gyrus	-	-	+	+	+	+	+	+	+	-	+	+	+	-	+	+	-	-	+	-	-	+	+	+	-	-	+	+	+	+	+	+	-	-	+	-	+	+	+	+	+	+	+	+	-	-	31	15	0.0053
Rostral anterior cingulate cortex	-	-	-	-	+	+	+	-	-	+	+	+	+	-	+	+	-	-	-	+	-	-	+	-	-	-	+	+	-	+	+	-	-	-	-	+	+	+	+	+	-	+	-	-	-	-	21	25	0.0047
Superior parietal cortex	+	+	-	+	+	-	+	+	-	+	-	+	+	+	+	+	-	+	+	-	+	+	+	+	+	+	-	+	+	+	-	-	+	+	-	+	+	+	-	+	+	+	+	+	+	+	35	11	0.0041
Overall	-	-	-	-	+	+	+	-	+	+	+	+	+	-	+	+	-	-	+	-	+	-	-	-	+	-	+	+	-	+	+	+	-	-	-	-	+	-	+	+	+	+	+	+	-	-	25	21	0.00080
Pars opercularis	-	-	-	-	+	+	+	+	-	-	-	-	+	-	-	+	-	+	-	-	-	-	-	+	-	-	+	+	+	+	-	+	-	-	+	-	+	-	+	-	-	+	+	+	-	-	19	27	-0.0028
Supramarginal gyrus	-	+	-	+	+	-	-	+	-	-	-	-	-	-	-	-	-	+	-	-	-	-	-	+	+	-	-	+	-	-	-	+	-	+	+	-	+	+	-	-	+	-	+	+	-	-	16	30	-0.0030
Pars triangularis	-	-	-	-	+	+	+	-	+	-	-	+	+	-	+	-	-	-	+	-	-	-	-	-	+	-	+	-	-	+	-	-	-	-	-	-	+	-	+	+	+	-	-	-	-	-	15	31	-0.0033
Fusiform gyrus	+	+	-	-	-	-	-	+	-	-	+	-	-	+	-	+	-	+	+	-	+	-	-	+	+	+	-	+	-	-	-	+	-	+	-	+	-	-	-	-	+	+	-	-	-	-	18	28	-0.0036
Precuneus	+	+	-	+	-	-	-	-	+	-	-	-	-	-	-	-	-	-	+	-	-	-	-	+	+	-	-	-	-	-	-	-	-	+	-	+	-	-	+	-	+	+	-	+	-	+	14	32	-0.0048
Pericalcarine cortex	+	-	+	-	-	+	-	-	+	-	+	-	-	-	-	+	-	+	-	+	-	-	+	-	-	-	+	+	-	-	+	-	-	-	-	-	-	-	+	-	-	+	+	-	-	-	15	31	-0.0057
Paracentral lobule	+	-	-	-	-	-	-	+	-	-	+	-	-	-	-	-	+	-	+	-	-	-	-	+	+	-	-	-	-	-	-	-	-	-	-	+	-	-	-	-	+	+	-	-	-	+	11	35	-0.0058
Inferior temporal gyrus	-	-	-	+	-	-	-	+	+	-	+	-	-	-	-	-	-	+	-	-	-	-	-	+	-	+	+	+	-	-	-	+	+	-	+	-	-	-	-	-	-	+	+	+	-	-	15	31	-0.0083
Inferior parietal cortex	-	-	-	+	-	-	-	-	-	-	-	-	-	-	-	-	-	-	-	-	+	-	+	-	+	+	-	-	-	-	-	+	+	+	-	+	-	+	-	-	+	-	+	+	+	-	14	32	-0.0088
Middle temporal gyrus	-	-	-	-	-	+	-	-	-	-	-	-	-	-	-	-	-	+	-	-	-	-	-	-	-	-	+	-	-	-	-	-	-	-	+	-	-	-	+	-	-	-	+	+	-	-	7	39	-0.011
Superior temporal gyrus	-	-	-	-	-	+	-	-	+	-	+	-	-	-	-	-	-	-	-	-	-	-	-	-	-	-	+	-	-	-	-	-	-	-	-	-	+	-	+	-	-	-	-	-	-	-	6	40	-0.012
Cuneus	+	-	+	-	+	-	-	-	-	-	-	-	-	-	-	-	-	-	-	-	-	-	-	+	+	-	-	+	-	-	+	-	-	-	-	-	-	-	-	-	+	+	+	+	-	-	11	35	-0.012
Transverse temporal gyrus	+	-	-	-	-	-	-	+	-	-	+	-	-	-	+	-	-	-	-	-	+	-	-	-	-	-	-	-	-	-	-	-	-	-	-	-	-	-	-	-	-	-	-	+	+	-	7	39	-0.015
Lingual gyrus	-	-	-	+	-	-	-	-	-	-	-	-	-	-	-	-	-	-	-	-	-	-	-	-	-	-	-	+	-	-	-	-	-	-	-	-	-	-	+	-	-	+	-	+	-	-	5	41	-0.021
Lateral occipital cortex	-	-	-	-	-	-	-	-	-	-	-	-	-	-	-	-	-	-	-	-	+	-	-	-	-	-	-	-	-	-	-	-	-	-	-	-	-	-	-	-	+	-	-	-	-	-	2	44	-0.023
Temporal pole	-	-	-	-	-	-	-	-	+	-	+	-	-	-	-	-	-	-	-	-	-	-	-	+	-	-	+	+	-	-	-	-	-	-	-	-	-	-	-	-	-	-	-	-	-	-	5	41	-0.030
Banks of superior temporal sulcus	-	-	-	-	-	-	-	-	-	-	-	-	-	-	-	-	-	-	-	-	-	-	-	-	-	-	-	-	-	-	-	-	-	-	-	-	-	-	-	-	-	-	-	-	-	-	0	46	-0.037
Entorhinal cortex	-	-	-	-	-	-	-	-	+	-	-	-	-	-	-	-	-	-	-	-	-	-	-	-	-	-	-	+	-	-	-	-	-	-	-	-	-	-	-	-	-	-	-	-	-	-	2	44	-0.038

Table S3B. Mean cortical surface area AI direction per dataset compared to sample size weighted grand mean across datasets.

Region	AMC	ASRB	CAMH	CASSI	CIAM	CLING	COBRE	EdinburghEHRS	EdinburghFunc	EdinburghSFMH	EONCKS	ESO	FBIRN	FIDMAG	FOR2107 Marburg	FOR2107 Muenster	Frankfurt	GAP	GIPSI	GROUP	HMS	HUBIN	Huilong	IGP	IMH	JBNU	KaSP	Madrid	MCIC	MPRC	OLIN	Osaka	Oxford	PAFIP	RomeSL	RSCZ	SCORE	SNUH	SWIFT	TOP	UCISZ	UMCU	UNIBA	UNIMAAS	UPENN	Zurich	Datasets with + direction	Datasets with – direction	Weighted grand-mean AI
Transverse temporal gyrus	+	+	+	+	+	+	+	+	+	+	+	+	+	+	+	+	+	+	+	+	+	+	+	+	+	+	+	+	+	+	+	+	+	+	+	+	+	+	+	+	+	+	+	+	+	+	46	0	0.29
Rostral anterior cingulate cortex	+	+	+	+	+	+	+	+	+	+	+	+	+	+	+	+	+	+	+	+	+	+	+	+	+	+	+	+	+	+	+	+	+	+	+	+	+	+	+	+	+	+	+	+	+	+	46	0	0.22
Pars opercularis	+	+	+	+	+	+	+	+	+	+	+	+	+	+	+	+	+	+	+	+	+	+	+	+	+	+	+	+	+	+	+	+	+	+	+	+	+	+	+	+	+	+	+	+	+	+	46	0	0.17
Entorhinal cortex	+	+	+	+	+	+	+	+	+	+	+	+	+	+	+	+	+	+	+	+	+	+	+	+	+	+	+	+	+	+	+	+	+	+	+	+	+	+	+	+	+	+	+	+	+	+	46	0	0.16
Temporal pole	+	+	+	+	+	+	+	+	+	+	+	+	+	+	+	+	+	+	+	+	+	+	+	+	+	+	+	+	+	+	+	+	+	+	+	+	+	+	+	+	+	+	+	+	+	+	46	0	0.12
Banks of superior temporal sulcus	+	+	+	+	+	+	+	+	+	+	+	+	+	+	+	+	+	+	+	+	+	+	+	+	+	+	+	+	+	+	+	+	+	+	+	+	+	+	+	+	+	+	+	+	+	+	46	0	0.090
Caudal middle frontal gyrus	+	+	+	+	+	+	+	+	+	+	+	+	+	+	+	+	+	+	+	+	+	+	+	+	+	+	+	+	+	+	+	+	+	+	+	+	+	+	+	+	+	+	+	+	+	+	46	0	0.077
Isthmus cingulate cortex	+	+	+	+	+	+	+	+	+	+	+	+	+	+	+	+	+	+	+	+	+	+	+	+	+	+	+	+	+	+	+	+	+	+	+	+	+	+	+	+	+	+	+	+	+	+	46	0	0.067
Supramarginal gyrus	+	+	+	+	-	+	+	+	+	+	+	+	+	+	+	+	+	+	+	+	+	+	+	+	+	+	+	+	+	+	+	+	+	+	+	+	+	+	+	+	+	+	+	+	+	+	45	1	0.061
Superior temporal gyrus	+	+	+	+	+	+	+	+	+	+	+	+	+	+	+	+	+	+	+	+	+	+	+	+	+	+	+	+	+	+	+	+	+	+	+	+	+	+	+	+	+	+	+	+	+	+	46	0	0.049
Inferior temporal gyrus	+	+	+	+	+	+	+	+	+	+	+	+	+	+	+	+	+	+	+	+	+	+	+	+	+	+	+	+	+	+	+	+	+	+	+	+	-	+	+	+	+	+	+	+	+	+	45	1	0.048
Parahippocampal gyrus	+	+	+	+	+	+	+	+	+	+	+	-	+	+	+	+	+	+	-	+	+	+	+	+	+	+	+	+	+	+	+	+	+	+	+	+	+	+	+	+	+	+	+	+	+	+	44	2	0.041
Postcentral gyrus	+	+	+	+	+	+	+	+	+	+	+	+	+	+	+	+	+	+	+	+	+	+	+	+	+	+	+	+	+	+	+	+	+	+	+	+	+	+	+	+	+	+	+	+	+	+	46	0	0.039
Superior frontal gyrus	+	+	+	+	+	+	+	+	+	+	+	+	+	+	+	+	+	+	+	+	+	+	+	+	+	+	+	+	+	+	+	+	+	+	+	+	+	+	+	+	+	+	+	+	+	+	46	0	0.031
Fusiform gyrus	+	+	+	+	+	+	+	+	+	+	+	-	+	+	+	+	+	+	-	+	+	+	+	+	+	+	+	+	+	+	+	+	+	+	+	+	-	+	+	+	+	+	+	+	+	+	43	3	0.029
Lateral occipital cortex	+	+	+	+	+	+	+	+	+	+	+	+	+	+	+	+	+	+	+	+	+	+	+	+	+	+	+	+	+	+	+	+	+	+	+	+	+	+	+	+	+	+	+	+	+	+	46	0	0.028
Medial orbitofrontal cortex	-	+	+	-	-	+	-	+	+	-	+	-	+	+	-	+	+	+	-	-	+	+	-	+	-	-	+	+	+	-	-	+	-	-	+	-	+	-	-	-	+	+	-	-	+	-	23	23	0.0098
Lateral orbitofrontal cortex	+	+	-	-	+	+	+	+	-	+	-	+	+	-	+	+	+	-	-	-	+	+	-	-	-	-	-	-	+	+	+	-	+	-	+	+	+	+	-	+	+	-	+	-	+	-	26	20	0.0083
Superior parietal cortex	+	-	-	+	-	-	+	+	-	-	-	+	+	-	+	+	+	-	+	+	-	-	+	-	+	+	-	+	+	-	-	+	+	+	+	-	+	-	+	-	+	+	+	+	-	+	27	19	0.0033
Overall	-	-	-	-	-	-	-	-	-	-	-	-	-	-	-	-	+	-	-	NA	-	-	-	-	-	-	-	+	-	-	-	-	-	+	+	-	-	-	-	-	+	-	-	-	-	-	5	40	-0.0036
Precentral gyrus	-	-	-	+	-	-	-	-	-	-	+	-	-	-	-	-	-	-	-	-	-	-	+	-	+	+	-	-	-	-	-	+	-	+	-	-	-	+	-	-	-	-	-	-	-	-	8	38	-0.0067
Posterior cingulate cortex	+	-	-	-	-	-	-	-	+	+	+	+	-	-	-	-	+	-	-	+	-	-	-	-	-	-	-	-	-	-	-	-	-	-	+	-	+	-	-	-	+	-	-	-	-	-	10	36	-0.013
Lingual gyrus	+	-	+	-	-	-	-	+	-	-	-	-	-	+	-	-	+	+	+	-	+	-	-	+	-	-	-	-	+	+	-	-	+	-	-	+	+	-	-	-	-	-	-	-	+	-	15	31	-0.013
Insula	-	-	-	+	+	-	-	-	+	-	+	+	-	-	-	-	+	-	-	+	-	-	-	+	+	+	-	-	-	-	+	-	-	+	+	+	+	-	+	-	-	-	-	+	-	+	18	28	-0.015
Rostral middle frontal gyrus	-	-	-	-	-	-	-	-	-	-	-	-	-	-	-	-	-	-	-	-	-	-	-	-	-	-	-	-	-	-	-	-	-	-	-	-	-	-	-	-	-	-	-	-	-	-	0	46	-0.034
Precuneus	-	-	-	-	-	-	-	-	-	-	-	-	-	-	-	-	-	-	-	-	-	-	-	-	-	-	-	-	-	-	-	-	-	-	-	-	-	-	-	-	-	-	-	-	-	-	0	46	-0.043
Cuneus	-	-	-	-	-	-	-	-	-	-	-	-	-	-	-	-	-	-	-	-	-	-	-	-	-	-	-	-	-	-	-	-	-	-	-	-	-	-	-	-	-	-	-	-	-	-	0	46	-0.045
Middle temporal gyrus	-	-	-	-	-	-	-	-	-	-	-	-	-	-	-	-	-	-	-	-	-	-	-	-	-	-	-	-	-	-	-	-	-	-	-	-	-	-	-	-	-	-	-	-	-	-	0	46	-0.095
Pericalcarine cortex	-	-	-	-	-	-	-	-	-	-	-	-	-	-	-	-	-	-	-	-	-	-	-	-	-	-	-	-	-	-	-	-	-	-	-	-	-	-	-	-	-	-	-	-	-	-	0	46	-0.10
Paracentral lobule	-	-	-	-	-	-	-	-	-	-	-	-	-	-	-	-	-	-	-	-	-	-	-	-	-	-	-	-	-	-	-	-	-	-	-	-	-	-	-	-	-	-	-	-	-	-	0	46	-0.12
Pars triangularis	-	-	-	-	-	-	-	-	-	-	-	-	-	-	-	-	-	-	-	-	-	-	-	-	-	-	-	-	-	-	-	-	-	-	-	-	-	-	-	-	-	-	-	-	-	-	0	46	-0.15
Inferior parietal cortex	-	-	-	-	-	-	-	-	-	-	-	-	-	-	-	-	-	-	-	-	-	-	-	-	-	-	-	-	-	-	-	-	-	-	-	-	-	-	-	-	-	-	-	-	-	-	0	46	-0.16
Caudal anterior cingulate cortex	-	-	-	-	-	-	-	-	-	-	-	-	-	-	-	-	-	-	-	-	-	-	-	-	-	-	-	-	-	-	-	-	-	-	-	-	-	-	-	-	-	-	-	-	-	-	0	46	-0.17
Pars orbitalis	-	-	-	-	-	-	-	-	-	-	-	-	-	-	-	-	-	-	-	-	-	-	-	-	-	-	-	-	-	-	-	-	-	-	-	-	-	-	-	-	-	-	-	-	-	-	0	46	-0.21
Frontal pole	-	-	-	-	-	-	-	-	-	-	-	-	-	-	-	-	-	-	-	-	-	-	-	-	-	-	-	-	-	-	-	-	-	-	-	-	-	-	-	-	-	-	-	-	-	-	0	46	-0.29

Table S3C. Mean subcortical volume AI direction per dataset compared to sample size weighted grand mean across datasets.

Region	AMC	ASRB	CAMH	CASSI	CIAM	CLING	COBRE	EdinburghEHRS	EdinburghFunc	EdinburghSFMH	EONCKS	ESO	FBIRN	FIDMAG	FOR2107 Marburg	FOR2107 Muenster	Frankfurt	GAP	GIPSI	GROUP	HMS	HUBIN	Huilong	IGP	IMH	JBNU	KaSP	Madrid	MCIC	MPRC	OLIN	Osaka	Oxford	PAFIP	RomeSL	RSCZ	SCORE	SNUH	SWIFT	TOP	UCISZ	UMCU	UNIBA	UNIMAAS	UPENN	Zurich	Datasets with + direction	Datasets with - direction	Weighted grand-mean AI
Lateral Ventricles	+	+	+	+	+	+	+	+	+	+	+	+	+	+	+	+	+	+	+	+	+	+	+	+	+	+	+	+	+	+	+	+	+	+	+	+	NA	+	+	+	+	+	+	+	+	+	45	0	0.095
Thalamus	+	-	+	+	+	-	+	+	-	+	+	+	-	+	+	+	+	+	+	+	-	+	NA	+	+	+	+	+	+	+	-	+	+	+	+	+	+	+	+	+	+	+	+	+	+	+	39	6	0.049
Putamen	+	+	+	-	+	+	+	-	+	+	+	+	+	+	+	+	-	+	+	+	+	+	+	+	-	-	+	+	+	+	+	+	+	-	+	+	+	+	-	+	+	+	+	-	+	+	38	8	0.033
Pallidum	+	+	+	-	+	+	+	+	+	+	-	+	+	+	-	-	-	-	-	-	+	+	-	-	+	-	-	+	+	-	+	-	+	+	-	+	-	-	+	-	-	+	-	-	+	+	25	21	0.030
Caudate Nucelus	-	+	-	-	-	-	-	-	-	+	-	+	-	+	+	-	-	-	-	-	-	-	NA	-	-	-	-	+	+	-	-	+	+	-	-	-	+	+	-	-	-	-	-	-	+	-	12	33	-0.011
Hippocampus	-	-	-	-	-	-	-	+	-	-	-	-	-	-	-	-	-	-	-	-	-	-	-	-	-	-	+	-	-	-	-	-	-	-	-	-	-	-	-	-	-	+	-	-	-	-	3	43	-0.019
Accumbens	-	+	+	-	+	-	-	+	-	-	+	-	-	+	-	-	-	-	+	-	+	-	-	+	+	-	-	+	-	+	-	+	+	-	-	-	-	-	-	+	-	+	-	-	-	-	16	30	-0.049
Amygdala	-	-	-	-	-	-	+	-	-	-	-	+	-	-	-	-	-	-	-	-	-	-	-	-	-	-	-	-	-	-	-	-	-	-	-	-	-	-	-	-	-	-	-	-	-	-	2	44	-0.056

Table S4A. Weighted mean thickness and surface area AIs for cortical regions.

	Cortical Thickness						Cortical Surface Area					
	Unaffected individuals			Schizophrenia individuals			Unaffected individuals			Schizophrenia individuals		
**Region**	**AI (mean)**	**sd (pooled)**	**N**	**AI (mean)**	**sd (pooled)**	**N**	**AI (mean)**	**sd (pooled)**	**N**	**AI (mean)**	**sd (pooled)**	**N**
Banks of superior temporal sulcus	-0.0379	0.0757	5670	-0.0365	0.0764	4764	0.0888	0.1573	5667	0.0911	0.162	4762
Caudal anterior cingulate cortex	0.0337	0.1027	5849	0.0312	0.1096	4936	-0.1723	0.2394	5846	-0.1654	0.2428	4934
Caudal middle frontal gyrus	0.0066	0.0484	5844	0.0037	0.0495	4913	0.0767	0.1477	5840	0.0765	0.1506	4908
Cuneus	-0.0134	0.0634	5785	-0.011	0.0636	4841	-0.0423	0.1244	5781	-0.049	0.1286	4839
Entorhinal cortex	-0.0384	0.1086	5564	-0.0377	0.1095	4682	0.1636	0.2154	5560	0.149	0.2155	4680
Fusiform gyrus	-0.0049	0.0444	5802	-0.002	0.0442	4837	0.0302	0.1009	5796	0.0276	0.1007	4836
Inferior parietal cortex	-0.0091	0.0406	5695	-0.0085	0.0412	4697	-0.1622	0.1025	5691	-0.164	0.1042	4699
Inferior temporal gyrus	-0.0083	0.0534	5761	-0.0082	0.0536	4814	0.049	0.1113	5758	0.0466	0.1122	4812
Isthmus cingulate cortex	0.0201	0.0770	5875	0.0164	0.0784	4949	0.0656	0.1431	5871	0.0676	0.1444	4948
Lateral occipital cortex	-0.0228	0.0444	5819	-0.0223	0.0441	4838	0.0273	0.0953	5814	0.0287	0.0979	4836
Lateral orbitofrontal cortex	0.0123	0.0545	5870	0.0112	0.0541	4936	0.011	0.0767	5865	0.0051	0.0794	4937
Lingual gyrus	-0.0219	0.0510	5843	-0.0201	0.0503	4932	-0.0115	0.1004	5840	-0.0144	0.1033	4928
Medial orbitofrontal cortex	0.0107	0.0673	5825	0.0062	0.0668	4906	0.0085	0.1133	5824	0.0113	0.1143	4904
Middle temporal gyrus	-0.0080	0.0481	5673	-0.0148	0.048	4727	-0.0957	0.0888	5668	-0.0943	0.0896	4724
Parahippocampal gyrus	0.0074	0.0910	5849	0.0081	0.0904	4906	0.0404	0.1266	5845	0.0419	0.1257	4899
Paracentral lobule	-0.0060	0.0515	5871	-0.0055	0.0522	4956	-0.124	0.1239	5868	-0.1227	0.1232	4953
Pars opercularis of inferior frontal gyrus	-0.0029	0.0555	5824	-0.0026	0.0565	4875	0.1718	0.1587	5818	0.1722	0.1565	4873
Pars orbitalis of inferior frontal gyrus	0.0068	0.0841	5838	0.005	0.0843	4904	-0.2089	0.1197	5835	-0.2081	0.1211	4904
Pars triangularis of inferior frontal gyrus	-0.0028	0.0608	5807	-0.0039	0.0611	4861	-0.1508	0.1495	5801	-0.1498	0.1498	4860
Pericalcarine cortex	-0.0062	0.0753	5833	-0.0051	0.0758	4945	-0.0984	0.1158	5831	-0.1018	0.119	4941
Postcentral gyrus	0.0110	0.0453	5770	0.0119	0.044	4836	0.0393	0.0871	5766	0.0387	0.0849	4835
Posterior cingulate cortex	0.0120	0.0612	5880	0.0109	0.0629	4959	-0.0143	0.15	5873	-0.0104	0.152	4957
Precentral gyrus	0.0128	0.0380	5807	0.0107	0.0405	4851	-0.0073	0.0751	5800	-0.006	0.0775	4849
Precuneus	-0.0059	0.0387	5864	-0.0035	0.0385	4911	-0.0431	0.0764	5862	-0.0424	0.0771	4912
Rostral anterior cingulate cortex	0.0116	0.0863	5811	-0.0035	0.0923	4894	0.2083	0.2027	5807	0.2273	0.209	4892
Rostral middle frontal gyrus	0.0154	0.0404	5811	0.0095	0.0393	4849	-0.0333	0.0806	5808	-0.0353	0.0839	4846
Superior frontal gyrus	0.0109	0.0287	5833	0.0069	0.029	4897	0.03	0.0673	5830	0.0317	0.0762	4894
Superior parietal cortex	0.0032	0.0338	5766	0.0053	0.0345	4777	0.0033	0.0852	5763	0.0033	0.0852	4777
Superior temporal gyrus	-0.0111	0.0425	5596	-0.013	0.0433	4665	0.0474	0.0774	5591	0.0518	0.0791	4668
Supramarginal gyrus	-0.0036	0.0439	5613	-0.0023	0.0454	4649	0.0568	0.1194	5608	0.0663	0.1241	4650
Frontal pole	0.0143	0.1185	5880	0.0102	0.118	4975	-0.2887	0.1818	5878	-0.2828	0.181	4970
Temporal pole	-0.0316	0.0960	5765	-0.0271	0.0964	4808	0.1214	0.1502	5763	0.1138	0.1481	4808
Transverse temporal gyrus	-0.0150	0.0944	5880	-0.0159	0.0944	4966	0.2866	0.1525	5873	0.2945	0.1537	4963
Insula	0.0065	0.0464	5807	0.0048	0.0478	4963	-0.0162	0.0831	5801	-0.0136	0.0862	4961
Overall	0.0013	0.0136	5915	0.0001	0.0137	4988	-0.0039	0.0142	5730	-0.0033	0.0156	4899

AI: Asymmetry Index; sd: standard deviation; N: Sample size.

Table S4B. Weighted mean volume AIs for subcortical regions.

	Subcortical Volume					
	Unaffected individuals			Schizophrenia individuals		
**Region**	**AI (mean)**	**sd (pooled)**	**N**	**AI (mean)**	**sd (pooled)**	**N**
Lateral Ventricles	0.093	0.2702	5770	0.0981	0.2449	4872
Thalamus	0.0507	0.064	5718	0.0464	0.0643	4788
Caudate Nucelus	-0.0101	0.0603	5709	-0.0123	0.0595	4791
Putamen	0.0363	0.0629	5758	0.0284	0.063	5035
Pallidum	0.0277	0.1134	5687	0.033	0.1086	5007
Hippocampus	-0.019	0.0693	5771	-0.0183	0.0696	5015
Amygdala	-0.0487	0.1068	5784	-0.0633	0.1023	5033
Accumbens	-0.0441	0.1578	5775	-0.0553	0.1732	5009

AI: Asymmetry Index; sd: standard deviation; N: Sample size.

Table S5A. Meta-analysis results of case-control differences for cortical thickness AIs.

Region	Cohen's d	se	95% CI	z	p	p_{FDR}	Q	p_Q	I^2	df	N SZ	N CTR
Banks of superior temporal sulcus	0.0060	0.020	[-0.033, 0.045]	0.30	7.62E-01	9.84E-01	36	8.00E-01	0	44	4721	5670
Caudal anterior cingulate cortex	0.015	0.022	[-0.029, 0.058]	0.65	5.14E-01	9.69E-01	53.4	1.56E-01	16.5	44	4893	5849
Caudal middle frontal gyrus	-0.056	0.030	[-0.115, 0.002]	-1.89	5.81E-02	3.93E-01	85.3	1.89E-04	49.6	44	4870	5844
Cuneus	0.00030	0.020	[-0.038, 0.039]	0.01	9.89E-01	9.89E-01	35.9	8.03E-01	0	44	4798	5785
Entorhinal cortex	-0.0082	0.021	[-0.048, 0.032]	-0.40	6.91E-01	9.69E-01	39.4	6.69E-01	3.3	44	4639	5564
Fusiform gyrus	0.041	0.026	[-0.011, 0.092]	1.54	1.24E-01	6.19E-01	67.8	1.22E-02	36	44	4794	5802
Inferior parietal cortex	-0.060	0.026	[-0.111, -0.009]	-2.32	2.01E-02	2.35E-01	67.5	1.29E-02	32.7	44	4654	5695
Inferior temporal gyrus	-0.0011	0.027	[-0.053, 0.051]	-0.04	9.68E-01	9.89E-01	72.8	4.11E-03	36.7	44	4771	5761
Isthmus cingulate cortex	-0.00060	0.019	[-0.039, 0.037]	-0.03	9.77E-01	9.89E-01	33.7	8.71E-01	0	44	4906	5875
Lateral occipital cortex	-0.014	0.032	[-0.076, 0.049]	-0.43	6.69E-01	9.69E-01	95	1.31E-05	55.2	44	4795	5819
Lateral orbitofrontal cortex	-0.012	0.033	[-0.077, 0.054]	-0.36	7.20E-01	9.69E-01	112.2	7.04E-08	60.2	44	4893	5870
Lingual gyrus	0.012	0.028	[-0.044, 0.067]	0.42	6.77E-01	9.69E-01	80	7.38E-04	44.3	44	4889	5843
Medial orbitofrontal cortex	0.0085	0.031	[-0.053, 0.07]	0.27	7.87E-01	9.84E-01	96.5	8.42E-06	54.4	44	4863	5825
Middle temporal gyrus	-0.074	0.025	[-0.123, -0.026]	-2.99	2.75E-03	4.82E-02	65.9	1.80E-02	27.7	44	4684	5673
Parahippocampal gyrus	-0.029	0.025	[-0.079, 0.021]	-1.15	2.49E-01	7.26E-01	66.3	1.64E-02	32.9	44	4863	5849
Paracentral lobule	-0.0022	0.019	[-0.04, 0.036]	-0.11	9.11E-01	9.89E-01	39.5	6.65E-01	0	44	4913	5871
Pars opercularis of inferior frontal gyrus	0.016	0.029	[-0.039, 0.072]	0.57	5.66E-01	9.69E-01	80.8	6.08E-04	45.1	44	4832	5824
Pars orbitalis of inferior frontal gyrus	-0.021	0.029	[-0.078, 0.037]	-0.71	4.77E-01	9.69E-01	81.6	4.88E-04	47.9	44	4861	5838
Pars triangularis of inferior frontal gyrus	0.014	0.029	[-0.044, 0.071]	0.47	6.41E-01	9.69E-01	85.3	1.90E-04	47.8	44	4818	5807
Pericalcarine cortex	0.027	0.020	[-0.013, 0.066]	1.33	1.84E-01	6.62E-01	46.4	3.72E-01	3.4	44	4902	5833
Postcentral gyrus	0.010	0.026	[-0.041, 0.06]	0.37	7.12E-01	9.69E-01	66.4	1.62E-02	33.7	44	4793	5770
Posterior cingulate cortex	0.023	0.025	[-0.025, 0.071]	0.94	3.49E-01	8.30E-01	55.7	1.11E-01	29.5	44	4916	5880
Precentral gyrus	-0.037	0.028	[-0.092, 0.018]	-1.31	1.89E-01	6.62E-01	76.7	1.63E-03	43.1	44	4808	5807
Precuneus	-0.0054	0.028	[-0.059, 0.049]	-0.19	8.45E-01	9.89E-01	75.6	2.15E-03	41.9	44	4868	5864
Rostral anterior cingulate cortex	-0.083	0.026	[-0.134, -0.032]	-3.21	1.34E-03	4.69E-02	67.6	1.27E-02	34.5	44	4851	5811
Rostral middle frontal gyrus	-0.044	0.033	[-0.11, 0.021]	-1.33	1.84E-01	6.62E-01	106.1	4.79E-07	59.4	44	4806	5811
Superior frontal gyrus	-0.049	0.027	[-0.101, 0.003]	-1.83	6.73E-02	3.93E-01	71.8	5.16E-03	38.3	44	4854	5833
Superior parietal cortex	0.012	0.026	[-0.039, 0.063]	0.46	6.45E-01	9.69E-01	68.6	1.02E-02	33.6	44	4734	5766
Superior temporal gyrus	-0.0029	0.028	[-0.058, 0.052]	-0.10	9.18E-01	9.89E-01	77.1	1.48E-03	40.9	44	4622	5596
Supramarginal gyrus	-0.032	0.034	[-0.099, 0.036]	-0.92	3.56E-01	8.30E-01	99.9	3.15E-06	60.3	44	4606	5613
Frontal pole	-0.018	0.019	[-0.056, 0.02]	-0.92	3.55E-01	8.30E-01	49.9	2.51E-01	0	44	4932	5880
Temporal pole	-0.012	0.020	[-0.05, 0.027]	-0.60	5.48E-01	9.69E-01	39.9	6.47E-01	0	44	4765	5765
Transverse temporal gyrus	0.026	0.022	[-0.016, 0.068]	1.21	2.27E-01	7.24E-01	56.8	9.40E-02	13.1	44	4923	5880
Insula	-0.0013	0.024	[-0.049, 0.046]	-0.05	9.58E-01	9.89E-01	55.5	1.14E-01	27	44	4920	5807
Overall	-0.053	0.027	[-0.107, 0.001]	-1.92	5.51E-02	3.93E-01	72.6	4.28E-03	42.2	44	4945	5915

Cohen's d: meta-analysis Cohen's d effect size; se: Standard error of Cohen's d effect size; CI: Confidence interval; z: Meta-analysis z-value; p: meta-analysis p-value; p_{FDR}: FDR-corrected p-value; Q: Cochran's Q-statistic of between-study heterogeneity; p_Q: p-value of Cochran's Q-statistic; I^2: Percentage of variation across studies due to heterogeneity; df: Degrees of freedom in meta-analysis; N SZ: Number of individuals affected with schizophrenia; N CTR: Number of unaffected individuals.

Table S5B. Meta-analysis results of case-control differences for cortical surface area AIs.

Region	Cohen's d	se	95% CI	z	p	p_{FDR}	Q	p_Q	I^2	df	N SZ	N CTR
Banks of superior temporal sulcus	-0.014	0.025	[-0.063, 0.036]	-0.53	5.94E-01	9.04E-01	66.5	1.58E-02	30.8	44	4719	5667
Caudal anterior cingulate cortex	0.017	0.026	[-0.033, 0.068]	0.67	5.04E-01	8.27E-01	70.8	6.39E-03	35	44	4892	5846
Caudal middle frontal gyrus	0.028	0.024	[-0.019, 0.075]	1.15	2.48E-01	6.66E-01	56.1	1.04E-01	26.4	44	4865	5840
Cuneus	-0.042	0.020	[-0.081, -0.003]	-2.09	3.67E-02	5.03E-01	50.9	2.20E-01	2.2	44	4796	5781
Entorhinal cortex	-0.039	0.020	[-0.078, 0]	-1.94	5.19E-02	5.03E-01	47.1	3.46E-01	0	44	4637	5560
Fusiform gyrus	0.0062	0.024	[-0.04, 0.053]	0.26	7.94E-01	9.77E-01	60	5.43E-02	23.9	44	4793	5796
Inferior parietal cortex	0.028	0.020	[-0.011, 0.067]	1.42	1.55E-01	5.03E-01	60	5.48E-02	0	44	4656	5691
Inferior temporal gyrus	-0.00030	0.024	[-0.047, 0.046]	-0.01	9.89E-01	9.95E-01	61.6	4.09E-02	22.4	44	4769	5758
Isthmus cingulate cortex	-0.030	0.021	[-0.071, 0.01]	-1.48	1.40E-01	5.03E-01	43.6	4.88E-01	7.5	44	4905	5871
Lateral occipital cortex	0.018	0.026	[-0.033, 0.069]	0.69	4.93E-01	8.27E-01	63.3	2.95E-02	34.6	44	4793	5814
Lateral orbitofrontal cortex	-0.051	0.036	[-0.121, 0.019]	-1.42	1.57E-01	5.03E-01	126	7.81E-10	65.4	44	4894	5865
Lingual gyrus	-0.028	0.019	[-0.066, 0.01]	-1.43	1.54E-01	5.03E-01	55.5	1.14E-01	0	44	4885	5840
Medial orbitofrontal cortex	0.032	0.032	[-0.031, 0.094]	0.99	3.23E-01	7.54E-01	98.6	4.58E-06	55.8	44	4861	5824
Middle temporal gyrus	-0.0027	0.026	[-0.054, 0.048]	-0.1	9.17E-01	9.95E-01	69.4	8.58E-03	32.8	44	4681	5668
Parahippocampal gyrus	0.042	0.020	[0.004, 0.08]	2.14	3.22E-02	5.03E-01	38.4	7.08E-01	0.9	44	4856	5845
Paracentral lobule	0.015	0.020	[-0.025, 0.054]	0.72	4.70E-01	8.27E-01	46.5	3.69E-01	5.1	44	4910	5868
Pars opercularis of inferior frontal gyrus	0.0014	0.019	[-0.037, 0.04]	0.07	9.44E-01	9.95E-01	42.3	5.46E-01	0	44	4830	5818
Pars orbitalis of inferior frontal gyrus	-0.028	0.026	[-0.078, 0.022]	-1.11	2.66E-01	6.66E-01	63.9	2.66E-02	33.2	44	4861	5835
Pars triangularis of inferior frontal gyrus	0.013	0.020	[-0.025, 0.052]	0.69	4.92E-01	8.27E-01	37.8	7.32E-01	0	44	4817	5801
Pericalcarine cortex	-0.044	0.023	[-0.089, 0.001]	-1.9	5.79E-02	5.03E-01	55.5	1.14E-01	21.4	44	4898	5831
Postcentral gyrus	-0.0067	0.021	[-0.047, 0.034]	-0.32	7.49E-01	9.77E-01	48.8	2.85E-01	7.4	44	4792	5766
Posterior cingulate cortex	0.011	0.023	[-0.034, 0.056]	0.48	6.30E-01	9.18E-01	48.9	2.81E-01	19.9	44	4914	5873
Precentral gyrus	0.0047	0.020	[-0.034, 0.043]	0.24	8.09E-01	9.77E-01	43.8	4.79E-01	0	44	4807	5800
Precuneus	0.039	0.024	[-0.008, 0.085]	1.63	1.03E-01	5.03E-01	59.3	6.16E-02	23.9	44	4869	5862
Rostral anterior cingulate cortex	0.033	0.024	[-0.013, 0.08]	1.41	1.58E-01	5.03E-01	65.8	1.81E-02	23.6	44	4849	5807
Rostral middle frontal gyrus	-0.00010	0.023	[-0.045, 0.045]	-0.01	9.95E-01	9.95E-01	63.5	2.88E-02	19.8	44	4803	5808
Superior frontal gyrus	0.016	0.023	[-0.028, 0.061]	0.71	4.76E-01	8.27E-01	58.6	6.99E-02	18.6	44	4851	5830
Superior parietal cortex	-0.010	0.024	[-0.056, 0.037]	-0.41	6.84E-01	9.57E-01	58.3	7.33E-02	22.9	44	4734	5763
Superior temporal gyrus	-0.0038	0.020	[-0.043, 0.035]	-0.19	8.50E-01	9.92E-01	46.5	3.69E-01	0	44	4625	5591
Supramarginal gyrus	0.015	0.023	[-0.031, 0.061]	0.64	5.20E-01	8.27E-01	60.4	5.08E-02	19.6	44	4607	5608
Frontal pole	-0.0057	0.023	[-0.052, 0.04]	-0.24	8.07E-01	9.77E-01	59.9	5.53E-02	23.6	44	4927	5878
Temporal pole	0.0036	0.024	[-0.043, 0.05]	0.15	8.78E-01	9.92E-01	52.6	1.76E-01	22.3	44	4765	5763
Transverse temporal gyrus	0.042	0.024	[-0.005, 0.089]	1.76	7.87E-02	5.03E-01	54.4	1.35E-01	25.9	44	4920	5873
Insula	0.019	0.024	[-0.029, 0.066]	0.76	4.49E-01	8.27E-01	61	4.52E-02	28.1	44	4918	5801
Overall	0.027	0.022	[-0.016, 0.07]	1.23	2.18E-01	6.35E-01	47.4	2.98E-01	14	43	4856	5730

Cohen's d: meta-analysis Cohen's d effect size; se: Standard error of Cohen's d effect size; CI: Confidence interval; z: Meta-analysis z-value; p: meta-analysis p-value; p_{FDR}: FDR-corrected p-value; Q: Cochran's Q-statistic of between-study heterogeneity; p_Q: p-value of Cochran's Q-statistic; I^2: Percentage of variation across studies due to heterogeneity; df: Degrees of freedom in meta-analysis; N SZ: Number of individuals affected with schizophrenia; N CTR: Number of unaffected individuals.

Table S5C. Meta-analysis results of case-control differences for subcortical volume AIs.

Region	Cohen's d	se	95% CI	z	p	p_{FDR}	Q	p_Q	I^2	df	N SZ	N CTR
Lateral Ventricles	0.034	0.021	[-0.007, 0.076]	1.62	1.06E-01	1.69E-01	53.1	1.39E-01	9.8	43	4829	5770
Thalamus	0.046	0.041	[-0.034, 0.125]	1.12	2.63E-01	3.01E-01	145.5	4.58E-13	73	43	4746	5718
Caudate Nucelus	0.033	0.030	[-0.025, 0.091]	1.13	2.60E-01	3.01E-01	88.5	5.49E-05	48.3	43	4748	5709
Putamen	-0.043	0.025	[-0.092, 0.005]	-1.74	8.16E-02	1.63E-01	66.2	1.67E-02	30.4	44	4992	5758
Pallidum	0.070	0.039	[-0.006, 0.146]	1.82	6.92E-02	1.63E-01	152.9	6.09E-14	70.5	44	4964	5687
Hippocampus	-0.0070	0.023	[-0.053, 0.039]	-0.3	7.65E-01	7.65E-01	56.1	1.05E-01	23	44	4973	5771
Amygdala	-0.045	0.024	[-0.093, 0.002]	-1.88	5.98E-02	1.63E-01	62.6	3.37E-02	27.3	44	4990	5784
Accumbens	0.062	0.026	[0.01, 0.113]	2.33	1.97E-02	1.58E-01	74	3.10E-03	37.8	44	4966	5775

Cohen's d: meta-analysis Cohen's d effect size; se: Standard error of Cohen's d effect size; CI: Confidence interval; z: Meta-analysis z-value; p: meta-analysis p-value; p_{FDR}: FDR-corrected p-value; Q: Cochran's Q-statistic of between-study heterogeneity; p_Q: p-value of Cochran's Q-statistic; I^2: Percentage of variation across studies due to heterogeneity; df: Degrees of freedom in meta-analysis; N SZ: Number of individuals affected with schizophrenia; N CTR: Number of unaffected individuals.

Table S6. Analysis of directionality for significant AI alterations that arose in primary case-control analysis.

Hemisphere	Measurement	Region	Cohen's d	se	95% CI	z	p	p_{FDR}	Q	p_Q	I^2	df	N SZ	N CTR
AI	Thickness	Rostral anterior cingulate cortex	-0.083	0.026	[-0.134, -0.032]	-3.21	1.34E-03	4.69E-02	67.6	1.27E-02	34.5	44	4851	5811
Left	Thickness	Rostral anterior cingulate cortex	-0.20	0.042	[-0.278, -0.114]	-4.69	2.71E-06		160.5	3.62E-15	74.6	44	4851	5811
Right	Thickness	Rostral anterior cingulate cortex	-0.094	0.029	[-0.151, -0.036]	-3.19	1.44E-03		83.2	3.28E-04	48.1	44	4851	5811
AI	Thickness	Middle temporal gyrus	-0.074	0.025	[-0.123, -0.026]	-2.99	2.75E-03	4.82E-02	65.9	1.80E-02	27.7	44	4684	5673
Left	Thickness	Middle temporal gyrus	-0.41	0.046	[-0.496, -0.315]	-8.81	1.31E-18		175.5	1.26E-17	78.3	44	4684	5673
Right	Thickness	Middle temporal gyrus	-0.36	0.045	[-0.444, -0.266]	-7.8	5.95E-15		183	7.20E-19	77.9	44	4684	5673

For each region, the primary meta-analysis result is shown (AI), as well as the separate effects on cortical thickness of the left and right hemisphere.

AI: Asymmetry index; Cohen's d: meta-analysis Cohen's d effect size; se: Standard error of Cohen's d effect size; CI: Confidence interval; z: Meta-analysis z-value; p: meta-analysis p-value; p_{FDR}: FDR-corrected p-value; Q: Cochran's Q-statistic of between-study heterogeneity; p_Q: p-value of Cochran's Q-statistic; I^2: Percentage of variation across studies due to heterogeneity; df: Degrees of freedom in meta-analysis; N SZ: Number of individuals affected with schizophrenia; N CTR: Number of unaffected individuals.

Table S7. Meta-analysis results and analysis of directionality for significant AI alterations that arose in primary case-control analysis, here with outlier datasets removed.

Hemisphere	Measurement	Region	Cohen's d	se	95% CI	z	p	Q	p_Q	I^2	df	N SZ	N CTR	Outlier datasets removed
AI	Thickness	Rostral anterior cingulate cortex	-0.073	0.021	[-0.114, -0.032]	-3.51	4.45E-04	38.9	5.64E-01	0	41	4490	4802	CLING, FOR2107_Muenster, OLIN
Left	Thickness	Rostral anterior cingulate cortex	-0.17	0.037	[-0.242, -0.096]	-4.54	5.59E-06	103.9	2.27E-07	63	41	4490	4802	CLING, FOR2107_Muenster, OLIN
Right	Thickness	Rostral anterior cingulate cortex	-0.084	0.032	[-0.146, -0.022]	-2.66	7.76E-03	78.7	3.65E-04	48.6	41	4490	4802	CLING, FOR2107_Muenster, OLIN
AI	Thickness	Middle temporal gyrus	-0.079	0.023	[-0.124, -0.034]	-3.44	5.85E-04	52.6	1.26E-01	16.8	42	4518	5569	CIAM, IMH
Left	Thickness	Middle temporal gyrus	-0.39	0.046	[-0.484, -0.303]	-8.52	1.54E-17	166.7	8.99E-17	77.9	42	4518	5569	CIAM, IMH
Right	Thickness	Middle temporal gyrus	-0.34	0.046	[-0.432, -0.251]	-7.41	1.26E-13	174.6	4.20E-18	78	42	4518	5569	CIAM, IMH

For each region, the meta-analysis result with outlier datasets removed is shown (AI), as well as the separate effects on cortical thickness of the left and right hemisphere with the AI outlier datasets removed.

AI: Asymmetry index; Cohen's d: meta-analysis Cohen's d effect size; se: Standard error of Cohen's d effect size; CI: Confidence interval; z: Meta-analysis z-value; p: meta-analysis p-value; Q: Cochran's Q-statistic of between-study heterogeneity; pQ: p-value of Cochran's Q-statistic; I^2: Percentage of variation across studies due to heterogeneity; df: Degrees of freedom in meta-analysis; N SZ: Number of individuals affected with schizophrenia; N CTR: Number of unaffected individuals.

Table S8. Meta-analysis results of case-control differences for significant AI alterations that arose in primary case-control analysis, here using models with additional covariates.

Measurement	Region	Model	Cohen's d	se	95% CI	z	p	Q	p_Q	I^2	df	N SZ	N CTR
Thickness	Rostral anterior cingulate cortex	Primary	-0.083	0.026	[-0.134, -0.032]	-3.21	1.34E-03	67.6	1.27E-02	34.5	44	4851	5811
Thickness	Rostral anterior cingulate cortex	Primary + Handedness	-0.10	0.029	[-0.162, -0.048]	-3.6	3.16E-04	46.7	5.80E-02	29.4	33	3481	4222
Thickness	Rostral anterior cingulate cortex	Primary + ICV	-0.086	0.026	[-0.136, -0.035]	-3.32	8.93E-04	66.6	1.54E-02	33.8	44	4845	5811
Thickness	Rostral anterior cingulate cortex	Primary + Handedness + ICV	-0.11	0.029	[-0.163, -0.05]	-3.71	2.03E-04	46	6.58E-02	27.8	33	3478	4222
Thickness	Rostral anterior cingulate cortex	Primary + Age^2	-0.088	0.026	[-0.139, -0.037]	-3.37	7.60E-04	70.1	7.38E-03	35.3	44	4851	5811
Thickness	Middle temporal gyrus	Primary	-0.074	0.025	[-0.123, -0.026]	-2.99	2.75E-03	65.9	1.80E-02	27.7	44	4684	5673
Thickness	Middle temporal gyrus	Primary + Handedness	-0.074	0.030	[-0.133, -0.015]	-2.44	1.46E-02	52.7	1.61E-02	31.1	33	3318	4099
Thickness	Middle temporal gyrus	Primary + ICV	-0.075	0.025	[-0.124, -0.026]	-2.98	2.87E-03	67.2	1.36E-02	28.3	44	4678	5673
Thickness	Middle temporal gyrus	Primary + Handedness + ICV	-0.069	0.029	[-0.125, -0.012]	-2.36	1.84E-02	51.1	2.28E-02	26.8	33	3315	4099
Thickness	Middle temporal gyrus	Primary + Age^2	-0.067	0.023	[-0.112, -0.021]	-2.84	4.50E-03	61.8	3.94E-02	20.4	44	4684	5673

For each significantly different structural AI from the primary analysis, the meta-analysis result is shown ('Primary') as well as the meta-analysis results of the primary analysis model with additional covariates added.

Cohen's d: meta-analysis Cohen's d effect size; se: Standard error of Cohen's d effect size; CI: Confidence interval; z: Meta-analysis z-value; p: meta-analysis p-value; Q: Cochran's Q-statistic of between-study heterogeneity; p_Q: p-value of Cochran's Q-statistic; I^2: Percentage of variation across studies due to heterogeneity; df: Degrees of freedom in meta-analysis; N SZ: Number of individuals affected with schizophrenia; N CTR: Number of unaffected individuals.

Table S9. Meta-analysis results of antipsychotic medication group differences, for AIs that showed alterations in primary case-control analysis.

Region	Group 1	Group 2	Cohen's d	se	95% CI	z	p	p_{FDR}	Q	p_Q	I^2	df	N Group 1	N Group 2
Rostral anterior cingulate cortex	First-generation antipsychotics	Unmedicated	0.072	0.097	[-0.117, 0.261]	0.74	4.57E-01	7.83E-01	8.6	4.77E-01	0	9	268	186
Rostral anterior cingulate cortex	Second-generation antipsychotics	Unmedicated	0.021	0.065	[-0.107, 0.148]	0.32	7.52E-01	8.97E-01	26.2	7.08E-02	38.5	17	1382	365
Rostral anterior cingulate cortex	First- and second-generation antipsychotics	Unmedicated	0.014	0.12	[-0.22, 0.249]	0.12	9.04E-01	9.04E-01	10.3	2.46E-01	0	8	146	157
Rostral anterior cingulate cortex	First-generation antipsychotics	Second-generation antipsychotics	0.016	0.072	[-0.125, 0.157]	0.22	8.22E-01	8.97E-01	41.7	1.21E-03	60	18	458	1789
Rostral anterior cingulate cortex	First- and second-generation antipsychotics	First-generation antipsychotics	-0.18	0.146	[-0.464, 0.109]	-1.22	2.24E-01	5.38E-01	28.7	4.33E-03	59.8	12	213	392
Rostral anterior cingulate cortex	First- and second-generation antipsychotics	Second-generation antipsychotics	-0.070	0.119	[-0.304, 0.164]	-0.59	5.57E-01	8.36E-01	61	1.70E-07	79.1	15	234	1312
Middle temporal gyrus	First-generation antipsychotics	Unmedicated	-0.14	0.159	[-0.451, 0.171]	-0.88	8.10E-01	8.97E-01	19.2	2.37E-02	54.8	9	256	176
Middle temporal gyrus	Second-generation antipsychotics	Unmedicated	-0.097	0.075	[-0.244, 0.05]	-1.3	1.94E-01	5.38E-01	34.7	6.72E-03	49.6	17	1259	355
Middle temporal gyrus	First- and second-generation antipsychotics	Unmedicated	-0.21	0.13	[-0.467, 0.041]	-1.64	1.00E-01	4.24E-01	4	7.81E-01	0	7	133	125
Middle temporal gyrus	First-generation antipsychotics	Second-generation antipsychotics	-0.21	0.081	[-0.365, -0.048]	-2.56	1.06E-02	1.27E-01	52.3	3.41E-05	65.8	18	445	1673
Middle temporal gyrus	First- and second-generation antipsychotics	First-generation antipsychotics	0.092	0.111	[-0.127, 0.31]	0.82	4.11E-01	7.83E-01	16.4	1.27E-01	31.8	11	204	380
Middle temporal gyrus	First- and second-generation antipsychotics	Second-generation antipsychotics	-0.11	0.067	[-0.239, 0.023]	-1.61	1.06E-01	4.24E-01	22.8	8.76E-02	29.5	15	225	1198

For each significantly different structural AI from the primary analysis, meta-analysis results of between medication-group comparisons in affected individuals are shown. The effect size reflects the effect of group 1 compared to group 2.

Cohen's d: meta-analysis Cohen's d effect size; se: Standard error of Cohen's d effect size; CI: Confidence interval; z: Meta-analysis z-value; p: meta-analysis p-value; p_{FDR}: FDR-corrected p-value; Q: Cochran's Q-statistic of between-study heterogeneity; p_Q: p-value of Cochran's Q-statistic; I^2: Percentage of variation across studies due to heterogeneity; df: Degrees of freedom in meta-analysis; N Group 1: Number of individuals in first medication group; N Group 2: Number of individuals in second medication group.

Table S10A. Meta-analysis results of partial correlations between rostral anterior cingulate thickness AI and schizophrenia-specific variables.

Variable	r	se	95% CI	z	p	p_{FDR}	Q	p_Q	I^2	df	N
Chlorpromazine equivalent medication dose	-0.016	0.021	[-0.058, 0.025]	-0.77	4.43E-01	6.44E-01	48.5	1.77E-02	12.4	30	2688
Age at onset	-0.0032	0.017	[-0.036, 0.029]	-0.19	8.46E-01	9.03E-01	23.9	9.39E-01	0	36	3661
Duration of illness	0.0032	0.016	[-0.029, 0.035]	0.19	8.47E-01	9.03E-01	27.5	8.45E-01	0	36	3679
PANSS - Total score	0.046	0.030	[-0.014, 0.105]	1.51	1.30E-01	3.47E-01	53.8	2.39E-03	44.8	28	2236
PANSS - Positive symptom score	0.042	0.027	[-0.011, 0.095]	1.56	1.18E-01	3.47E-01	48.1	1.45E-02	31.9	29	2288
PANSS - Negative symptom score	0.016	0.026	[-0.034, 0.066]	0.61	5.41E-01	7.21E-01	42.5	5.10E-02	25.4	29	2290
SAPS - Total score	0.00070	0.030	[-0.057, 0.059]	0.02	9.81E-01	9.81E-01	7.3	7.01E-01	0	10	1140
SANS - Total score	0.049	0.024	[0.003, 0.096]	2.08	3.77E-02	3.20E-01	12.4	6.46E-01	0.4	15	1769

r: meta-analysis partial correlation coefficient; se: Standard error of partial correlation coefficient; CI: Confidence interval; z: Meta-analysis z-value; p: meta-analysis p-value; p_{FDR}: FDR-corrected p-value; Q: Cochran's Q-statistic of between-study heterogeneity; p_Q: p-value of Cochran's Q-statistic; I^2: Percentage of variation across studies due to heterogeneity; df: Degrees of freedom in meta-analysis; N: Sample size

Table S10B. Meta-analysis results of partial correlations between middle temporal gyrus thickness AI and schizophrenia-specific variables.

Variable	r	se	95% CI	z	p	p_{FDR}	Q	p_Q	I^2	df	N
Chlorpromazine equivalent medication dose	-0.030	0.032	[-0.092, 0.032]	-0.94	3.46E-01	6.15E-01	64	2.96E-04	57.7	30	2658
Age at onset	0.045	0.024	[-0.002, 0.092]	1.86	6.30E-02	3.20E-01	67.4	1.15E-03	44.3	36	3500
Duration of illness	-0.048	0.024	[-0.095, 0]	-1.97	4.91E-02	3.20E-01	68.8	7.98E-04	45.2	36	3519
PANSS - Total score	0.026	0.025	[-0.022, 0.075]	1.06	2.87E-01	5.74E-01	33.5	2.19E-01	18.9	28	2196
PANSS - Positive symptom score	0.029	0.026	[-0.021, 0.079]	1.13	2.57E-01	5.74E-01	41.8	5.79E-02	25	29	2247
PANSS - Negative symptom score	0.0046	0.021	[-0.037, 0.046]	0.22	8.28E-01	9.03E-01	24.4	7.08E-01	0	29	2249
SAPS - Total score	0.029	0.037	[-0.043, 0.1]	0.78	4.37E-01	6.44E-01	12.7	2.39E-01	28	10	1109
SANS - Total score	-0.043	0.025	[-0.092, 0.005]	-1.75	7.99E-02	3.20E-01	9.5	8.47E-01	0	15	1624

r: meta-analysis partial correlation coefficient; se: Standard error of partial correlation coefficient; CI: Confidence interval; z: Meta-analysis z-value; p: meta-analysis p-value; p_{FDR}: FDR-corrected p-value; Q: Cochran's Q-statistic of between-study heterogeneity; p_Q: p-value of Cochran's Q-statistic; I^2: Percentage of variation across studies due to heterogeneity; df: Degrees of freedom in meta-analysis; N: Sample size

Table S11A. Meta-analysis results of diagnosis-by-age effects for cortical thickness AIs.

Region	Cohen's d	se	95% CI	z	p	p_{FDR}	Q	p_Q	I^2	df	N SZ	N CTR
Banks of superior temporal sulcus	-0.017	0.020	[-0.056, 0.021]	-0.88	3.77E-01	8.83E-01	37.3	7.52E-01	0	44	4721	5670
Caudal anterior cingulate cortex	-0.041	0.030	[-0.099, 0.018]	-1.35	1.76E-01	8.83E-01	85.2	1.92E-04	50.5	44	4893	5849
Caudal middle frontal gyrus	0.058	0.027	[0.005, 0.11]	2.17	3.03E-02	5.31E-01	69.7	8.13E-03	37.9	44	4870	5844
Cuneus	-0.0031	0.021	[-0.043, 0.037]	-0.15	8.81E-01	9.69E-01	41.3	5.86E-01	6	44	4798	5785
Entorhinal cortex	0.012	0.02	[-0.027, 0.051]	0.6	5.51E-01	9.65E-01	70.1	7.40E-03	0	44	4639	5564
Fusiform gyrus	-0.023	0.025	[-0.073, 0.026]	-0.91	3.61E-01	8.83E-01	63.9	2.65E-02	31.5	44	4794	5802
Inferior parietal cortex	0.029	0.020	[-0.01, 0.068]	1.46	1.43E-01	8.83E-01	39.7	6.57E-01	0	44	4654	5695
Inferior temporal gyrus	0.0055	0.020	[-0.034, 0.045]	0.27	7.84E-01	9.69E-01	54.8	1.28E-01	2.7	44	4771	5761
Isthmus cingulate cortex	0.0063	0.022	[-0.037, 0.05]	0.28	7.79E-01	9.69E-01	48.3	3.02E-01	17.3	44	4906	5875
Lateral occipital cortex	0.0011	0.027	[-0.052, 0.054]	0.04	9.69E-01	9.69E-01	74	3.11E-03	39.3	44	4795	5819
Lateral orbitofrontal cortex	0.0025	0.024	[-0.044, 0.049]	0.11	9.16E-01	9.69E-01	54.2	1.40E-01	23.9	44	4893	5870
Lingual gyrus	-0.016	0.023	[-0.061, 0.029]	-0.69	4.92E-01	9.36E-01	56.7	9.41E-02	20	44	4889	5843
Medial orbitofrontal cortex	0.0017	0.019	[-0.036, 0.04]	0.09	9.31E-01	9.69E-01	32.5	8.99E-01	0	44	4863	5825
Middle temporal gyrus	-0.026	0.020	[-0.064, 0.013]	-1.3	1.93E-01	8.83E-01	58	7.66E-02	0	44	4684	5673
Parahippocampal gyrus	-0.031	0.028	[-0.085, 0.023]	-1.13	2.57E-01	8.83E-01	77.1	1.48E-03	41.9	44	4863	5849
Paracentral lobule	0.0018	0.023	[-0.044, 0.048]	0.08	9.38E-01	9.69E-01	59.1	6.37E-02	23.5	44	4913	5871
Pars opercularis of inferior frontal gyrus	0.020	0.022	[-0.023, 0.062]	0.92	3.60E-01	8.83E-01	47.4	3.37E-01	12.7	44	4832	5824
Pars orbitalis of inferior frontal gyrus	-0.019	0.024	[-0.066, 0.028]	-0.79	4.29E-01	8.83E-01	57.4	8.46E-02	25.9	44	4861	5838
Pars triangularis of inferior frontal gyrus	-0.016	0.020	[-0.054, 0.022]	-0.82	4.13E-01	8.83E-01	38.8	6.93E-01	0	44	4818	5807
Pericalcarine cortex	0.013	0.019	[-0.025, 0.051]	0.66	5.08E-01	9.36E-01	44	4.70E-01	0	44	4902	5833
Postcentral gyrus	0.025	0.030	[-0.033, 0.084]	0.85	3.93E-01	8.83E-01	84.4	2.40E-04	49	44	4793	5770
Posterior cingulate cortex	-0.0010	0.023	[-0.046, 0.044]	-0.04	9.67E-01	9.69E-01	56.3	1.01E-01	21.2	44	4916	5880
Precentral gyrus	0.019	0.020	[-0.019, 0.057]	0.98	3.26E-01	8.83E-01	31.8	9.14E-01	0	44	4808	5807
Precuneus	-0.032	0.023	[-0.078, 0.013]	-1.39	1.63E-01	8.83E-01	59.1	6.40E-02	21.5	44	4868	5864
Rostral anterior cingulate cortex	-0.029	0.028	[-0.084, 0.026]	-1.04	2.99E-01	8.83E-01	78.5	1.05E-03	43.9	44	4851	5811
Rostral middle frontal gyrus	-0.013	0.027	[-0.066, 0.041]	-0.46	6.42E-01	9.69E-01	72.2	4.70E-03	40.2	44	4806	5811
Superior frontal gyrus	0.022	0.020	[-0.018, 0.061]	1.07	2.82E-01	8.83E-01	49.2	2.74E-01	3.2	44	4854	5833
Superior parietal cortex	0.048	0.020	[0.009, 0.086]	2.44	1.49E-02	5.21E-01	43.6	4.89E-01	0	44	4734	5766
Superior temporal gyrus	-0.0034	0.021	[-0.045, 0.038]	-0.16	8.73E-01	9.69E-01	65	2.12E-02	7.6	44	4622	5596
Supramarginal gyrus	0.0058	0.025	[-0.043, 0.055]	0.23	8.17E-01	9.69E-01	65.9	1.79E-02	27.1	44	4606	5613
Frontal pole	0.00080	0.019	[-0.037, 0.039]	0.04	9.68E-01	9.69E-01	33.4	8.77E-01	0	44	4932	5880
Temporal pole	-0.030	0.023	[-0.075, 0.014]	-1.34	1.80E-01	8.83E-01	50.3	2.37E-01	17.7	44	4765	5765
Transverse temporal gyrus	0.0047	0.019	[-0.033, 0.043]	0.24	8.11E-01	9.69E-01	46.5	3.69E-01	0.4	44	4923	5880
Insula	0.015	0.029	[-0.041, 0.072]	0.53	5.98E-01	9.69E-01	81.2	5.50E-04	46.2	44	4920	5807
Overall	0.0043	0.019	[-0.034, 0.042]	0.22	8.24E-01	9.69E-01	40.9	6.04E-01	0.8	44	4945	5915

Cohen's d: meta-analysis Cohen's d effect size; se: Standard error of Cohen's d effect size; CI: Confidence interval; z: Meta-analysis z-value; p: meta-analysis p-value; p_{FDR}: FDR-corrected p-value; Q: Cochran's Q-statistic of between-study heterogeneity; p_Q: p-value of Cochran's Q-statistic; I^2: Percentage of variation across studies due to heterogeneity; df: Degrees of freedom in meta-analysis; N SZ: Number of individuals affected with schizophrenia; N CTR: Number of unaffected individuals.

Table S11B. Meta-analysis results of diagnosis-by-age effects for cortical surface area AIs.

Region	Cohen's d	se	95% CI	z	p	p_{FDR}	Q	p_Q	I^2	df	N SZ	N CTR
Banks of superior temporal sulcus	-0.052	0.020	[-0.091, -0.013]	-2.64	8.36E-03	2.93E-01	45.9	3.92E-01	0	44	4719	5667
Caudal anterior cingulate cortex	-0.043	0.023	[-0.088, 0.001]	-1.9	5.78E-02	4.05E-01	58	7.62E-02	19.2	44	4892	5846
Caudal middle frontal gyrus	-0.019	0.030	[-0.078, 0.041]	-0.61	5.40E-01	9.62E-01	87.5	1.05E-04	51.2	44	4865	5840
Cuneus	-0.030	0.027	[-0.083, 0.024]	-1.08	2.79E-01	7.40E-01	75.1	2.41E-03	40.4	44	4796	5781
Entorhinal cortex	0.0082	0.021	[-0.032, 0.049]	0.4	6.90E-01	9.62E-01	53.8	1.49E-01	4	44	4637	5560
Fusiform gyrus	0.0047	0.020	[-0.034, 0.043]	0.24	8.09E-01	9.62E-01	38.5	7.07E-01	0	44	4793	5796
Inferior parietal cortex	-0.0035	0.022	[-0.046, 0.039]	-0.16	8.72E-01	9.62E-01	51	2.17E-01	11.1	44	4656	5691
Inferior temporal gyrus	0.0024	0.023	[-0.042, 0.047]	0.1	9.18E-01	9.62E-01	64.1	2.54E-02	18.6	44	4769	5758
Isthmus cingulate cortex	0.049	0.023	[0.004, 0.095]	2.14	3.25E-02	3.95E-01	70.3	7.18E-03	21.5	44	4905	5871
Lateral occipital cortex	0.035	0.020	[-0.004, 0.073]	1.77	7.63E-02	4.44E-01	50.7	2.26E-01	0.1	44	4793	5814
Lateral orbitofrontal cortex	0.015	0.032	[-0.048, 0.077]	0.46	6.43E-01	9.62E-01	96.1	9.62E-06	56	44	4894	5865
Lingual gyrus	-0.039	0.019	[-0.077, -0.001]	-2	4.51E-02	3.95E-01	40.1	6.41E-01	0.3	44	4885	5840
Medial orbitofrontal cortex	0.025	0.019	[-0.013, 0.063]	1.29	1.95E-01	7.40E-01	50.5	2.32E-01	0	44	4861	5824
Middle temporal gyrus	-0.030	0.022	[-0.073, 0.012]	-1.4	1.63E-01	7.11E-01	46.8	3.59E-01	11.6	44	4681	5668
Parahippocampal gyrus	-0.0030	0.019	[-0.041, 0.035]	-0.16	8.75E-01	9.62E-01	37.8	7.33E-01	0	44	4856	5845
Paracentral lobule	0.0055	0.022	[-0.038, 0.049]	0.25	8.05E-01	9.62E-01	57.1	8.88E-02	16.8	44	4910	5868
Pars opercularis of inferior frontal gyrus	0.017	0.029	[-0.04, 0.073]	0.58	5.59E-01	9.62E-01	79.1	9.11E-04	46	44	4830	5818
Pars orbitalis of inferior frontal gyrus	-0.0028	0.023	[-0.047, 0.041]	-0.12	9.01E-01	9.62E-01	58.2	7.37E-02	18	44	4861	5835
Pars triangularis of inferior frontal gyrus	-0.0037	0.021	[-0.046, 0.038]	-0.18	8.61E-01	9.62E-01	56.1	1.05E-01	10.6	44	4817	5801
Pericalcarine cortex	-0.035	0.029	[-0.091, 0.022]	-1.21	2.27E-01	7.40E-01	80.2	7.00E-04	46.3	44	4898	5831
Postcentral gyrus	0.020	0.022	[-0.023, 0.063]	0.93	3.53E-01	7.72E-01	58.3	7.30E-02	13.7	44	4792	5766
Posterior cingulate cortex	0.022	0.021	[-0.019, 0.064]	1.04	2.96E-01	7.40E-01	54.7	1.29E-01	10.6	44	4914	5873
Precentral gyrus	-0.048	0.024	[-0.095, -0.002]	-2.04	4.09E-02	3.95E-01	58	7.62E-02	23.4	44	4807	5800
Precuneus	0.0035	0.025	[-0.045, 0.052]	0.14	8.86E-01	9.62E-01	62.7	3.33E-02	29.5	44	4869	5862
Rostral anterior cingulate cortex	0.0015	0.024	[-0.046, 0.049]	0.06	9.50E-01	9.62E-01	67.1	1.39E-02	26.9	44	4849	5807
Rostral middle frontal gyrus	0.0050	0.036	[-0.066, 0.076]	0.14	8.90E-01	9.62E-01	122	2.95E-09	65.4	44	4803	5808
Superior frontal gyrus	0.028	0.025	[-0.021, 0.077]	1.12	2.64E-01	7.40E-01	64.9	2.19E-02	31	44	4851	5830
Superior parietal cortex	-0.0052	0.028	[-0.06, 0.05]	-0.19	8.52E-01	9.62E-01	79.7	7.98E-04	43.1	44	4734	5763
Superior temporal gyrus	0.010	0.033	[-0.055, 0.075]	0.3	7.61E-01	9.62E-01	106	5.84E-07	57.1	44	4625	5591
Supramarginal gyrus	0.020	0.020	[-0.02, 0.06]	0.99	3.22E-01	7.51E-01	42.1	5.55E-01	2.5	44	4607	5608
Frontal pole	-0.023	0.022	[-0.066, 0.02]	-1.05	2.95E-01	7.40E-01	57.6	8.21E-02	14.5	44	4927	5878
Temporal pole	0.0088	0.026	[-0.043, 0.06]	0.33	7.38E-01	9.62E-01	67.9	1.19E-02	35.2	44	4765	5763
Transverse temporal gyrus	0.033	0.019	[-0.005, 0.071]	1.7	8.88E-02	4.44E-01	40.5	6.22E-01	0	44	4920	5873
Insula	-0.0012	0.025	[-0.051, 0.049]	-0.05	9.62E-01	9.62E-01	68.8	9.73E-03	32.4	44	4918	5801
Overall	-0.0017	0.020	[-0.04, 0.037]	-0.09	9.32E-01	9.62E-01	38.6	6.63E-01	0	43	4856	5730

Cohen's d: meta-analysis Cohen's d effect size; se: Standard error of Cohen's d effect size; CI: Confidence interval; z: Meta-analysis z-value; p: meta-analysis p-value; p_{FDR}: FDR-corrected p-value; Q: Cochran's Q-statistic of between-study heterogeneity; p_Q: p-value of Cochran's Q-statistic; I^2: Percentage of variation across studies due to heterogeneity; df: Degrees of freedom in meta-analysis; N SZ: Number of individuals affected with schizophrenia; N CTR: Number of unaffected individuals.

Table S11C. Meta-analysis results of diagnosis-by-age effects for subcortical volume AIs.

Region	Cohen's d	se	95% CI	z	p	p_{FDR}	Q	p_Q	I^2	df	N SZ	N CTR
Lateral Ventricles	-0.0026	0.031	[-0.064, 0.058]	-0.08	9.34E-01	9.34E-01	87.7	6.80E-05	53.7	43	4829	5770
Thalamus	0.016	0.022	[-0.027, 0.059]	0.73	4.62E-01	7.40E-01	52.8	1.46E-01	12.4	43	4746	5718
Caudate Nucelus	-0.0038	0.023	[-0.05, 0.042]	-0.16	8.70E-01	9.34E-01	58.3	5.93E-02	22	43	4748	5709
Putamen	-0.023	0.022	[-0.066, 0.021]	-1.01	3.15E-01	6.29E-01	48.6	2.93E-01	17.9	44	4992	5758
Pallidum	0.081	0.025	[0.032, 0.129]	3.26	1.13E-03	9.00E-03	64.5	2.35E-02	29.8	44	4964	5687
Hippocampus	0.027	0.025	[-0.022, 0.076]	1.08	2.79E-01	6.29E-01	64.9	2.17E-02	31	44	4973	5771
Amygdala	-0.058	0.026	[-0.109, -0.006]	-2.2	2.76E-02	1.10E-01	72	4.84E-03	36.8	44	4990	5784
Accumbens	0.0057	0.022	[-0.037, 0.048]	0.26	7.93E-01	9.34E-01	57.1	8.91E-02	14.1	44	4966	5775

Cohen's d: meta-analysis Cohen's d effect size; se: Standard error of Cohen's d effect size; CI: Confidence interval; z: Meta-analysis z-value; p: meta-analysis p-value; p_{FDR}: FDR-corrected p-value; Q: Cochran's Q-statistic of between-study heterogeneity; p_Q: p-value of Cochran's Q-statistic; I^2: Percentage of variation across studies due to heterogeneity; df: Degrees of freedom in meta-analysis; N SZ: Number of individuals affected with schizophrenia; N CTR: Number of unaffected individuals.

Table S12A. Analysis of directionality for pallidum volume AI diagnosis-by-age effect.

Hemisphere	Cohen's d	se	95% CI	z	p	p_{FDR}	Q	p_Q	I^2	df	N SZ	N CTR
AI	0.081	0.025	[0.032, 0.129]	3.26	1.13E-03	9.00E-03	64.5	2.35E-02	29.8	44	4964	5687
Left	0.073	0.029	[0.016, 0.129]	2.53	1.14E-02		85.5	1.78E-04	46.3	44	4964	5687
Right	0.022	0.030	[-0.036, 0.08]	0.74	4.58E-01		90.2	5.02E-05	49	44	4964	5687

For pallidum volume, the diagnosis-by-age meta-analysis results for asymmetry is shown, as well as the bilateral meta-analysis results.

AI: Asymmetry index; Cohen's d: meta-analysis Cohen's d effect size; se: Standard error of Cohen's d effect size; CI: Confidence interval; z: Meta-analysis z-value; p: meta-analysis p-value; p_{FDR}: FDR-corrected p-value; Q: Cochran's Q-statistic of between-study heterogeneity; p_Q: p-value of Cochran's Q-statistic; I^2: Percentage of variation across studies due to heterogeneity; df: Degrees of freedom in meta-analysis; N SZ: Number of individuals affected with schizophrenia; N CTR: Number of unaffected individuals.

Table S12B. Meta-analysis results of correlations between pallidum volume and age in cases and controls.

Individuals	Hemisphere	r	se	95% CI	z	p	Q	p_Q	I^2	df	N
Cases	AI	0.011	0.022	[-0.032, 0.053]	0.48	6.30E-01	90.7	4.32E-05	52.6	44	5001
Cases	Left	-0.17	0.030	[-0.232, -0.115]	-5.86	4.73E-09	167.3	2.87E-16	77	44	5001
Cases	Right	-0.20	0.022	[-0.245, -0.16]	-9.42	4.73E-21	87.6	7.02E-05	52.1	43	5001
Controls	AI	-0.077	0.023	[-0.123, -0.031]	-3.28	1.05E-03	116.1	2.05E-08	63.4	44	5687
Controls	Left	-0.27	0.027	[-0.319, -0.212]	-9.74	2.14E-22	165.5	5.56E-16	78	44	5687
Controls	Right	-0.24	0.029	[-0.297, -0.184]	-8.36	6.18E-17	196.6	3.46E-21	80.1	44	5687

For pallidum volume, the correlation of age with volume asymmetry and bilateral volume measures is shown in cases and controls.

AI: Asymmetry index; r: meta-analysis partial correlation coefficient; se: Standard error of partial correlation coefficient; CI: Confidence interval; z: Meta-analysis z-value; p: meta-analysis p-value; Q: Cochran's Q-statistic of between-study heterogeneity; p_Q: p-value of Cochran's Q-statistic; I^2: Percentage of variation across studies due to heterogeneity; df: Degrees of freedom in meta-analysis; N: Sample size

Table S13A. Meta-analysis results of diagnosis-by-sex effects for cortical thickness AIs.

Region	Cohen's d	SE	CI	z	p	p_{FDR}	Q	p_Q	I^2	df	N SZ	N CTR
Banks of superior temporal sulcus	-0.019	0.026	[-0.071, 0.033]	-0.73	4.67E-01	9.57E-01	66.1	1.34E-02	35.1	43	4682	5622
Caudal anterior cingulate cortex	0.0043	0.019	[-0.034, 0.042]	0.22	8.26E-01	9.63E-01	38.8	6.54E-01	0	43	4848	5797
Caudal middle frontal gyrus	0.023	0.024	[-0.023, 0.069]	0.97	3.34E-01	9.57E-01	56	8.82E-02	23.3	43	4824	5792
Cuneus	0.011	0.021	[-0.03, 0.051]	0.51	6.12E-01	9.57E-01	47.2	3.05E-01	6.9	43	4752	5733
Entorhinal cortex	0.0071	0.020	[-0.033, 0.047]	0.35	7.30E-01	9.57E-01	46.1	3.45E-01	2.8	43	4619	5534
Fusiform gyrus	0.025	0.021	[-0.016, 0.067]	1.2	2.30E-01	9.57E-01	53.6	1.29E-01	8.8	43	4750	5750
Inferior parietal cortex	0.013	0.023	[-0.033, 0.059]	0.56	5.75E-01	9.57E-01	54.2	1.18E-01	19.2	43	4608	5643
Inferior temporal gyrus	-0.022	0.023	[-0.066, 0.023]	-0.96	3.37E-01	9.57E-01	65.6	1.49E-02	16.3	43	4725	5709
Isthmus cingulate cortex	0.0088	0.026	[-0.041, 0.059]	0.35	7.30E-01	9.57E-01	64.8	1.72E-02	33.1	43	4860	5823
Lateral occipital cortex	0.010	0.027	[-0.042, 0.062]	0.38	7.03E-01	9.57E-01	70.3	5.35E-03	36.6	43	4749	5767
Lateral orbitofrontal cortex	0.024	0.024	[-0.023, 0.071]	1.01	3.12E-01	9.57E-01	59.8	4.60E-02	24.6	43	4847	5818
Lingual gyrus	-0.0084	0.022	[-0.052, 0.035]	-0.38	7.04E-01	9.57E-01	55.8	9.11E-02	15.1	43	4843	5791
Medial orbitofrontal cortex	-0.0058	0.022	[-0.049, 0.037]	-0.26	7.93E-01	9.57E-01	48.6	2.57E-01	14.5	43	4817	5773
Middle temporal gyrus	-0.021	0.020	[-0.06, 0.018]	-1.05	2.93E-01	9.57E-01	40.3	5.89E-01	0	43	4641	5623
Parahippocampal gyrus	0.013	0.027	[-0.04, 0.066]	0.47	6.38E-01	9.57E-01	67.9	9.01E-03	39	43	4818	5797
Paracentral lobule	0.0017	0.023	[-0.043, 0.046]	0.08	9.40E-01	9.69E-01	64.9	1.71E-02	18.6	43	4867	5819
Pars opercularis of inferior frontal gyrus	-0.019	0.027	[-0.072, 0.033]	-0.72	4.71E-01	9.57E-01	70.1	5.67E-03	38.3	43	4786	5772
Pars orbitalis of inferior frontal gyrus	0.021	0.020	[-0.017, 0.059]	1.08	2.82E-01	9.57E-01	44	4.31E-01	0.1	43	4815	5786
Pars triangularis of inferior frontal gyrus	0.0079	0.020	[-0.031, 0.047]	0.4	6.92E-01	9.57E-01	45.1	3.85E-01	2.1	43	4772	5755
Pericalcarine cortex	0.017	0.023	[-0.029, 0.063]	0.73	4.66E-01	9.57E-01	53.5	1.31E-01	22.4	43	4856	5782
Postcentral gyrus	0.0054	0.020	[-0.033, 0.044]	0.27	7.83E-01	9.57E-01	40.4	5.85E-01	0	43	4748	5718
Posterior cingulate cortex	0.044	0.028	[-0.01, 0.098]	1.59	1.13E-01	9.57E-01	74.3	2.16E-03	41.6	43	4870	5828
Precentral gyrus	-0.0061	0.022	[-0.049, 0.037]	-0.28	7.80E-01	9.57E-01	47.9	2.81E-01	13.9	43	4762	5755
Precuneus	-0.044	0.022	[-0.087, -0.001]	-2	4.54E-02	9.57E-01	55.4	9.68E-02	13.9	43	4822	5812
Rostral anterior cingulate cortex	-0.0091	0.020	[-0.047, 0.029]	-0.46	6.42E-01	9.57E-01	44.1	4.25E-01	0	43	4805	5759
Rostral middle frontal gyrus	-0.031	0.025	[-0.08, 0.019]	-1.21	2.27E-01	9.57E-01	58.9	5.40E-02	31	43	4760	5759
Superior frontal gyrus	-0.00070	0.020	[-0.039, 0.038]	-0.04	9.72E-01	9.72E-01	37.9	6.91E-01	0	43	4808	5781
Superior parietal cortex	-0.014	0.022	[-0.057, 0.029]	-0.63	5.28E-01	9.57E-01	58.8	5.43E-02	13.7	43	4689	5714
Superior temporal gyrus	0.027	0.023	[-0.019, 0.072]	1.13	2.57E-01	9.57E-01	55.4	9.72E-02	18.8	43	4579	5545
Supramarginal gyrus	-0.024	0.028	[-0.078, 0.03]	-0.88	3.80E-01	9.57E-01	68.8	7.41E-03	38.2	43	4561	5561
Frontal pole	-0.0036	0.024	[-0.051, 0.044]	-0.15	8.84E-01	9.69E-01	67.1	1.08E-02	27.8	43	4886	5828
Temporal pole	-0.0026	0.027	[-0.056, 0.051]	-0.1	9.24E-01	9.69E-01	70.8	4.78E-03	39	43	4719	5713
Transverse temporal gyrus	0.035	0.019	[-0.004, 0.073]	1.78	7.55E-02	9.57E-01	40	6.02E-01	0.2	43	4877	5828
Insula	-0.020	0.022	[-0.063, 0.024]	-0.88	3.77E-01	9.57E-01	67.3	1.04E-02	14.7	43	4874	5755
Overall	0.0015	0.020	[-0.038, 0.041]	0.07	9.41E-01	9.69E-01	57.3	7.13E-02	4.1	43	4899	5863

Cohen's d: meta-analysis Cohen's d effect size; se: Standard error of Cohen's d effect size; CI: Confidence interval; z: Meta-analysis z-value; p: meta-analysis p-value; p_{FDR}: FDR-corrected p-value; Q: Cochran's Q-statistic of between-study heterogeneity; p_Q: p-value of Cochran's Q-statistic; I^2: Percentage of variation across studies due to heterogeneity; df: Degrees of freedom in meta-analysis; N SZ: Number of individuals affected with schizophrenia; N CTR: Number of unaffected individuals.

Table S13B. Meta-analysis results of diagnosis-by-sex effects for cortical surface area AIs.

Region	Cohen's d	SE	CI	z	p	p_{FDR}	Q	p_Q	I^2	df	N SZ	N CTR
Banks of superior temporal sulcus	0.0090	0.022	[-0.034, 0.052]	0.41	6.79E-01	9.78E-01	45.9	3.51E-01	11.3	43	4680	5619
Caudal anterior cingulate cortex	-0.021	0.019	[-0.059, 0.017]	-1.09	2.75E-01	9.44E-01	28.6	9.54E-01	0	43	4847	5794
Caudal middle frontal gyrus	0.0056	0.022	[-0.037, 0.048]	0.26	7.94E-01	9.78E-01	54.6	1.11E-01	11.9	43	4819	5788
Cuneus	-0.029	0.025	[-0.079, 0.021]	-1.15	2.50E-01	9.44E-01	62.5	2.77E-02	31.6	43	4750	5729
Entorhinal cortex	-0.010	0.020	[-0.05, 0.029]	-0.52	6.02E-01	9.78E-01	43.8	4.39E-01	0	43	4617	5530
Fusiform gyrus	0.010	0.024	[-0.036, 0.057]	0.42	6.72E-01	9.78E-01	64.7	1.79E-02	23.2	43	4749	5744
Inferior parietal cortex	0.013	0.023	[-0.031, 0.058]	0.59	5.58E-01	9.78E-01	49.7	2.23E-01	16.5	43	4610	5639
Inferior temporal gyrus	-0.0026	0.021	[-0.045, 0.039]	-0.12	9.03E-01	9.78E-01	45.3	3.76E-01	10.3	43	4723	5706
Isthmus cingulate cortex	-0.035	0.023	[-0.079, 0.009]	-1.54	1.23E-01	8.54E-01	65.8	1.43E-02	18.1	43	4859	5819
Lateral occipital cortex	-0.032	0.022	[-0.075, 0.011]	-1.45	1.46E-01	8.54E-01	50.8	1.92E-01	14.2	43	4747	5762
Lateral orbitofrontal cortex	0.011	0.027	[-0.042, 0.064]	0.41	6.82E-01	9.78E-01	72.6	3.15E-03	38.9	43	4848	5813
Lingual gyrus	-0.016	0.021	[-0.057, 0.025]	-0.76	4.45E-01	9.74E-01	50	2.15E-01	9.4	43	4839	5788
Medial orbitofrontal cortex	-0.016	0.020	[-0.054, 0.022]	-0.81	4.15E-01	9.69E-01	44.2	4.21E-01	0	43	4815	5772
Middle temporal gyrus	-0.023	0.023	[-0.068, 0.023]	-0.98	3.27E-01	9.53E-01	57.2	7.25E-02	18.3	43	4638	5618
Parahippocampal gyrus	-0.026	0.024	[-0.073, 0.022]	-1.05	2.94E-01	9.44E-01	60.4	4.10E-02	26.6	43	4811	5793
Paracentral lobule	0.023	0.026	[-0.027, 0.073]	0.89	3.72E-01	9.69E-01	69.5	6.35E-03	34	43	4864	5816
Pars opercularis of inferior frontal gyrus	0.00020	0.023	[-0.044, 0.044]	0.01	9.93E-01	9.93E-01	60.1	4.33E-02	17.3	43	4784	5766
Pars orbitalis of inferior frontal gyrus	-0.048	0.022	[-0.091, -0.006]	-2.23	2.56E-02	4.48E-01	50.3	2.06E-01	12.5	43	4815	5783
Pars triangularis of inferior frontal gyrus	-0.055	0.021	[-0.096, -0.014]	-2.62	8.71E-03	3.05E-01	44.2	4.21E-01	7.5	43	4771	5749
Pericalcarine cortex	0.0074	0.020	[-0.031, 0.046]	0.38	7.04E-01	9.78E-01	41.7	5.29E-01	0	43	4852	5780
Postcentral gyrus	-0.0028	0.020	[-0.041, 0.036]	-0.14	8.85E-01	9.78E-01	38	6.86E-01	0	43	4747	5714
Posterior cingulate cortex	0.0055	0.022	[-0.038, 0.049]	0.25	8.03E-01	9.78E-01	56.3	8.34E-02	15.1	43	4868	5821
Precentral gyrus	-0.016	0.020	[-0.055, 0.022]	-0.83	4.06E-01	9.69E-01	50.1	2.12E-01	0	43	4761	5748
Precuneus	-0.0026	0.020	[-0.041, 0.036]	-0.13	8.96E-01	9.78E-01	47.7	2.86E-01	1.8	43	4823	5810
Rostral anterior cingulate cortex	-0.010	0.022	[-0.054, 0.033]	-0.47	6.40E-01	9.78E-01	54	1.21E-01	16.1	43	4803	5755
Rostral middle frontal gyrus	0.035	0.033	[-0.03, 0.1]	1.04	2.97E-01	9.44E-01	99.4	2.33E-06	58.7	43	4757	5756
Superior frontal gyrus	0.0012	0.020	[-0.037, 0.04]	0.06	9.50E-01	9.78E-01	41	5.61E-01	0	43	4805	5778
Superior parietal cortex	-0.0041	0.026	[-0.056, 0.047]	-0.15	8.77E-01	9.78E-01	69.6	6.21E-03	34.9	43	4689	5711
Superior temporal gyrus	-0.043	0.024	[-0.091, 0.005]	-1.77	7.75E-02	6.85E-01	62.4	2.81E-02	23.9	43	4582	5540
Supramarginal gyrus	0.0050	0.020	[-0.034, 0.044]	0.25	8.03E-01	9.78E-01	40.5	5.82E-01	0	43	4562	5556
Frontal pole	0.035	0.020	[-0.004, 0.074]	1.76	7.83E-02	6.85E-01	57.3	7.10E-02	3.2	43	4881	5826
Temporal pole	-0.0015	0.023	[-0.047, 0.044]	-0.06	9.49E-01	9.78E-01	55.7	9.27E-02	20.5	43	4719	5711
Transverse temporal gyrus	0.0082	0.027	[-0.045, 0.061]	0.3	7.63E-01	9.78E-01	71.3	4.30E-03	40	43	4874	5821
Insula	0.0019	0.022	[-0.042, 0.045]	0.09	9.31E-01	9.78E-01	55.4	9.72E-02	15.8	43	4872	5749
Overall	-0.041	0.032	[-0.104, 0.022]	-1.27	2.02E-01	9.44E-01	93.9	7.74E-06	55.6	42	4810	5678

Cohen's d: meta-analysis Cohen's d effect size; se: Standard error of Cohen's d effect size; CI: Confidence interval; z: Meta-analysis z-value; p: meta-analysis p-value; p_{FDR}: FDR-corrected p-value; Q: Cochran's Q-statistic of between-study heterogeneity; p_Q: p-value of Cochran's Q-statistic; I^2: Percentage of variation across studies due to heterogeneity; df: Degrees of freedom in meta-analysis; N SZ: Number of individuals affected with schizophrenia; N CTR: Number of unaffected individuals.

Table S13C. Meta-analysis results of diagnosis-by-sex effects for subcortical volume AIs.

Region	Cohen's d	SE	CI	z	p	p_{FDR}	Q	p_Q	I^2	df	N SZ	N CTR
Lateral Ventricles	0.026	0.030	[-0.032, 0.085]	0.88	3.79E-01	6.06E-01	80.4	3.32E-04	49.6	42	4783	5718
Thalamus	0.043	0.020	[0.004, 0.081]	2.15	3.13E-02	2.21E-01	48.2	2.35E-01	0	42	4701	5666
Caudate Nucelus	0.017	0.025	[-0.032, 0.067]	0.68	4.98E-01	6.64E-01	63.5	1.77E-02	30.8	42	4702	5657
Putamen	-0.0072	0.023	[-0.052, 0.038]	-0.31	7.55E-01	8.32E-01	51.6	1.73E-01	20.6	43	4946	5706
Pallidum	-0.0044	0.021	[-0.045, 0.036]	-0.21	8.32E-01	8.32E-01	43.8	4.37E-01	7.6	43	4928	5647
Hippocampus	-0.044	0.023	[-0.089, 0.001]	-1.92	5.53E-02	2.21E-01	60.6	3.94E-02	20.1	43	4929	5720
Amygdala	-0.027	0.021	[-0.068, 0.015]	-1.25	2.10E-01	5.60E-01	46.9	3.15E-01	10.5	43	4944	5732
Accumbens	0.024	0.026	[-0.026, 0.075]	0.95	3.40E-01	6.06E-01	61.1	3.57E-02	33.8	43	4921	5725

Cohen's d: meta-analysis Cohen's d effect size; se: Standard error of Cohen's d effect size; CI: Confidence interval; z: Meta-analysis z-value; p: meta-analysis p-value; p_{FDR}: FDR-corrected p-value; Q: Cochran's Q-statistic of between-study heterogeneity; p_Q: p-value of Cochran's Q-statistic; I^2: Percentage of variation across studies due to heterogeneity; df: Degrees of freedom in meta-analysis; N SZ: Number of individuals affected with schizophrenia; N CTR: Number of unaffected individuals.

SI References

1. M. Harrer, P. Cuijpers, T. Furukawa, D.D. Ebert, dmetar: Companion R Package For The Guide 'Doing Meta-Analysis in R'. (2019).
2. S.R. Kay, A. Fiszbein, L.A. Opler, The positive and negative syndrome scale (PANSS) for schizophrenia. *Schizophr Bull* **13**, 261-276 (1987).
3. N.C. Andreasen, The scale for the assessment of positive symptoms (SAPS), (The University of Iowa, 1984).
4. N.C. Andreasen, The scale for the assessment of negative symptoms (SANS), (The University of Iowa, 1984).
5. S. Kim, ppcor: An R Package for a Fast Calculation to Semi-partial Correlation Coefficients. *Commun Stat Appl Methods* **22**, 665-674 (2015).
6. H. Wickham, ggplot2: Elegant Graphics for Data Analysis, (Springer-Verlag New York, 2016).
7. A. South, rnaturalearth: World Map Data from Natural Earth. R package version 0.1.0. https://CRAN.R-project.org/package=rnaturalearth. (2017).
8. E. Pebesma, Simple Features for R: Standardized Support for Spatial Vector Data. *R J* **10**, 439-446 (2018).
9. K. Slowikowski, ggrepel: Automatically Position Non-Overlapping Text Labels with 'ggplot2'. R package version 0.9.1. https://CRAN.R-project.org/package=ggrepel. (2021).
10. J. Radua *et al.*, Increased power by harmonizing structural MRI site differences with the ComBat batch adjustment method in ENIGMA. *Neuroimage* **218**, 116956 (2020).
11. T. Wei, V. Simko, R package 'corrplot': Visualization of a Correlation Matrix (Version 0.92). https://github.com/taiyun/corrplot. (2021).
